# Enantioselective vicinal olefin disulfonoxylation

**DOI:** 10.1016/j.tet.2026.135286

**Published:** 2026-04-11

**Authors:** Abhijit Manna, Raju Silver, Mintu Munda, Shyam Sathyamoorthi

**Affiliations:** Department of Medicinal Chemistry, University of Kansas, Lawrence, KS, 66047, USA

## Abstract

We disclose a new protocol for enantioselective, vicinal disulfonoxylation of terminal olefins. Our procedure is operationally simple and involves stirring olefin substrate with 1.2 equivalents of an enantiopure I(III) reagent and two equivalents of a sulfonic acid in 1,2-dichlorobenzene at 0 °C for 19 h. No special precautions are required to exclude air or ambient moisture. The reaction is scalable, many interesting functional groups survive, and the products are useful chirons.

Vicinal olefin dihydroxylation is a cornerstone transformation in organic chemistry [1,2]. After Sharpless and co-workers developed a general, enantioselective variant [3], the use of dihydroxylation chemistry skyrocketed, and this technology is now indispensable to multiple areas of chemical synthesis [4]. In many cases, after olefin dihydroxylation, the resulting alcohols are derivatized for further transformations. Common derivatives are mesylates and tosylates, which are active participants in a variety of substitution and elimination reactions. Thus, it logically follows that processes that allow for the direct transformation of olefins into vicinal dimesylates or ditosylates will be hugely advantageous from the perspective of step-economy. Building on pioneering contributions from Koser and co-workers [5,6], our laboratory recently developed and disclosed very convenient racemic protocols for the vicinal disulfonoxylation of olefins [7]. Our reactions utilized olefin as the limiting reagent (an advance from earlier protocols that essentially employed olefin as the reaction solvent), and a variety of alkyl (methanesulfonic acid, ethanesulfonic acid, camphorsulfonic acid) and aryl sulfonic acids were compatible. There are very few examples of enantioselective vicinal disulfonoxylation reactions in the literature, and the substrate scope of these reactions is limited to just styrene! [8–11] We hypothesized that addressing this clear literature gap will allow for the development of general, enantioselective vicinal disulfonoxylation reactions whose products will be useful to a range of chemical communities (Scheme 1).

Inspired by the work of Wirth and Fujita on the enantioselective ditosyloxylation of styrene [8,11], we began optimization of our proposed enantioselective vicinal olefin disulfonoxylation reaction by screening enantiopure hypervalent iodine reagents (Table 1) [12–15]. We found that when 1-octene was stirred with enantiopure I(III) oxidant **V** [16] and *p*-toluenesulfonic acid (*p*-TsOH) in CH_2_Cl_2_ at room temperature, desired ditosylate product formed in a 74% yield and with an enantioselectivity of 70%. We were pleased to see that when the solvent was switched from CH_2_Cl_2_ to 1,2-dichlorobenzene, the yield improved to an impressive 86% and the enantioselectivity improved to a respectable 77%. Maintaining the reaction temperature at 0 ^◦^C slightly dropped the product yield but improved the enantioselectivity to 88%. Attempts to make this transformation catalytic with respect to enantiopure aryl iodide were unsuccessful even when using several equivalents of a cheaper stoichiometric oxidant like *m*CPBA, but we note that the reduced aryl iodide could often be recovered and purified from the reaction mixtures.

As we had exclusively conducted our optimization experiments with *p*-TsOH, we were curious about the compatibility of other sulfonic acids (Scheme 2). We were pleased to find that a diverse array of sulfonic acids reacted smoothly with oct-1-ene to give scalemic vicinal disulfonates in synthetically useful yields and with good to excellent enantioselectivities. Methoxy groups, nitro groups, and aryl halides were all tolerated (Scheme 2, **Products 2B, 2C, 2D, 2F, 2G**). Even “bulky” partners such as 1-naphthalene and 2-naphthalenesulfonic acids (Scheme 2, **Products 2I and 2J**) were amenable. Not all sulfonic acids were compatible, however, and a selection of these are also provided (Scheme 2, **Poor Performers**).

A diverse array of terminal olefins was compatible with our optimized reaction conditions (Scheme 3). Depending on the substrate, enantioselectivities up to 93% could be achieved. In general, sterically unencumbered terminal olefins, i.e. those not proximal to bulky groups such as phenyl rings, gave products with an optimal combination of high yields and enantioselectivities (Scheme 3, **Products 3A, 3J, 3M, 3S, etc.**). In contrast, allylbenzene substrates were low yielding, and the scalemic products were barely enantioenriched (Scheme 3, **Poor Performers**). Our optimized protocol was tolerant of several interesting functional groups, including nitriles, tosylates, esters, sulfamates, and phthalimides (Scheme 3, **Products 3D and 3 M – 3S**). Terminal olefin substrates were required for productive reactivity, as no disulfonates were observed with *trans*-stilbene or with *trans*-3-hexenedinitrile (Scheme 3, **Poor Performers**). The absolute stereochemistry of these products was assigned by comparison to authentic standards synthesized from commercially available enantiopure alcohols (see Supporting Information, Structural Reasoning Section).

A plausible reaction mechanism for this transformation is depicted in Scheme 4. Enantiodifferentiation likely occurs during attack of the olefin substrate by the chiral I(III) oxidant [17]. A reductive elimination furnishes the product and the reduced, enantiopure aryl iodide.

The reaction scale could be increased from 0.2 mmol to 1 mmol without loss of yield or enantioselectivity (Scheme 5A). Ditosylate **2A** was a versatile chiron (Scheme 5B). Both tosylates could be displaced using excess sodium azide. The azides could be reduced to amines upon hydrogenation with palladium on carbon. The resulting amines could then be converted into di-carbamates or di-ureas. We were particularly pleased with the latter result, as scalemic ureas are well known as hydrogen-bonding catalysts for a variety of interesting, enantioselective transformations [18,19].

In summary, we have invented technology for enantioselective, vicinal disulfonoxylation of terminal olefins. Our protocol is operationally simple and involves stirring olefin substrate with 1.2 equivalents of an enantiopure I(III) reagent and two equivalents of a sulfonic acid in 1,2-dichlorobenzene at 0 °C for 19 h. No special precautions are required to exclude air or ambient moisture. Many interesting functional groups survive the reaction conditions, and the products are useful chirons. Given the central importance of enantioselective dihydroxylation, we expect its cousin to be equally embraced in time.

## Supplementary Material

Supporting Information

## Figures and Tables

**Scheme 1.**
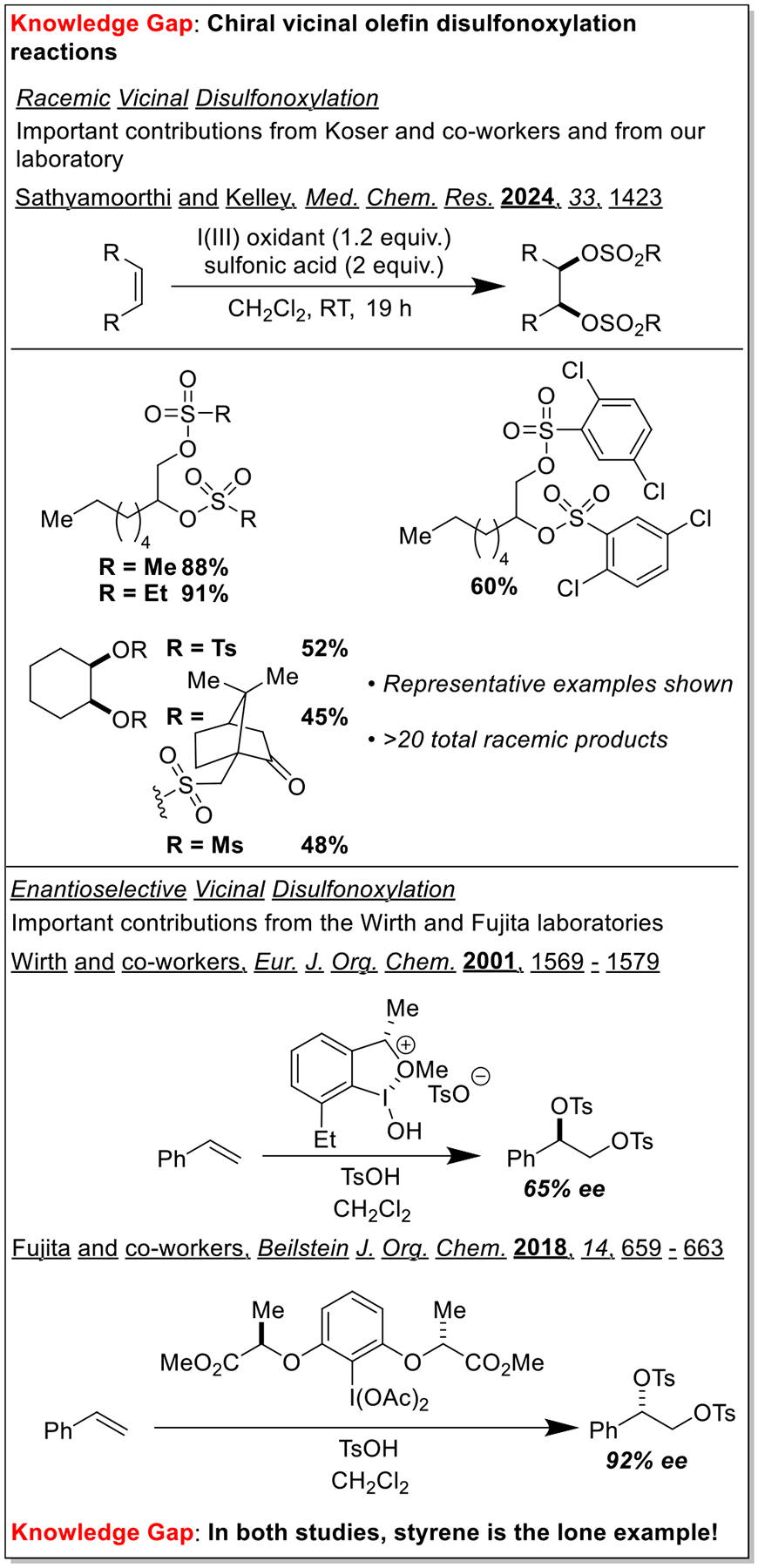
There are few examples of racemic olefin disulfonoxylation reactions and even fewer examples of the enantioselective versions.

**Scheme 2.**
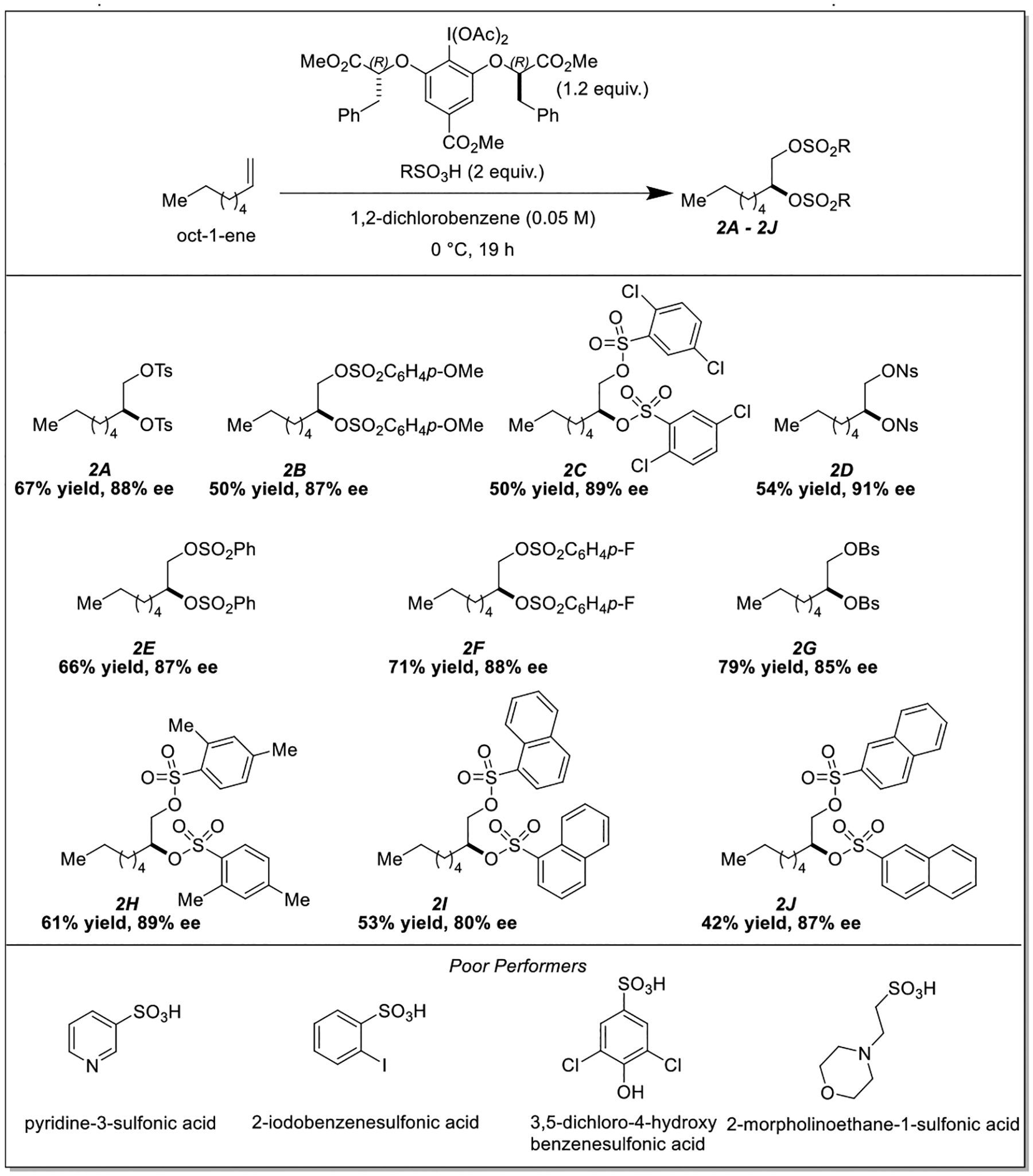
Examining the vicinal difunctionalization of oct-1-ene with various sulfonic acid partners.

**Scheme 3.**
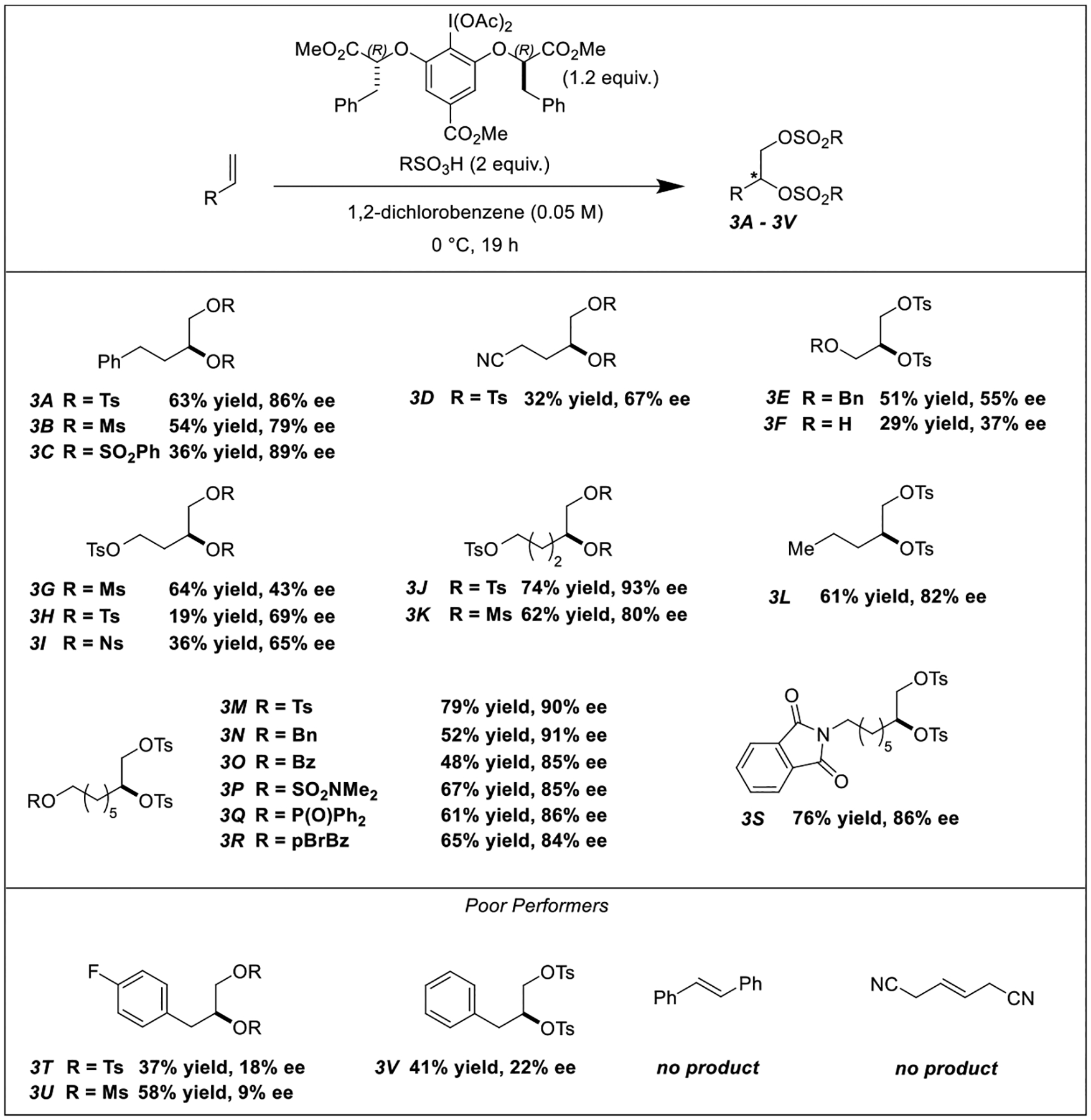
Survey of various alkenes with diverse coupling partners.

**Scheme 4.**
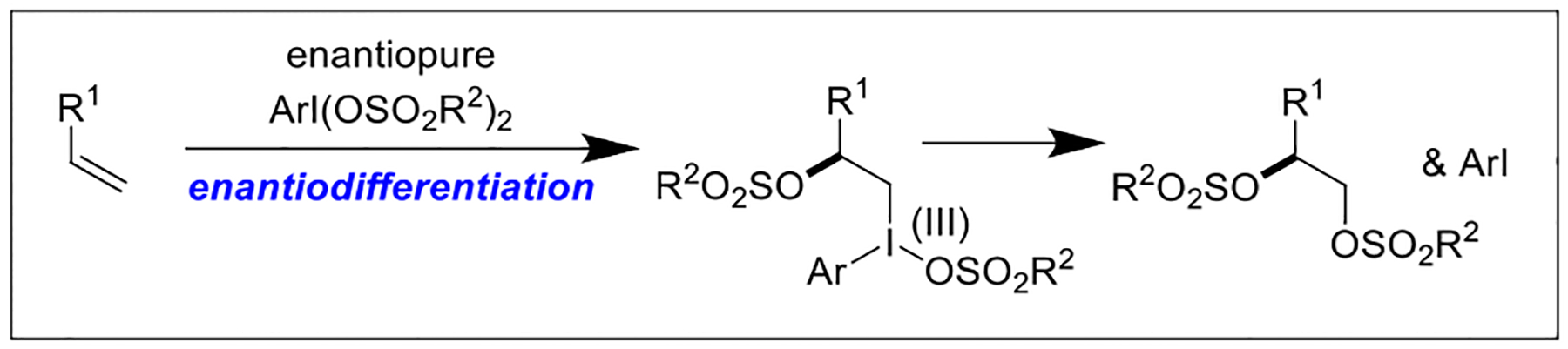
Proposed reaction mechanism for the enantioselective vicinal olefin disulfonoxylation.

**Scheme 5.**
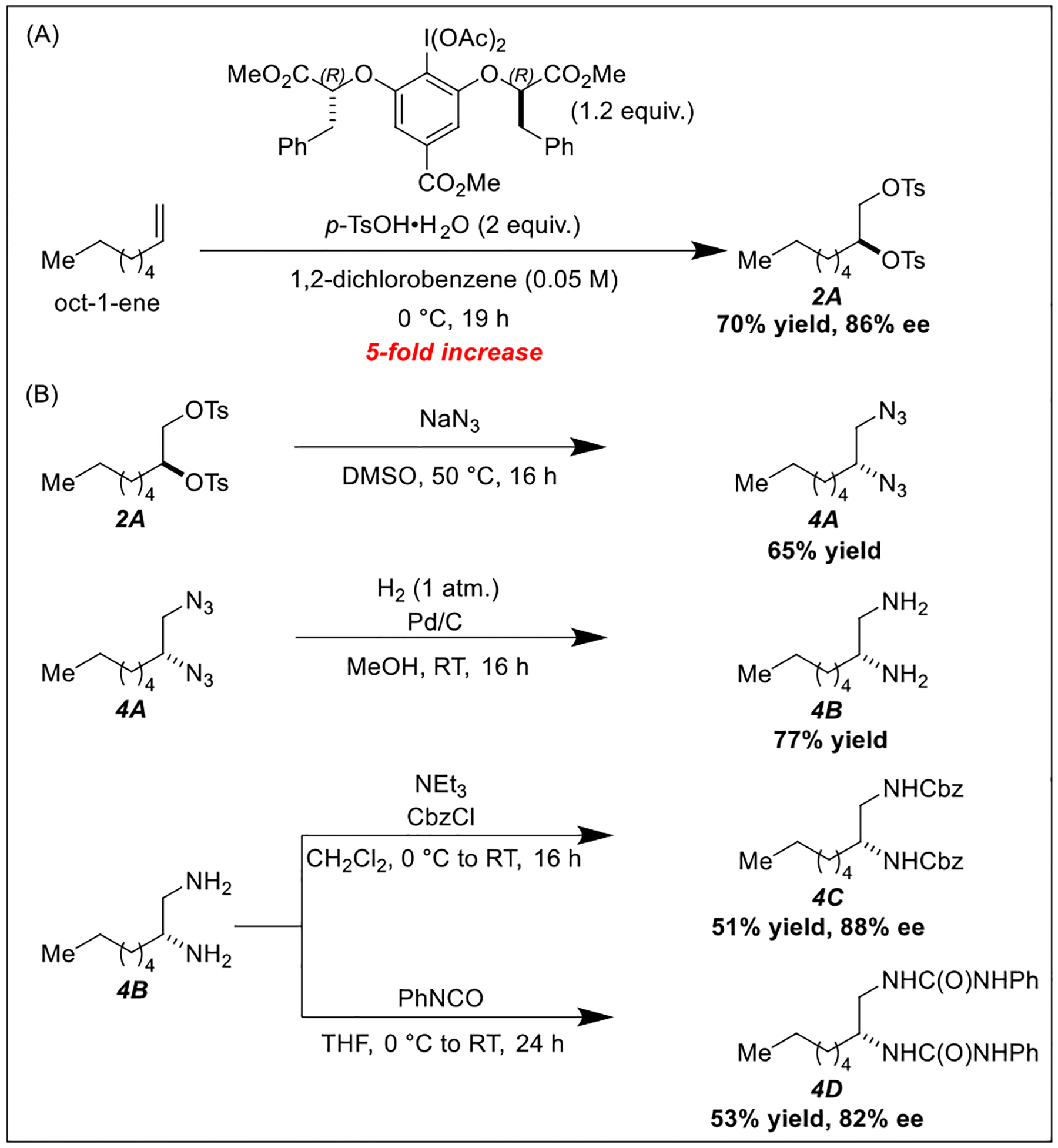
(A) Scale-up reaction (B) Applications.

**Table 1:** Optimization studies.

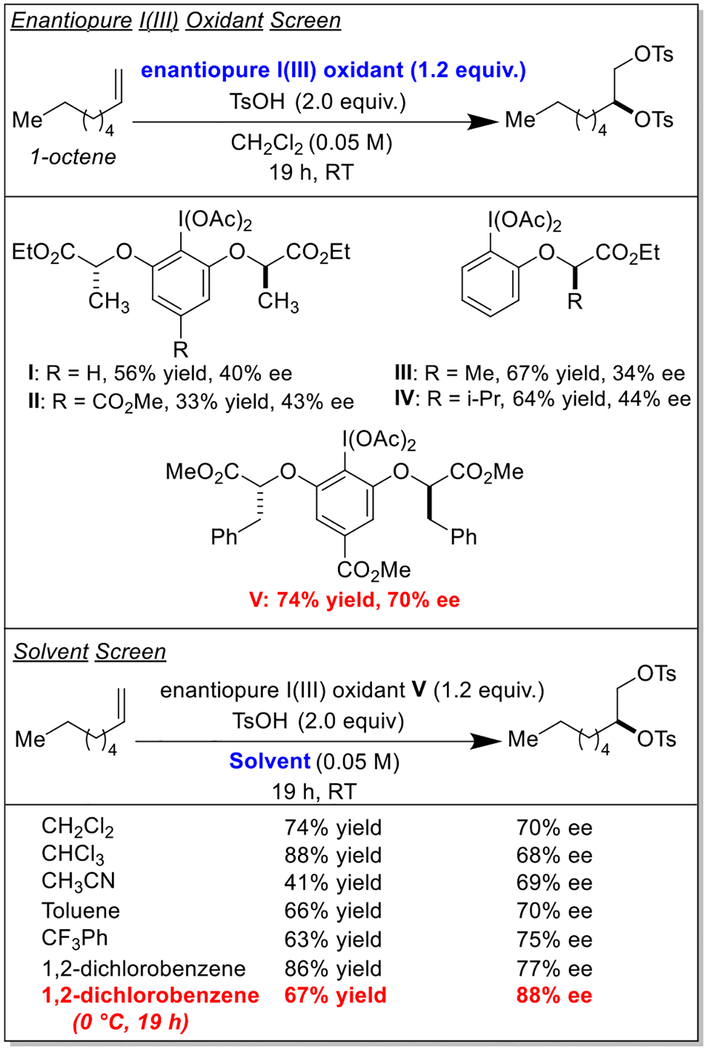

## Data Availability

Data will be made available on request.

## Appendix A. Supplementary data

Supplementary data to this article can be found online at https://doi.org/10.1016/j.tet.2026.135286.
