# Supplementary material for: Enantioselective vicinal olefin disulfonoxylation
Source: Tetrahedron. Author manuscript; Available in PMC 2026 Apr 24. (PMC13099161; doi:10.1016/j.tet.2026.135286)

Supporting Information

Enantioselective Vicinal Olefin Disulfonoxylation

Abhijit Manna,[a] Raju Silver,[a] Mintu Munda,[a] and Shyam Sathyamoorthi*,[a]

[a] Department of Medicinal Chemistry, University of Kansas, Lawrence, KS 66047, USA

corresponding author email: ssathyam@ku.edu

Table of Contents

I. General Considerations
II. Procedures, Characterization Data, and NMR Spectra for Enantiopure Hypervalent Iodine Reagent Preparation (Reagent V in Table 1)
III. General Procedure for the Enantioselective Vicinal Olefin Disulfonoxylation
IV. Characterization Data, HPLC Traces, and NMR Spectra for Substrates and Products of Enantioselective Vicinal Olefin Disulfonoxylation
V. Procedures, Characterization Data, HPLC Traces, and NMR Spectra for Scale-Up and Application Examples
VI. Structural Reasoning

I. General Considerations

All reagents were obtained commercially unless otherwise noted. Solvents were purified by passage under 10 psi N_2 through activated alumina columns. Infrared (IR) spectra were recorded on a Thermo Scientific™ Nicolet™ iS™5 FT-IR Spectrometer; data are reported in frequency of absorption (cm^{-1}). ^1H NMR spectra were recorded at 400, 500, or 600 MHz. Data are recorded as: chemical shift in ppm referenced internally using residual solvent peaks, multiplicity (s = singlet, br s = broad singlet, d = doublet, t = triplet, q = quartet, m = multiplet or overlap of nonequivalent resonances, qdd = quartet of doublet of doublets, tdt = triplet of doublet of triplets, dtq = doublet of triplet of quartets, qd = quartet of doublets, tdq = triplet of doublet of quartets), coupling constant (Hz), integration. ^{13}C NMR spectra were recorded at 101 or 126 MHz. Exact mass spectra were recorded using an electrospray ion source (ESI) either in positive mode or negative mode and with a time-of-flight (TOF) analyzer on a Waters LCT PremierTM mass spectrometer and are given in m/z. Thin Layer Chromatography (TLC) was performed on pre-coated glass plates (Merck) and visualized either with a UV lamp (254 nm) or by dipping into a solution of $KMnO_4$–K_2CO_3 in water followed by heating. Flash chromatography was performed on silica gel (230-400 mesh) or Florisil (60-100 mesh). "Room temperature" refers to an ambient temperature of 23 – 25 °C.

II. Procedures, Characterization Data, and NMR Spectra for Enantiopure Hypervalent Iodine Reagent Preparation (Reagent V in Table 1)

The synthesis of the enantiopure hypervalent iodine reagent involves two steps: the first step is a Mitsunobu reaction, and the second step is an oxidation reaction to generate the active hypervalent iodine species.

First Step:

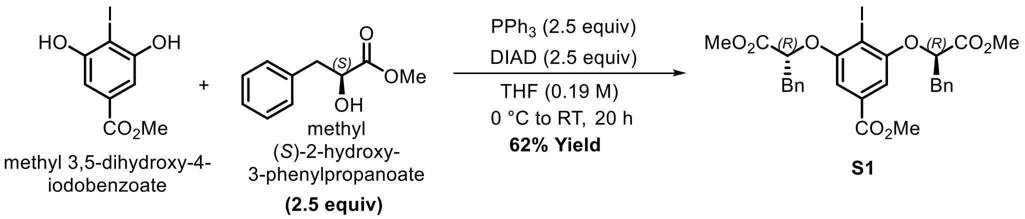

An oven-dried 250 mL round-bottom flask equipped with a magnetic stir bar was charged with methyl 3,5-dihydroxy-4-iodobenzoate (5.00 g, 17.00 mmol, 1.00 equiv.), methyl (*S*)-2-hydroxy-3-phenylpropanoate (7.66 g, 42.51 mmol, 2.5 equiv.), triphenylphosphine (11.15 g, 42.51 mmol, 2.5 equiv.), and HPLC-grade tetrahydrofuran (89 mL, Reaction concentration = ~0.2 M). With magnetic stirring, the solution was cooled to 0 °C using an ice-water bath. Then, diisopropyl azodicarboxylate (DIAD) (8.37 mL, 8.62 g, 42.63 mmol, 2.5 equiv.) was added dropwise over a period of 25 minutes *via* syringe. The reaction mixture was then allowed to warm to room temperature and stirred for 20 hours. The reaction mixture was then concentrated under reduced pressure. The crude residue was subjected to flash column chromatography on silica gel using a gradient of 10–60% ethyl acetate in hexane, which afforded an inseparable mixture of the desired product and a DIAD-related impurity. The mixture was then washed thoroughly with methanol (300 mL) to remove the impurities, yielding the pure product **S1** as a white solid (6.51 g, 10.53 mmol, 62% yield). (**Note**: Methanol washing was continued until all impurities were completely removed)

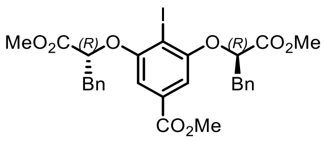

dimethyl 2,2'-((2-iodo-5-(methoxycarbonyl)-1,3-phenylene)bis(oxy))(2*R*,2'*R*)-bis(3-phenylpropanoate)

Compound S1:

^{1}H NMR (400 MHz, $CDCl_3$) δ 7.48 – 7.42 (m, 4H), 7.37 – 7.31 (m, 4H), 7.30 – 7.27 (m, 2H), 6.90 (s, 2H), 4.96 (dd, *J* = 7.6, 4.8 Hz, 2H), 3.87 (s, 3H), 3.72 (s, 6H), 3.43 – 3.31 (m, 4H).

$^{13}C\{^1H\}$ NMR (101 MHz, $CDCl_3$) δ 170.6, 166.1, 158.1, 136.0, 131.7, 130.0, 128.6, 127.2, 106.5, 86.3, 78.8, 52.6, 52.5, 39.1.

IR ν 2950, 2359, 1733, 1578, 1418, 1285, 1113, 1010, 757, 700, 660 cm^{-1}.

HRMS (ESI) m/z = $[M + Na]^+$ Calcd $C_{28}H_{27}IO_8Na^+$ 641.0648. Found Mass 641.0641 (1.1 ppm error).

$[\alpha]_D^{23}$ = +58.76 (c = 1.71g/100 mL, $CHCl_3$).

The data is consistent with that previously reported in *Science*, **2016**, *353*, 51-54.

Compound S1 ($CDCl_3$, ^1H NMR: 400 MHz, $^{13}C\{^1H\}$ NMR: 101 MHz)

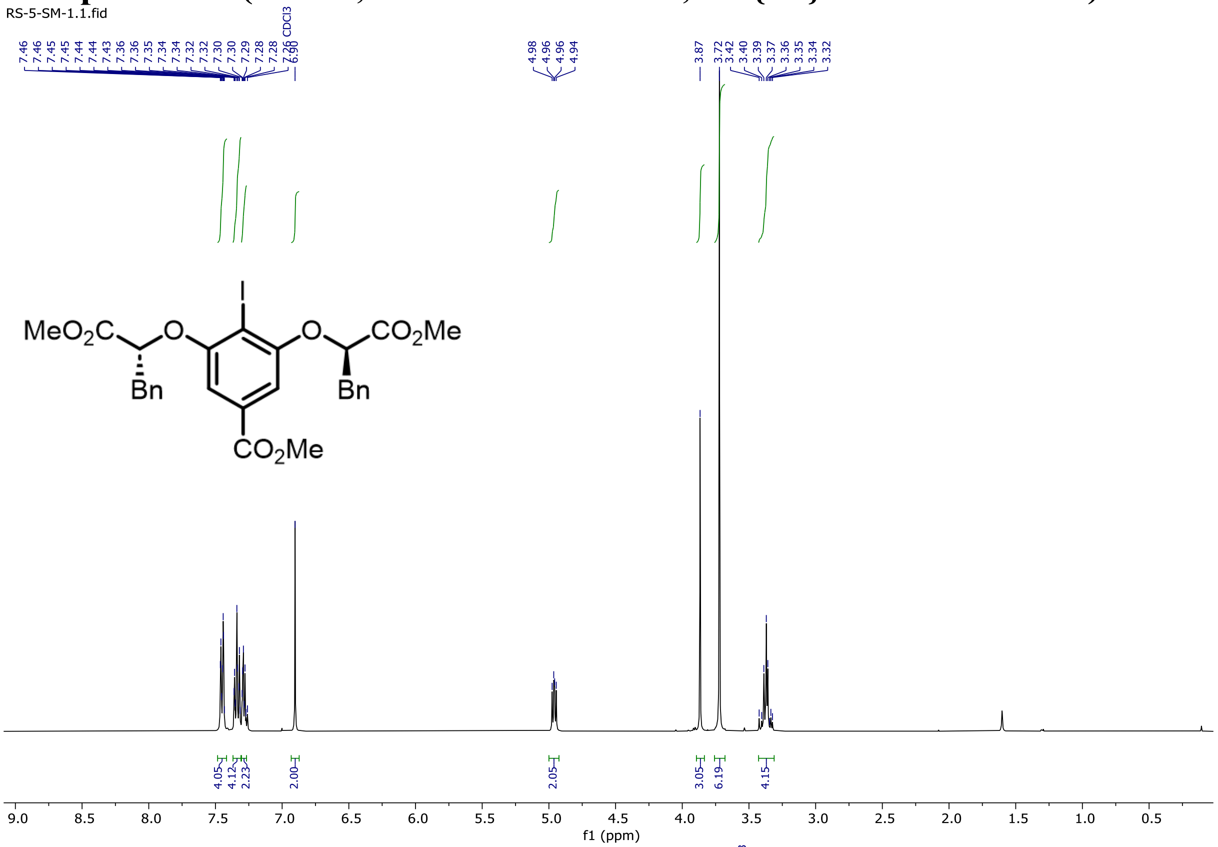

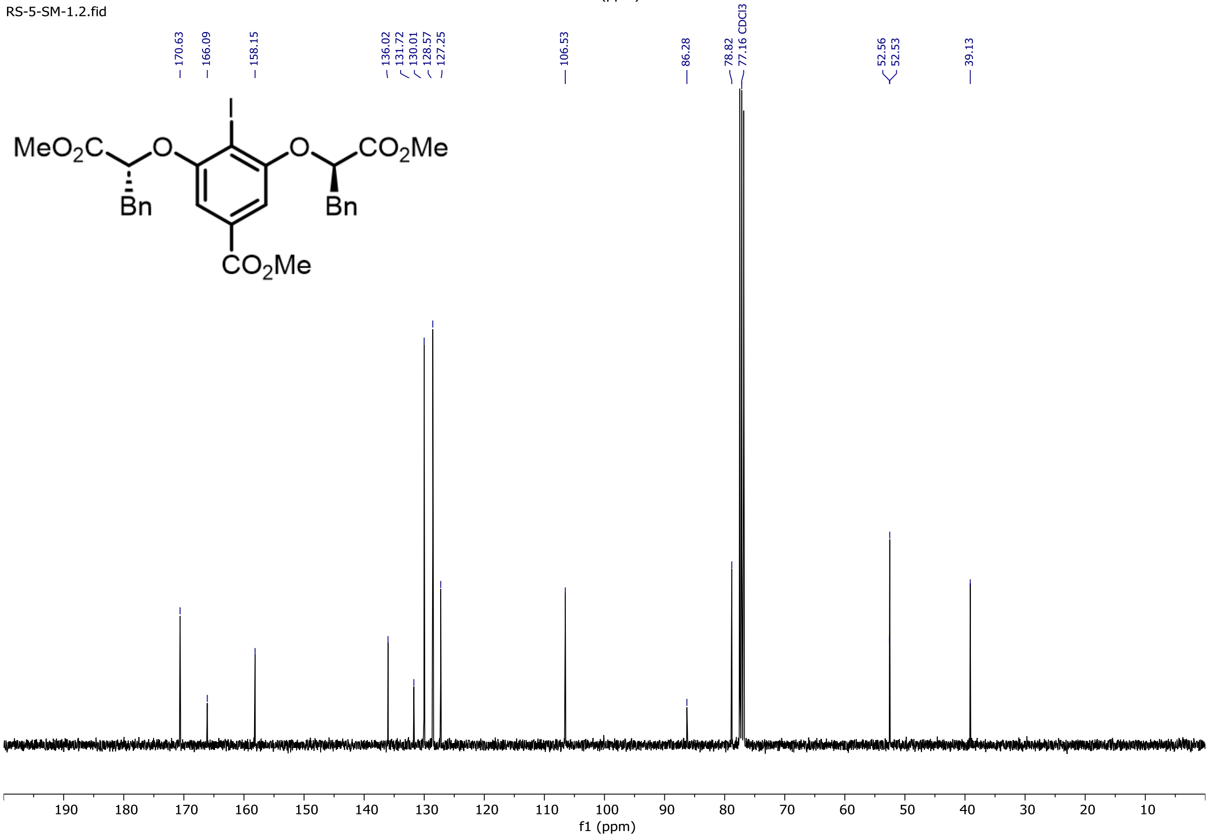

Second Step:

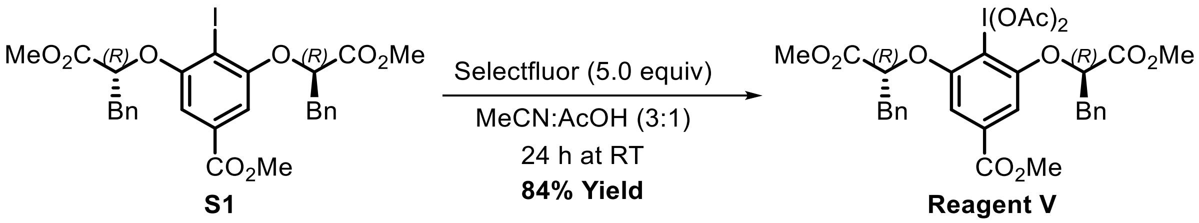

An oven-dried 500 mL round-bottom flask equipped with a magnetic stir bar was charged with **S1** (5.00 g, 8.08 mmol, 1.00 equiv.) in HPLC-grade acetonitrile (240 mL). Selectfluor (14.32 g, 40.42 mmol, 5.00 equiv.) was added in a single portion, and the reaction mixture was stirred until a homogeneous solution was obtained. Acetic acid (80 mL) was then added dropwise over 20 minutes, and the reaction mixture was stirred at room temperature for 24 hours. The mixture was then concentrated under reduced pressure, and water (200 mL) was added to the residue. After transferring to a separatory funnel, the resulting mixture was extracted with dichloromethane (3 × 100 mL). The combined organic layers were washed with water (100 mL), dried over anhydrous $MgSO_4$, filtered, and concentrated under reduced pressure. The crude residue was azeotroped with heptane to remove residual acetic acid, affording the enantiopure hypervalent iodine **Reagent V** as a yellow solid (5.00 g, 6.79 mmol, 84% yield).

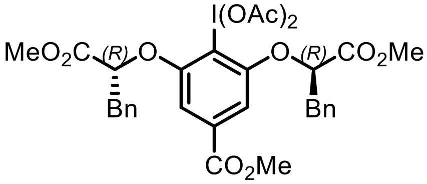

dimethyl 2,2'-((2-(diacetoxy-λ^3-iodaneyl)-5-(methoxycarbonyl)-1,3-phenylene)bis(oxy))(2*R*,2'*R*)-bis(3-phenylpropanoate)

Reagent V:

^{1}H NMR (400 MHz, $CDCl_3$) δ 7.34 – 7.26 (m, 8H), 7.25 – 7.20 (m, 2H), 7.09 (s, 2H), 5.05 (dd, *J* = 7.3, 5.2 Hz, 2H), 3.87 (s, 3H), 3.69 (s, 6H), 3.36 – 3.26 (m, 4H), 1.88 (s, 6H).

$^{13}C\{^1H\}$ NMR (101 MHz, $CDCl_3$) δ 177.1, 169.9, 165.1, 156.3, 136.4, 135.5, 129.7, 128.7, 127.3, 111.3, 106.5, 78.9, 52.9, 52.6, 38.9, 20.5.

IR ν 3008, 2325, 1733, 1647, 1584, 1423, 1263, 1113, 1004 cm^{-1}.

HRMS (ESI) m/z = $[M + Na]^+$ Calcd $C_{32}H_{33}IO_{12}Na^+$ 759.0915. Found 759.0914 (0.1 ppm error).

$[\alpha]_D^{23}$ = -5.77 (c = 1.46 g/100 mL, $CHCl_3$).

Reagent V ($CDCl_3$, ^1H NMR: 400 MHz, $^{13}C\{^1H\}$ NMR: 101 MHz)

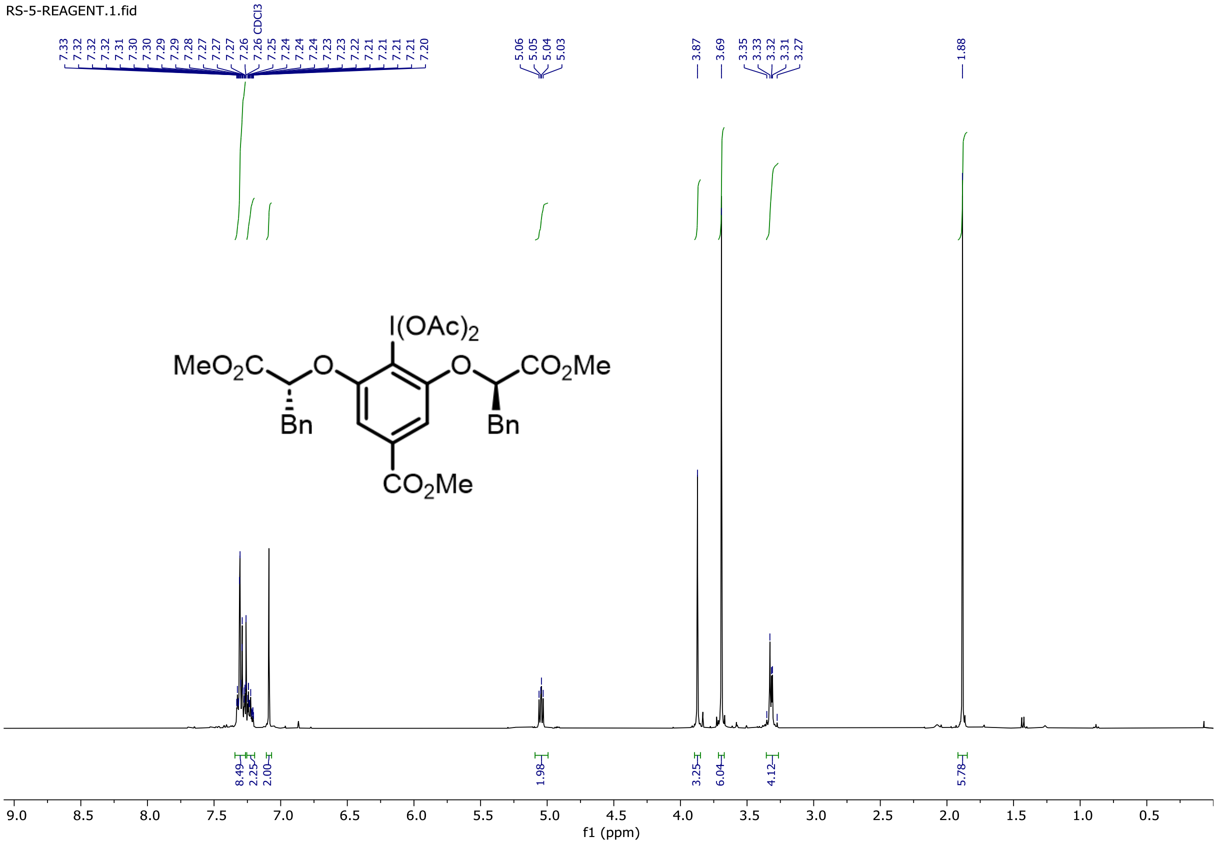

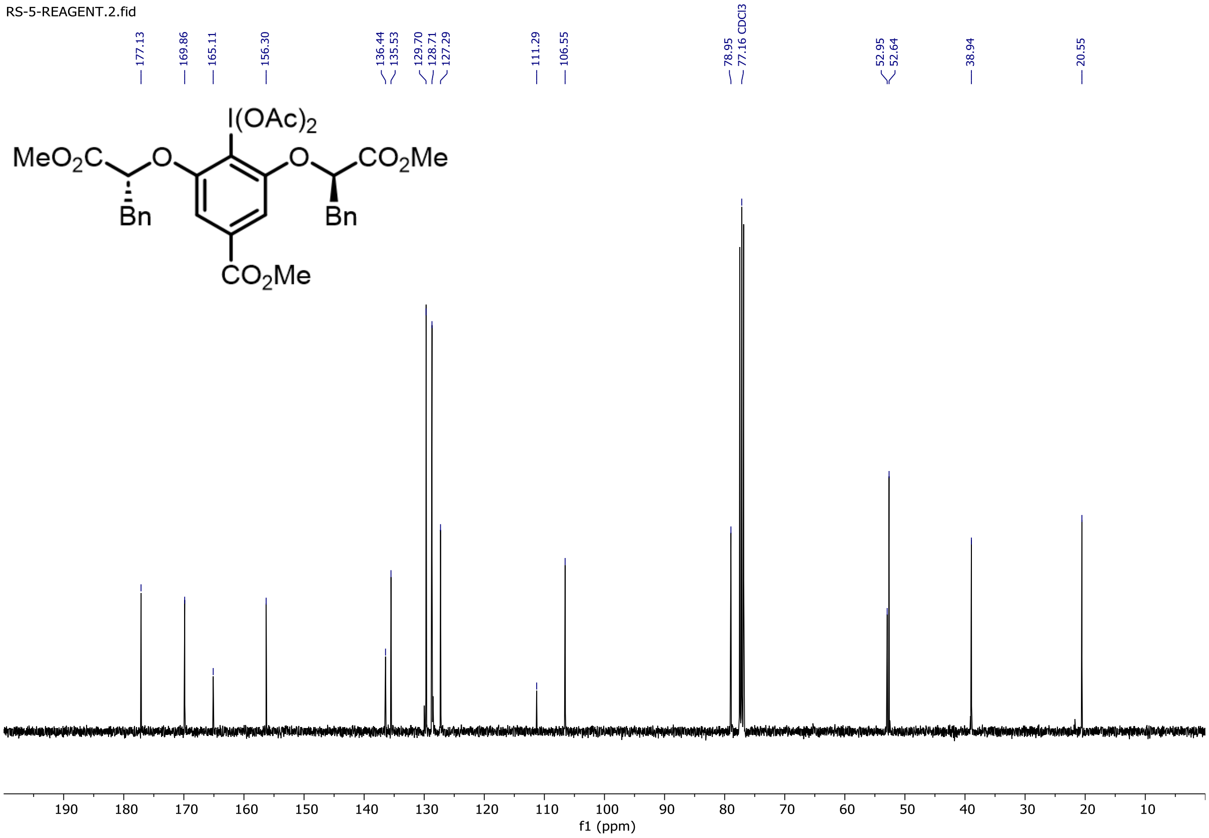

III. General Procedure for the Enantioselective Vicinal Olefin Disulfonoxylation

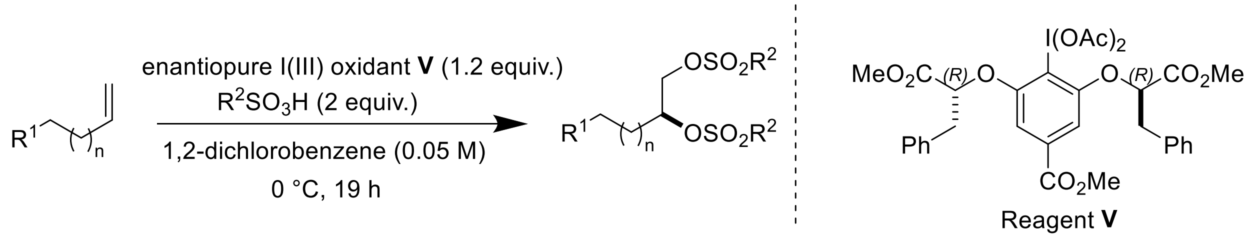

A 10 mL microwave vial was charged with a stir bar, alkene substrate (0.2 mmol, 1 equiv.), 1,2-dichlorobenzene (4 mL, reaction concentration = 0.05 M), enantiopure hypervalent I(III) oxidant **V** (0.177 g, 0.24 mmol, 1.2 equiv.), and sulfonic acid (0.4 mmol, 2 equiv.). The vial was sealed, and the reaction mixture was cooled to 0 °C using a Julabo immersion cooler. The reaction was stirred at 0 ℃ for 19 h. Following this time, the seal was broken, and the contents of the vial were transferred to a separatory funnel with CH_2Cl_2 (10 mL). The organic layer was washed with saturated aqueous $Na_2S_2O_3$ solution (10 mL) followed by saturated aqueous $NaHCO_3$ solution (10 mL). The organic layer was collected, dried with Na_2SO_4, filtered, and concentrated under reduced pressure. The resulting residue was purified by chromatography on silica gel. The reaction enantioselectivity was determined using chiral HPLC or SFC analysis. Specific reaction and purification conditions are given with each product.

IV. Characterization Data, HPLC Traces, and NMR Spectra for Substrates and Products of Enantioselective Vicinal Olefin Disulfonoxylation

Substrate-2 ($CDCl_3$, ^1H NMR: 400 MHz, $^{13}C\{^1H\}$ NMR: 101 MHz)

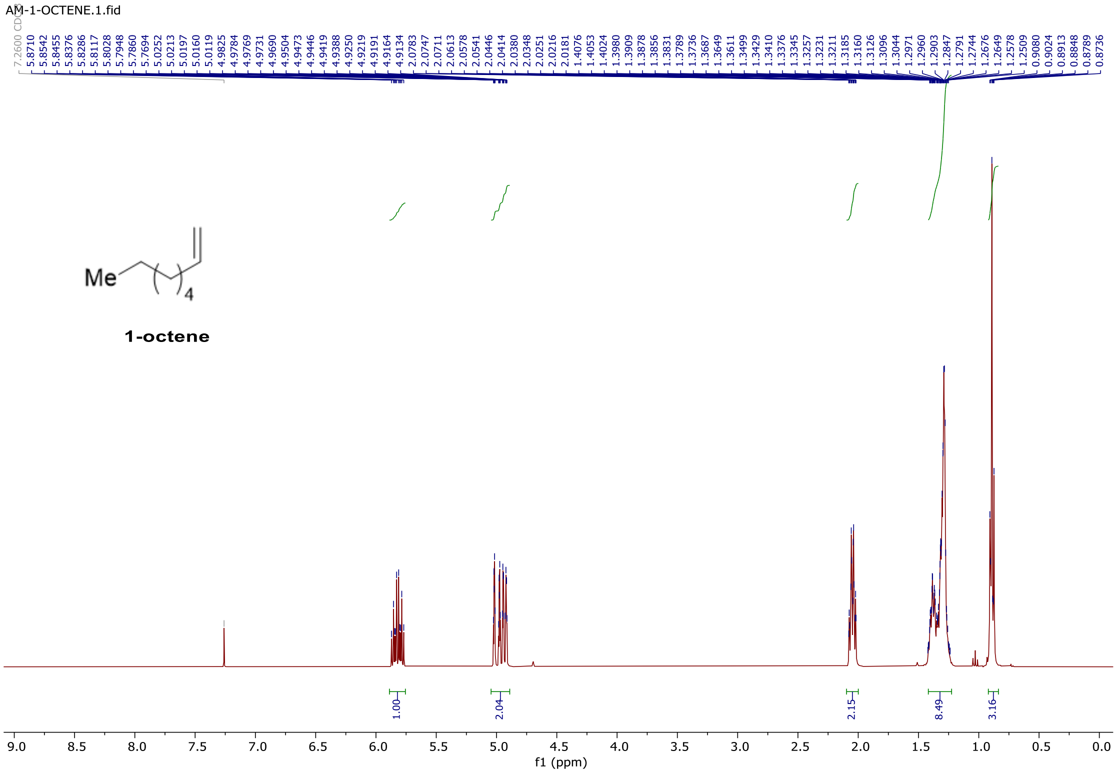

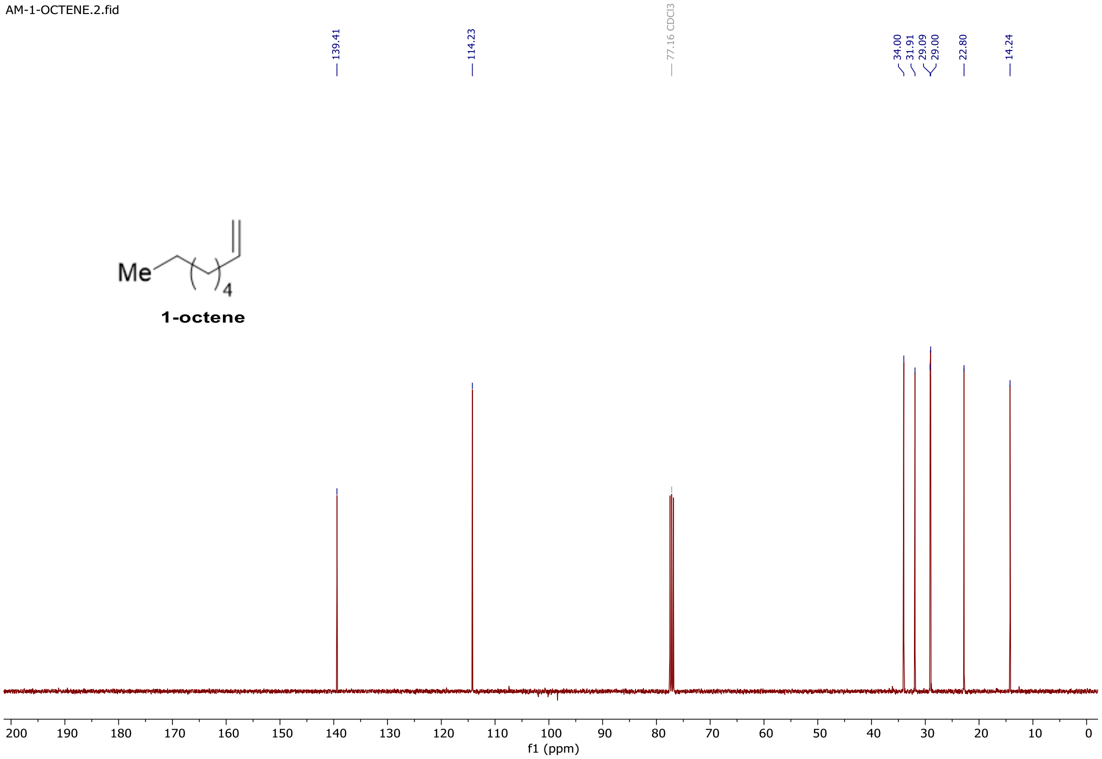

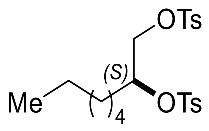

(*S*)-octane-1,2-diyl bis(4-methylbenzenesulfonate)

Compound 2A: Synthesized using the General Procedure on a 0.2 mmol scale; Purified using a gradient of 2% ethyl acetate in hexane to 8% ethyl acetate in hexane on silica gel; Predominant enantiomer depicted; (colorless oil, 61.0 mg, 0.134 mmol, 67% yield, 88% ee).

^{1}H NMR (400 MHz, $CDCl_3$) δ 7.77 – 7.61 (m, 4H), 7.39 – 7.28 (m, 4H), 4.57 (ddt, *J* = 7.1, 5.7, 4.6 Hz, 1H), 4.08 – 3.97 (m, 2H), 2.44 (s, 3H), 2.43 (s, 3H), 1.69 – 1.49 (m, 2H), 1.26 – 1.06 (m, 8H), 0.83 (t, *J* = 7.1 Hz, 3H).

$^{13}C\{^1H\}$ NMR (101 MHz, $CDCl_3$) δ 145.3, 145.1, 133.5, 132.4, 130.0, 129.9, 128.1, 128.0, 79.0, 69.5, 31.5, 31.1, 28.7, 24.4, 22.5, 21.8, 21.7, 14.1.

IR υ 3054, 2959, 2931, 2305, 1599, 1495, 1366, 1266, 1191, 1177, 1096, 997, 912, 815, 740, 554 cm^{-1}.

HRMS (ESI) m/z = $[M + Na]^+$ Calcd $C_{22}H_{30}O_6S_2Na^+$ 477.1382. Found 477.1371 (2.3 ppm error).

Specific Rotation: $[\alpha]_D^{25}$ = –18.6 (c = 2.98 g/100 mL, $CHCl_3$, 88% ee).

Stereochemistry assigned by analogy to an authentic sample of 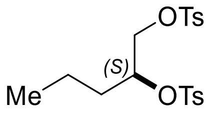

.

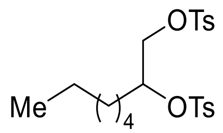

Racemic Sample: HPLC (IC-3, Isopropanol/hexanes = 10/90, flow rate = 1 mL/min, I = 254 nm), t_R = 79.5 min, 91.4 min.

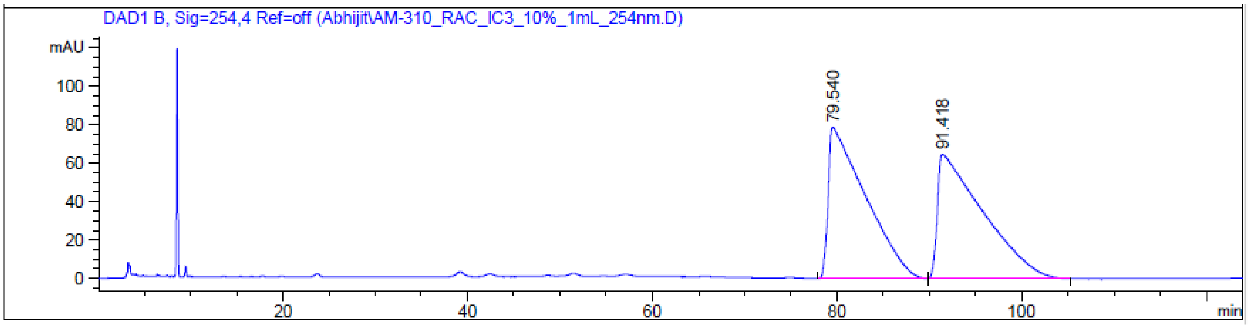

Peak #	RetTime [min]	Type	Width [min]	Area [mAU*s]	Height [mAU]	Area %
1	79.540	BB	3.4424	2.21416e4	78.79465	49.9636
2	91.418	BB	4.1422	2.21739e4	64.76858	50.0364

Scalemic Sample, +88% ee: HPLC (IC-3, Isopropanol/hexanes = 10/90, flow rate = 1 mL/min, I = 254 nm), t_R = 77.0 min, 97.0 min

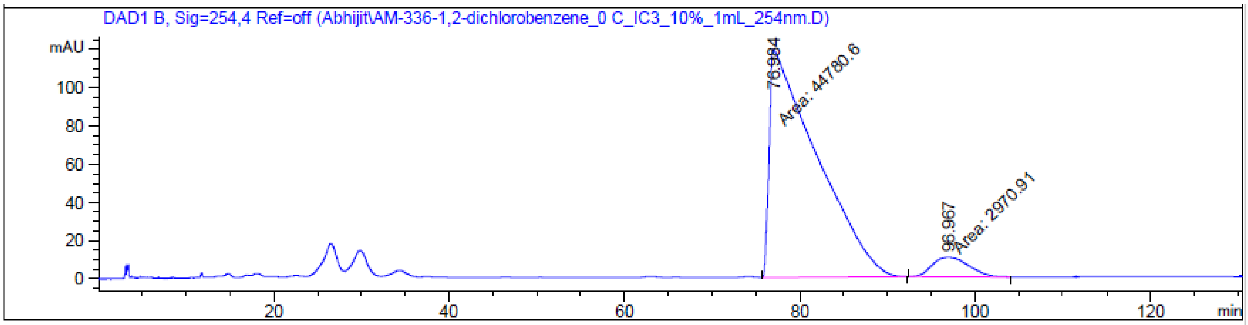

Peak #	RetTime [min]	Type	Width [min]	Area [mAU*s]	Height [mAU]	Area %
1	76.984	MM	6.2454	4.47806e4	119.50276	93.7784
2	96.967	MM	4.8392	2970.91357	10.23203	6.2216

Compound 2A ($CDCl_3$, ^1H NMR: 400 MHz, $^{13}C\{^1H\}$ NMR: 101 MHz)

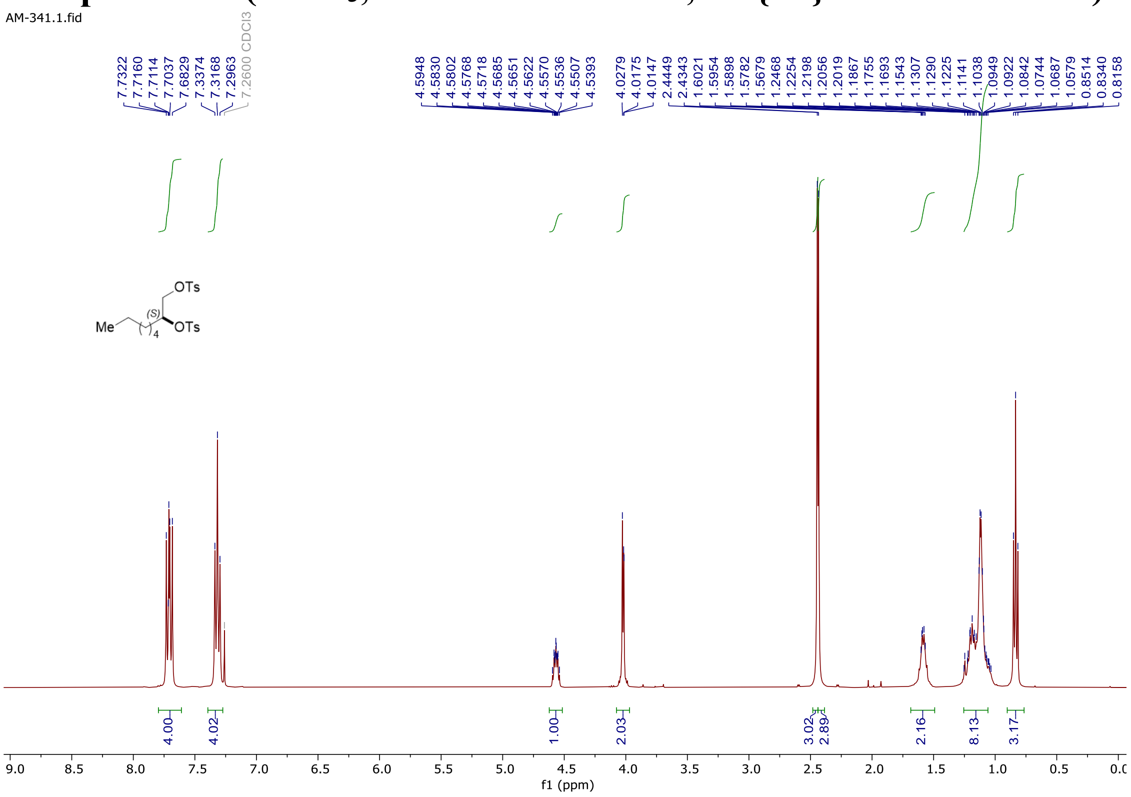

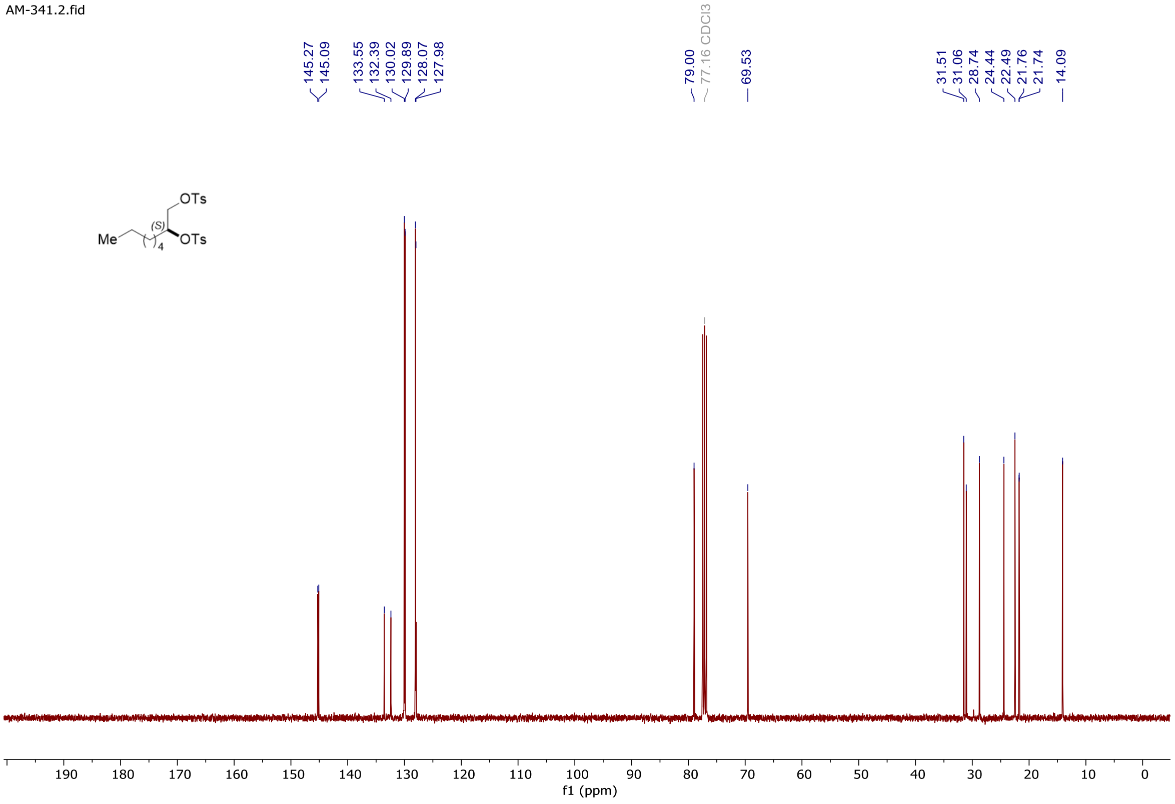

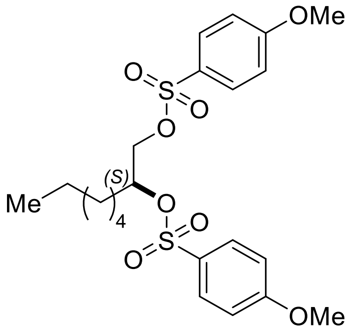

(*S*)-octane-1,2-diyl bis(4-methoxybenzenesulfonate)

Compound 2B: Synthesized using the General Procedure on a 0.2 mmol scale; Purified using a gradient of 2% ethyl acetate in hexane to 8% ethyl acetate in hexane on silica gel; Predominant enantiomer depicted; (colorless oil, 49.0 mg, 0.101 mmol, 50% yield, 87% ee).

^{1}H NMR (400 MHz, $CDCl_3$) δ 7.80 – 7.73 (m, 4H), 7.02 – 6.95 (m, 4H), 4.59 – 4.51 (m, 1H), 4.01 (d, *J* = 4.6 Hz, 2H), 3.89 (s, 3H), 3.88 (s, 3H), 1.64 – 1.54 (m, 2H), 1.25 – 1.08 (m, 8H), 0.84 (t, *J* = 7.0 Hz, 3H).

^{13}C{^{1}H} NMR (101 MHz, $CDCl_3$) δ 164.1, 164.0, 130.3, 130.2, 128.0, 126.7, 114.6, 114.5, 78.9, 69.4, 55.9, 55.8, 31.6, 31.2, 28.8, 24.5, 22.5, 14.1.

IR υ 2930, 2856, 1598, 1498, 1461, 1363, 1311, 1265, 1191, 1171, 1099, 1024, 912, 835, 760, 671 cm^{-1}.

HRMS (ESI) m/z = $[M + Na]^+$ Calcd $C_{22}H_{30}O_8S_2Na^+$ 509.1280. Found 509.1263 (3.3 ppm error).

Specific Rotation: $[\alpha]_D^{22}$ = –10.5 (c = 2.3 g/100 mL, $CHCl_3$, 87% ee).

Stereochemistry assigned by analogy to an authentic sample of 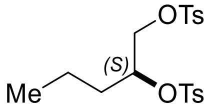

.

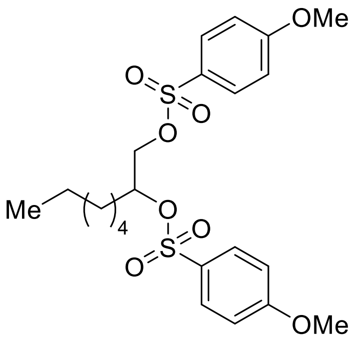

Racemic Sample: HPLC (IC-3, Isopropanol/hexanes = 20/80, flow rate = 1 mL/min, I = 254 nm), t_R = 75.3 min, 83.0 min.

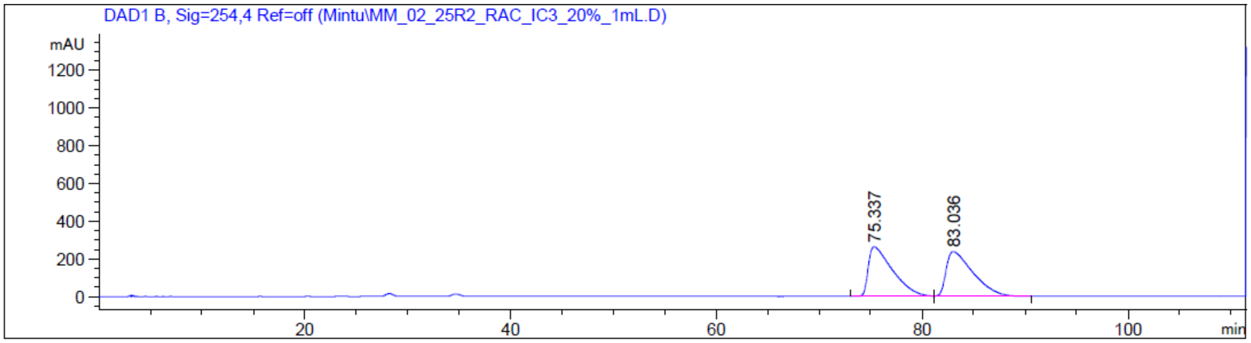

Peak #	RetTime [min]	Type	Width [min]	Area [mAU*s]	Height [mAU]	Area %
1	75.337	BB	2.1458	4.26147e4	262.52518	49.9767
2	83.036	BB	2.4579	4.26544e4	236.30493	50.0233

Scalemic Sample, +87% ee: HPLC (IC-3, Isopropanol/hexanes = 20/80, flow rate = 1 mL/min, I = 254 nm), t_R = 72.7 min, 85.3 min.

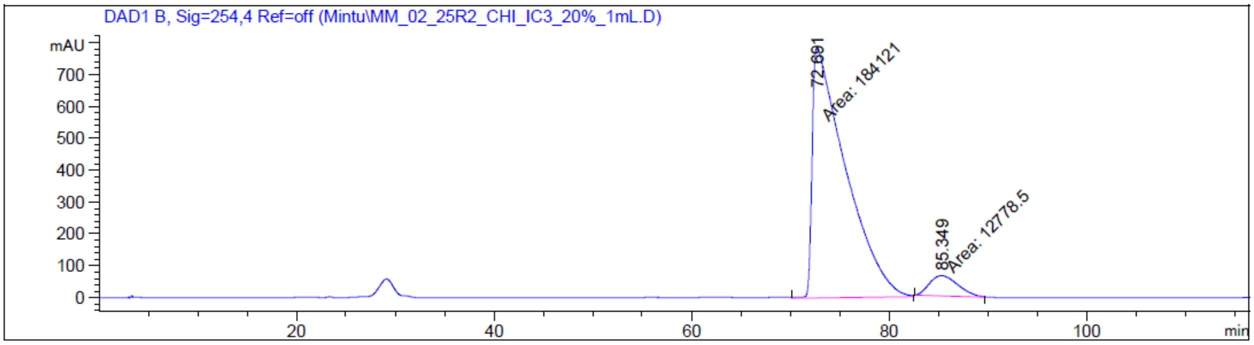

Peak #	RetTime [min]	Type	Width [min]	Area [mAU*s]	Height [mAU]	Area %
1	72.691	MM	3.9217	1.84121e5	782.48541	93.5101
2	85.349	MM	3.3586	1.27785e4	63.41121	6.4899

Compound 2B ($CDCl_3$, ^{1}H NMR: 400 MHz, $^{13}C\{^{1}H\}$ NMR: 101 MHz)

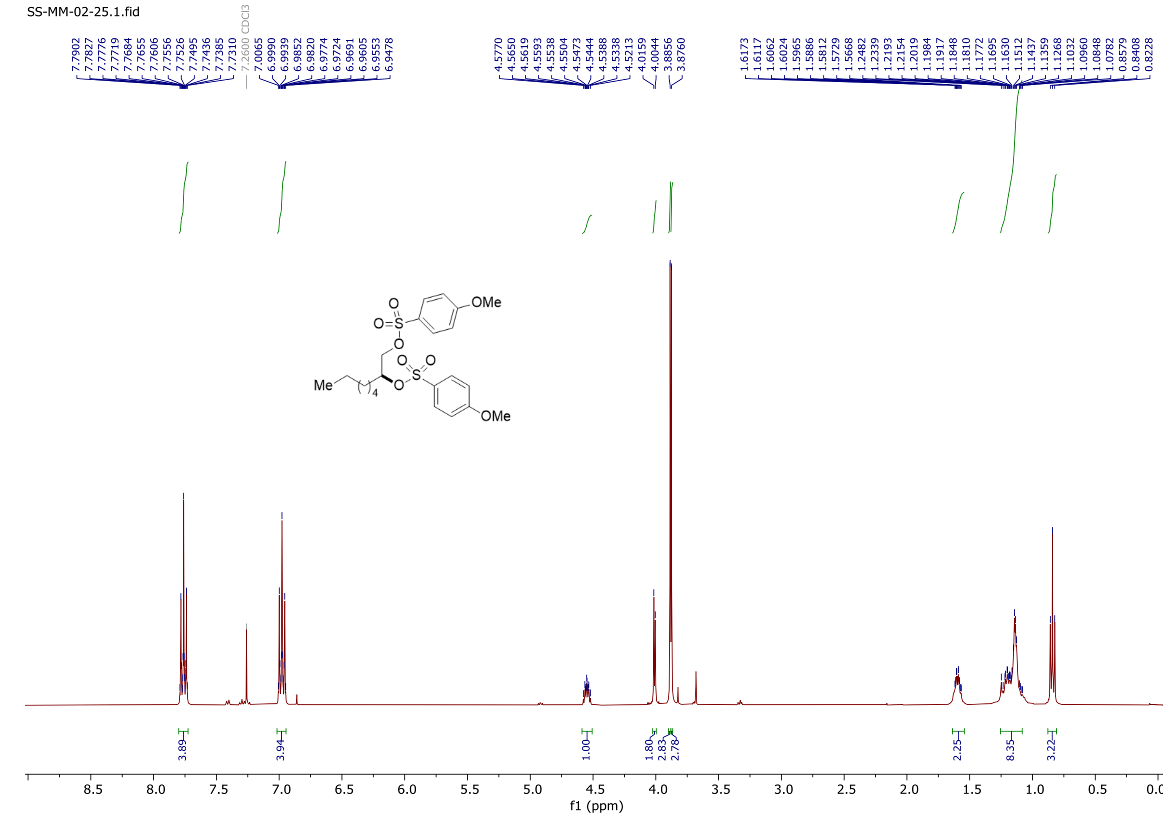

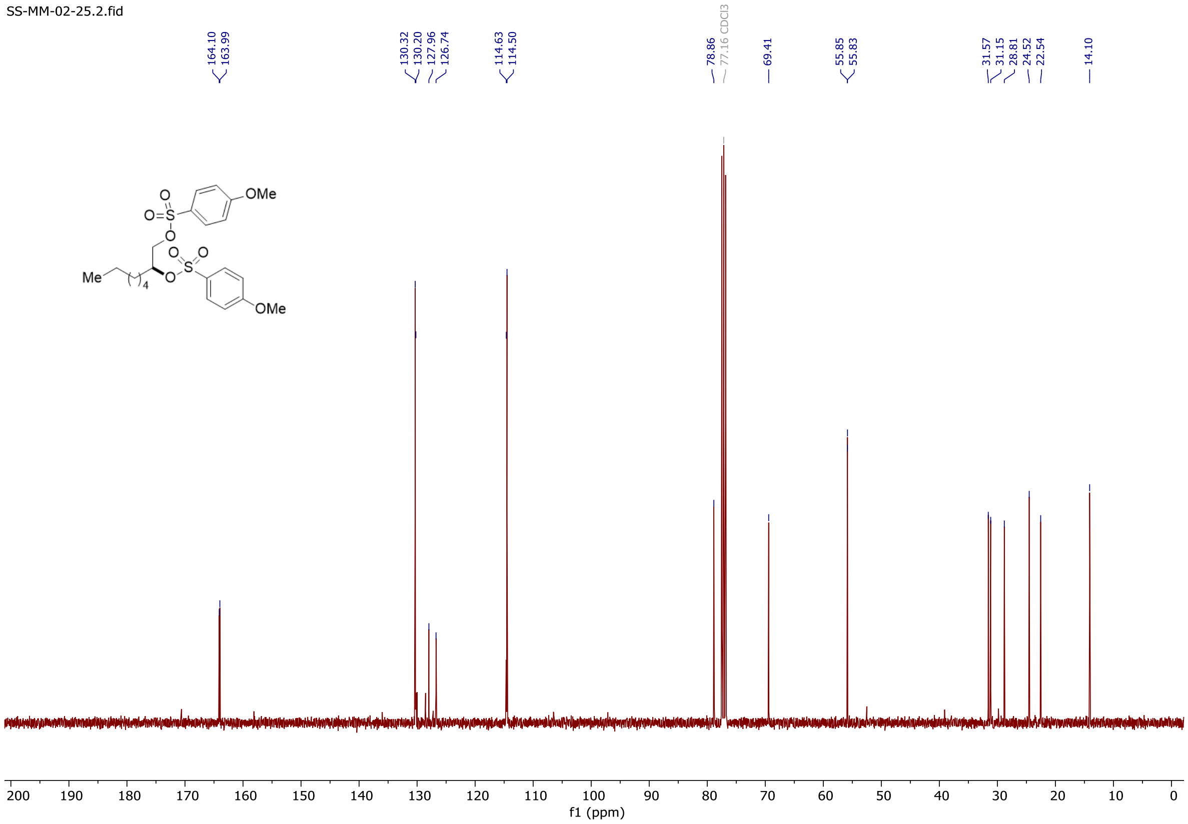

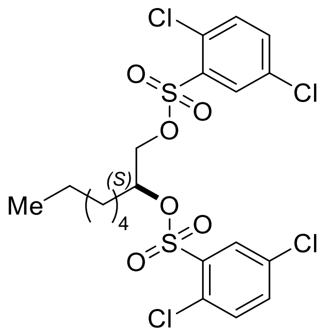

(*S*)-octane-1,2-diyl bis(2,5-dichlorobenzenesulfonate)

Compound 2C: Synthesized using the General Procedure on a 0.2 mmol scale; Purified using a gradient of 2% ethyl acetate in hexane to 8% ethyl acetate in hexane on silica gel; Predominant enantiomer depicted; (white solid, 56.0 mg, 0.1 mmol, 50% yield, 89% ee).

^{1}H NMR (400 MHz, $CDCl_3$) δ 7.99 – 7.93 (m, 2H), 7.53 (dd, *J* = 8.5, 2.4 Hz, 1H), 7.50 – 7.42 (m, 3H), 4.78 (dtd, *J* = 6.8, 5.3, 3.8 Hz, 1H), 4.30 – 4.21 (m, 2H), 1.87 – 1.69 (m, 2H), 1.34 – 1.17 (m, 8H), 0.86 (t, *J* = 6.9 Hz, 3H).

^{13}C{^{1}H} NMR (101 MHz, $CDCl_3$) δ 136.1, 135.02, 134.97, 134.7, 133.5, 133.38, 133.35, 133.32, 131.7, 131.5, 131.4, 131.3, 81.0, 70.5, 31.6, 31.2, 28.9, 24.5, 22.6, 14.1.

IR υ 2927, 1455, 1360, 1248, 1142, 1041, 912, 829, 588 cm^{-1}.

HRMS (ESI) m/z = $[M + Na]^+$ Calcd $C_{20}H_{22}Cl_4O_6S_2Na^+$ 584.9510. Found 584.9478 (5.5 ppm error).

Specific Rotation: $[\alpha]_D^{23}$ = + 4.14 (c = 2.8 g/100 mL, $CHCl_3$, 89% ee).

Stereochemistry assigned by analogy to an authentic sample of 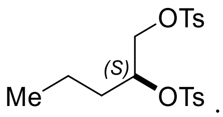

.

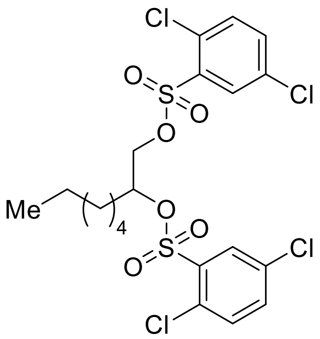

Racemic sample: HPLC (IA-3, isopropanol/n-hexane = 0.5/99.5, flow rate = 0.5 mL/min, λ = 220 nm), t_R = 93.7 min, 108.5 min.

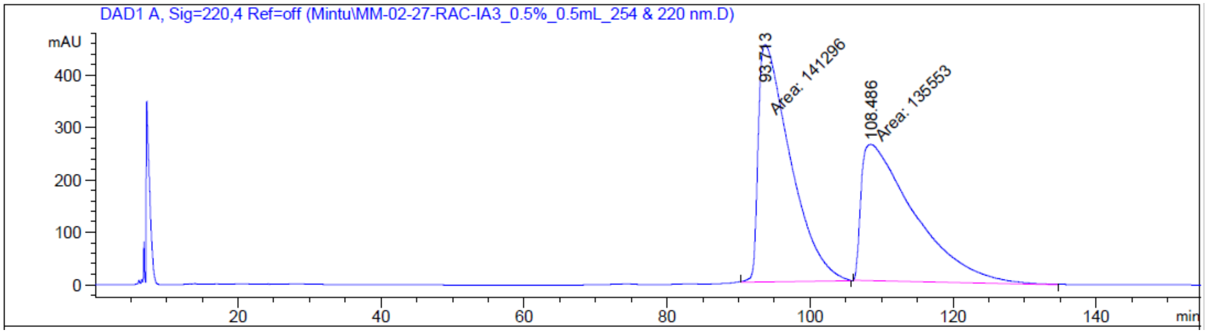

Peak #	RetTime [min]	Type	Width [min]	Area [mAU*s]	Height [mAU]	Area %
1	93.713	MM	5.2036	1.41296e5	452.56097	51.0371
2	108.486	MM	8.6681	1.35553e5	260.63681	48.9629

Scalemic sample, +89% ee: HPLC (IA-3, isopropanol/n-hexane = 0.5/99.5, flow rate = 0.5 mL/min, λ = 220 nm), t_R = 88.3 min, 112.4 min.

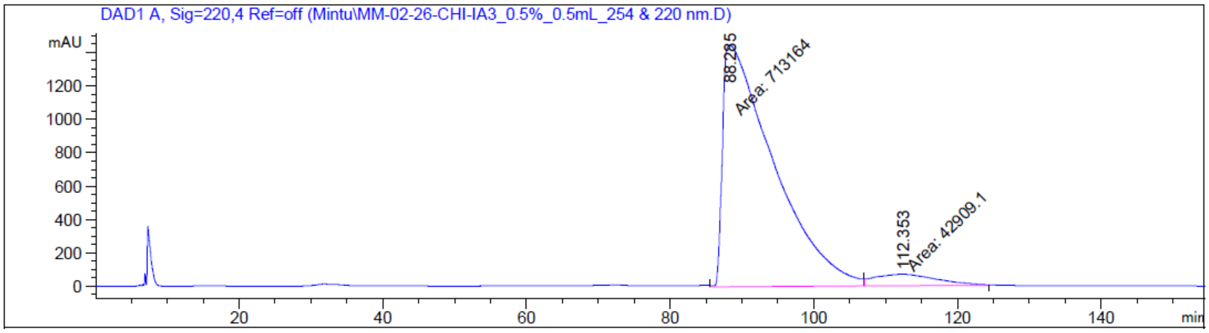

Peak #	RetTime [min]	Type	Width [min]	Area [mAU*s]	Height [mAU]	Area %
1	88.285	MF	8.2238	7.13164e5	1445.32263	94.3247
2	112.353	FM	10.3202	4.29091e4	69.29633	5.6753

Compound 2C (CDCl₃, ^{1}H NMR: 400 MHz, ^{13}C{^{1}H} NMR: 101 MHz)

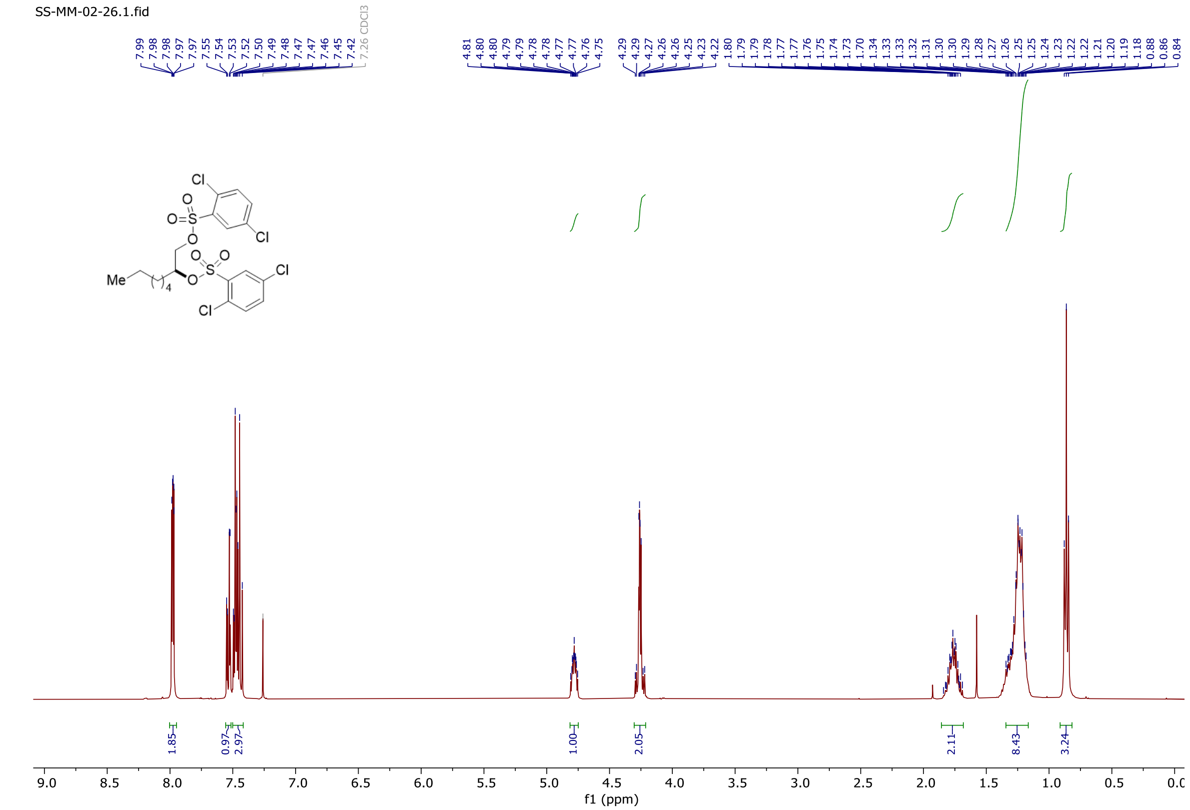

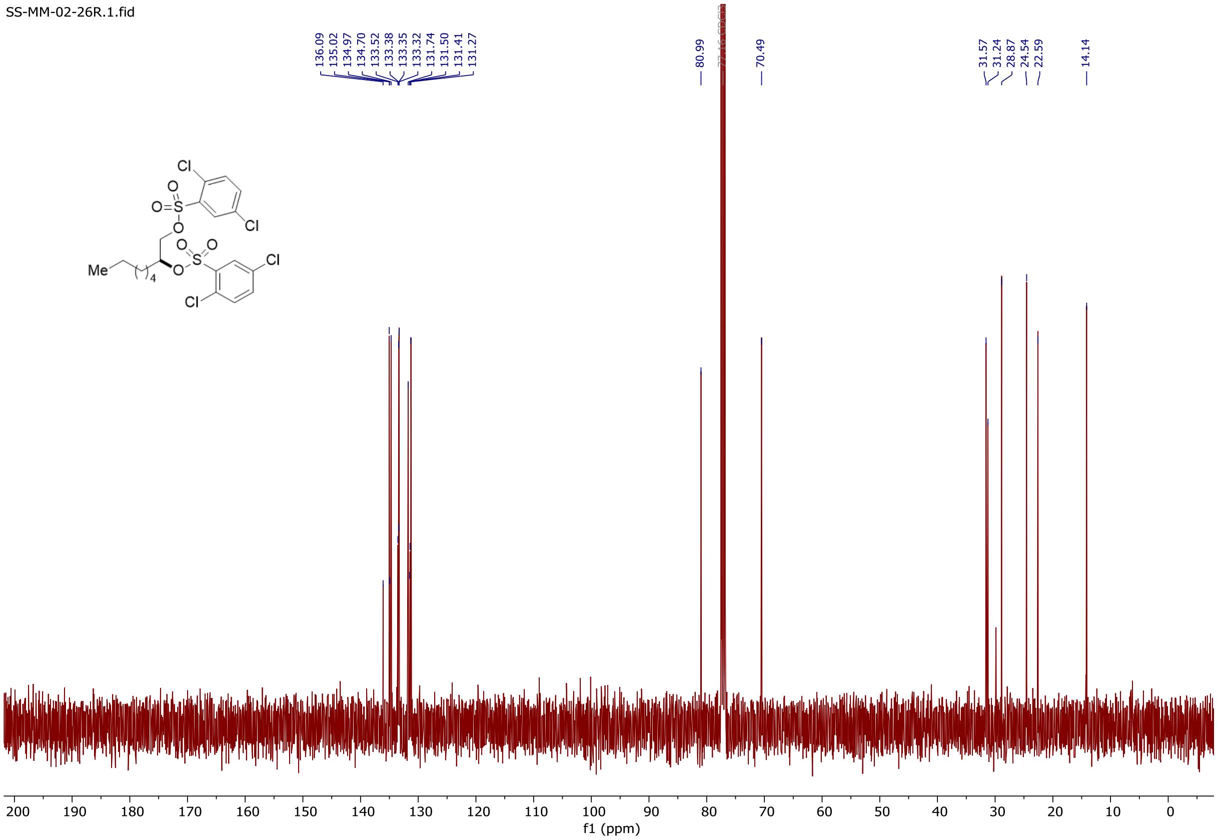

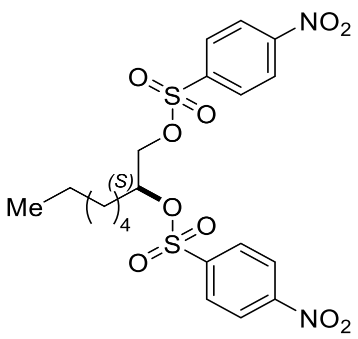

(*S*)-octane-1,2-diyl bis(4-nitrobenzenesulfonate)

Compound 2D: Synthesized using the General Procedure on a 0.2 mmol scale; Purified using a gradient of 2% ethyl acetate in hexane to 20% ethyl acetate in hexane on silica gel; Predominant enantiomer depicted; (colorless oil, 56.0 mg, 0.108 mmol, 54% yield, 91% ee).

^{1}H NMR (400 MHz, $CDCl_3$) δ 8.43 – 8.35 (m, 4H), 8.10 – 8.01 (m, 4H), 4.80 (dtd, *J* = 7.4, 5.9, 3.0 Hz, 1H), 4.23 (dd, *J* = 11.4, 3.1 Hz, 1H), 4.15 (dd, *J* = 11.4, 6.1 Hz, 1H), 1.78 – 1.55 (m, 2H), 1.26 – 1.11 (m, 8H), 0.83 (t, *J* = 7.0 Hz, 3H).

^{13}C{^{1}H} NMR (101 MHz, $CDCl_3$) δ 151.1, 151.0, 142.2, 141.0, 129.4, 129.3, 124.7, 124.6, 80.7, 70.6, 31.5, 31.1, 28.7, 24.7, 22.5, 14.0.

IR υ 2922, 1529, 1349, 1185, 909, 683 cm^{-1}.

HRMS (ESI) m/z = $[M + Na]^+$ Calcd $C_{20}H_{24}N_2O_{10}S_2Na^+$ 539.0770. Found 539.0753 (3.2 ppm error).

Specific Rotation: $[\alpha]_D^{23}$ = + 2.68 (c = 2.8 g/100 mL, $CHCl_3$, 91% ee).

Stereochemistry assigned by analogy to an authentic sample of 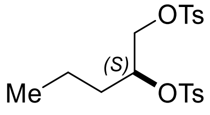

.

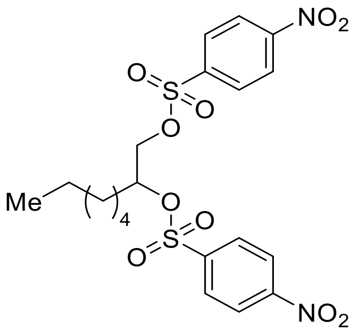

Racemic Sample: HPLC (IC-3, Isopropanol/hexanes = 20/80, flow rate = 1 mL/min, I = 254 nm), t_R = 107.9 min, 137.4 min.

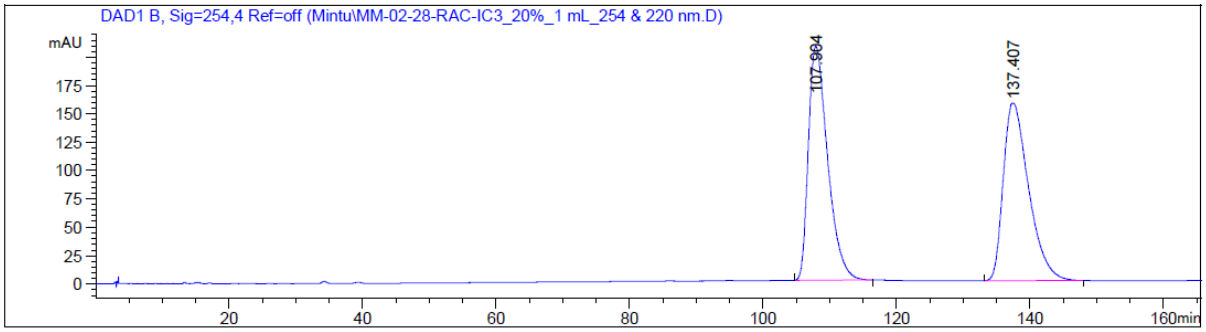

Peak #	RetTime [min]	Type	Width [min]	Area [mAU*s]	Height [mAU]	Area %
1	107.904	BB	2.6959	4.13749e4	206.46912	49.9923
2	137.407	BB	3.0955	4.13877e4	156.28926	50.0077

Scalemic Sample, +91% ee: HPLC (IC-3, Isopropanol/hexanes = 20/80, flow rate = 1 mL/min, I = 254 nm), t_R = 108.9 min, 138.1 min.

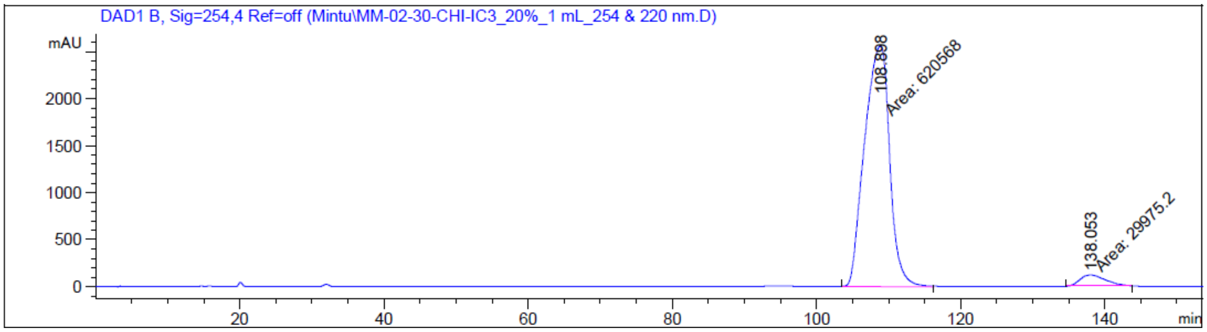

Peak #	RetTime [min]	Type	Width [min]	Area [mAU*s]	Height [mAU]	Area %
1	108.898	MM	4.0432	6.20568e5	2558.05103	95.3923
2	138.053	MM	4.1670	2.99752e4	119.89244	4.6077

Compound 2D ($CDCl_3$, ^{1}H NMR: 400 MHz, ^{13}C{^{1}H} NMR: 101 MHz)

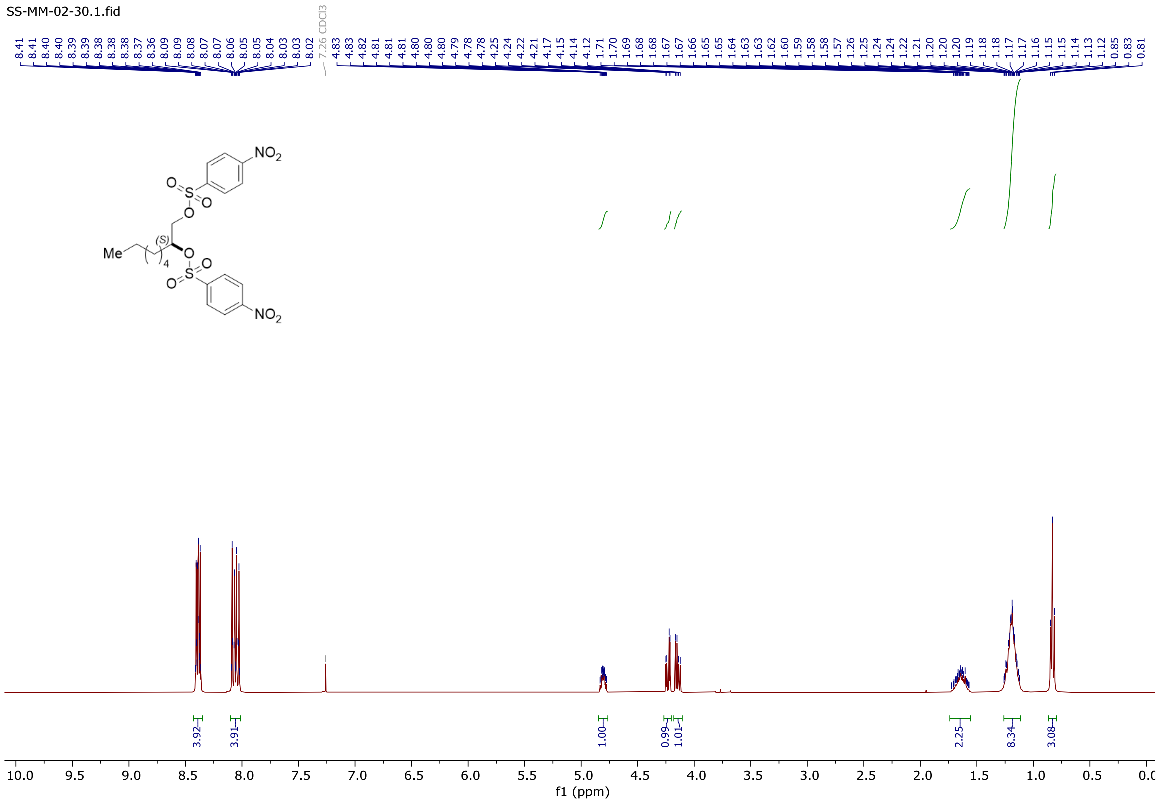

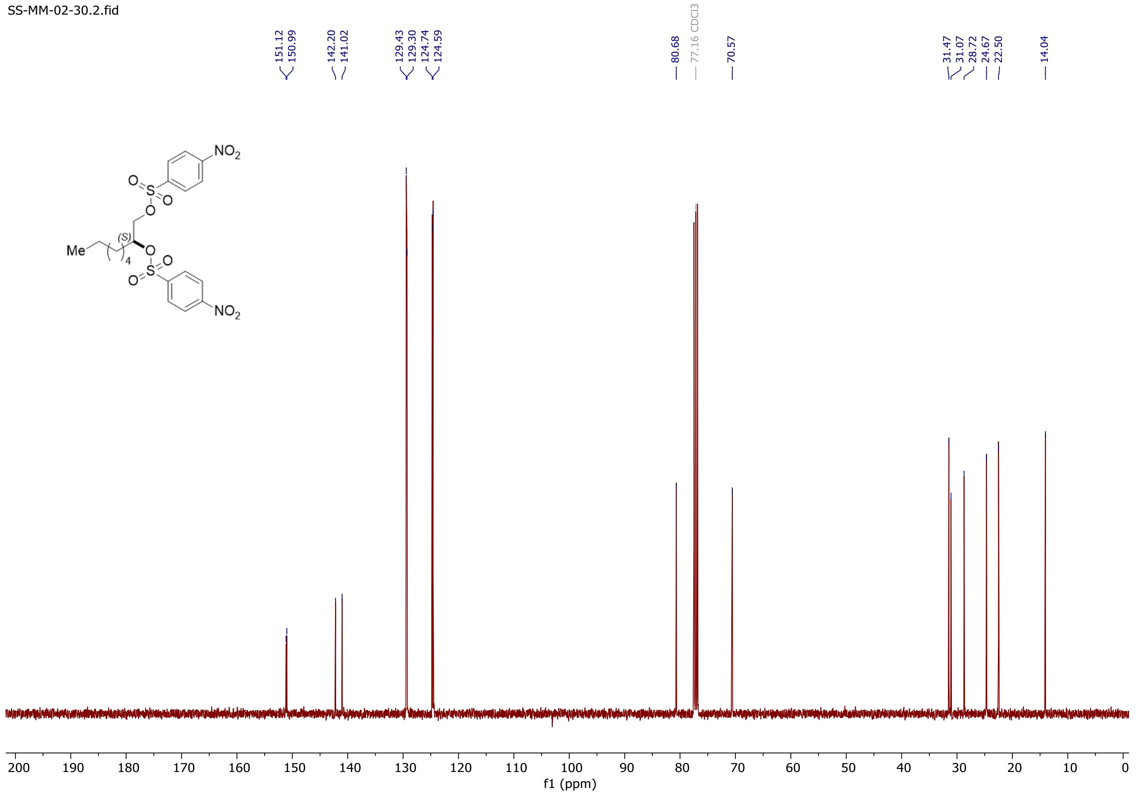

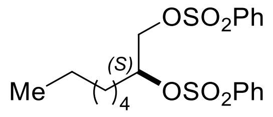

(*S*)-octane-1,2-diyl dibenzenesulfonate

Compound 2E: Synthesized using the General Procedure on a 0.2 mmol scale; Purified using a gradient of 2% ethyl acetate in hexane to 8% ethyl acetate in hexane on silica gel; Predominant enantiomer depicted; (colorless oil, 56.0 mg, 0.131 mmol, 66% yield, 87% ee).

^{1}H NMR (400 MHz, $CDCl_3$) δ 7.89 – 7.77 (m, 4H), 7.70 – 7.60 (m, 2H), 7.58 – 7.48 (m, 4H), 4.62 (tdd, *J* = 7.1, 5.2, 4.1 Hz, 1H), 4.11 – 4.00 (m, 2H), 1.70 – 1.51 (m, 2H), 1.25 – 1.08 (m, 8H), 0.83 (t, *J* = 7.1 Hz, 3H).

^{13}C{^{1}H} NMR (101 MHz, $CDCl_3$) δ 136.6, 135.4, 134.2, 134.0, 129.5, 129.3, 128.0, 127.9, 79.3, 69.7, 31.5, 31.1, 28.7, 24.5, 22.5, 14.1.

IR υ 3054, 2988, 2930, 2861, 2305, 1449, 1369, 1265, 1188, 1096, 912, 898, 740, 706, 588 cm^{-1}.

HRMS (ESI) m/z = $[M + Na]^+$ Calcd $C_{20}H_{26}O_6S_2Na^+$ 449.1069. Found 449.1052 (3.8 ppm error).

Specific Rotation: $[\alpha]_D^{22}$ = –2.2 (c = 2.8 g/100 mL, $CHCl_3$, 87% ee).

Stereochemistry assigned by analogy to an authentic sample of 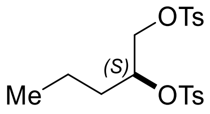 .

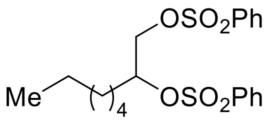

Racemic Sample: HPLC (IC-3, Isopropanol/hexanes = 10/90, flow rate = 1 mL/min, I = 254 nm), t_R = 49.6 min, 57.9 min.

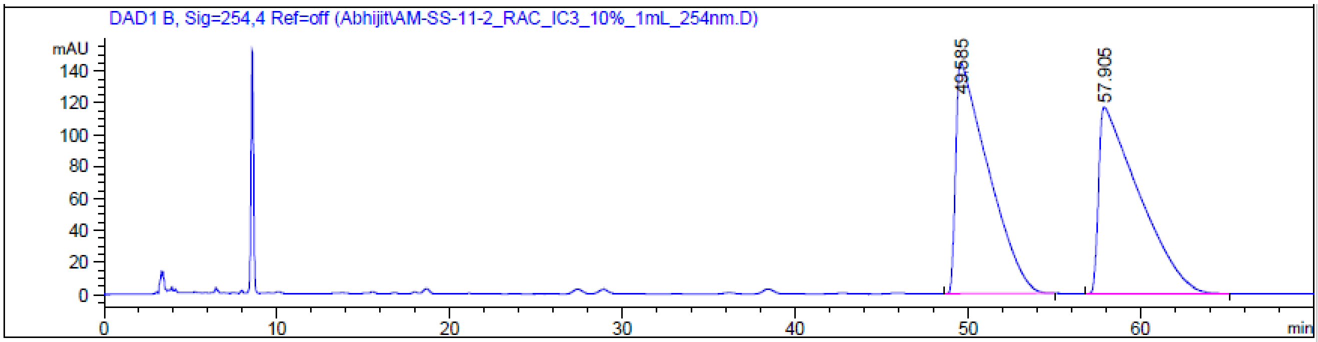

Peak #	RetTime [min]	Type	Width [min]	Area [mAU*s]	Height [mAU]	Area %
1	49.585	BB	1.7075	1.89695e4	144.69295	49.9584
2	57.905	BB	2.0439	1.90011e4	116.67696	50.0416

Scalemic Sample, +87% ee: HPLC (IC-3, Isopropanol/hexanes = 10/90, flow rate = 1 mL/min, I = 254 nm), t_R = 44.4 min, 57.1 min.

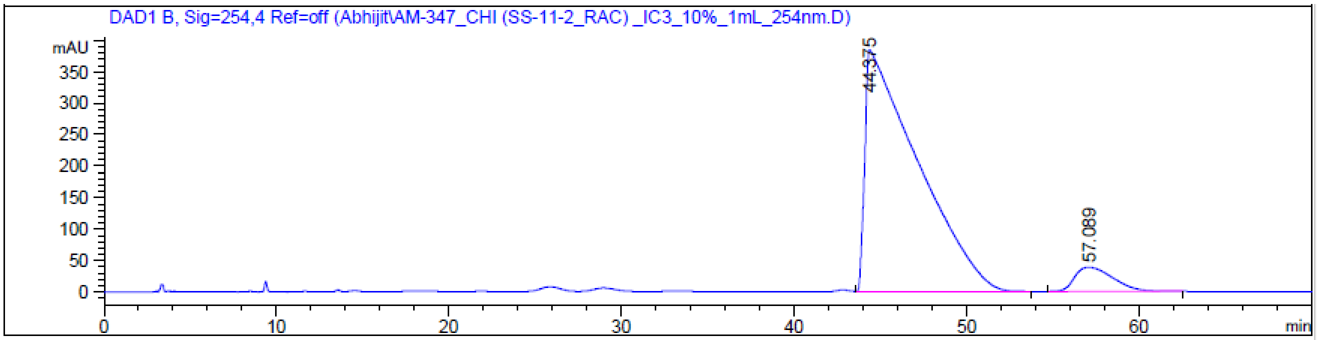

Peak #	RetTime [min]	Type	Width [min]	Area [mAU*s]	Height [mAU]	Area %
1	44.375	BB	2.5968	8.17462e4	383.50882	93.2744
2	57.089	BB	1.8407	5894.34912	38.44508	6.7256

Compound 2E ($CDCl_3$, ^{1}H NMR: 400 MHz, $^{13}C\{^{1}H\}$ NMR: 101 MHz)

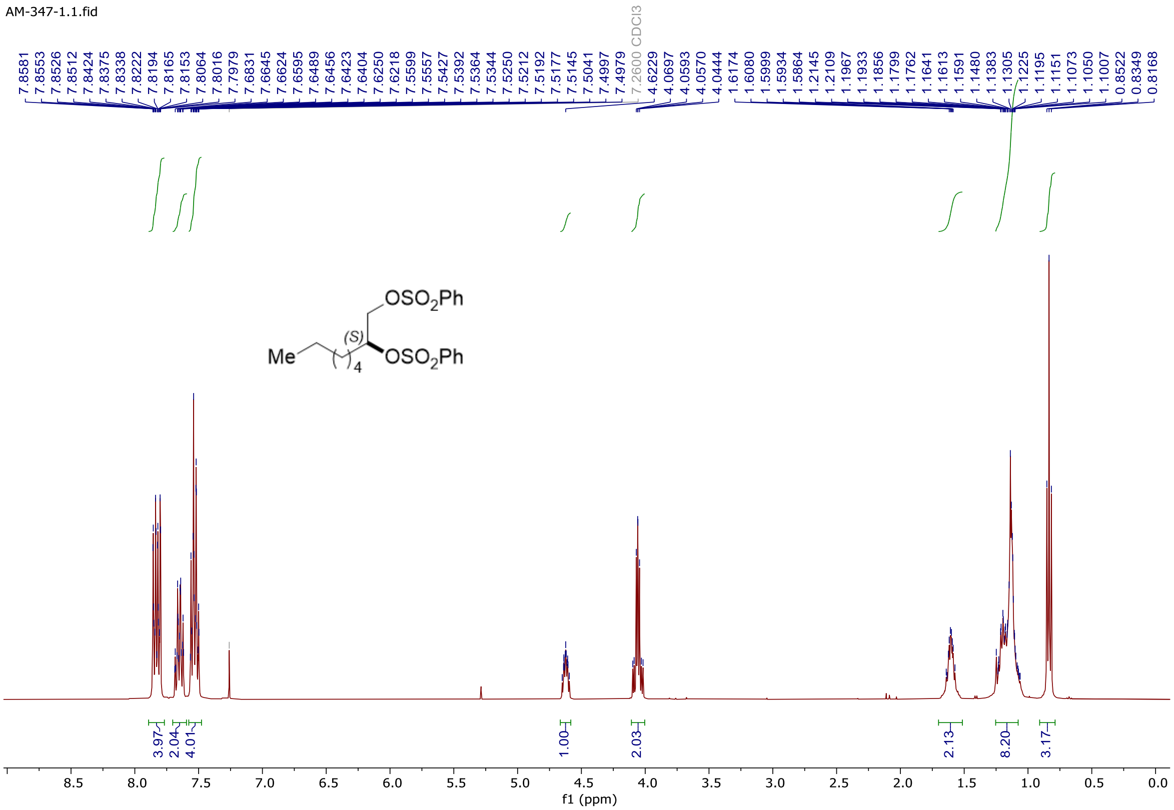

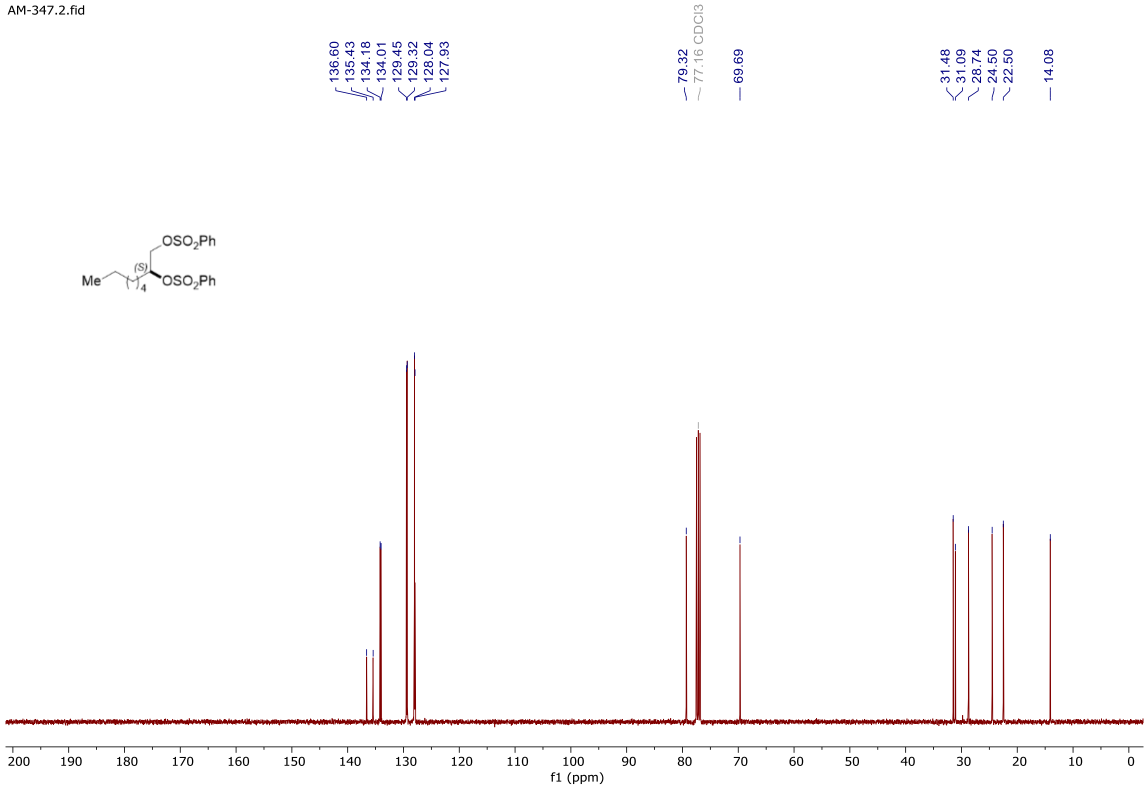

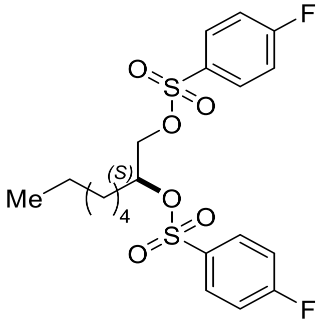

(*S*)-octane-1,2-diyl bis(4-fluorobenzenesulfonate)

Compound 2F: Synthesized using the General Procedure on a 0.2 mmol scale; Purified using a gradient of 2% ethyl acetate in hexane to 6% ethyl acetate in hexane on silica gel; Predominant enantiomer depicted; (colorless oil, 66.0 mg, 0.142 mmol, 71% yield, 88% ee).

^{1}H NMR (400 MHz, $CDCl_3$) δ 8.00 – 7.87 (m, 4H), 7.34 – 7.23 (m, 4H), 4.72 (dtd, *J* = 7.3, 5.6, 3.7 Hz, 1H), 4.22 – 4.09 (m, 2H), 1.77 – 1.60 (m, 2H), 1.33 – 1.15 (m, 8H), 0.91 (t, *J* = 7.0 Hz, 3H).

^{13}C{^{1}H} NMR (101 MHz, $CDCl_3$) δ 167.3 (d, *J* = 259 Hz), 167.2 (d, *J* = 258 Hz), 132.7 (d, *J* = 3.3 Hz), 131.5 (d, *J* = 3.4 Hz), 130.9 (d, *J* = 9.6 Hz), 130.8 (d, *J* = 9.6 Hz), 116.9 (d, *J* = 18.9 Hz), 116.7 (d, *J* = 18.9 Hz), 79.5, 69.8, 31.5, 31.1, 28.8, 24.6, 22.5, 14.1.

^{19}F{^{1}H} NMR (377 MHz, $CDCl_3$) δ -102.2, -102.6.

IR υ 3045, 2988, 2305, 1593, 1495, 1372, 1265, 1188, 1157, 912, 895, 841, 740, 706, 554 cm^{-1}.

HRMS (ESI) m/z = $[M + Na]^+$ Calcd $C_{20}H_{24}F_2O_6S_2Na^+$ 485.0880. Found 485.0869 (2.3 ppm error).

Specific Rotation: $[\alpha]_D^{23}$ = –6.6 (c = 3.0 g/100 mL, $CHCl_3$, 88% ee).

Stereochemistry assigned by analogy to an authentic sample of 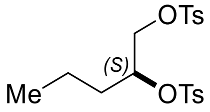

.

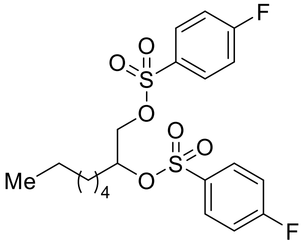

Racemic Sample: HPLC (IC-3, Isopropanol/hexanes = 10/90, flow rate = 1 mL/min, I = 254 nm), t_R = 25.4 min, 31.4 min.

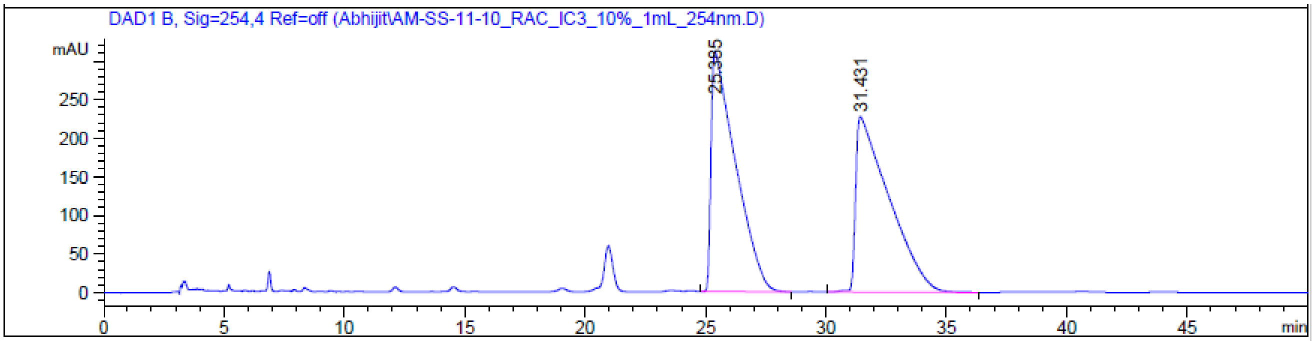

Peak #	RetTime [min]	Type	Width [min]	Area [mAU*s]	Height [mAU]	Area %
1	25.385	BB	0.9261	2.18854e4	310.58755	50.0647
2	31.431	BB	1.2755	2.18288e4	227.07309	49.9353

Scalemic Sample, +88% ee: HPLC (IC-3, Isopropanol/hexanes = 10/90, flow rate = 1 mL/min, I = 254 nm), t_R = 23.6 min, 31.9 min.

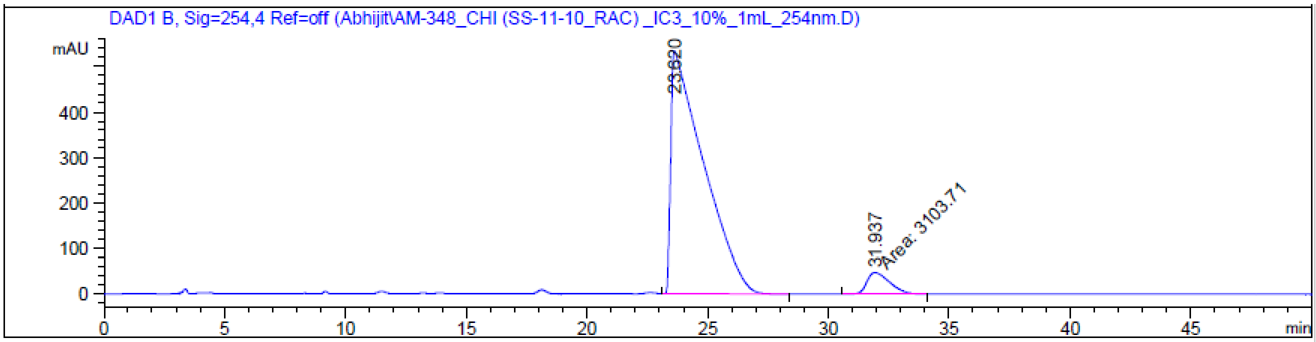

Peak #	RetTime [min]	Type	Width [min]	Area [mAU*s]	Height [mAU]	Area %
1	23.620	BB	1.2048	5.03916e4	533.08899	94.1982
2	31.937	MF	1.0904	3103.70801	47.43927	5.8018

Compound 2F ($CDCl_3$, ^1H NMR: 400 MHz, $^{13}C\{^1H\}$ NMR: 101 MHz)

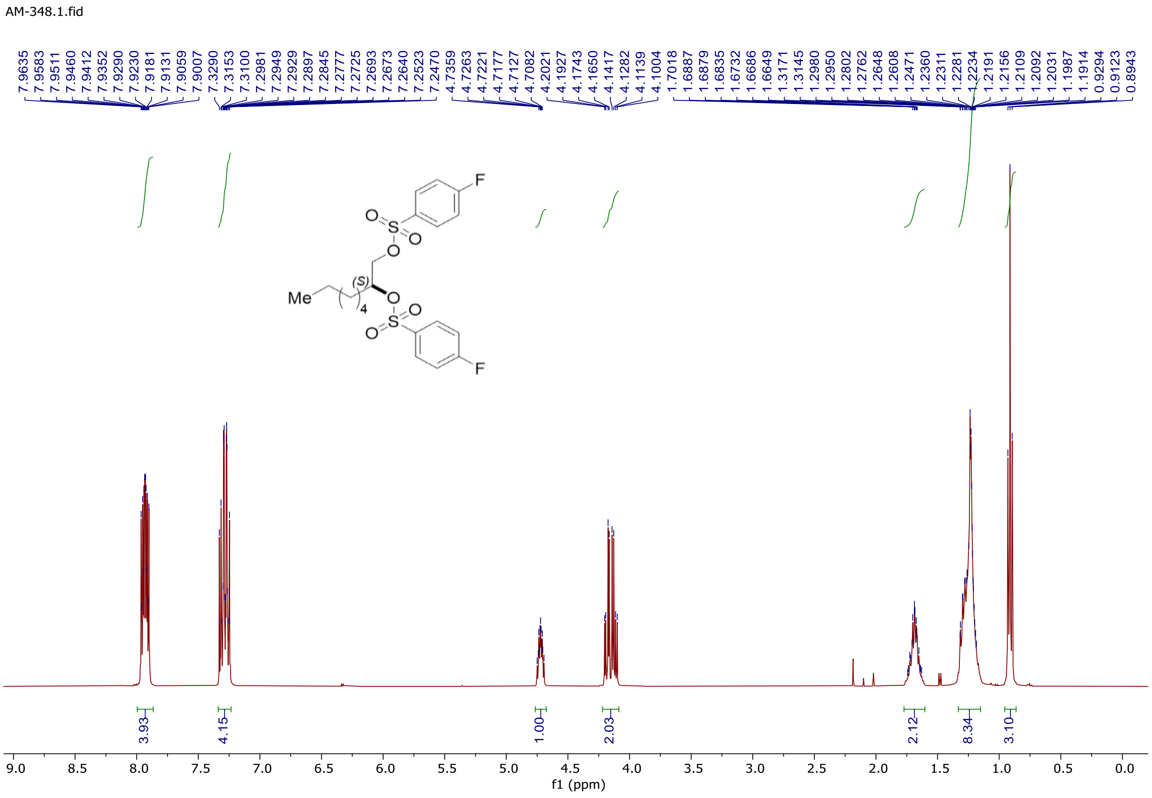

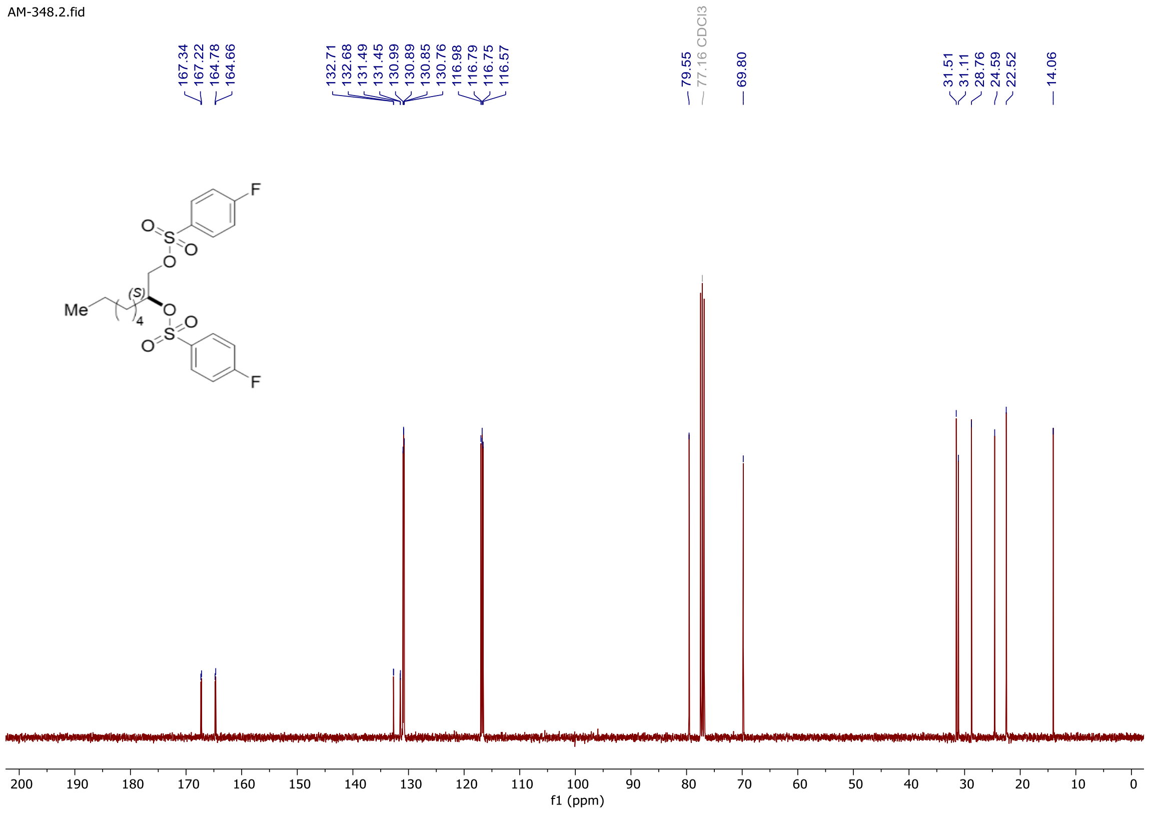

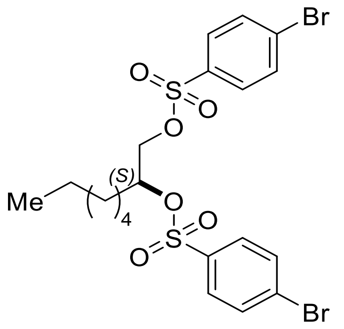

(*S*)-octane-1,2-diyl bis(4-bromobenzenesulfonate)

Compound 2G: Synthesized using the General Procedure on a 0.2 mmol scale; Purified using a gradient of 2% ethyl acetate in hexane to 6% ethyl acetate in hexane on silica gel; Predominant enantiomer depicted; (colorless oil, 93.0 mg, 0.159 mmol, 79% yield, 85% ee).

^{1}H NMR (400 MHz, $CDCl_3$) δ 7.75 – 7.62 (m, 8H), 4.67 (dtd, *J* = 7.3, 5.6, 3.7 Hz, 1H), 4.16 – 4.02 (m, 2H), 1.70 – 1.55 (m, 2H), 1.28 – 1.10 (m, 8H), 0.86 (t, *J* = 7.0 Hz, 3H).

$^{13}C\{^{1}H\}$ NMR (101 MHz, $CDCl_3$) δ 135.7, 134.5, 132.9, 132.7, 129.6, 129.5, 129.4, 129.3, 79.7, 69.9, 31.6, 31.2, 28.8, 24.6, 22.6, 14.1.

IR υ 3045, 2988, 2305, 1420, 1375, 1266, 1188, 895, 740, 611 cm^{-1}.

HRMS (ESI) m/z = $[M + Na]^+$ Calcd $C_{20}H_{24}Br_2O_6S_2Na^+$ 604.9279. Found 604.9310 (5.1 ppm error).

Specific Rotation: $[\alpha]_D^{23}$ = –6.8 (c = 1.7 g/100 mL, $CHCl_3$, 85% ee).

Stereochemistry assigned by analogy to an authentic sample of 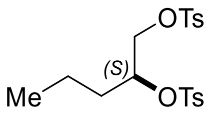

.

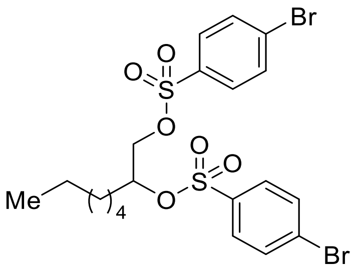

Racemic Sample: HPLC (IC-3, Isopropanol/hexanes = 10/90, flow rate = 1 mL/min, l = 254 nm), t_R = 30.5 min, 38.7 min.

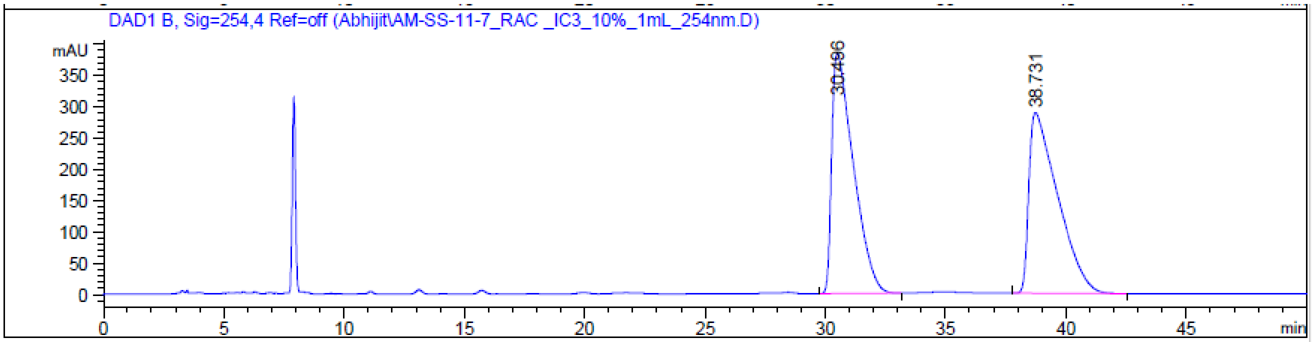

Peak #	RetTime [min]	Type	Width [min]	Area [mAU*s]	Height [mAU]	Area %
1	30.496	BB	0.9067	2.39788e4	384.98730	49.8385
2	38.731	BB	1.1599	2.41341e4	289.10162	50.1615

Scalemic Sample, +85% ee: HPLC (IC-3, Isopropanol/hexanes = 10/90, flow rate = 1 mL/min, l = 254 nm), t_R = 30.2 min, 40.7 min.

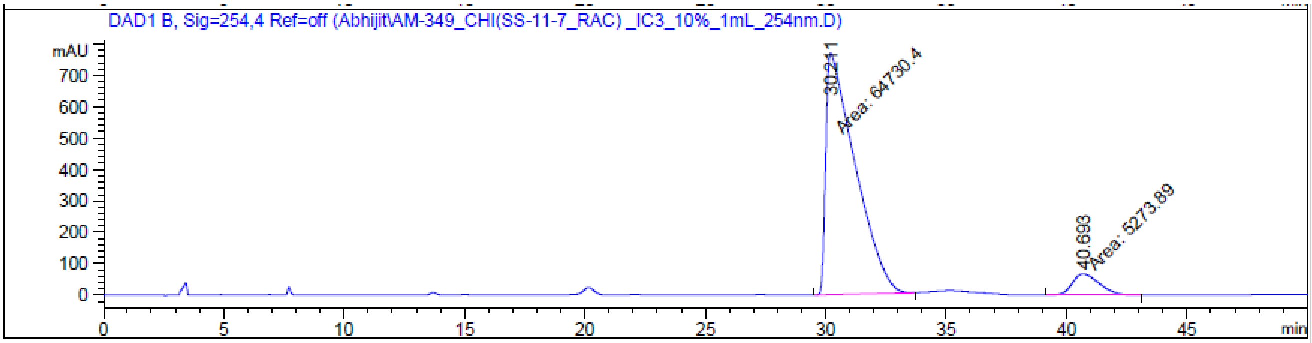

Peak #	RetTime [min]	Type	Width [min]	Area [mAU*s]	Height [mAU]	Area %
1	30.211	MM	1.4035	6.47304e4	768.69720	92.4663
2	40.693	MM	1.3010	5273.89355	67.56327	7.5337

Compound 2G ($CDCl_3$, ^1H NMR: 400 MHz, $^{13}C\{^1H\}$ NMR: 101 MHz)

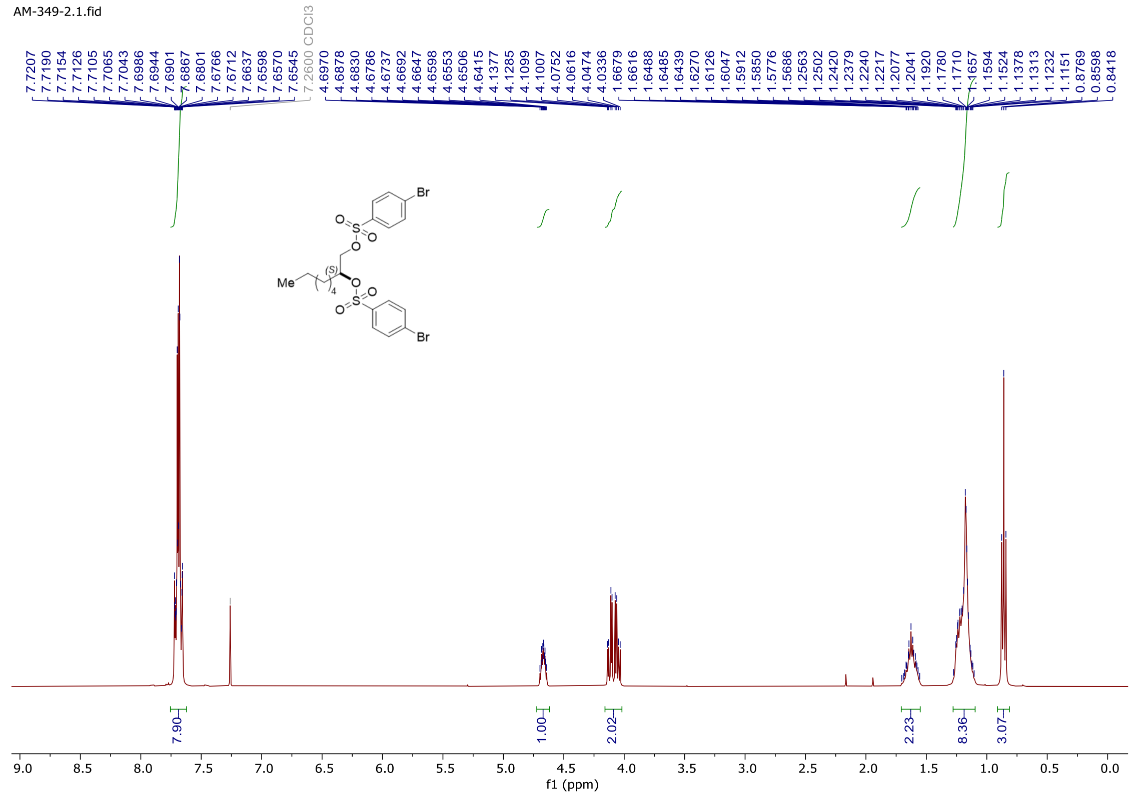

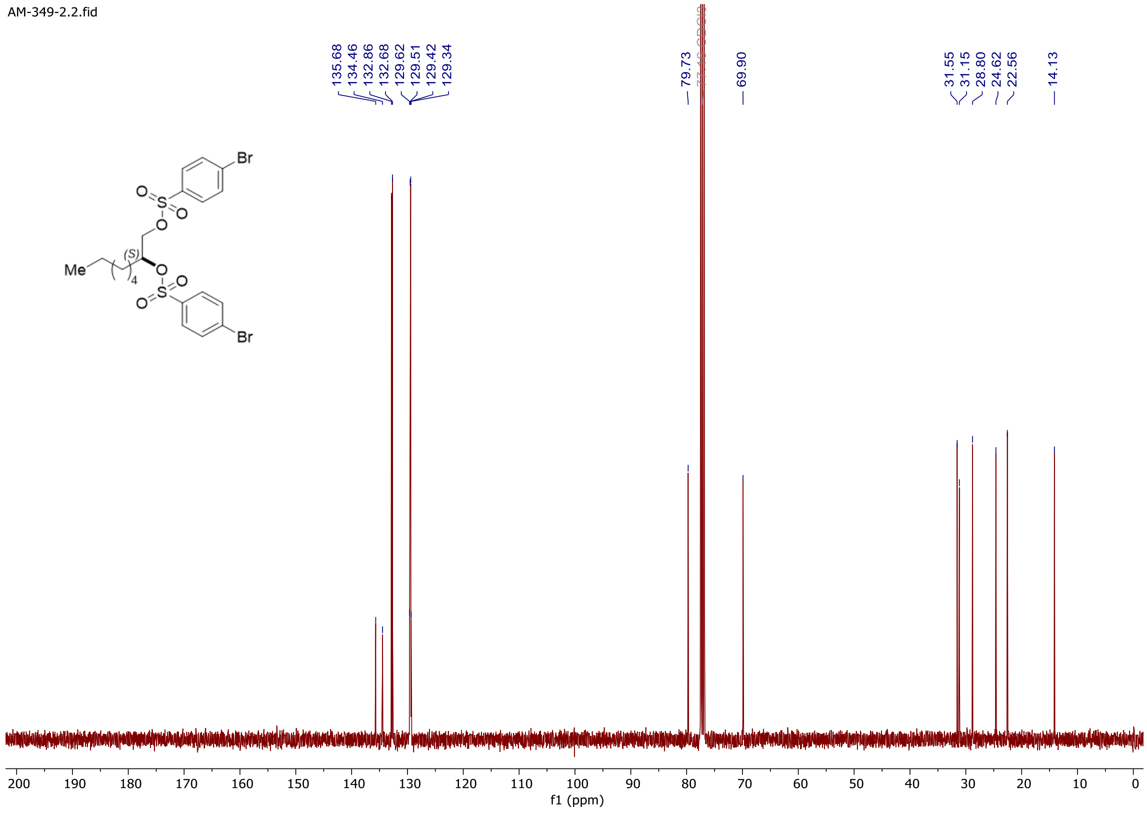

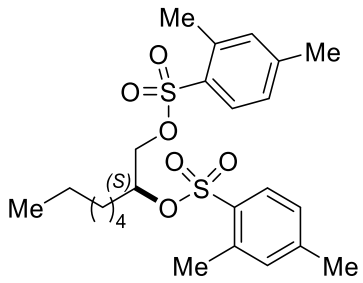

(*S*)-octane-1,2-diyl bis(2,4-dimethylbenzenesulfonate)

Compound 2H: Synthesized using the General Procedure on a 0.2 mmol scale; Purified using a gradient of 2% ethyl acetate in hexane to 6% ethyl acetate in hexane on silica gel; Predominant enantiomer depicted; (colorless oil, 58.6 mg, 0.121 mmol, 61% yield, 89% ee).

^{1}H NMR (400 MHz, $CDCl_3$) δ 7.82 – 7.71 (m, 2H), 7.17 – 7.04 (m, 4H), 4.52 (tt, *J* = 6.7, 4.3 Hz, 1H), 4.05 – 3.91 (m, 2H), 2.54 (s, 3H), 2.50 (s, 3H), 2.39 (s, 3H), 2.37 (s, 3H), 1.70 – 1.55 (m, 2H), 1.26 – 1.08 (m, 8H), 0.84 (t, *J* = 7.1 Hz, 3H).

$^{13}C\{^1H\}$ NMR (101 MHz, $CDCl_3$) δ 145.1, 144.9, 138.5, 138.4, 133.53, 133.48, 132.1, 130.8, 130.3, 129.8, 126.9, 126.8, 78.8, 69.2, 31.5, 31.0, 28.8, 24.4, 22.5, 21.51, 21.49, 20.3, 20.1, 14.1.

IR υ 3045, 2959, 2930, 2859, 2305, 1604, 1455, 1360, 1266, 1185, 909, 821, 740, 706, 666, 557 cm^{-1}.

HRMS (ESI) m/z = $[M + Na]^+$ Calcd $C_{24}H_{34}O_6S_2Na^+$ 505.1695. Found 505.1673 (4.4 ppm error).

Specific Rotation: $[\alpha]_D^{24}$ = –4.8 (c = 2.85 g/100 mL, $CHCl_3$, 89% ee).

Stereochemistry assigned by analogy to an authentic sample of 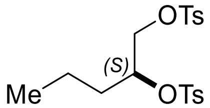

.

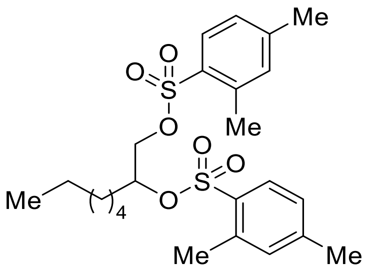

Racemic Sample: HPLC (IC-3, Isopropanol/hexanes = 10/90, flow rate = 1 mL/min, I = 254 nm), t_R = 40.6 min, 44.7 min.

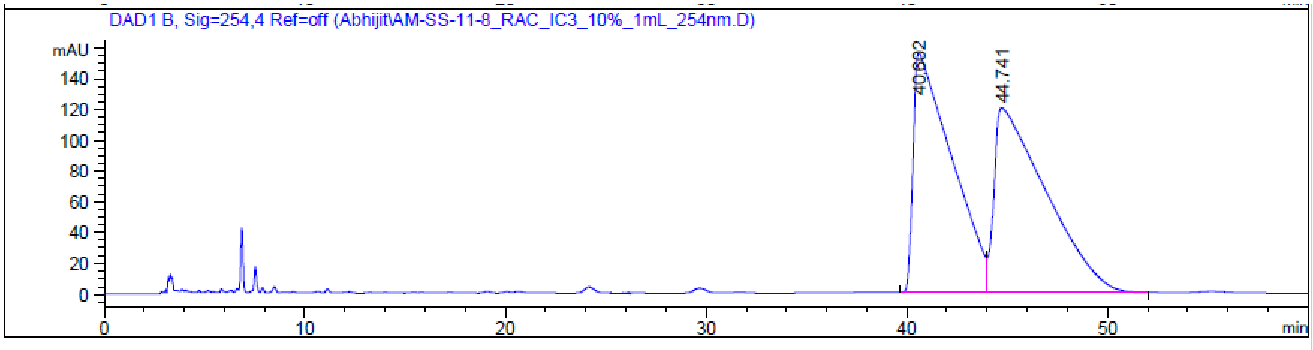

Peak #	RetTime [min]	Type	Width [min]	Area [mAU*s]	Height [mAU]	Area %
1	40.602	BV	1.6960	2.07429e4	155.52531	49.0928
2	44.741	VB	2.2523	2.15095e4	119.70794	50.9072

Scalemic Sample, +89% ee: HPLC (IC-3, Isopropanol/hexanes = 10/90, flow rate = 1 mL/min, I = 254 nm), t_R = 39.3 min, 48.0 min.

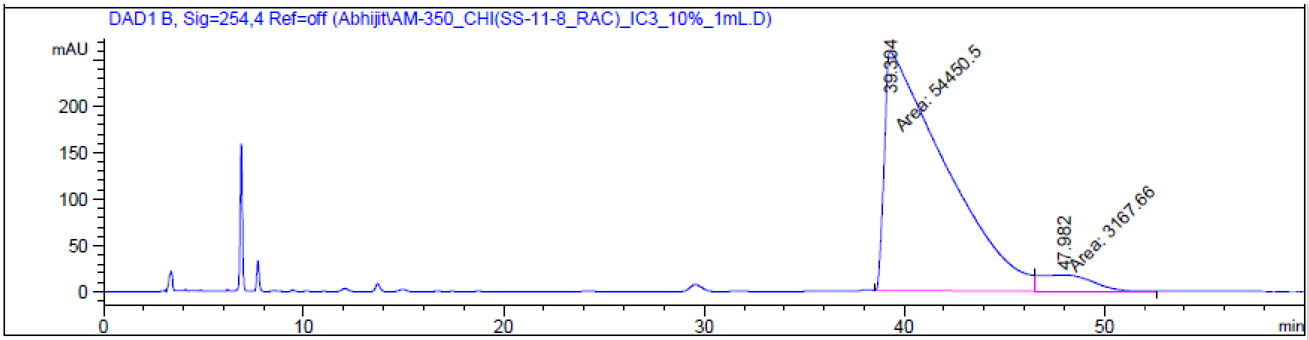

Peak #	RetTime [min]	Type	Width [min]	Area [mAU*s]	Height [mAU]	Area %
1	39.304	MF	3.5151	5.44505e4	258.17401	94.5023
2	47.982	FM	3.0723	3167.66479	17.18426	5.4977

Compound 2H (CDCl$_3$, ^{1}H NMR: 400 MHz, ^{13}C{^{1}H} NMR: 101 MHz)

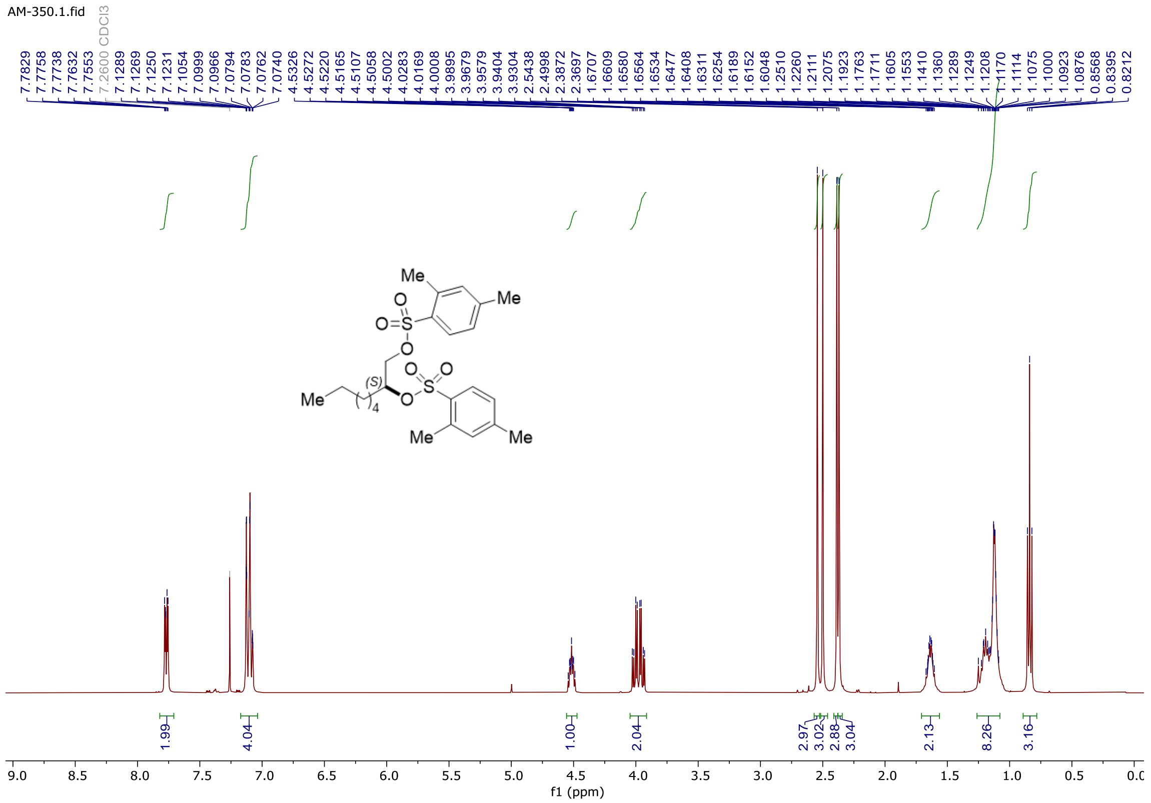

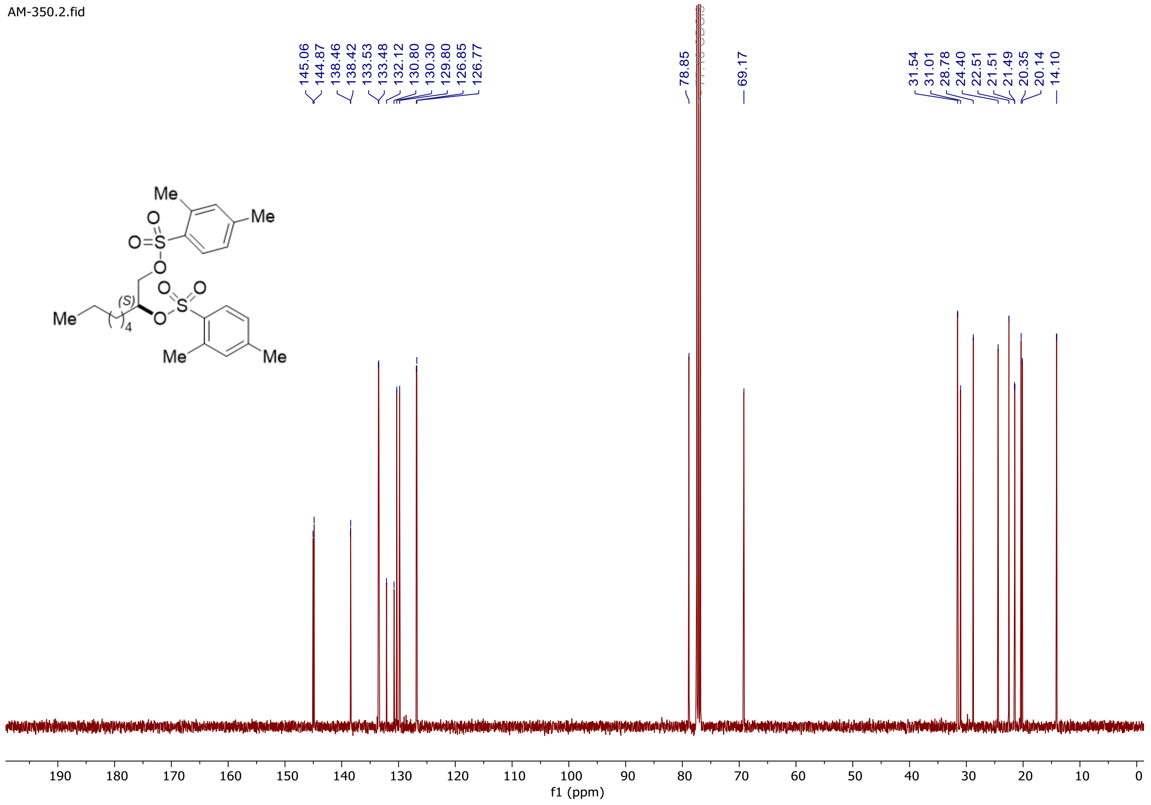

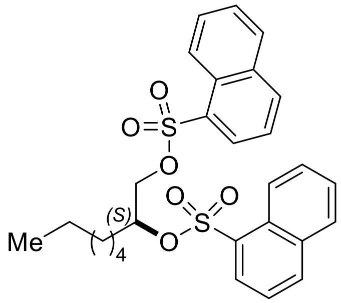

(*S*)-octane-1,2-diyl bis(naphthalene-1-sulfonate)

Compound 2I: Synthesized using the General Procedure on a 0.2 mmol scale; Purified using a gradient of 2% ethyl acetate in hexane to 8% ethyl acetate in hexane on silica gel; Predominant enantiomer depicted; (colorless oil, 55.8 mg, 0.106 mmol, 53% yield, 80% ee).

^{1}H NMR (400 MHz, $CDCl_3$) δ 8.55 – 8.35 (m, 2H), 8.18 – 8.03 (m, 4H), 7.99 – 7.85 (m, 2H), 7.67 – 7.55 (m, 4H), 7.55 – 7.43 (m, 2H), 4.49 (tt, *J* = 6.8, 4.7 Hz, 1H), 4.05 – 3.95 (m, 2H), 1.54 – 1.38 (m, 2H), 1.16 – 0.78 (m, 8H), 0.76 (t, *J* = 7.3 Hz, 3H).

^{13}C{^{1}H} NMR (101 MHz, $CDCl_3$) δ 135.6, 135.5, 134.21, 134.16, 131.7, 130.7, 130.6, 130.1, 129.0, 128.90, 128.86, 128.7, 128.4, 128.3, 127.4, 127.3, 125.1, 124.8, 124.1, 124.0, 79.4, 69.6, 31.3, 30.9, 28.5, 24.1, 22.4, 14.0.

IR υ 3504, 2959, 2931, 2859, 2305, 1510, 1421, 1366, 1266, 1180, 895, 804, 741, 706, 594 cm^{-1}.

HRMS (ESI) m/z = $[M + Na]^+$ Calcd $C_{28}H_{30}O_6S_2Na^+$ 549.1382. Found 549.1392 (1.8 ppm error).

Specific rotation: $[\alpha]_D^{24}$ = –16.2 (c = 2.5 g/100 mL, $CHCl_3$, 80% ee).

Stereochemistry assigned by analogy to an authentic sample of 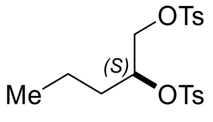 .

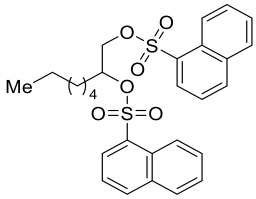

Racemic Sample: SFC (ColumnTek Enantiocel IDH-5, MeOH with 0.1% DEA/CO_2 = 35/65, flow rate = 2 mL/min, I = 220 nm), t_R = 5.4 min, 6.4 min.

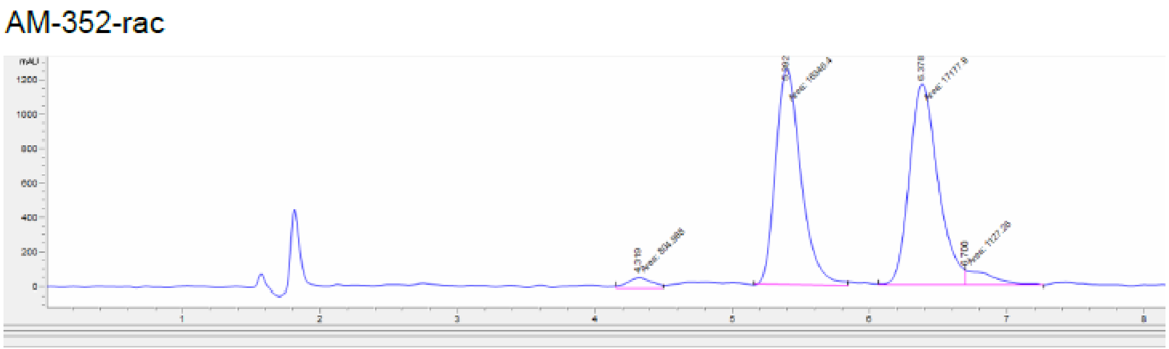

Index	Time (min)	Area (%)
Impurity	4.319	2.270
Peak-1	5.392	46.103
Peak-2	6.378	48.448
Impurity	6.7	3.179
Total		100.00

Scalemic Sample, -80% ee: SFC (ColumnTek Enantiocel IDH-5, MeOH with 0.1% DEA/CO_2 = 35/65, flow rate = 2 mL/min, I = 220 nm), t_R = 5.4 min, 6.4 min.

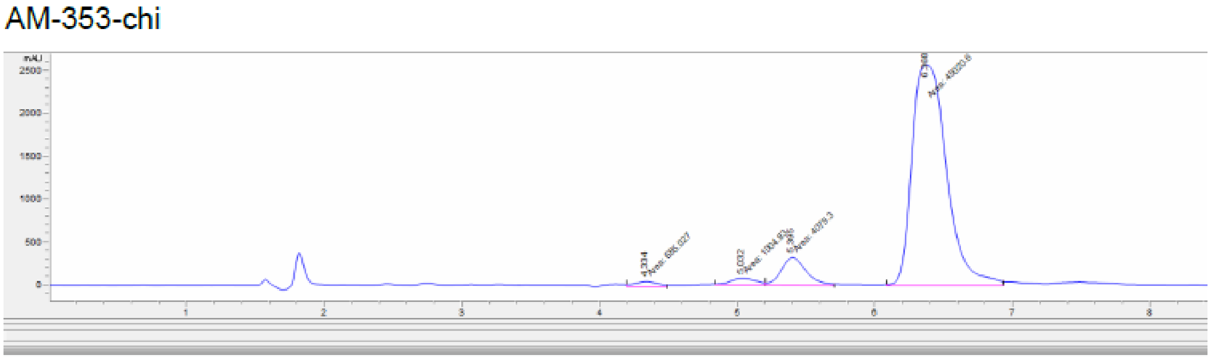

Index	Time (min)	Area (%)
Impurity	4.334	1.349
Impurity	5.032	1.979
Peak-1	5.395	8.032
Peak-2	6.368	88.641
Total		100.00

Compound 2I ($CDCl_3$, ^1H NMR: 400 MHz, $^{13}C\{^1H\}$ NMR: 101 MHz)

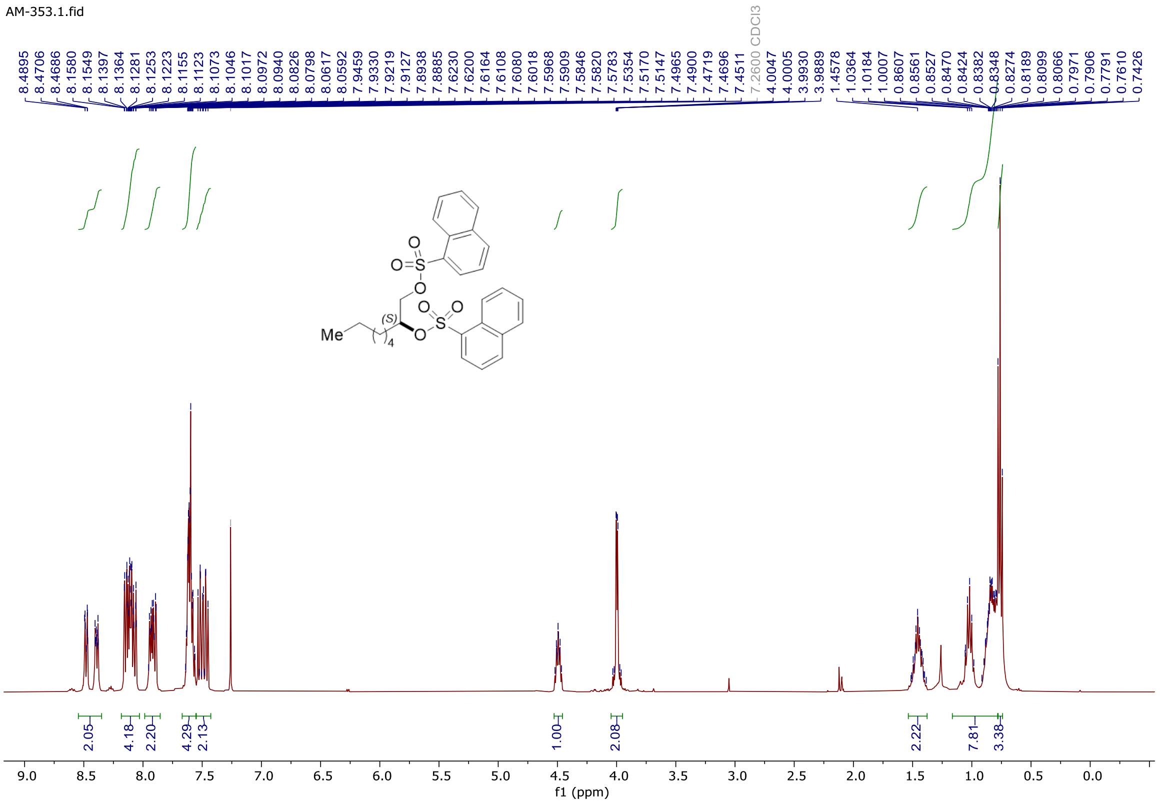

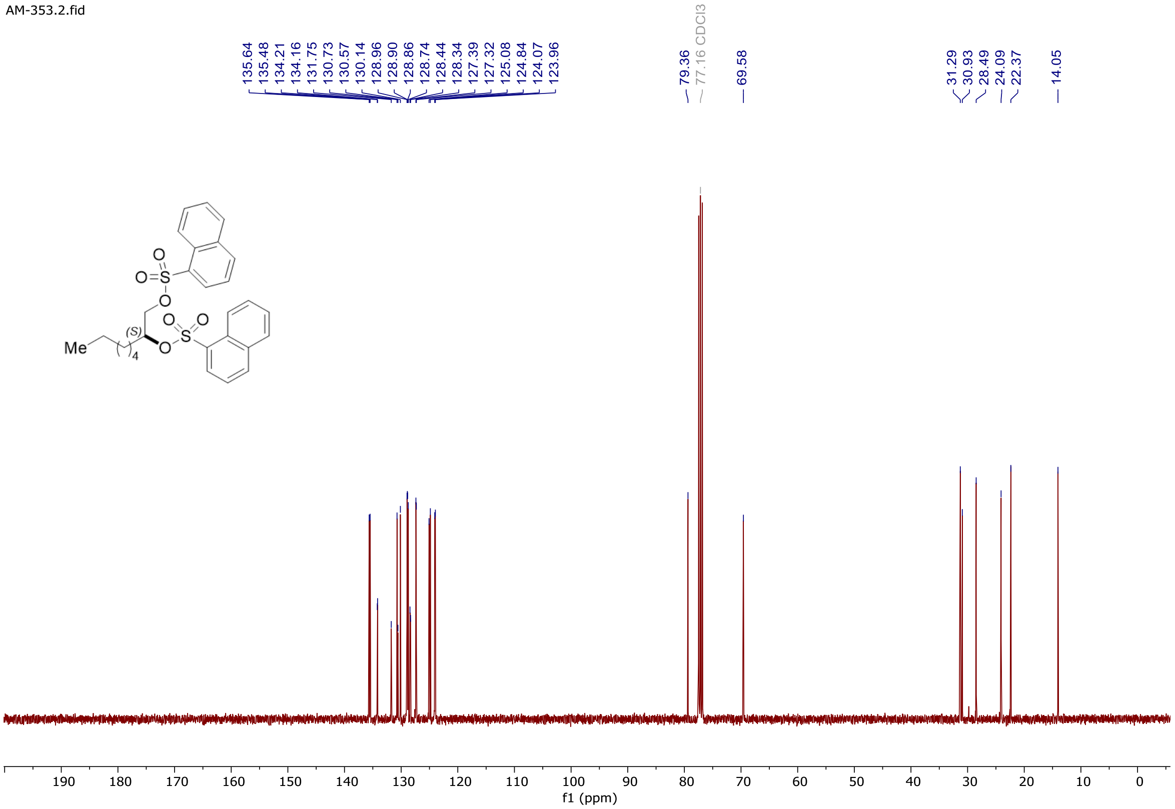

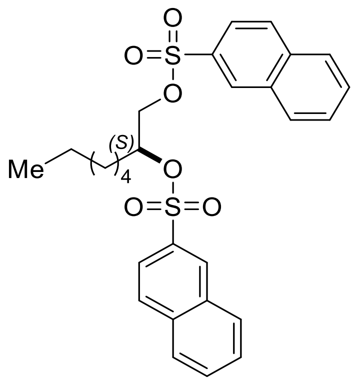

(*S*)-octane-1,2-diyl bis(naphthalene-2-sulfonate)

Compound 2J: Synthesized using the General Procedure on a 0.2 mmol scale; Purified using a gradient of 2% ethyl acetate in hexane to 8% ethyl acetate in hexane on silica gel; Predominant enantiomer depicted; (colorless oil, 44.0 mg, 0.084 mmol, 42% yield, 87% ee).

^{1}H NMR (400 MHz, $CDCl_3$) δ 8.44 – 8.29 (m, 2H), 7.97 – 7.77 (m, 6H), 7.75 – 7.56 (m, 6H), 4.75 – 4.64 (m, 1H), 4.16 – 4.07 (m, 2H), 1.73 – 1.55 (m, 2H), 1.21 – 0.97 (m, 8H), 0.77 (t, *J* = 6.9 Hz, 3H).

^{13}C{^{1}H} NMR (101 MHz, $CDCl_3$) δ 135.4, 135.3, 133.4, 132.2, 132.0, 131.9, 129.9, 129.8, 129.7, 129.6, 129.52, 129.50, 129.48, 128.12, 128.06, 128.0, 127.9, 122.6, 122.4, 79.4, 69.8, 31.5, 31.2, 28.8, 24.5, 22.4, 14.1.

IR υ 3054, 2988, 2305, 1420, 1366, 1266, 1180, 895, 740, 706, 554 cm^{-1}.

HRMS (ESI) m/z = $[M + Na]^+$ Calcd $C_{28}H_{30}O_6S_2Na^+$ 549.1382. Found 549.1395 (2.4 ppm error).

Specific rotation: $[\alpha]_D^{23}$ = –12.5 (c = 2.05 g/100 mL, $CHCl_3$, 87% ee).

Stereochemistry assigned by analogy to an authentic sample of 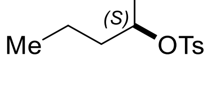 .

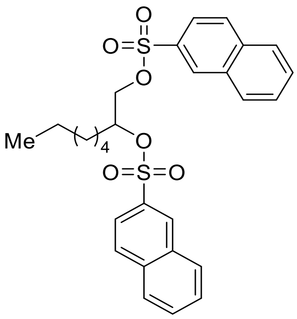

Racemic Sample: HPLC (IC-3, Isopropanol/hexanes = 20/80, flow rate = 1 mL/min, I = 254 nm), t_R = 42.3 min, 49.5 min.

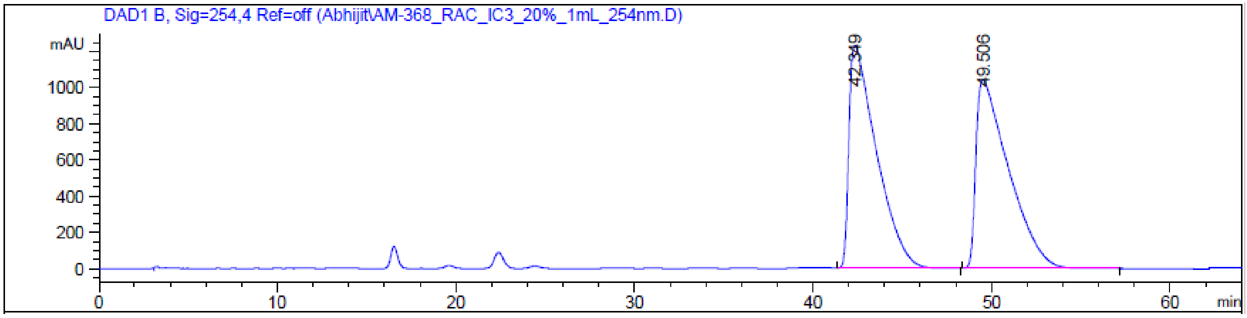

Peak #	RetTime [min]	Type	Width [min]	Area [mAU*s]	Height [mAU]	Area %
1	42.349	BB	1.4563	1.31556e5	1231.48145	49.9254
2	49.506	BB	1.7285	1.31949e5	1042.63965	50.0746

Scalemic Sample, +87% ee: HPLC (IC-3, Isopropanol/hexanes = 20/80, flow rate = 1 mL/min, I = 254 nm), t_R = 42.6 min, 51.8 min.

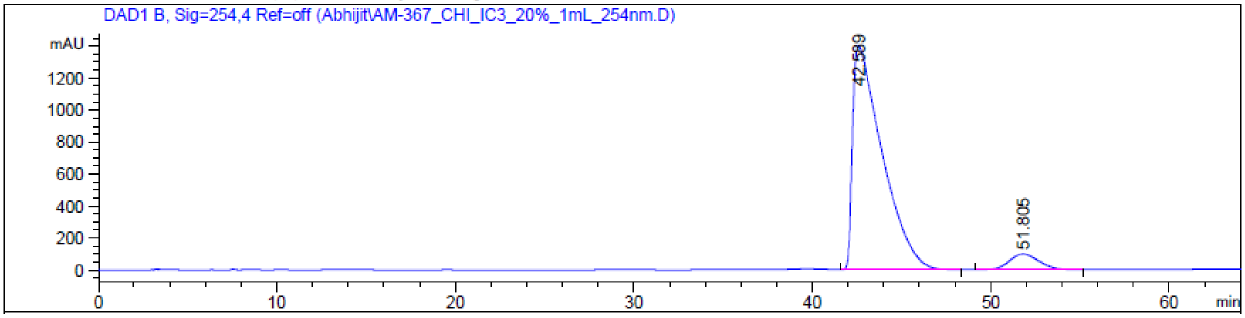

Peak #	RetTime [min]	Type	Width [min]	Area [mAU*s]	Height [mAU]	Area %
1	42.589	BB	1.5758	1.60827e5	1402.49988	93.6004
2	51.805	BB	1.6732	1.09960e4	95.93475	6.3996

Compound 2J ($CDCl_3$, ^{1}H NMR: 400 MHz, $^{13}C\{1H\}$ NMR: 101 MHz)

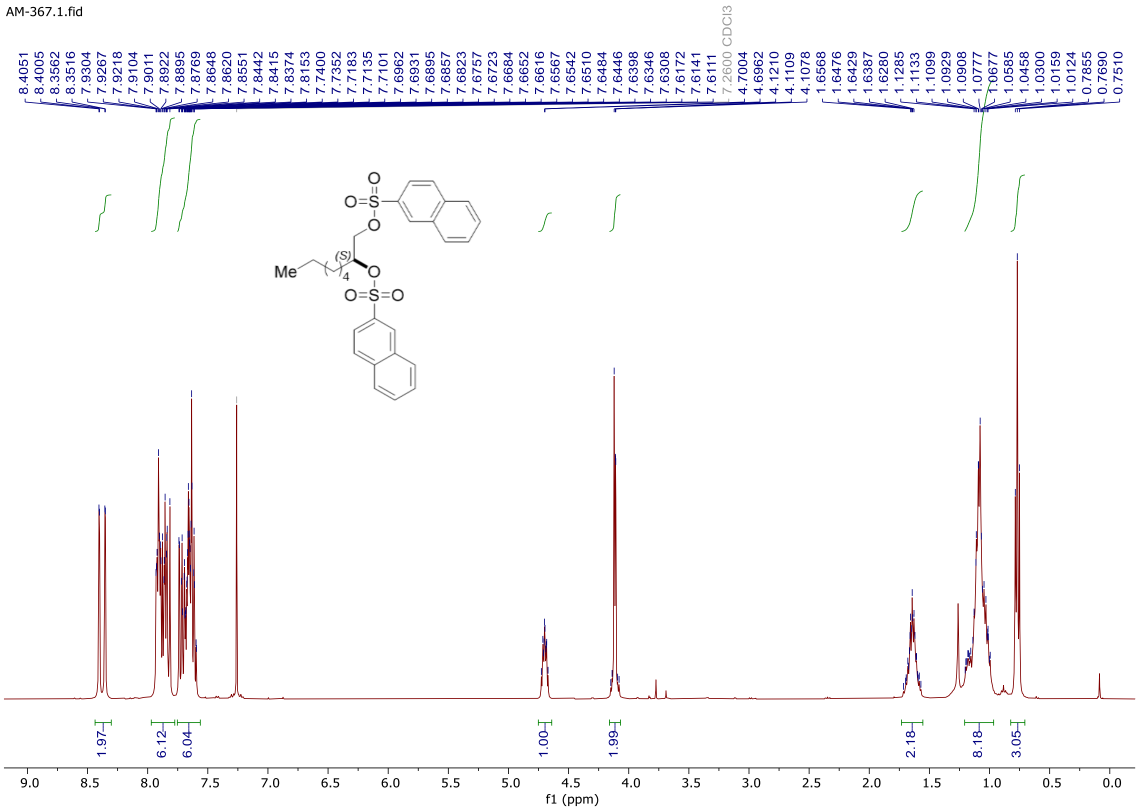

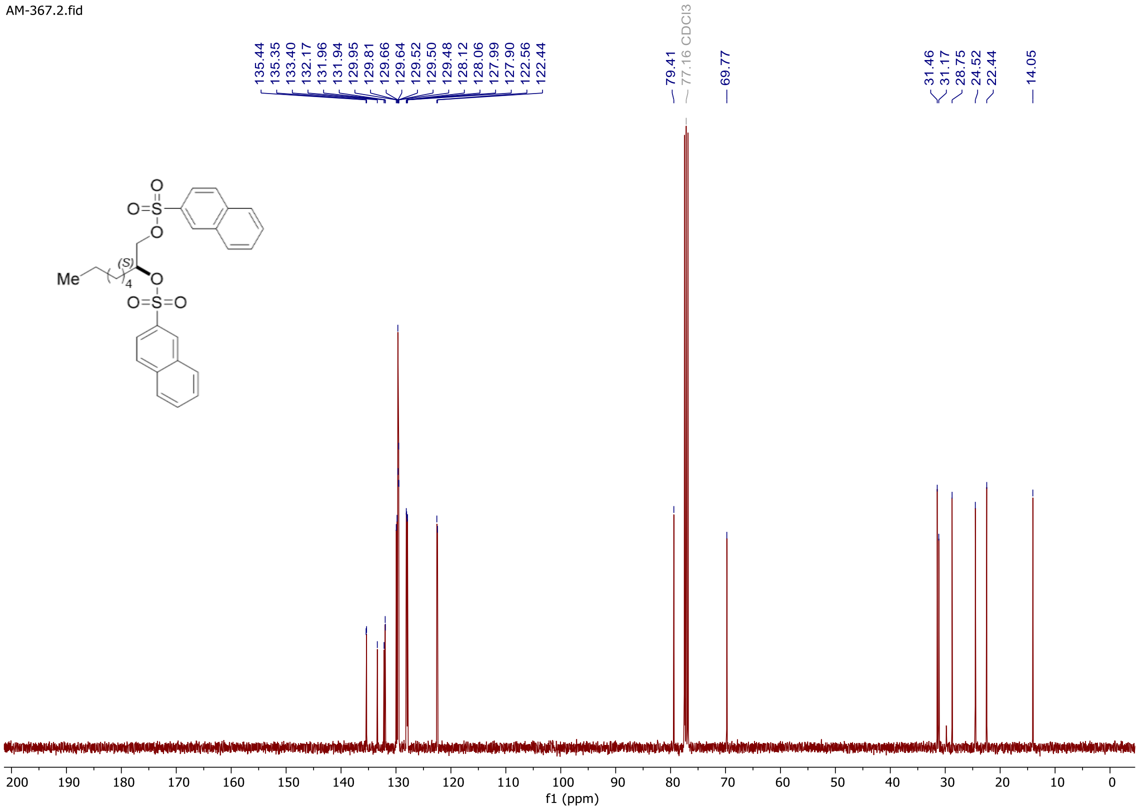

Substrate-3A-C ($CDCl_3$, ^{1}H NMR: 400 MHz, $^{13}C\{^{1}H\}$ NMR: 101 MHz)

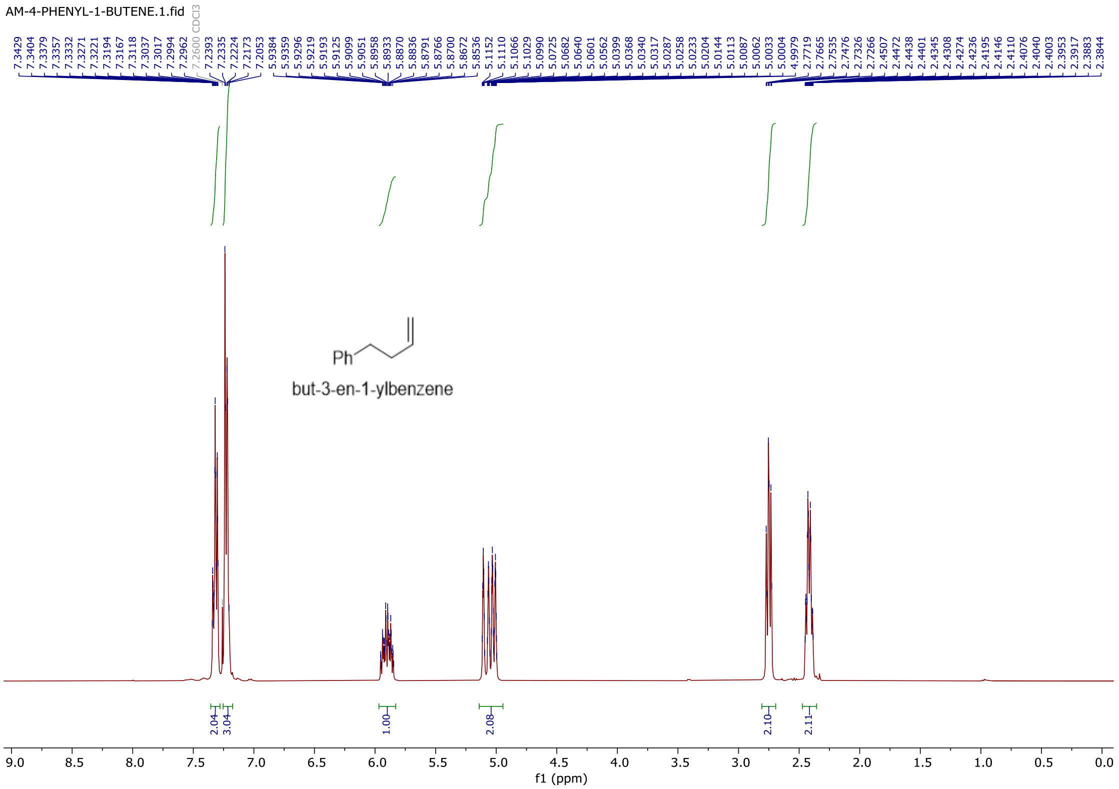

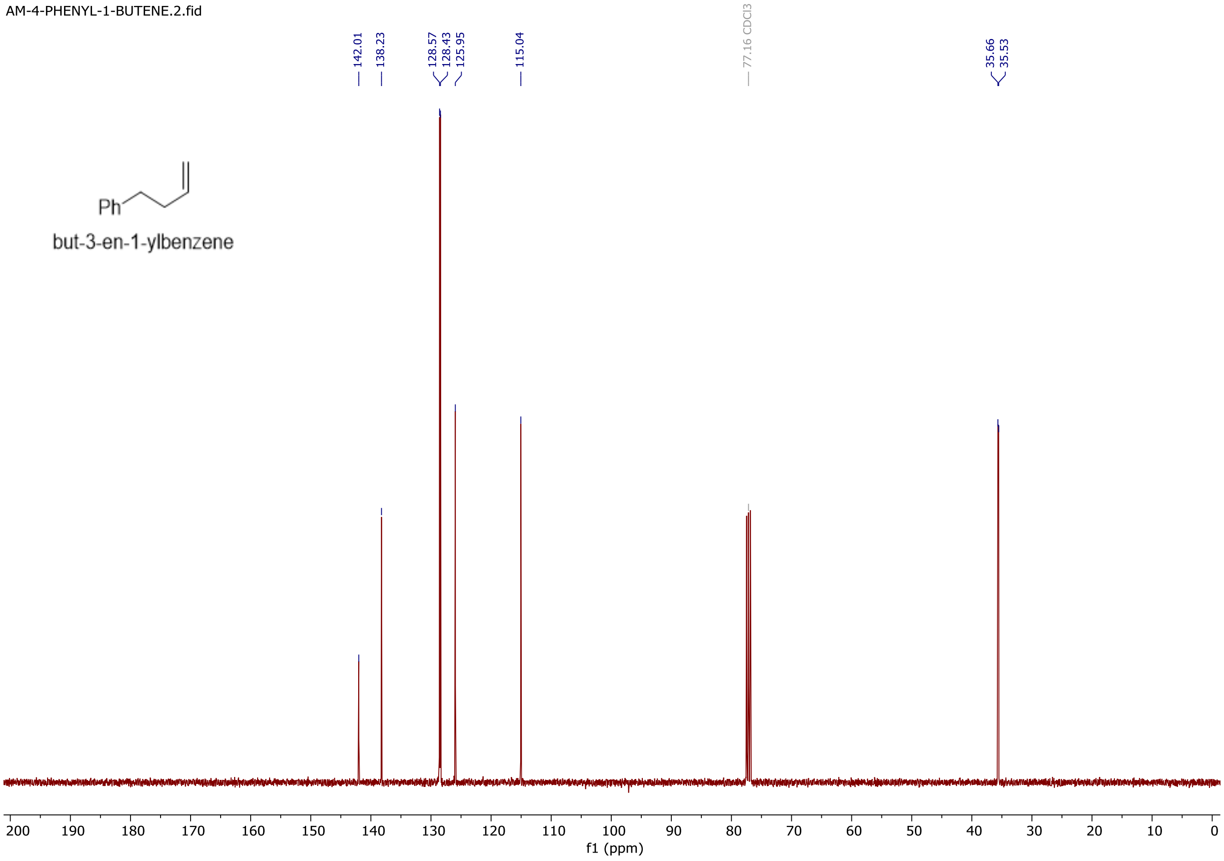

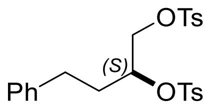

(*S*)-4-phenylbutane-1,2-diyl bis(4-methylbenzenesulfonate)

Compound 3A: Synthesized using the General Procedure on a 0.2 mmol scale; Purified using a gradient of 2% ethyl acetate in hexane to 20% ethyl acetate in hexane on silica gel; Predominant enantiomer depicted; (colorless oil, 59.8 mg, 0.126 mmol, 63% yield, 86% ee).

^{1}H NMR (400 MHz, $CDCl_3$) δ 7.77 – 7.65 (m, 4H), 7.36 – 7.28 (m, 4H), 7.27 – 7.22 (m, 2H), 7.22 – 7.16 (m, 1H), 7.06 – 6.99 (m, 2H), 4.63 (ddt, *J* = 7.1, 5.6, 4.5 Hz, 1H), 4.11 – 4.01 (m, 2H), 2.62 – 2.47 (m, 2H), 2.45 (overlapping s, 6H), 2.03 – 1.86 (m, 2H).

^{13}C{^{1}H} NMR (101 MHz, $CDCl_3$) δ 145.3, 145.2, 140.1, 133.5, 132.4, 130.1, 130.0, 128.6, 128.3, 128.1, 128.0, 126.4, 78.2, 69.4, 32.7, 30.7, 21.8.

IR υ 3054, 2988, 2305, 1420, 1369, 1266, 1191, 1177, 895, 740, 706, 554 cm^{-1}.

HRMS (ESI) m/z = $[M + Na]^+$ Calcd $C_{24}H_{26}O_6S_2Na^+$ 497.1069. Found 497.1060 (1.8 ppm error).

Specific rotation: $[\alpha]_D^{24}$ = –9.2 (c = 0.4 g/100 mL, $CHCl_3$, 86% ee).

Stereochemistry assigned by analogy to an authentic sample of 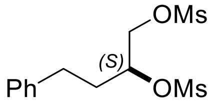 .

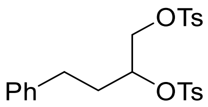

Racemic Sample: SFC (AS-H, EtOH with 0.1% DEA/CO_2 = 30/70, flow rate = 2 mL/min, I = 220 nm), t_R = 4.2 min, 5.2 min.

AM-SS-10-173-RAC

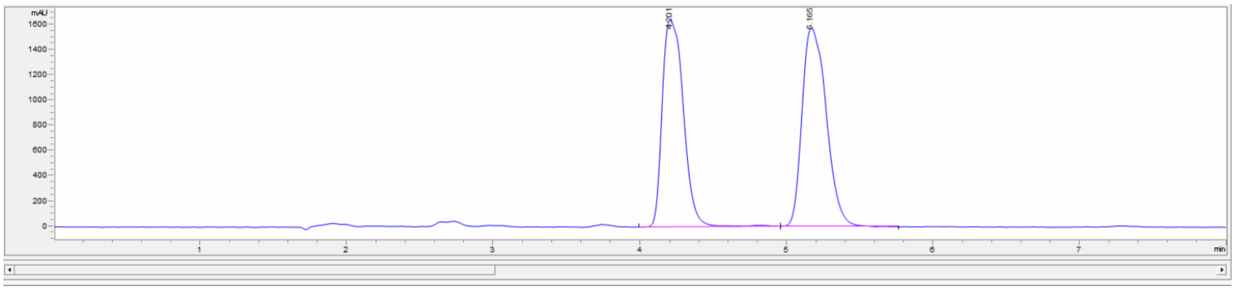

#	Time	Type	Area	Height	Width	Area%	Symmetry
1	4.201	BV R	16687.9	1650.5	0.1613	47.769	0.58
2	5.165	VV R	18246.7	1585.3	0.1642	52.231	0.61

Scalemic Sample, -86% ee: SFC (AS-H, EtOH with 0.1% DEA/CO_2 = 30/70, flow rate = 2 mL/min, I = 220 nm), t_R = 4.2 min, 5.2 min.

AM-357-CHI

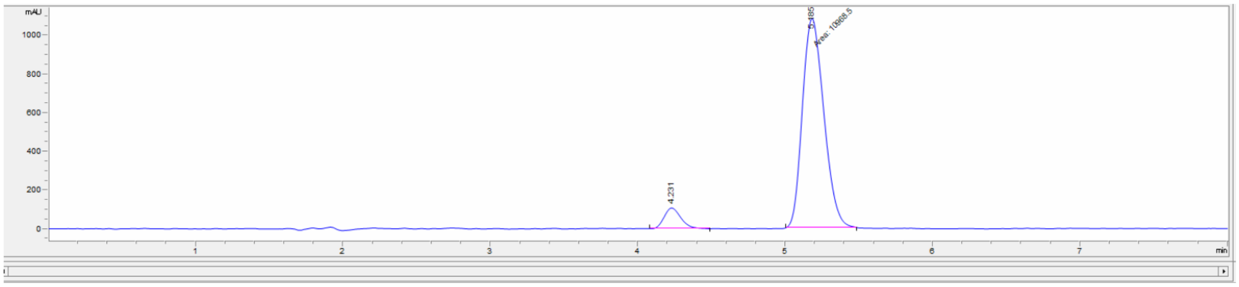

#	Time	Type	Area	Height	Width	Area%	Symmetry
1	4.231	BV	849.5	107.2	0.1225	7.188	0.757
2	5.185	MM	10968.5	1082.9	0.1688	92.812	0.792

Compound 3A (CDCl$_3$, ^{1}H NMR: 400 MHz, ^{13}C{^{1}H} NMR: 101 MHz)

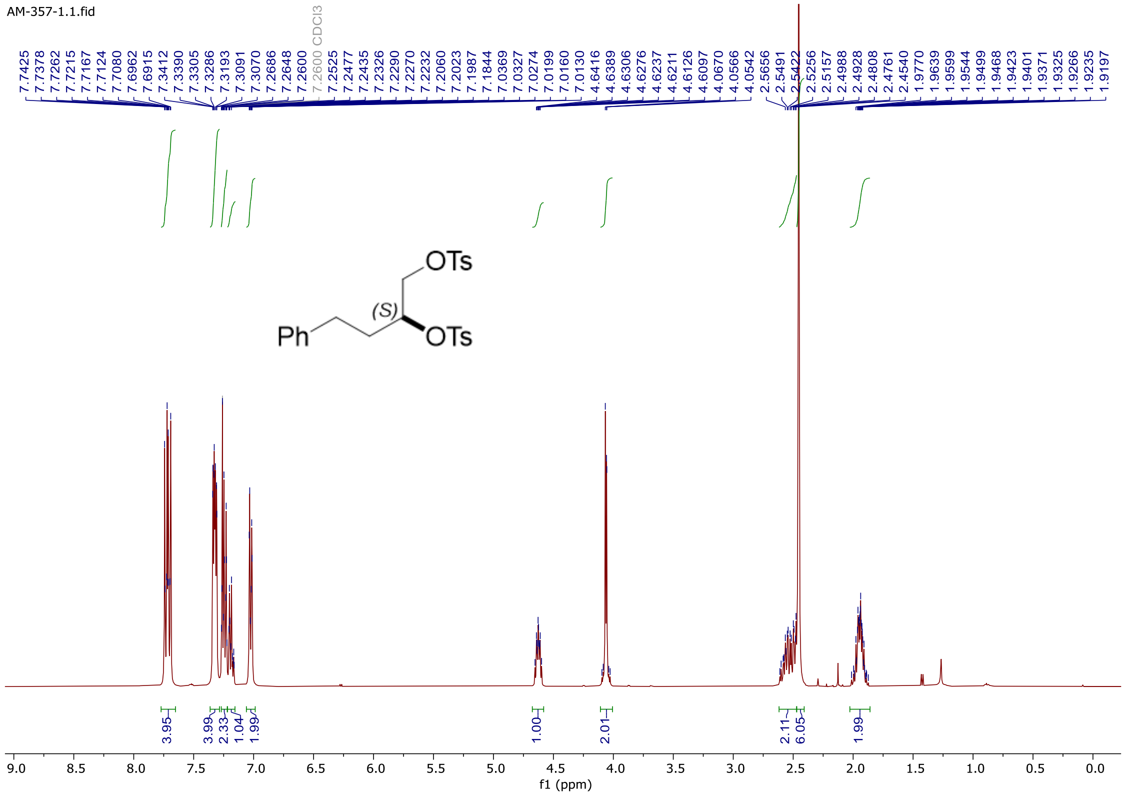

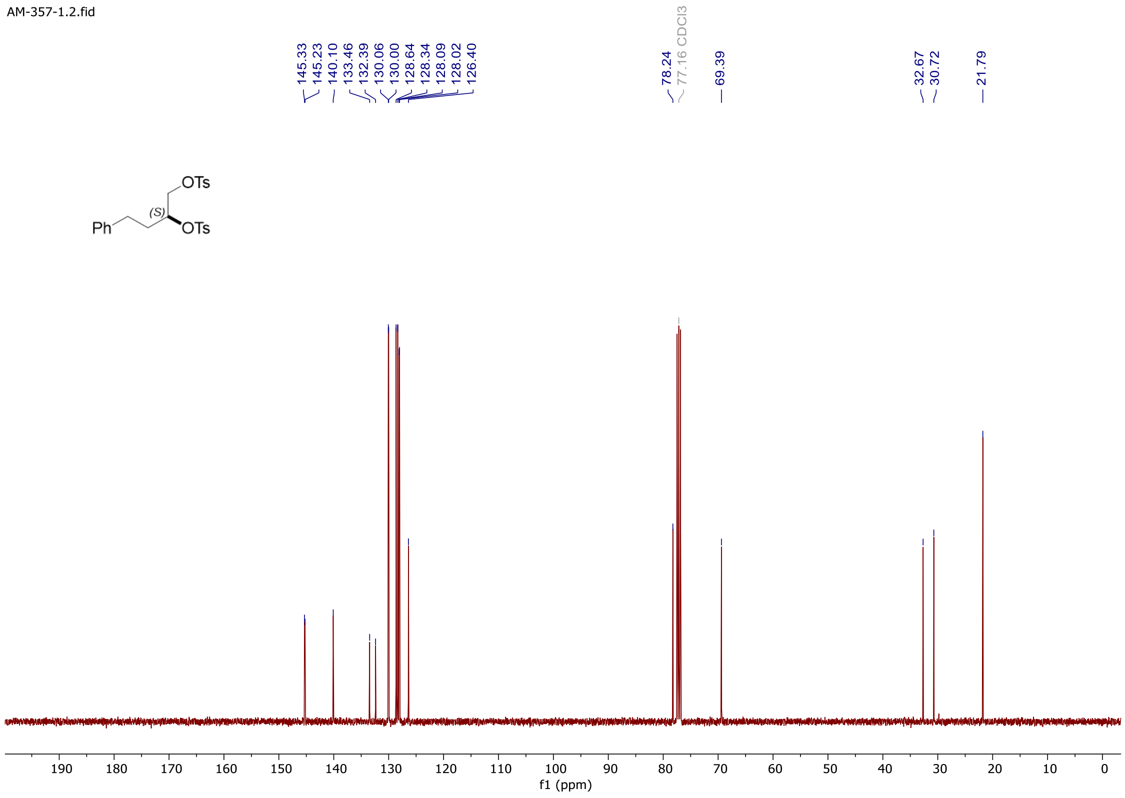

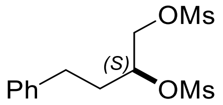

(*S*)-4-phenylbutane-1,2-diyl dimethanesulfonate

Compound 3B: Synthesized using the General Procedure on a 0.2 mmol scale; Purified using a gradient of 2% ethyl acetate in hexane to 35% ethyl acetate in hexane on silica gel; Predominant enantiomer depicted; (colorless oil, 35.0 mg, 0.108 mmol, 54% yield, 79% ee).

^{1}H NMR (400 MHz, $CDCl_3$) δ 7.34 – 7.26 (m, 2H), 7.25 – 7.18 (m, 3H), 4.91 (dddd, *J* = 7.9, 5.9, 5.0, 3.0 Hz, 1H), 4.40 (dd, *J* = 11.6, 3.0 Hz, 1H), 4.28 (dd, *J* = 11.6, 6.0 Hz, 1H), 3.08 (s, 3H), 3.06 (s, 3H), 2.87 – 2.70 (m, 2H), 2.20 – 1.97 (m, 2H).

^{13}C{^{1}H} NMR (101 MHz, $CDCl_3$) δ 140.0, 128.8, 128.5, 126.6, 78.5, 69.6, 38.9, 37.8, 32.9, 31.1.

IR υ 3054, 2988, 2305, 1421, 1363, 1266, 1177, 973, 918, 895, 740, 706, 528 cm^{-1}.

HRMS (ESI) m/z = $[M + Na]^+$ Calcd $C_{12}H_{18}O_6S_2Na^+$ 345.0443. Found 345.0419 (2.4 mmu error).

Specific rotation: $[\alpha]_D^{23}$ = +2.7 (c = 1.6 g/100 mL, $CHCl_3$, 79% ee).

Stereochemistry assigned by analogy to an authentic sample of 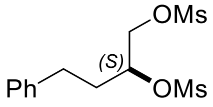

.

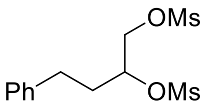

Racemic Sample: HPLC (IC-3, Isopropanol/hexanes = 20/80, flow rate = 1 mL/min, I = 254 nm), t_R = 43.5 min, 54.0 min.

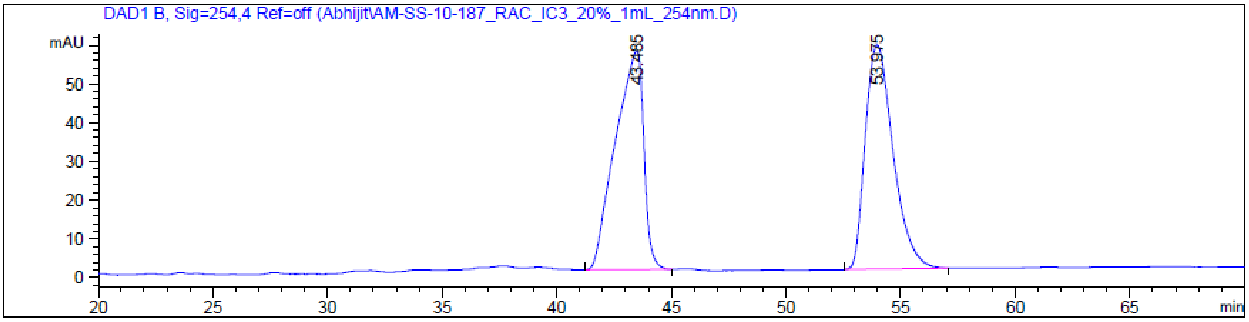

Peak #	RetTime [min]	Type	Width [min]	Area [mAU*s]	Height [mAU]	Area %
1	43.485	BB	1.1768	4846.65186	56.38557	49.7868
2	53.975	BB	1.2830	4888.16064	57.78041	50.2132

Scalemic Sample, +79% ee: HPLC (IC-3, Isopropanol/hexanes = 20/80, flow rate = 1 mL/min, I = 254 nm), t_R = 43.8 min, 54.0 min.

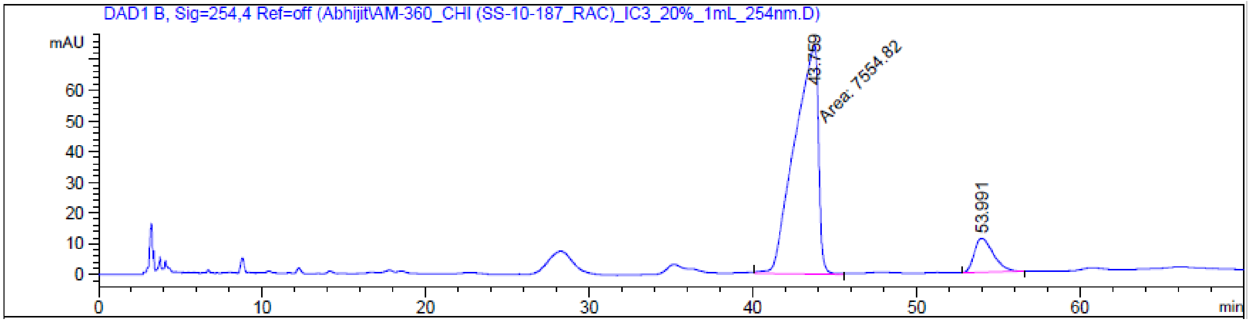

Peak #	RetTime [min]	Type	Width [min]	Area [mAU*s]	Height [mAU]	Area %
1	43.759	MM	1.6878	7554.81689	74.60140	89.2924
2	53.991	BB	0.9790	905.94543	11.13725	10.7076

Compound 3B ($CDCl_3$, ^1H NMR: 400 MHz, $^{13}C\{^1H\}$ NMR: 101 MHz)

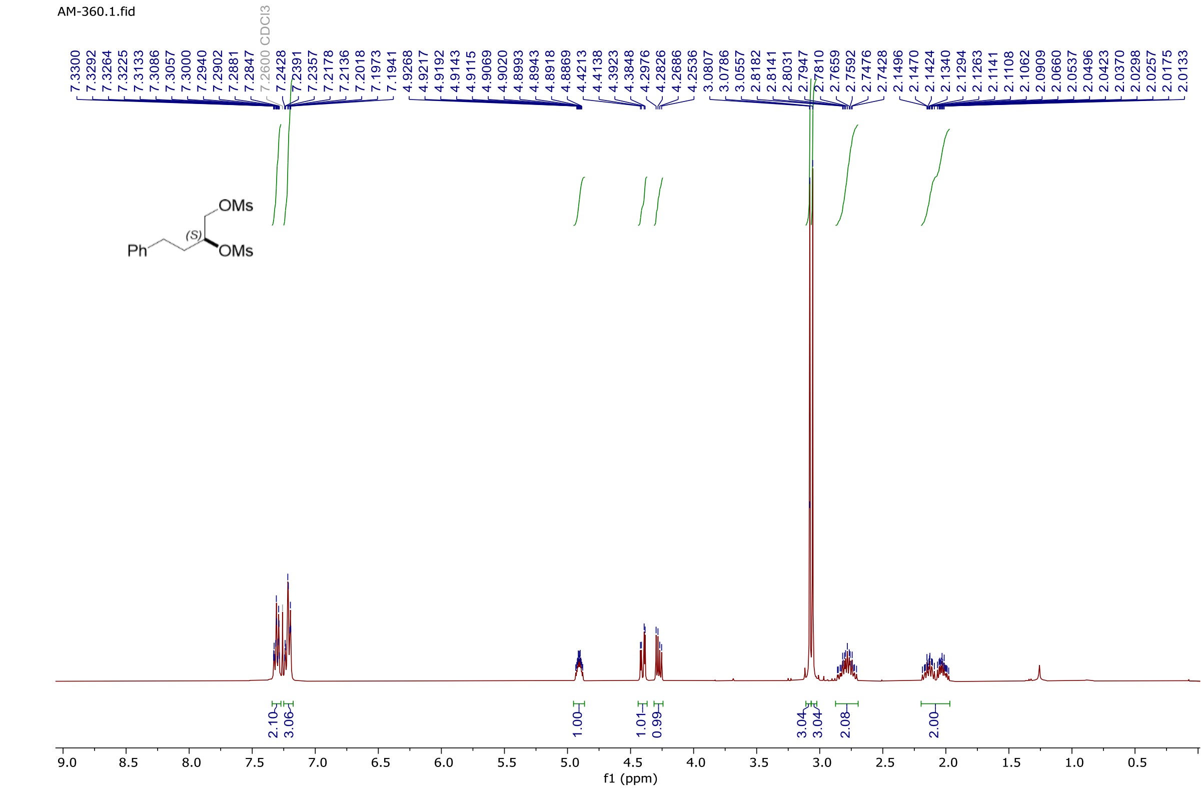

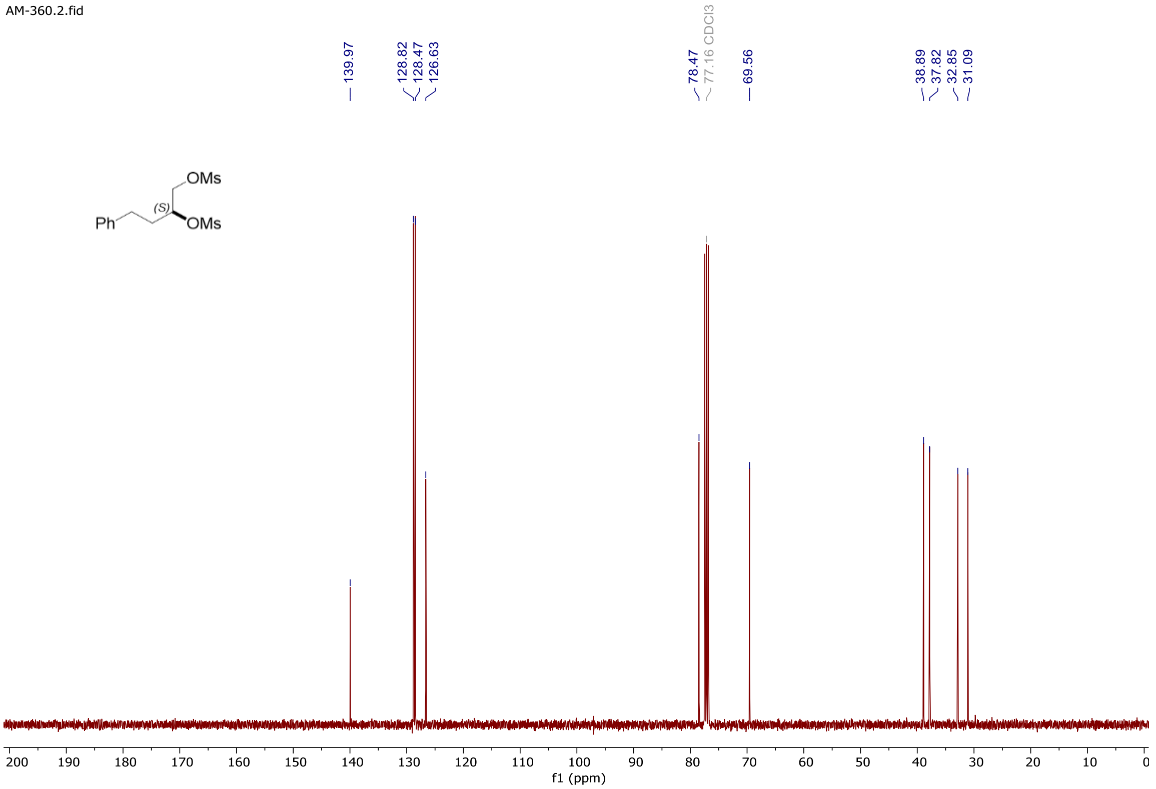

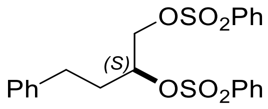

(*S*)-4-phenylbutane-1,2-diyl dibenzenesulfonate

Compound 3C: Synthesized using the General Procedure on a 0.2 mmol scale; Purified using a gradient of 2% ethyl acetate in hexane to 20% ethyl acetate in hexane on silica gel; Predominant enantiomer depicted; (colorless oil, 32.0 mg, 0.072 mmol, 36% yield, 89% ee).

^{1}H NMR (400 MHz, $CDCl_3$) δ 7.90 – 7.77 (m, 4H), 7.71 – 7.63 (m, 2H), 7.59 – 7.49 (m, 4H), 7.28 – 7.22 (m, 2H), 7.22 – 7.16 (m, 1H), 7.08 – 6.97 (m, 2H), 4.75 – 4.56 (m, 1H), 4.15 – 4.04 (m, 2H), 2.64 – 2.43 (m, 2H), 2.07 – 1.88 (m, 2H).

^{13}C{^{1}H} NMR (101 MHz, $CDCl_3$) δ 140.0, 136.5, 135.4, 134.2, 134.1, 129.5, 129.4, 128.7, 128.3, 128.1, 128.0, 126.5, 78.5, 69.6, 32.7, 30.8.

IR υ 3054, 2988, 2305, 1449, 1421, 1369, 1266, 1188, 1096, 915, 895, 740, 706, 588 cm^{-1}.

HRMS (ESI) m/z = $[M + Na]^+$ Calcd $C_{22}H_{22}O_6S_2Na^+$ 469.0756. Found 469.0737 (4.1 ppm error).

Specific rotation: $[\alpha]_D^{22}$ = –3.4 (c = 1.6 g/100 mL, $CHCl_3$, 89% ee).

Stereochemistry assigned by analogy to an authentic sample of 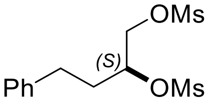.

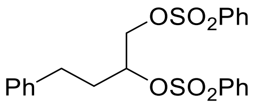

Racemic Sample: HPLC (IC-3, Isopropanol/hexanes = 05/95, flow rate = 0.5 mL/min, I = 254 nm), t_R = 83.9 min, 89.3 min.

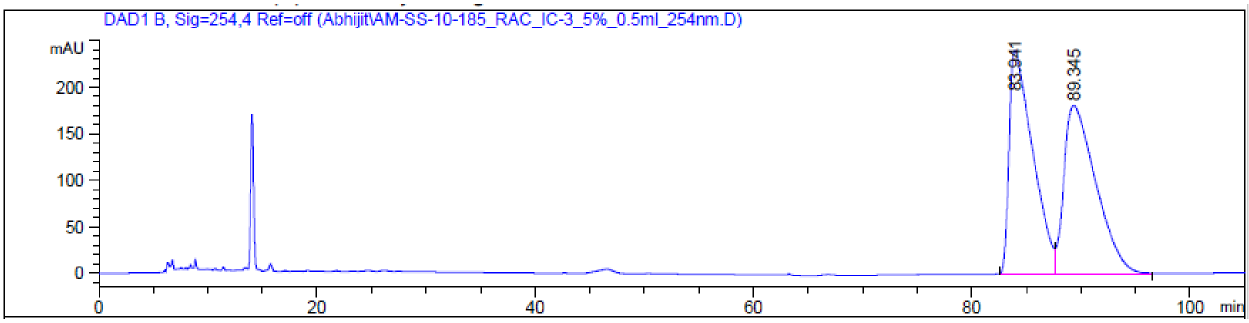

Peak #	RetTime [min]	Type	Width [min]	Area [mAU*s]	Height [mAU]	Area %
1	83.941	HH	2.0092	3.59452e4	240.56348	49.1687
2	89.345	HH	2.7726	3.71606e4	181.80675	50.8313

Scalemic Sample, +89% ee: HPLC (IC-3, Isopropanol/hexanes = 05/95, flow rate = 0.5 mL/min, I = 254 nm), t_R = 89.4 min, 98.9 min.

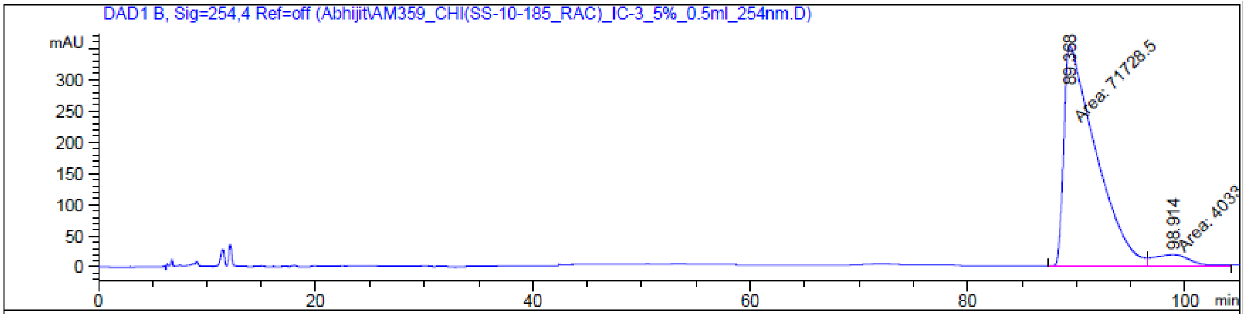

Peak #	RetTime [min]	Type	Width [min]	Area [mAU*s]	Height [mAU]	Area %
1	89.368	MF	3.3809	7.17285e4	353.59479	94.6759
2	98.914	FM	3.7479	4033.66846	17.93762	5.3241

Compound 3C ($CDCl_3$, ^1H NMR: 400 MHz, $^{13}C\{^1H\}$ NMR: 101 MHz)

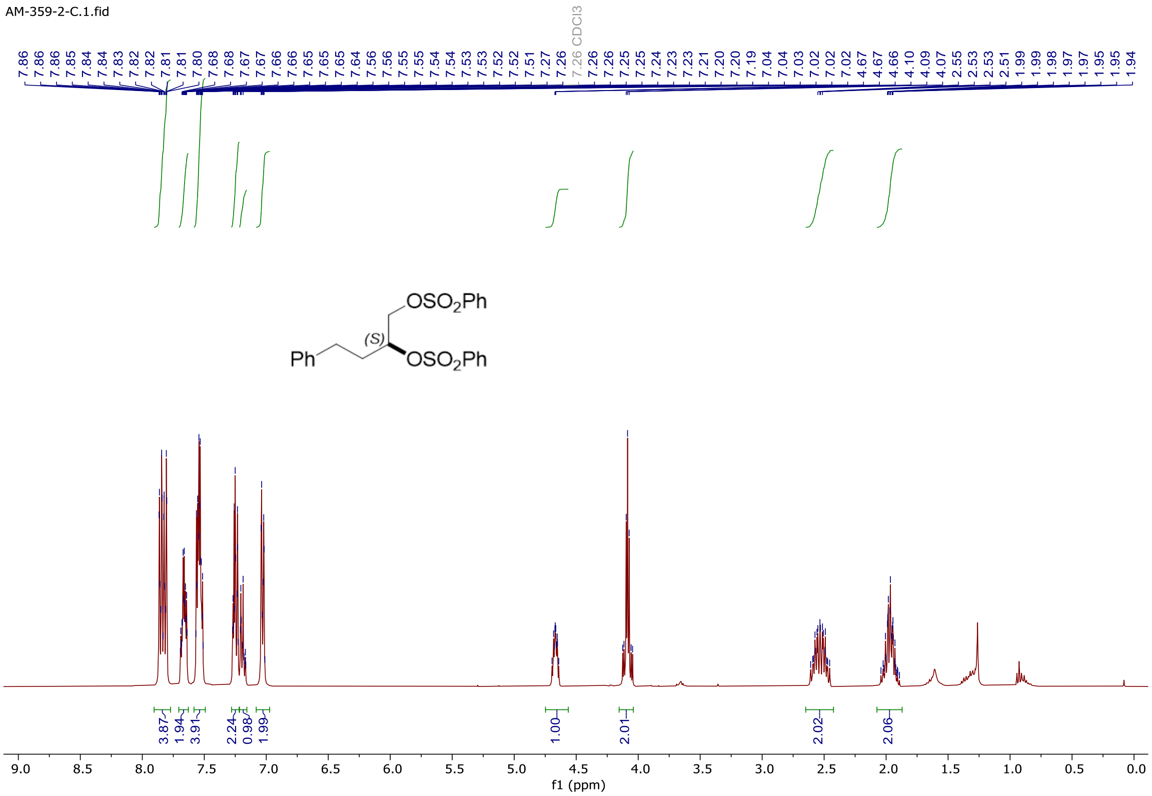

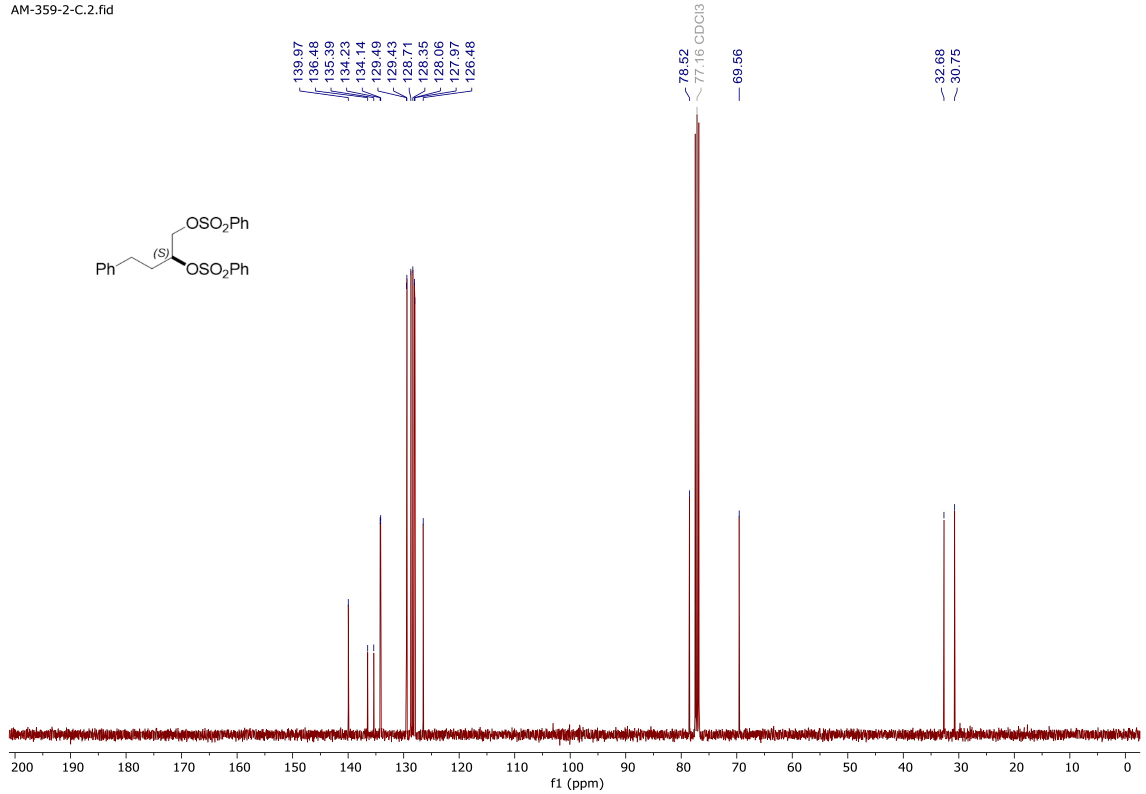

Substrate-3D (CDCl$_3$, ^{1}H NMR: 400 MHz, ^{13}C{^{1}H} NMR: 101 MHz)

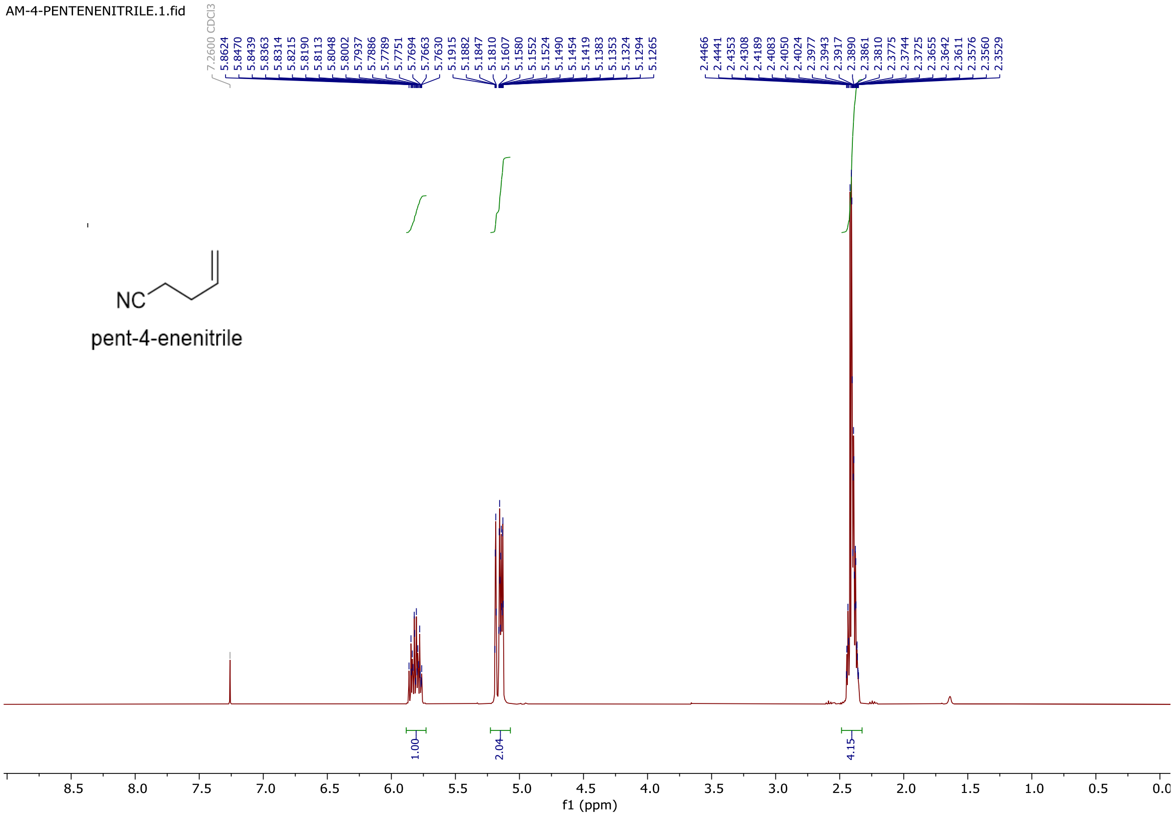

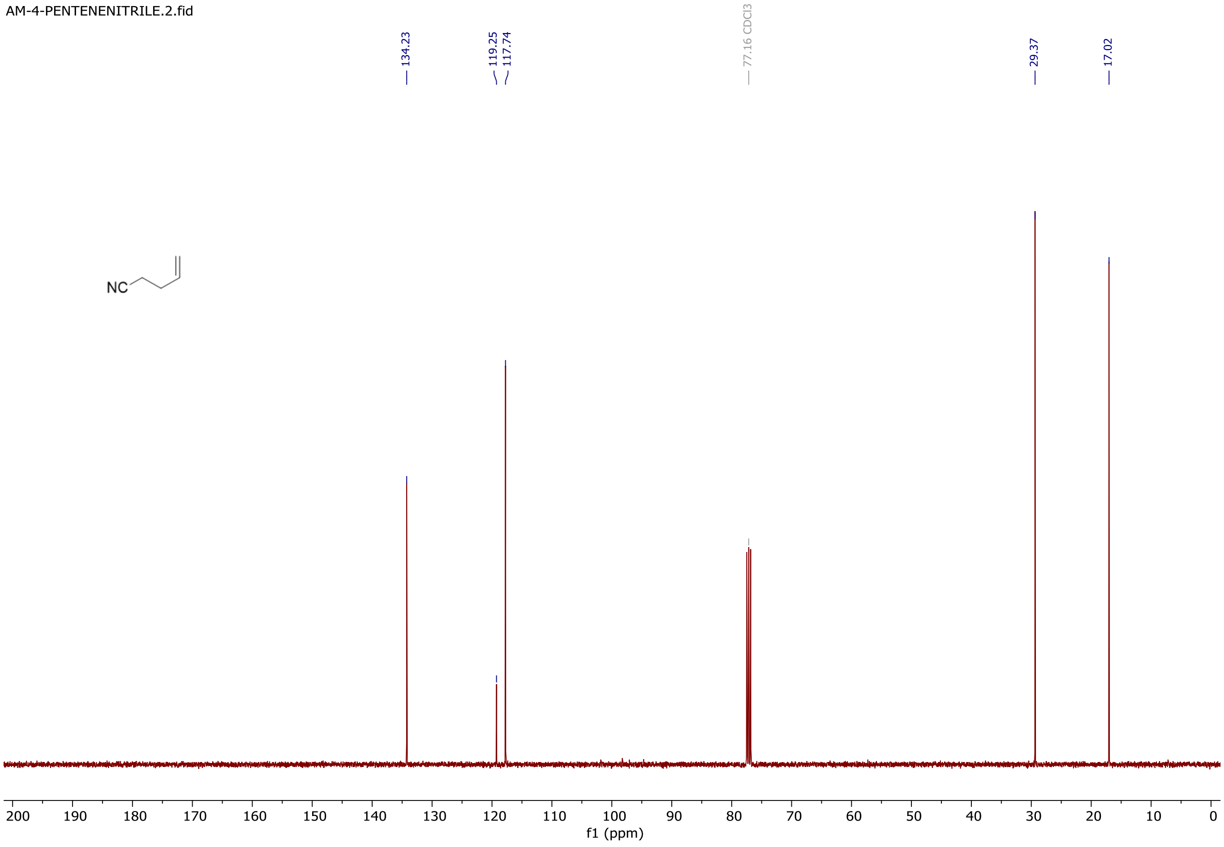

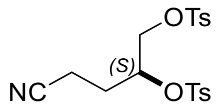

(*S*)-4-cyanobutane-1,2-diyl bis(4-methylbenzenesulfonate)

Compound 3D: Synthesized using the General Procedure on a 0.2 mmol scale; Purified using a gradient of 2% ethyl acetate in hexane to 25% ethyl acetate in hexane on silica gel; Predominant enantiomer depicted; (colorless oil, 27.5 mg, 0.065 mmol, 32% yield, 67% ee).

^{1}H NMR (400 MHz, $CDCl_3$) δ 7.79 – 7.67 (m, 4H), 7.39 – 7.32 (m, 4H), 4.67 (dq, *J* = 7.7, 4.6 Hz, 1H), 4.10 – 4.01 (m, 2H), 2.47 (overlapping s, 6H), 2.43 – 2.23 (m, 2H), 2.06 – 1.92 (m, 2H).

^{13}C{^{1}H} NMR (101 MHz, $CDCl_3$) δ 145.9, 145.7, 132.6, 132.1, 130.3, 130.2, 128.12, 128.09, 118.2, 76.1, 68.8, 27.4, 21.9, 21.8, 13.3.

IR ν 3054, 2988, 2305, 1599, 1424, 1372, 1266, 1191, 1180, 1096, 895, 815, 749, 706, 554 cm^{-1}.

HRMS (ESI) m/z = $[M + Na]^+$ Calcd $C_{19}H_{21}NO_6S_2Na^+$ 446.0708. Found 446.0688 (4.5 ppm error).

Specific rotation: $[\alpha]_D^{24}$ = –21.2 (c = 1.2 g/100 mL, $CHCl_3$, 67% ee).

Stereochemistry assigned by analogy to an authentic sample of 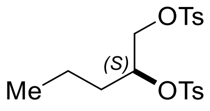 **.**

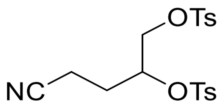

Racemic Sample: HPLC (IC-3, Isopropanol/hexanes = 50/50, flow rate = 1 mL/min, I = 254 nm), t_R = 61.7 min, 76.8 min.

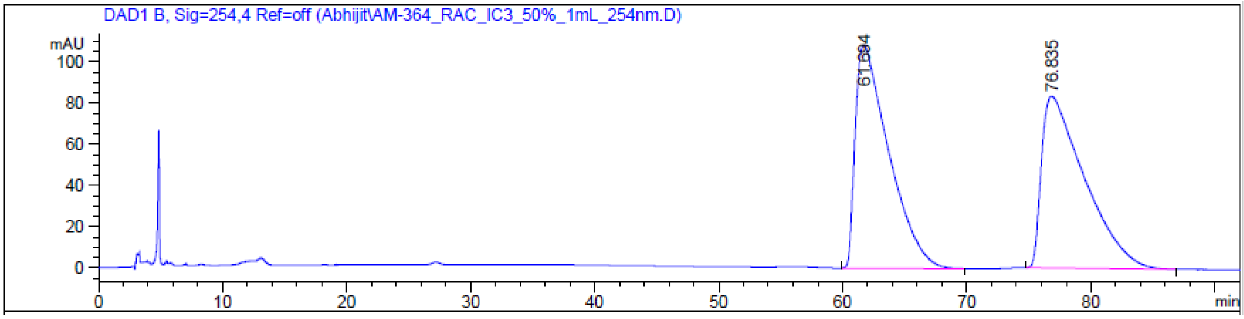

Peak #	RetTime [min]	Type	Width [min]	Area [mAU*s]	Height [mAU]	Area %
1	61.694	BB	2.4836	2.02729e4	108.11475	50.1859
2	76.835	BB	3.1214	2.01227e4	83.38701	49.8141

Scalemic Sample, -67% ee: HPLC (IC-3, Isopropanol/hexanes = 50/50, flow rate = 1 mL/min, I = 254 nm), t_R = 69.0 min, 81.3 min.

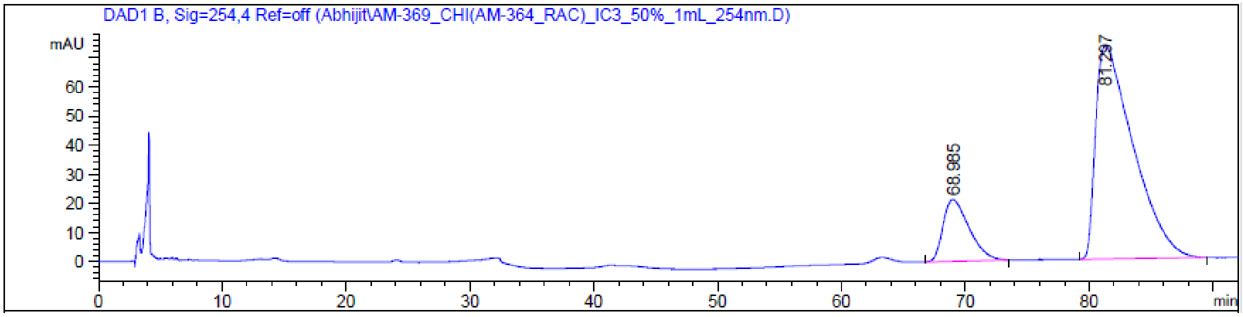

Peak #	RetTime [min]	Type	Width [min]	Area [mAU*s]	Height [mAU]	Area %
1	68.985	BB	1.7208	3097.71411	21.17368	16.6634
2	81.297	BB	2.4799	1.54922e4	73.35654	83.3366

Compound 3D ($CDCl_3$, ^1H NMR: 400 MHz, $^{13}C\{^1H\}$ NMR: 101 MHz)

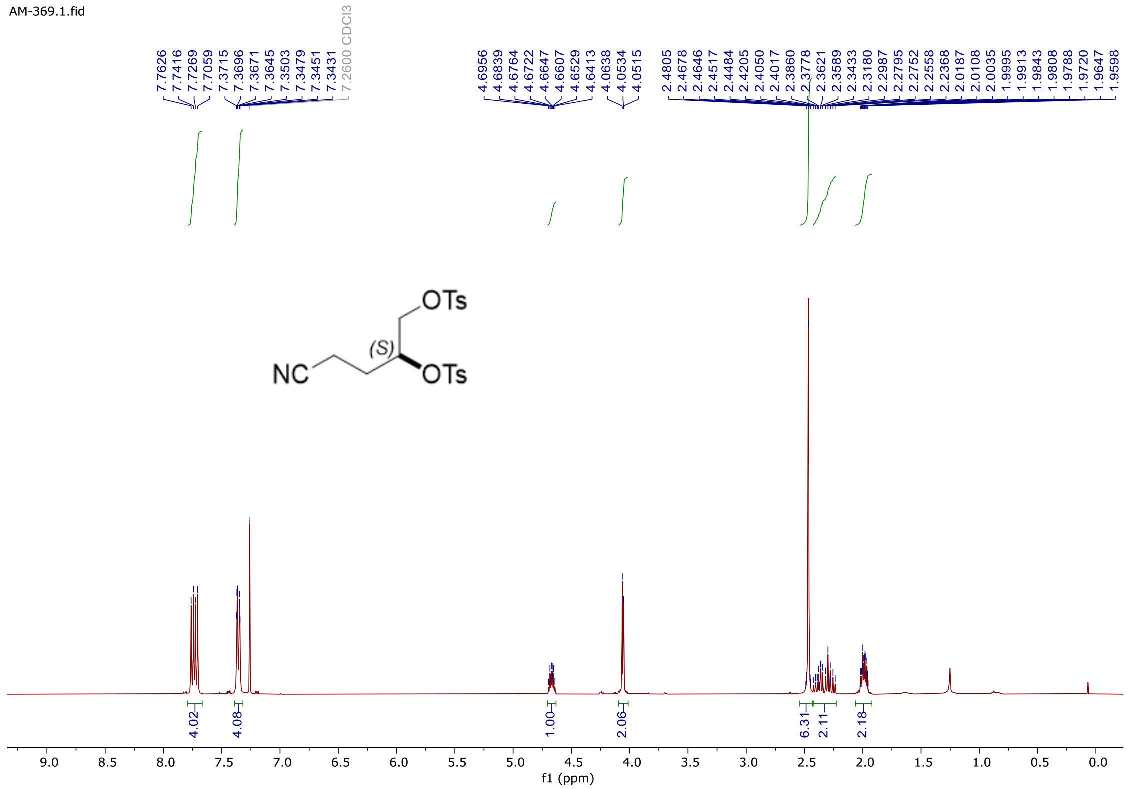

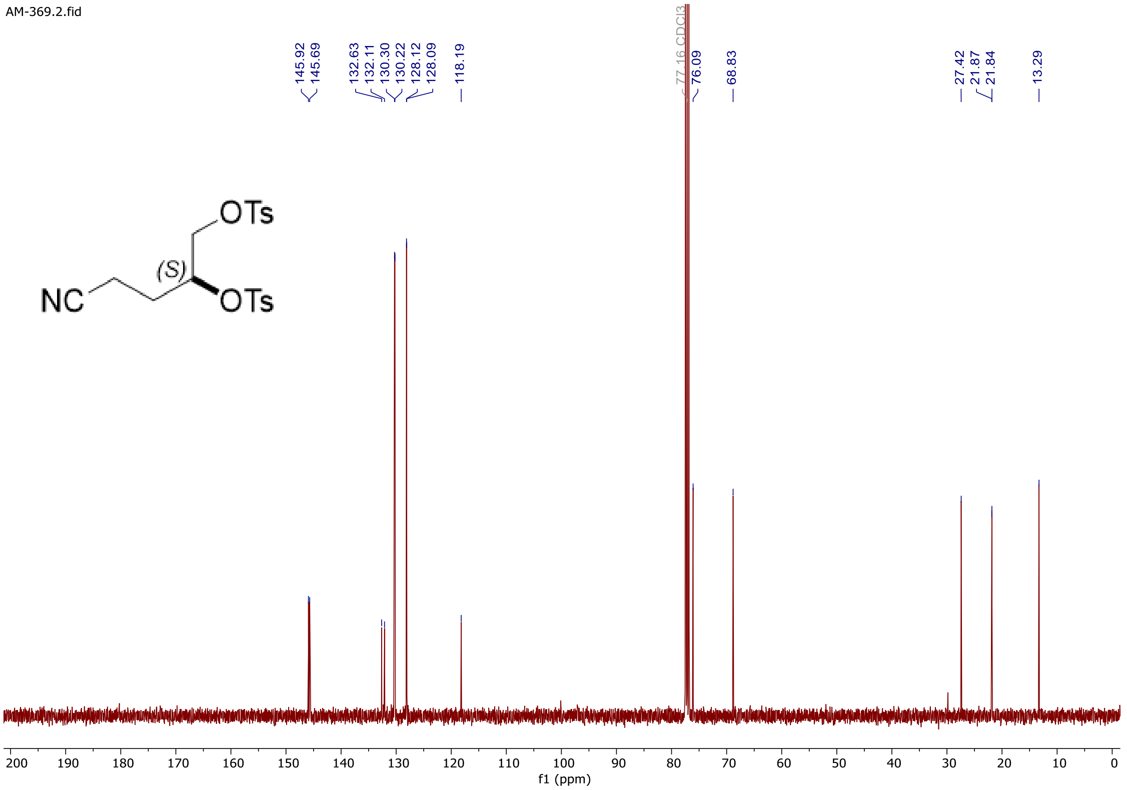

Substrate-3E ($CDCl_3$, ^1H NMR: 400 MHz, $^{13}C\{^1H\}$ NMR: 101 MHz)

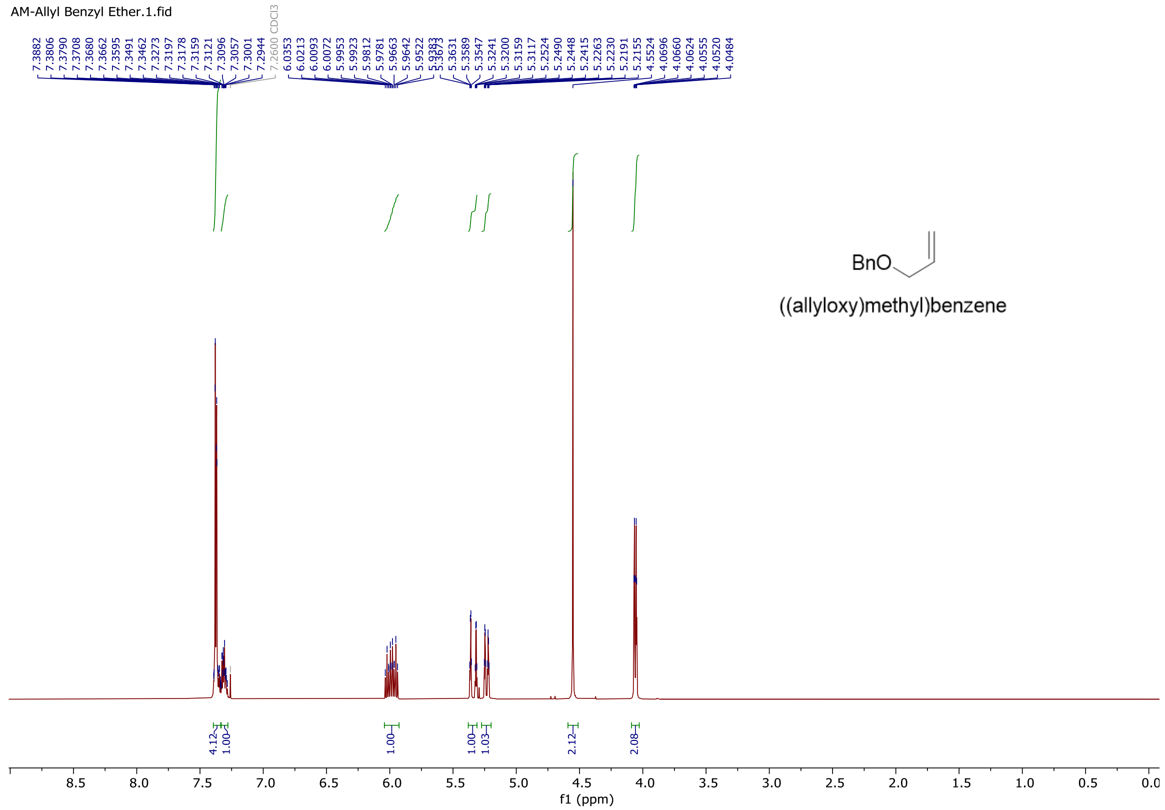

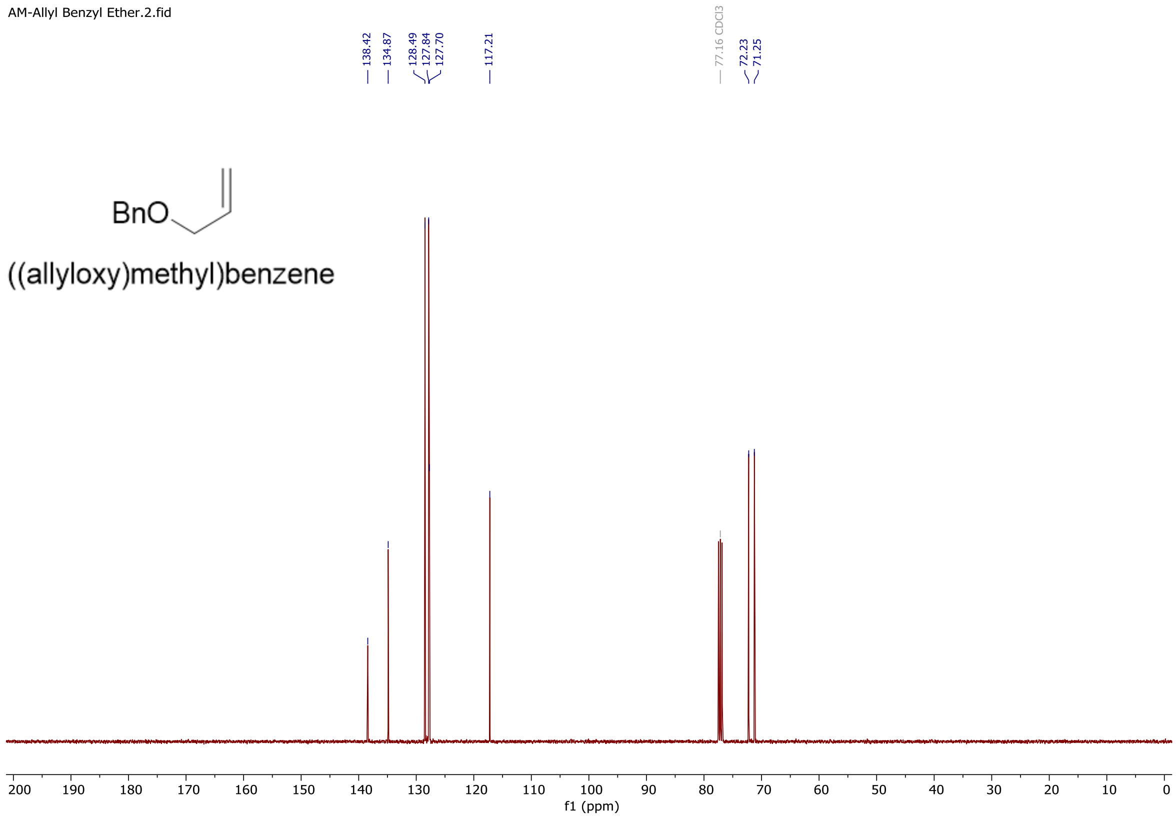

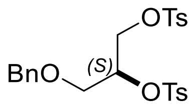

(S)-3-(benzyloxy)propane-1,2-diyl bis(4-methylbenzenesulfonate)

Compound 3E: Synthesized using the General Procedure on a 0.2 mmol scale; Purified using a gradient of 2% ethyl acetate in hexane to 8% ethyl acetate in hexane on silica gel; Predominant enantiomer depicted; (colorless oil, 49.7 mg, 0.101 mmol, 51% yield, 55% ee).

^{1}H NMR (400 MHz, $CDCl_3$) δ 7.76 – 7.65 (m, 4H), 7.37 – 7.26 (m, 7H), 7.22 – 7.14 (m, 2H), 4.69 (qd, *J* = 5.2, 4.2 Hz, 1H), 4.45 – 4.35 (m, 2H), 4.21 – 4.10 (m, 2H), 3.65 – 3.56 (m, 2H), 2.44 (s, 3H), 2.43 (s, 3H).

^{13}C{^{1}H} NMR (101 MHz, $CDCl_3$) δ 145.3, 145.2, 137.3, 133.2, 132.4, 130.0, 129.9, 128.5, 128.1, 128.0, 127.7, 76.9, 73.6, 67.8, 67.6, 21.8.

IR ν 3054, 2988, 2305, 1599, 1420, 1369, 1266, 1191, 1180, 933, 895, 815, 740, 706, 554 cm^{-1}.

HRMS (ESI) m/z = $[M + Na]^+$ Calcd $C_{24}H_{26}O_7S_2Na^+$ 513.1018. Found 513.1007 (2.1 ppm error).

Specific rotation: $[\alpha]_D^{23}$ = +2.7 (c = 2.45 g/100 mL, $CHCl_3$, 55% ee)

Stereochemistry assigned by analogy to an authentic sample of 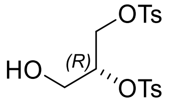 **.**

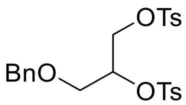

Racemic Sample: SFC (ColumnTek Enantiocel IDH-5, MeOH with 0.1% DEA/CO_2 = 35/65, flow rate = 2 mL/min, I = 220 nm), t_R = 4.3 min, 6.8 min.

AM-371-rac

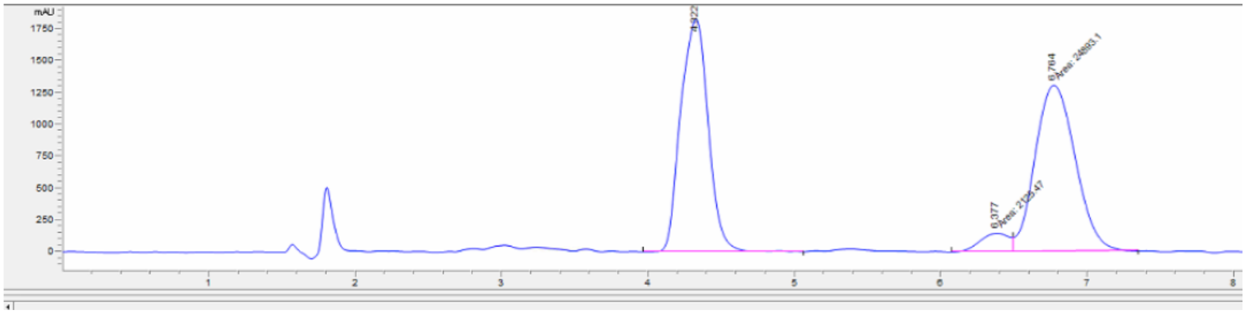

Index	Time (min)	Area (%)
Peak-1	4.322	46.980
Impurity	6.377	4.171
Peak-2	6.764	48.849
Total		100.00

Scalemic Sample, -55% ee: SFC (ColumnTek Enantiocel IDH-5, MeOH with 0.1% DEA/CO_2 = 35/65, flow rate = 2 mL/min, I = 220 nm), t_R = 4.3 min, 6.8 min.

AM-372-chi

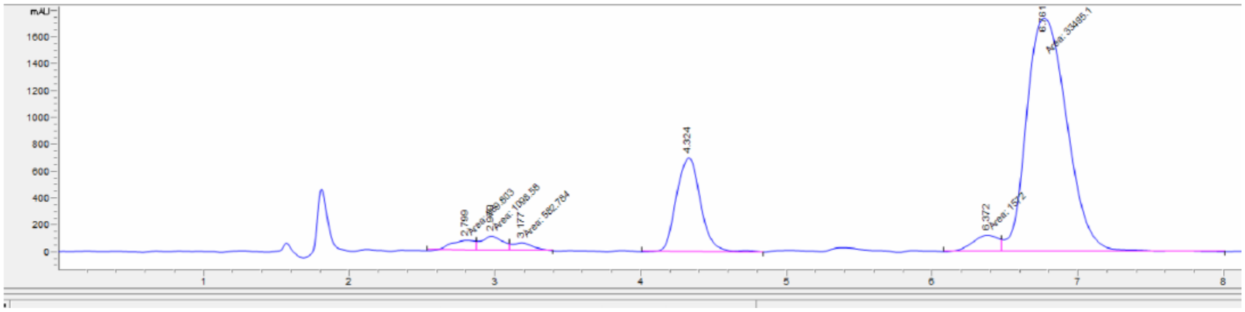

Index	Time (min)	Area (%)
Impurity	2.799	1.901
Impurity	2.97	2.401
Impurity	3.177	1.274
Peak-1	4.324	17.786
Impurity	6.372	3.436
Peak-2	6.761	73.203
Total		100.00

Compound 3E (CDCl₃, ^{1}H NMR: 400 MHz, ^{13}C{^{1}H} NMR: 101 MHz)

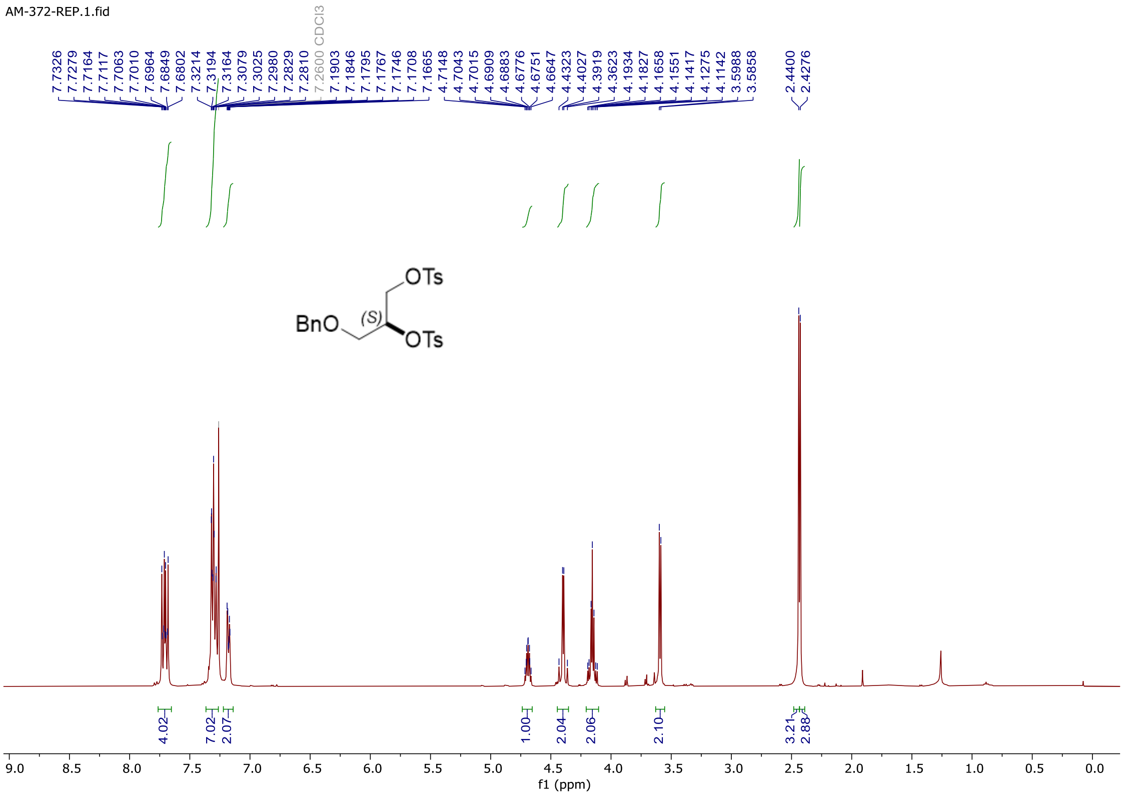

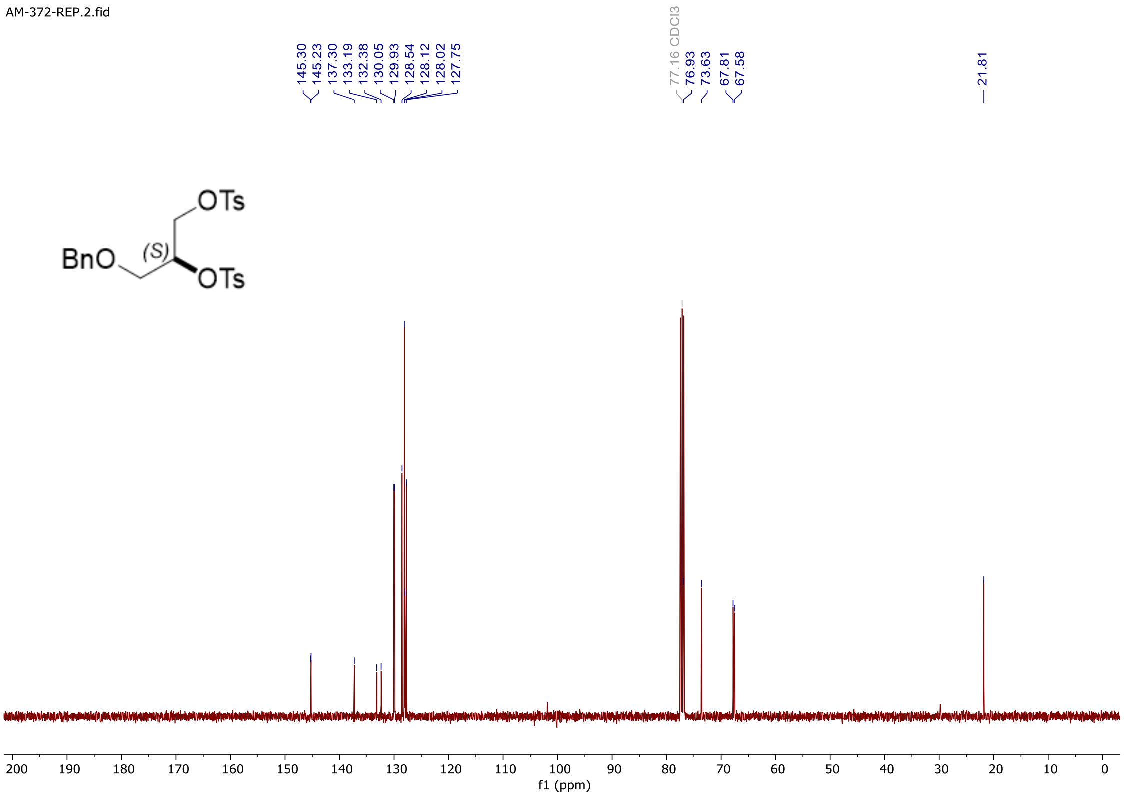

Substrate-3F ($CDCl_3$, ^1H NMR: 400 MHz, $^{13}C\{^1H\}$ NMR: 101 MHz)

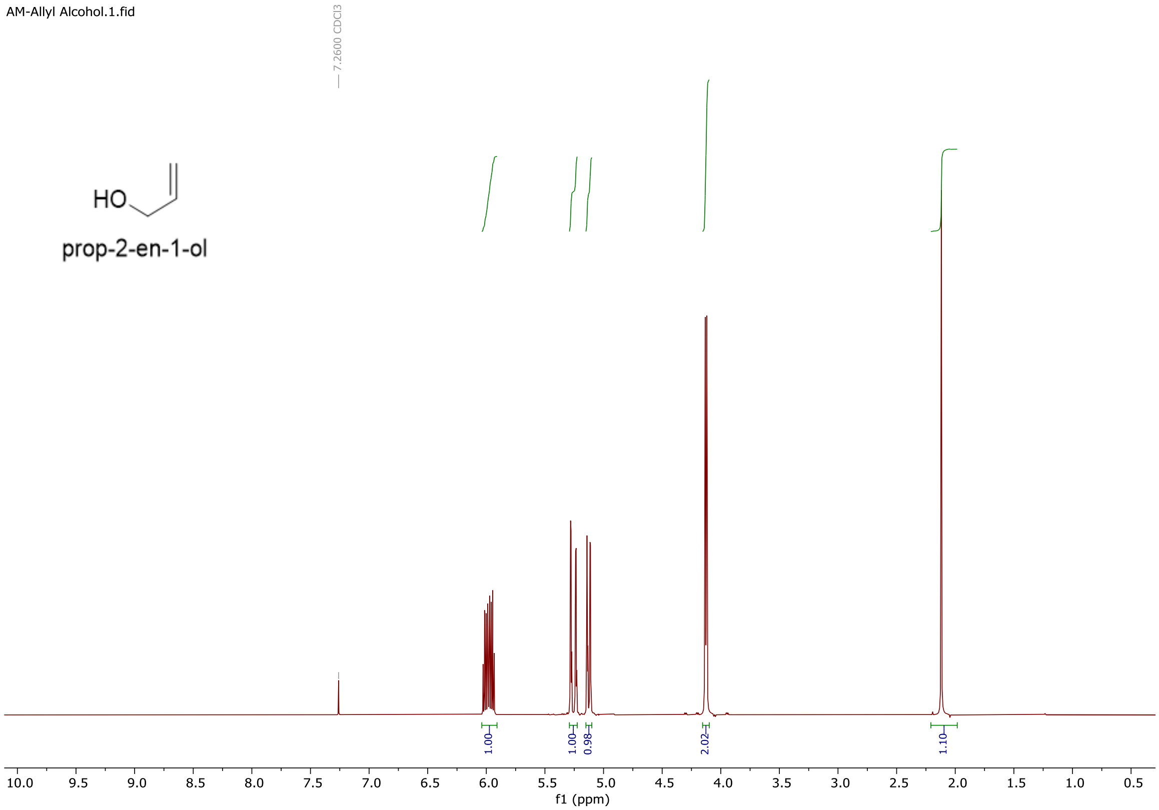

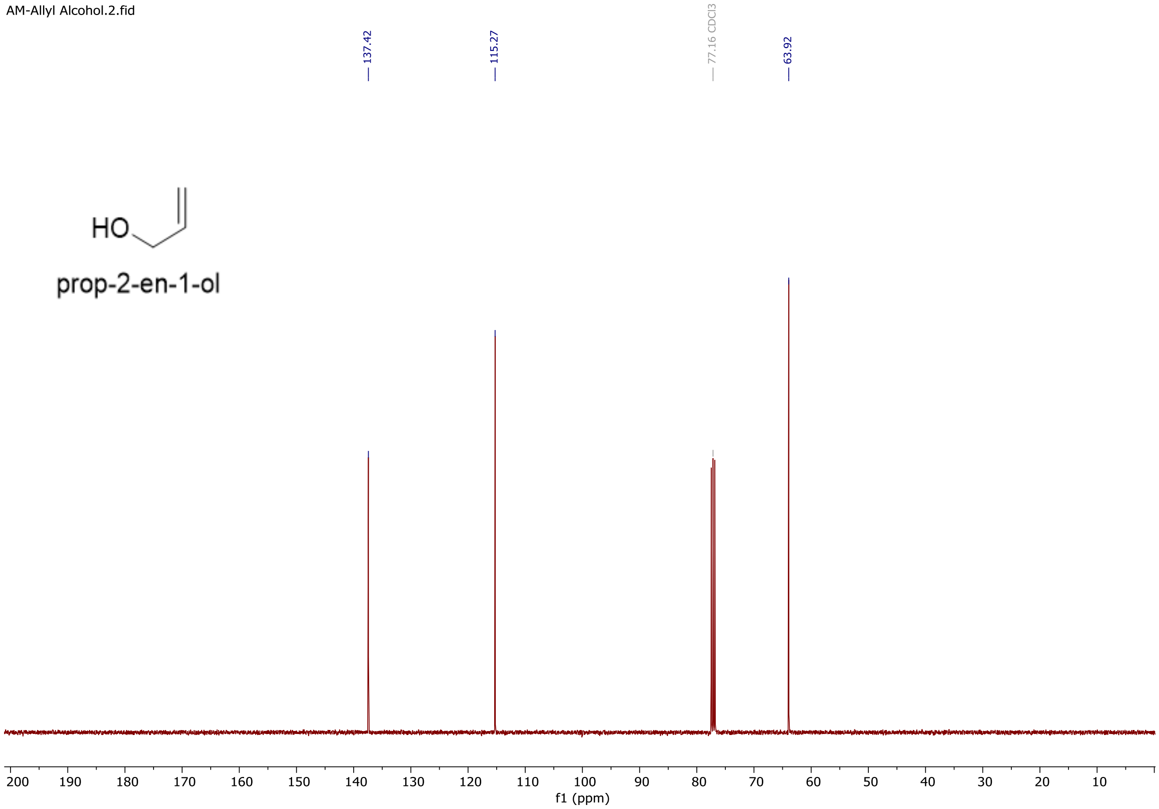

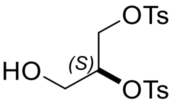

(*S*)-3-hydroxypropane-1,2-diyl bis(4-methylbenzenesulfonate)

Compound 3F: Synthesized using the General Procedure on a 0.2 mmol scale; Purified using a gradient of 2% ethyl acetate in hexane to 25% ethyl acetate in hexane on silica gel; Predominant enantiomer depicted; (colorless oil, 23.2 mg, 0.058 mmol, 29% yield, 37% ee).

^{1}H NMR (400 MHz, $CDCl_3$) δ 7.79 – 7.74 (m, 2H), 7.72 – 7.68 (m, 2H), 7.37 – 7.31 (m, 4H), 4.63 (p, *J* = 4.9 Hz, 1H), 4.18 – 4.10 (m, 2H), 3.84 – 3.67 (m, 2H), 2.46 (overlapping s, 6H), 2.05 – 1.99 (m, 1H).

^{13}C{^{1}H} NMR (101 MHz, $CDCl_3$) δ 145.6, 145.5, 132.9, 132.3, 130.1, 128.1, 78.7, 67.0, 61.1, 21.9, 21.8.

IR ν 3547, 1358, 1174, 1096, 924 cm^{-1}.

HRMS (ESI) m/z = $[M + Na]^+$ Calcd $C_{17}H_{20}O_7S_2Na^+$ 423.0548. Found 423.0535 (3.1 ppm error).

Specific Rotation: $[\alpha]_D^{21}$ = −5.77 (c = 0.7 g/100 mL, $CHCl_3$, 37% ee).

Stereochemistry assigned by analogy to an authentic sample of 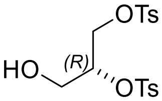 **.**

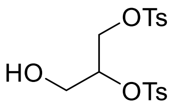

Racemic Sample: HPLC (IC-3, Isopropanol/hexanes = 20/80, flow rate = 1 mL/min, I = 254 nm), t_R = 65.7 min, 71.8 min.

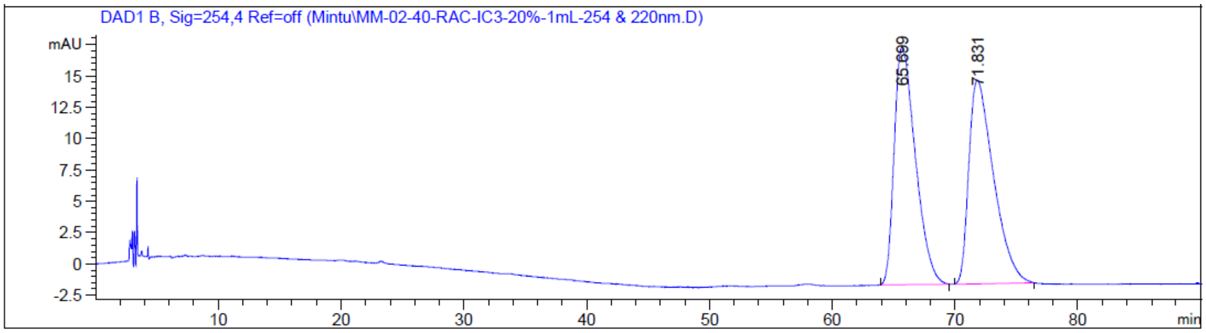

Peak #	RetTime [min]	Type	Width [min]	Area [mAU*s]	Height [mAU]	Area %
1	65.699	BB	1.4186	2279.39502	18.93259	50.0634
2	71.831	BB	1.6456	2273.61890	16.23780	49.9366

Scalemic Sample, -37% ee: HPLC (IC-3, Isopropanol/hexanes = 20/80, flow rate = 1 mL/min, I = 254 nm), t_R = 64.6 min, 69.6 min.

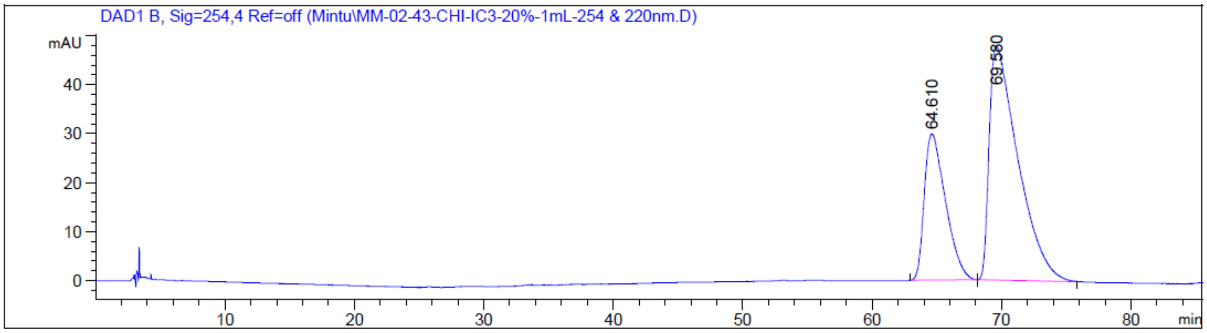

Peak #	RetTime [min]	Type	Width [min]	Area [mAU*s]	Height [mAU]	Area %
1	64.610	BB	1.5080	3402.68262	29.84715	31.6056
2	69.580	BB	1.9825	7363.39307	47.65899	68.3944

Compound 3F (CDCl₃, ¹H NMR: 400 MHz, ¹³C{¹H} NMR: 101 MHz)

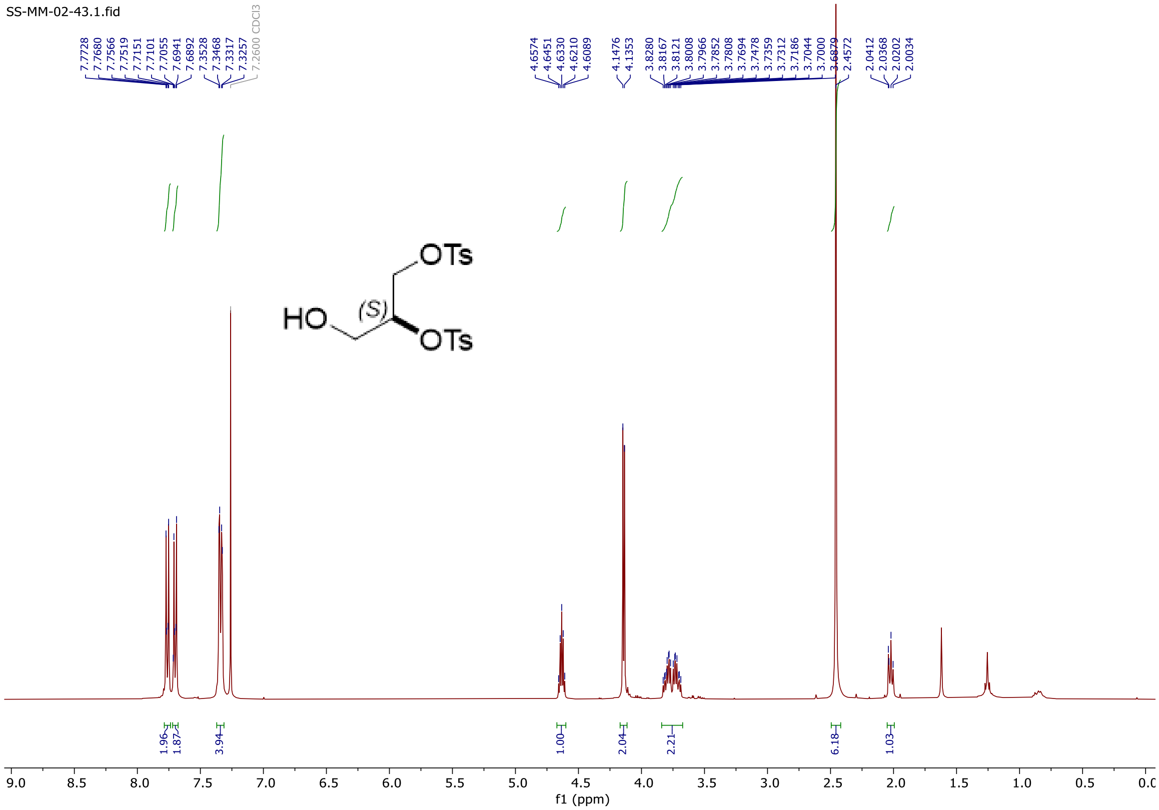

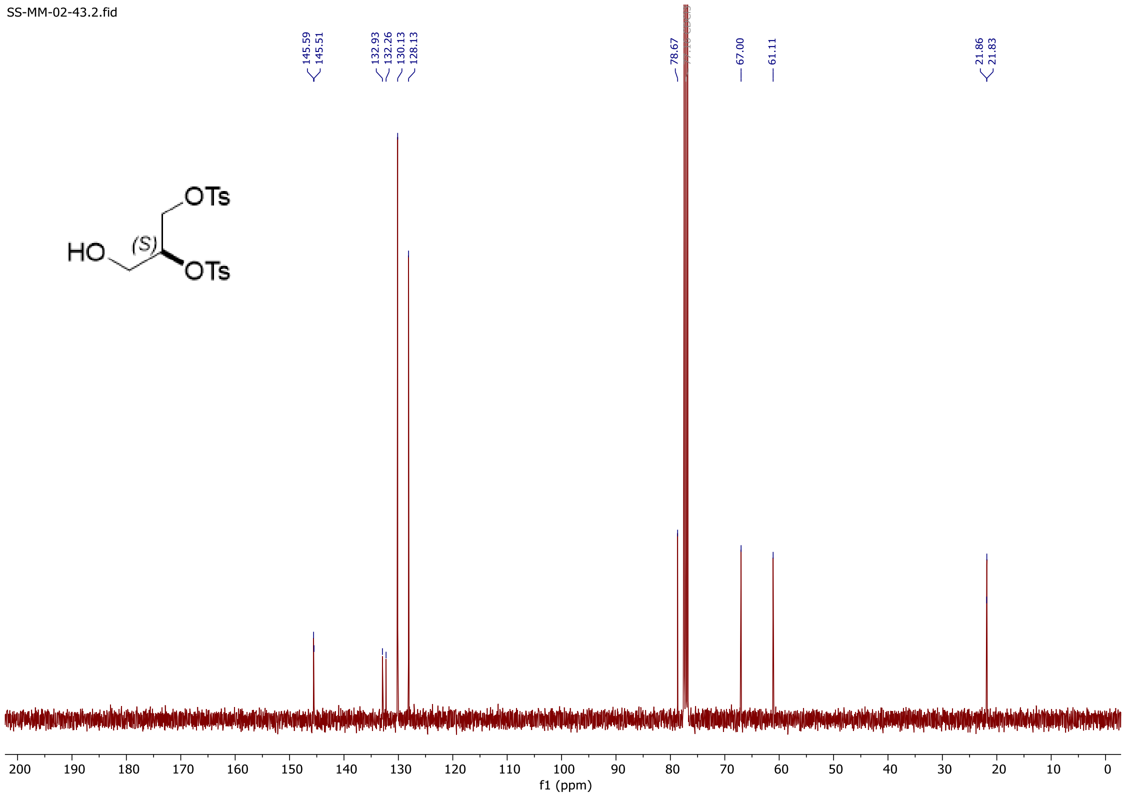

Substrate-3G-I ($CDCl_3$, ^1H NMR: 400 MHz, $^{13}C\{^1H\}$ NMR: 101 MHz)

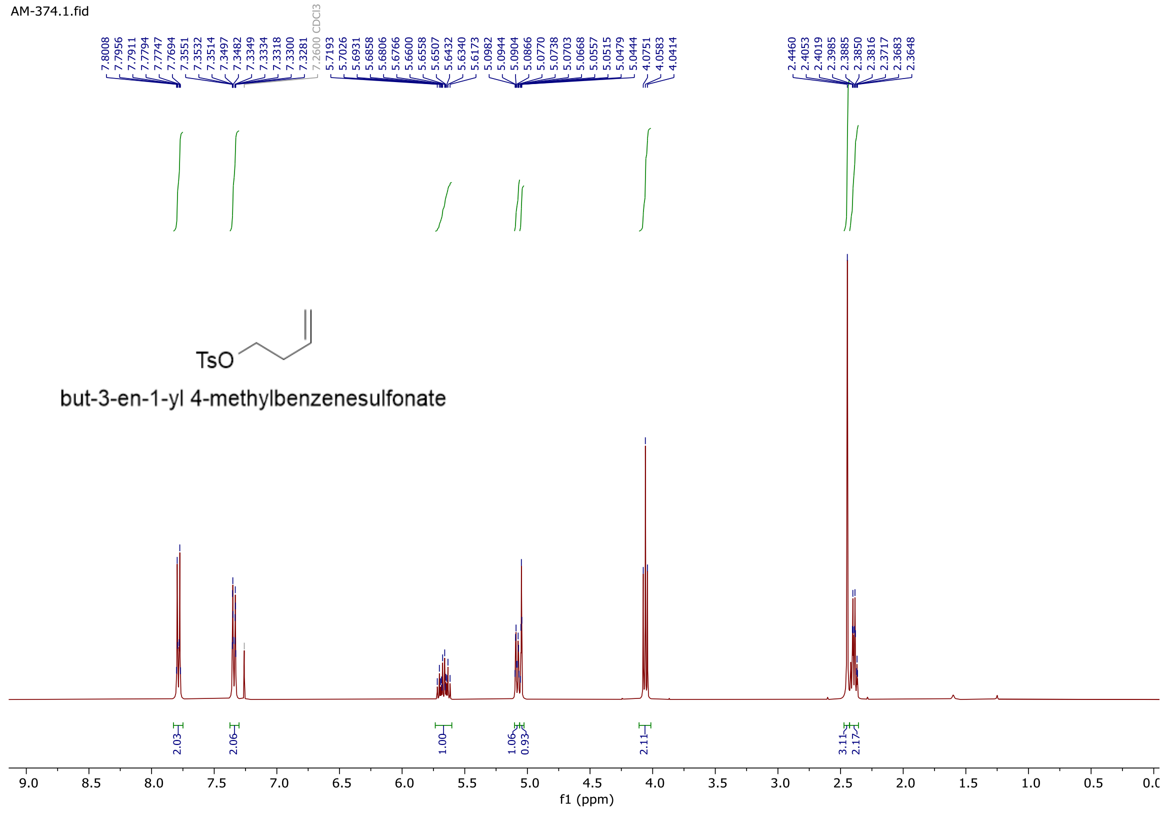

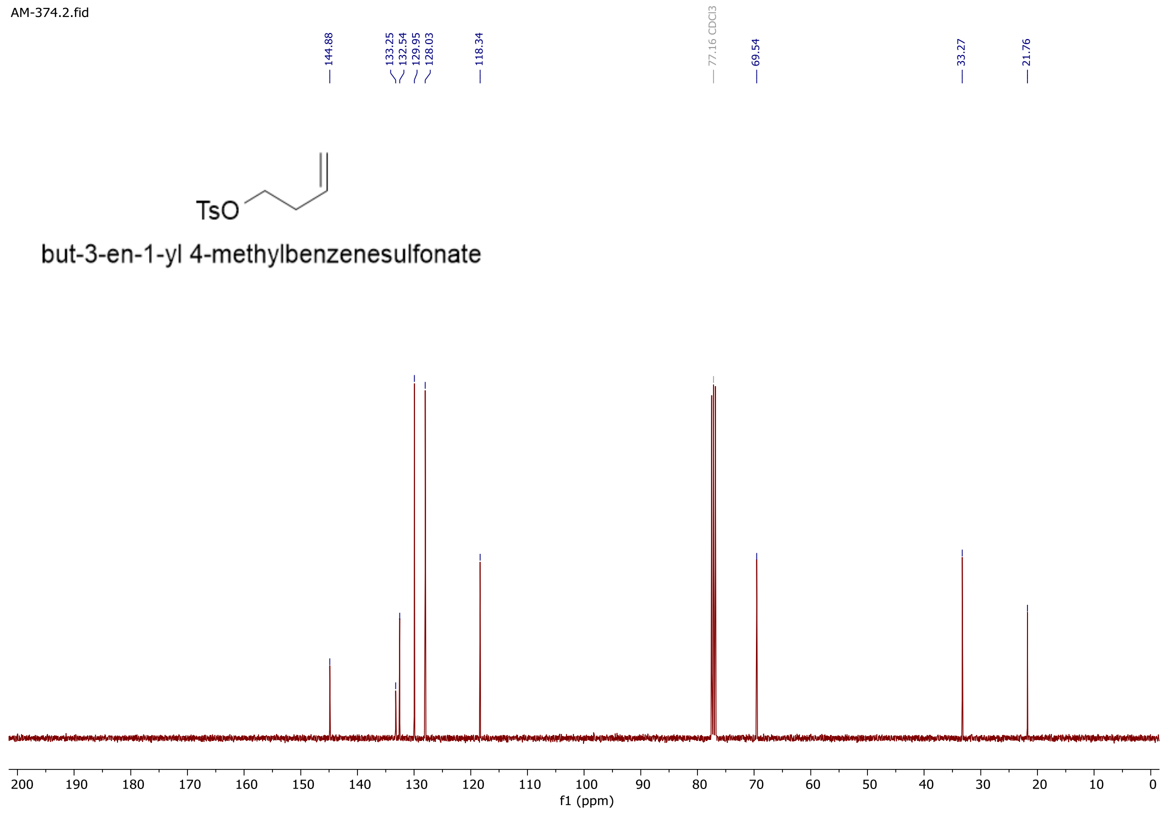

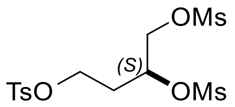

(*S*)-4-(tosyloxy)butane-1,2-diyl dimethanesulfonate

Compound 3G: Synthesized using the General Procedure on a 0.2 mmol scale; Purified using a gradient of 2% ethyl acetate in hexane to 35% ethyl acetate in hexane on silica gel; Predominant enantiomer depicted; (colorless oil, 53.5 mg, 0.128 mmol, 64% yield, 43% ee).

^{1}H NMR (400 MHz, $CDCl_3$) δ 7.81 – 7.73 (m, 2H), 7.41 – 7.31 (m, 2H), 4.97 (dtd, *J* = 7.2, 5.4, 3.1 Hz, 1H), 4.45 (dd, *J* = 11.7, 3.1 Hz, 1H), 4.28 (dd, *J* = 11.7, 5.4 Hz, 1H), 4.21 – 4.11 (m, 2H), 3.08 (s, 3H), 3.07 (s, 3H), 2.45 (s, 3H), 2.18 – 2.07 (m, 2H).

^{13}C{^{1}H} NMR (101 MHz, $CDCl_3$) δ 145.5, 132.4, 130.2, 128.1, 75.3, 69.6, 65.3, 38.7, 37.8, 30.9, 21.8.

IR ν 3054, 2988, 2305, 1599, 1420, 1366, 1266, 1180, 1096, 895, 815, 740, 706, 554, 528 cm^{-1}.

HRMS (ESI) m/z = $[M + Na]^+$ Calcd $C_{13}H_{20}O_9S_3Na^+$ 439.0167. Found 439.0146 (4.8 ppm error).

Specific rotation: $[\alpha]_D^{22}$ = –6.5 (c = 2.4 g/100 mL, $CHCl_3$, 43% ee).

Stereochemistry assigned by analogy to an authentic sample of 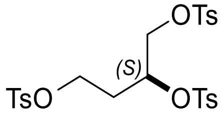

.

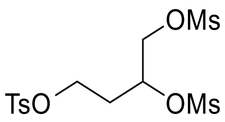

Racemic Sample: HPLC (IA-3, Isopropanol/hexanes = 10/90, flow rate = 1 mL/min, I = 220 nm), t_R = 97.3 min, 116.4 min.

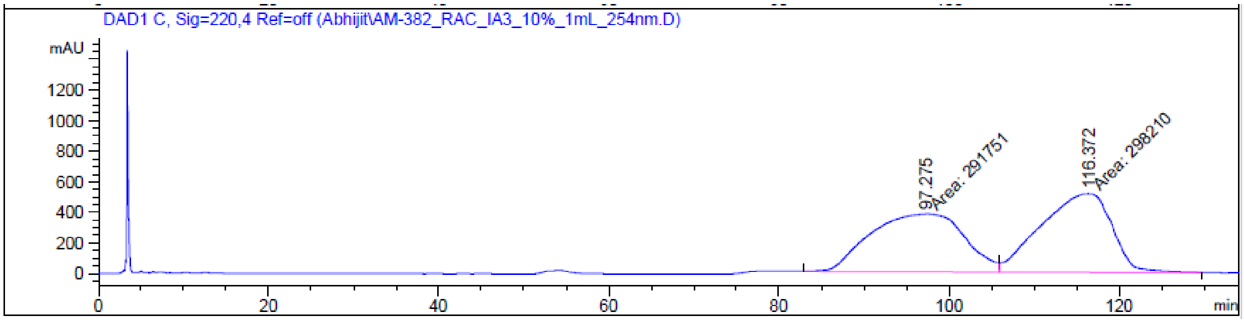

Peak #	RetTime [min]	Type	Width [min]	Area [mAU*s]	Height [mAU]	Area %
1	97.275	MF	12.8291	2.91751e5	379.02377	49.4526
2	116.372	FM	9.6624	2.98210e5	514.38214	50.5474

Scalemic Sample, -43% ee: HPLC (IA-3, Isopropanol/hexanes = 10/90, flow rate = 1 mL/min, I = 220 nm), t_R = 93.5 min, 115.3 min.

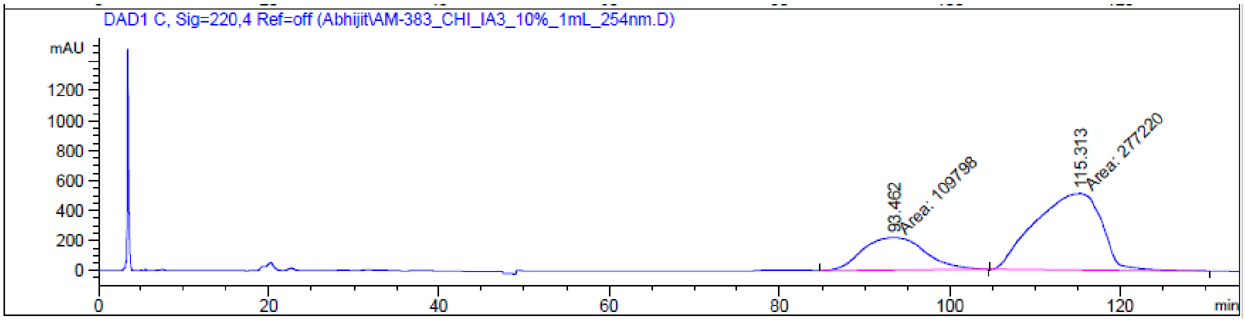

Peak #	RetTime [min]	Type	Width [min]	Area [mAU*s]	Height [mAU]	Area %
1	93.462	MM	8.5113	1.09798e5	215.00406	28.3703
2	115.313	MM	9.0918	2.77220e5	508.18661	71.6297

Compound 3G (CDCl₃, ^{1}H NMR: 400 MHz, ^{13}C{^{1}H} NMR: 101 MHz)

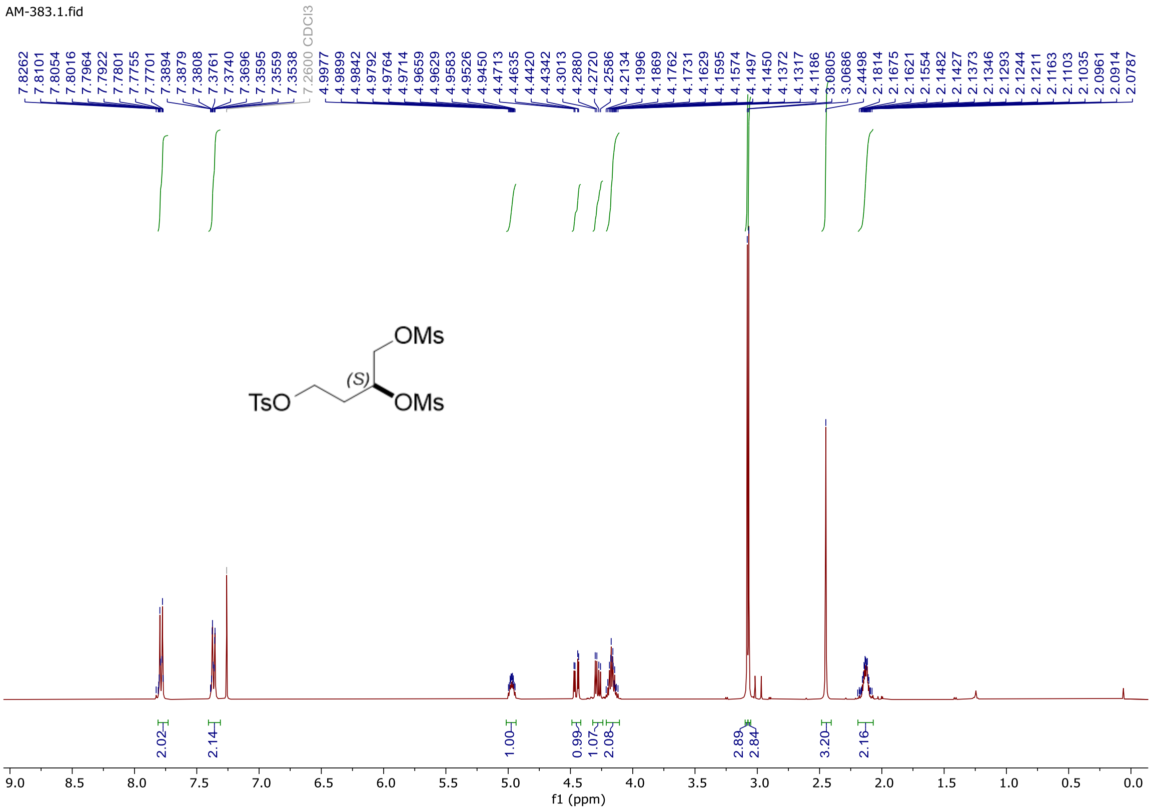

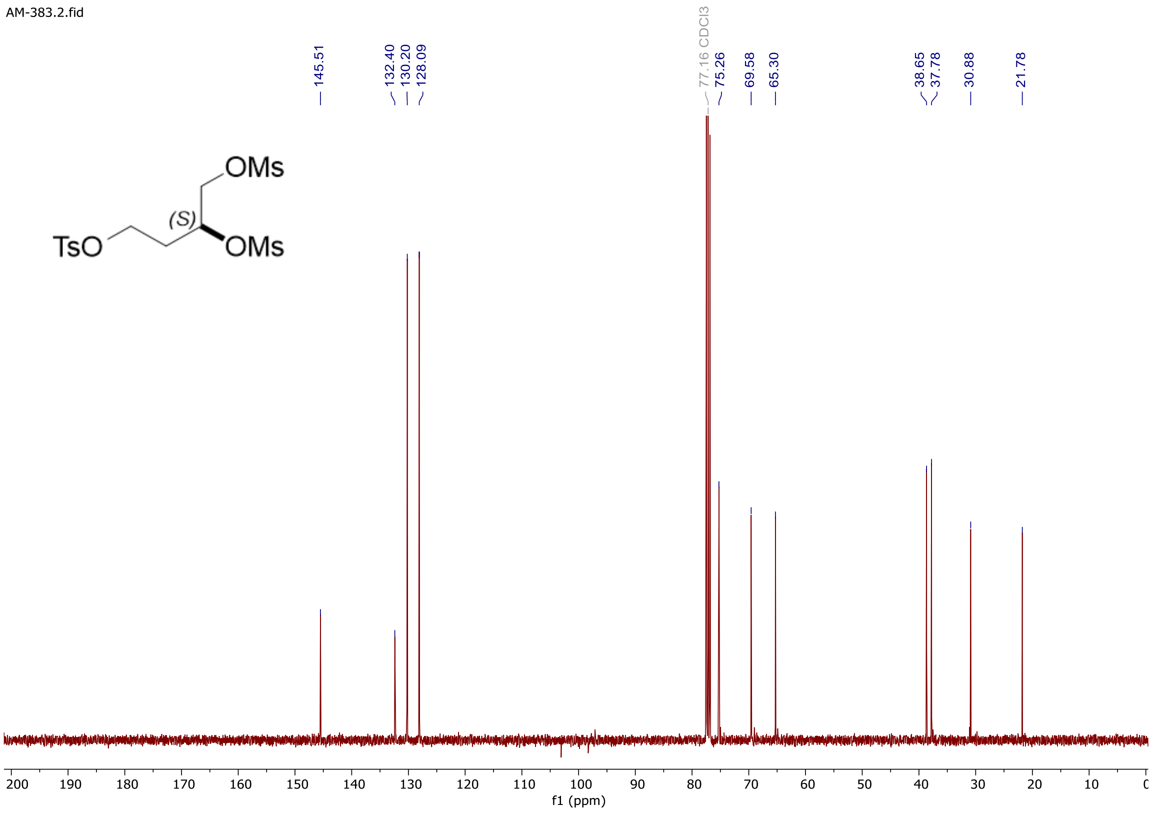

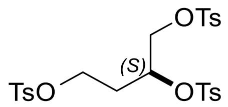

(*S*)-butane-1,2,4-triyl tris(4-methylbenzenesulfonate)

Compound 3H: Synthesized using the General Procedure on a 0.2 mmol scale; Purified using a gradient of 2% ethyl acetate in hexane to 25% ethyl acetate in hexane on silica gel; Predominant enantiomer depicted; (colorless oil, 21.8 mg, 0.038 mmol, 19% yield, 69% ee).

^{1}H NMR (400 MHz, $CDCl_3$) δ 7.76 – 7.66 (m, 6H), 7.39 – 7.29 (m, 6H), 4.69 (tt, *J* = 6.2, 4.2 Hz, 1H), 4.08 – 3.82 (m, 4H), 2.46 (s, 3H), 2.46 (overlapping s, 6H), 2.05 – 1.91 (m, 2H).

^{13}C{^{1}H} NMR (101 MHz, $CDCl_3$) δ 145.7, 145.5, 145.3, 132.8, 132.7, 132.2, 130.19, 130.17, 130.13, 128.1, 128.04, 128.02, 74.9, 69.3, 65.3, 31.0, 21.9, 21.84, 21.80.

IR ν 3054, 2988, 2305, 1599, 1420, 1369, 1266, 1191, 1180, 1096, 895, 749, 706, 554 cm^{-1}.

HRMS (ESI) m/z = $[M + Na]^+$ Calcd $C_{25}H_{28}O_9S_3Na^+$ 591.0793. Found 591.0815 (3.7 ppm error).

Specific rotation: $[\alpha]_D^{21}$ = –19.1 (c = 1.1 g/100 mL, $CHCl_3$, 69% ee).

Stereochemistry assigned by analogy to an authentic sample of 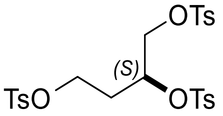 .

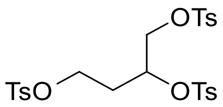

Racemic Sample: HPLC (IA-3, Isopropanol/hexanes = 10/90, flow rate = 1 mL/min, I = 254 nm), t_R = 65.2 min, 77.0 min.

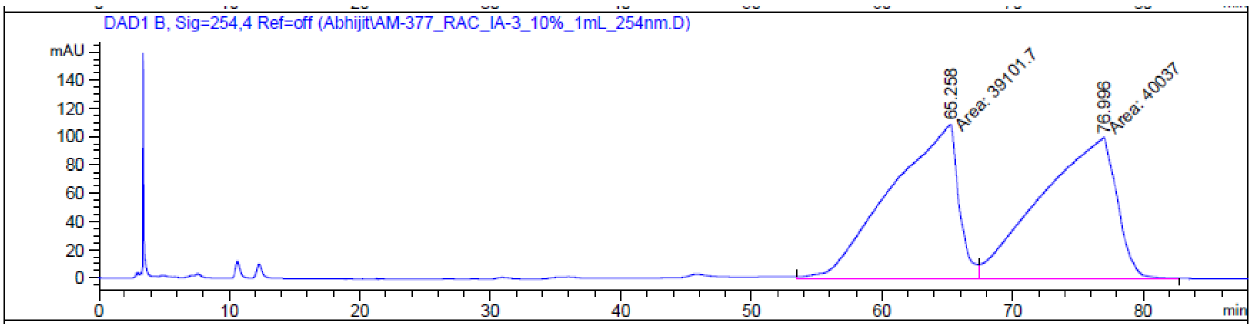

Peak #	RetTime [min]	Type	Width [min]	Area [mAU*s]	Height [mAU]	Area %
1	65.258	MF	5.9610	3.91017e4	109.32719	49.4091
2	76.996	FM	6.6817	4.00370e4	99.86734	50.5909

Scalemic Sample, -69% ee: HPLC (IA-3, Isopropanol/hexanes = 10/90, flow rate = 1 mL/min, I = 254 nm), t_R = 62.5 min, 77.4 min.

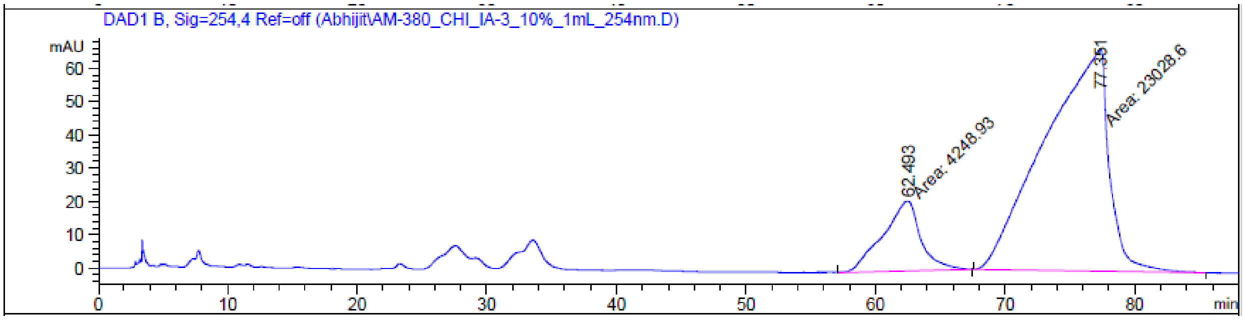

Peak #	RetTime [min]	Type	Width [min]	Area [mAU*s]	Height [mAU]	Area %
1	62.493	MM	3.3898	4248.92969	20.89062	15.5767
2	77.351	MF	5.7607	2.30286e4	66.62522	84.4233

Compound 3H (CDCl₃, ¹H NMR: 400 MHz, ¹³C{¹H} NMR: 101 MHz)

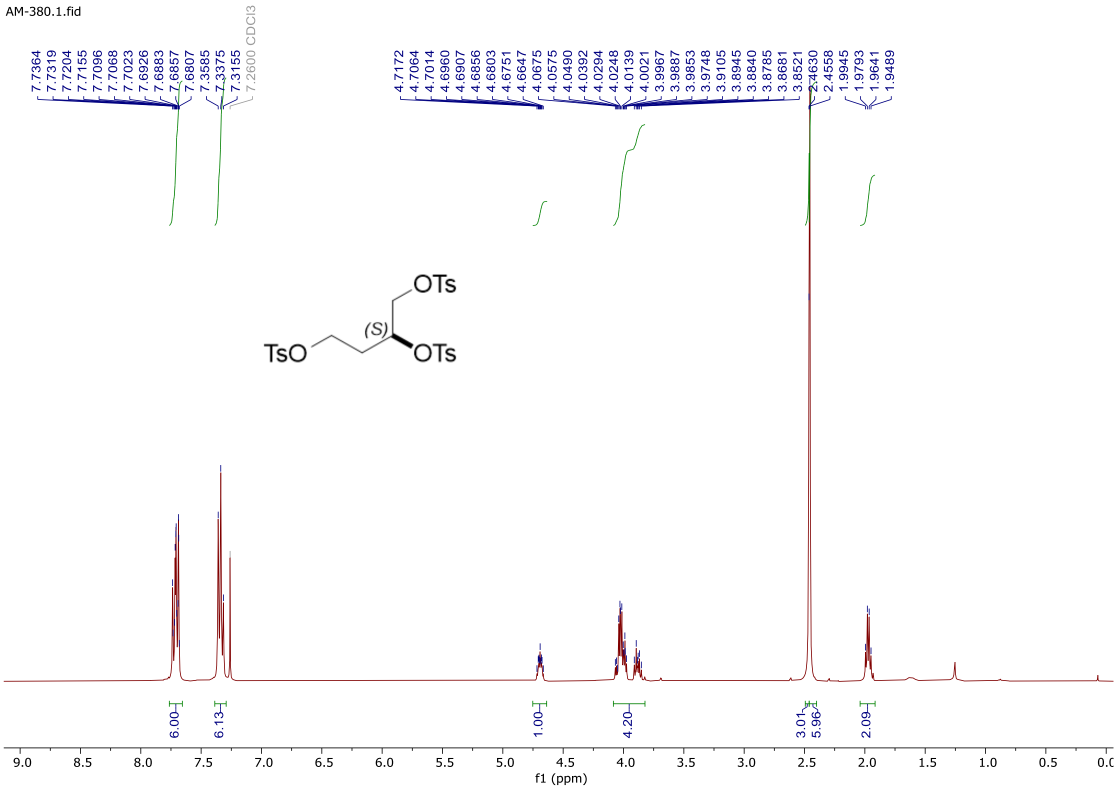

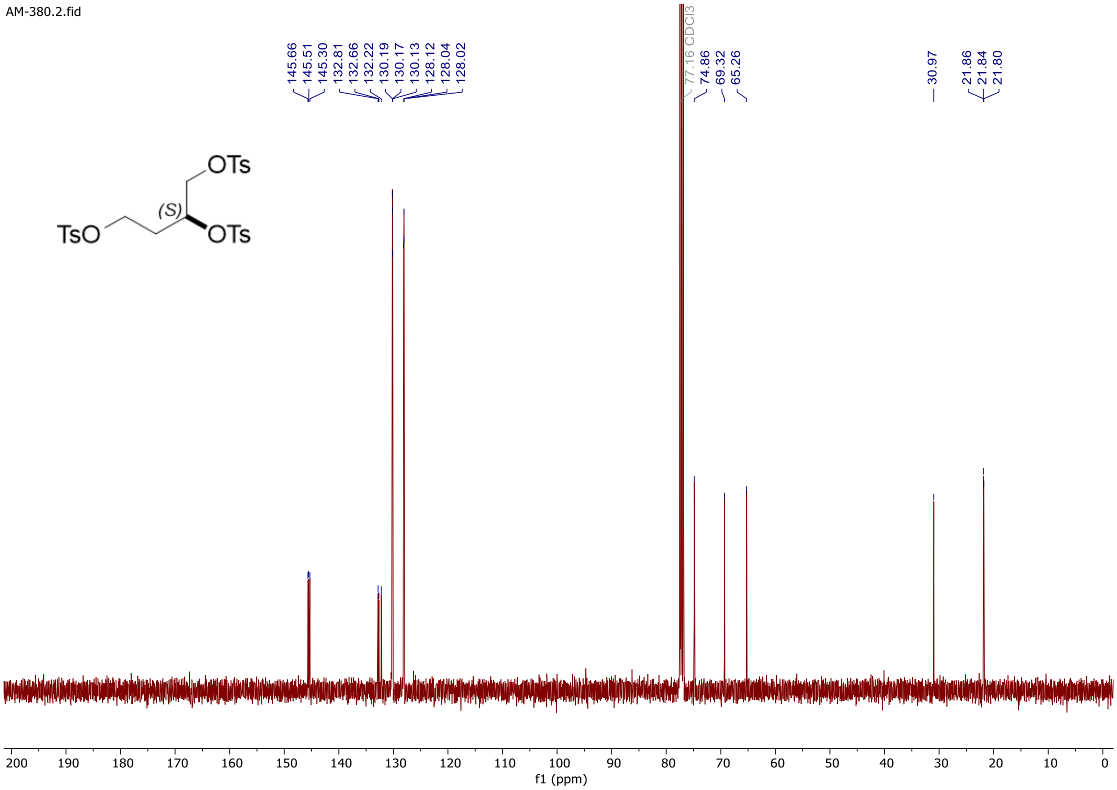

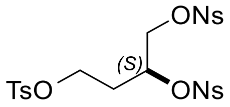

(*S*)-4-(tosyloxy)butane-1,2-diyl bis(4-nitrobenzenesulfonate)

Compound 3I: Synthesized using the General Procedure on a 0.2 mmol scale; Purified using a gradient of 2% ethyl acetate in hexane to 40% ethyl acetate in hexane on silica gel; Predominant enantiomer depicted; (colorless oil, 45.0 mg, 0.071 mmol, 36% yield, 65% ee).

^{1}H NMR (400 MHz, $CDCl_3$) δ 8.45 – 8.35 (m, 4H), 8.13 – 8.01 (m, 4H), 7.69 (d, *J* = 8.4 Hz, 2H), 7.36 (dd, *J* = 8.7, 0.8 Hz, 2H), 4.94 – 4.89 (m, 1H), 4.32 (dd, *J* = 11.6, 3.1 Hz, 1H), 4.22 (dd, *J* = 11.7, 5.2 Hz, 1H), 4.05 (dt, *J* = 10.4, 5.0 Hz, 1H), 3.88 (ddd, *J* = 10.8, 6.8, 5.8 Hz, 1H), 2.46 (s, 3H), 2.10 – 2.03 (m, 2H).

^{13}C{^{1}H} NMR (101 MHz, $CDCl_3$) δ 151.3, 145.7, 141.2, 140.8, 132.2, 130.2, 129.54, 129.46, 128.0, 124.89, 124.87, 76.4, 70.5, 64.7, 31.1, 21.8.

IR ν 3105, 1610, 1541, 1372, 1188, 1093, 947, 861, 754, 554, 465 cm^{-1}.

HRMS (ESI) m/z = $[M + Na]^+$ Calcd $C_{23}H_{22}N_2O_{13}S_3Na^+$ 653.0182. Found 653.0209 (4.1 ppm error).

$[\alpha]_D^{23}$ = +18.2 (c = 0.6 g/100 mL, $CHCl_3$, 65% ee).

Stereochemistry assigned by analogy to an authentic sample of 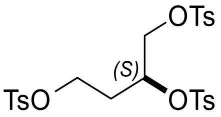 **.**

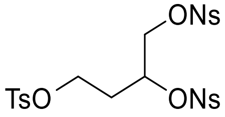

Racemic Sample: SFC (IC, MeOH with 0.1% DEA/CO_2 = 40/60, flow rate = 2 mL/min, I = 220 nm), t_R = 8.2 min, 9.7 min.

MM-02-47-rac

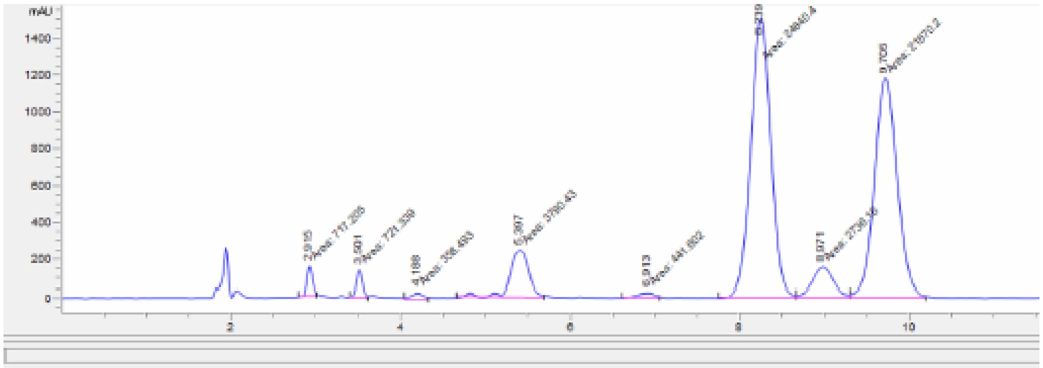

Index	Time (min)	Area (%)
Impurity	2.915	1.293
Impurity	3.501	1.3
Impurity	4.188	0.646
Impurity	5.397	6.833
Impurity	6.913	0.796
Peak-1	8.239	44.777
Impurity	8.971	4.932
Peak-2	9.705	39.423
Total		100.00

Scalemic Sample, -65% ee: SFC (IC, MeOH with 0.1% DEA/CO_2 = 40/60, flow rate = 2 mL/min, I = 220 nm), t_R = 8.2 min, 9.7 min.

MM-02-50-chi

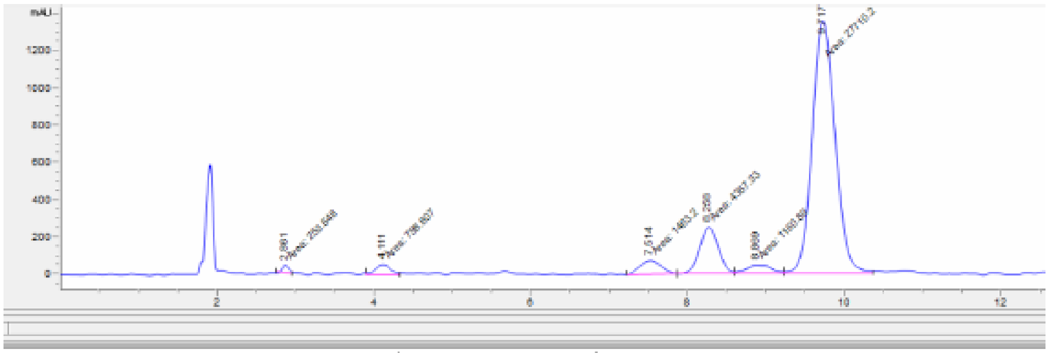

Index	Time (min)	Area (%)
Impurity	2.861	0.71
Impurity	4.111	2.235
Impurity	7.514	4.094
Peak-1	8.258	12.190
Impurity	8.869	3.248
Peak-2	9.717	77.524
Total		100.00

Compound 3I ($CDCl_3$, ^1H NMR: 400 MHz, $^{13}C\{^1H\}$ NMR: 101 MHz)

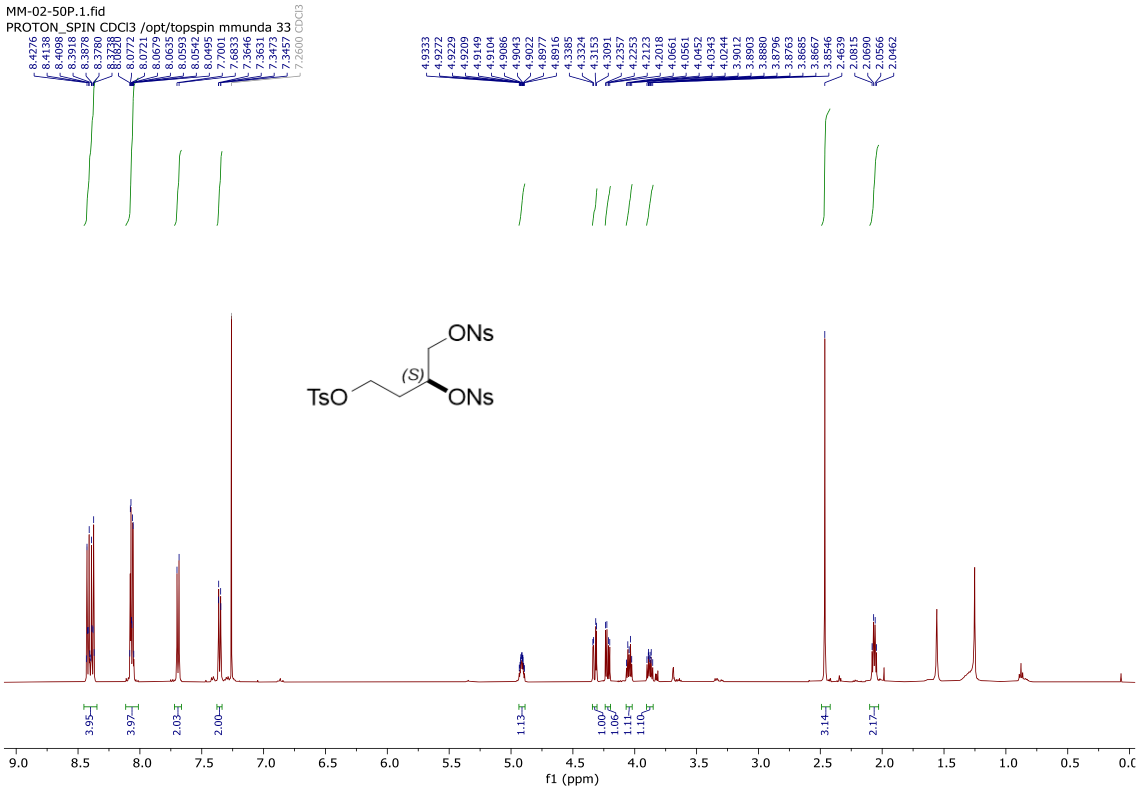

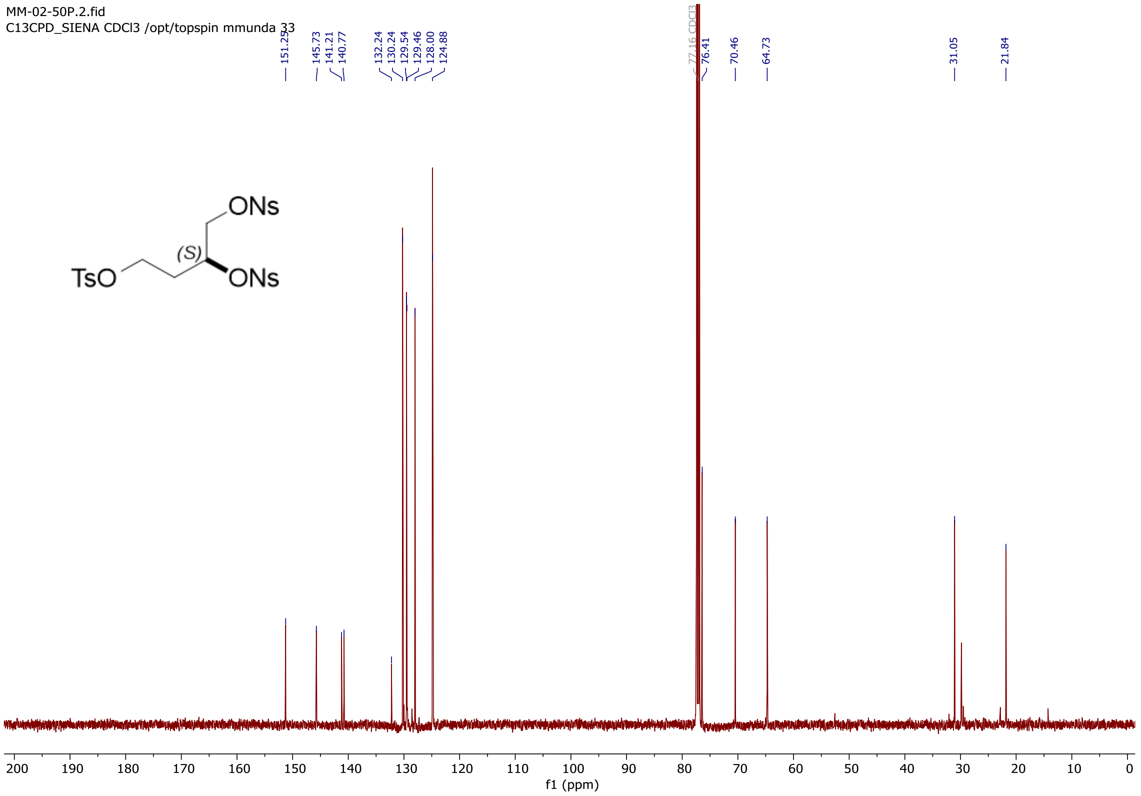

Substrate-3J-K ($CDCl_3$, ^1H NMR: 400 MHz, $^{13}C\{^1H\}$ NMR: 101 MHz)

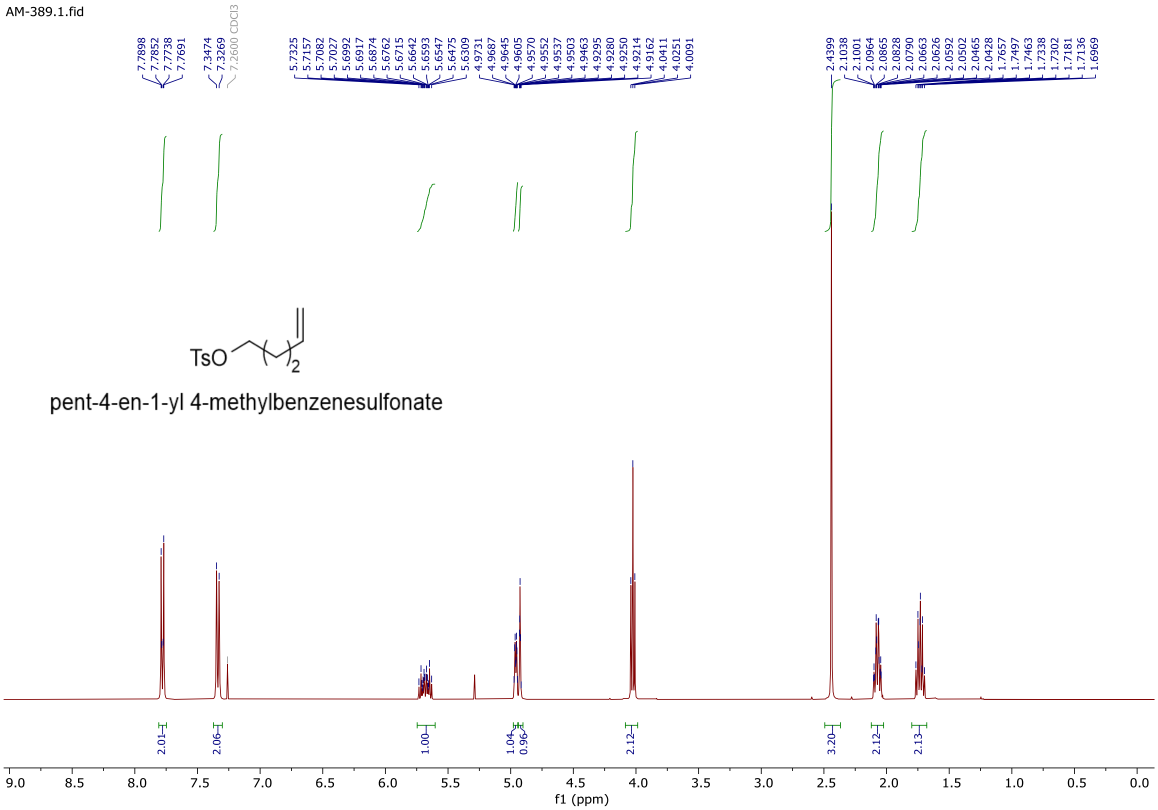

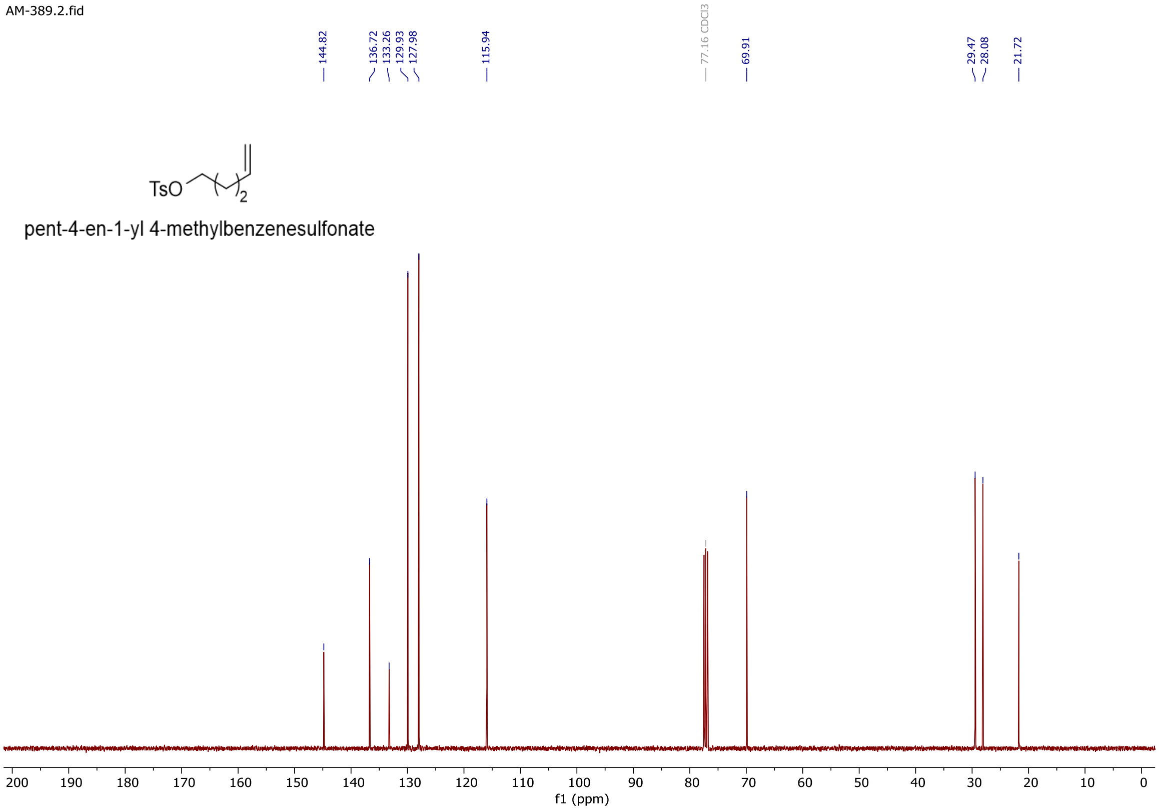

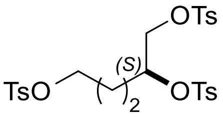

(*S*)-pentane-1,2,5-triyl tris(4-methylbenzenesulfonate)

Compound 3J: Synthesized using the General Procedure on a 0.2 mmol scale; Purified using a gradient of 2% ethyl acetate in hexane to 20% ethyl acetate in hexane on silica gel; Predominant enantiomer depicted; (colorless oil, 86.3 mg, 0.148 mmol, 74% yield, 93% ee).

^{1}H NMR (400 MHz, $CDCl_3$) δ 7.79 – 7.64 (m, 6H), 7.38 – 7.28 (m, 6H), 4.57 (dt, *J* = 7.2, 4.6 Hz, 1H), 3.99 – 3.84 (m, 4H), 2.45 (s, 3H), 2.44 (overlapping s, 6H), 1.69 – 1.49 (m, 4H).

$^{13}C\{^1H\}$ NMR (101 MHz, $CDCl_3$) δ 145.44, 145.43, 145.1, 133.2, 132.8, 132.2, 130.10, 130.07, 130.04, 128.0, 127.9, 77.7, 69.31, 69.26, 27.2, 24.2, 21.8, 21.7.

IR ν 3054, 2988, 2305, 1599, 1366, 1266, 1191, 1177, 1096, 915, 815, 740, 706, 666, 554 cm^{-1}.

HRMS (ESI) m/z = $[M + Na]^+$ Calcd $C_{26}H_{30}O_9S_3Na^+$ 605.0950. Found 605.0966 (2.6 ppm error).

Specific rotation: $[\alpha]_D^{25}$ = –8.3 (c = 2.9 g/100 mL, $CHCl_3$, 93% ee).

Stereochemistry assigned by analogy to an authentic sample of 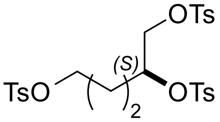 **.**

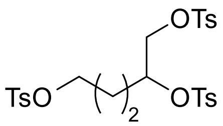

Racemic Sample: HPLC (IC-3, Isopropanol/hexanes = 50/50, flow rate = 1 mL/min, I = 254 nm), t_R = 134.4 min, 151.7 min.

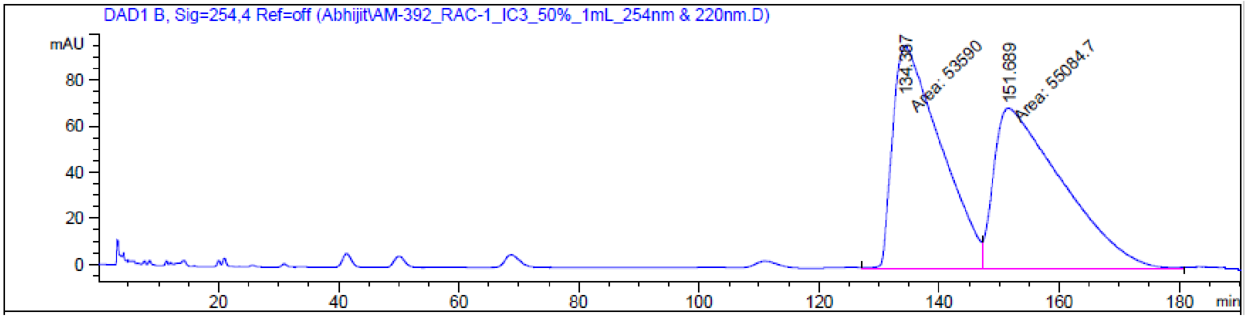

Peak #	RetTime [min]	Type	Width [min]	Area [mAU*s]	Height [mAU]	Area %
1	134.387	MF	9.2094	5.35900e4	96.98395	49.3123
2	151.689	FM	13.1915	5.50847e4	69.59633	50.6877

Scalemic Sample, +93% ee: HPLC (IC-3, Isopropanol/hexanes =50/50, flow rate = 1 mL/min, I = 254 nm), t_R = 132.9 min, 163.7 min.

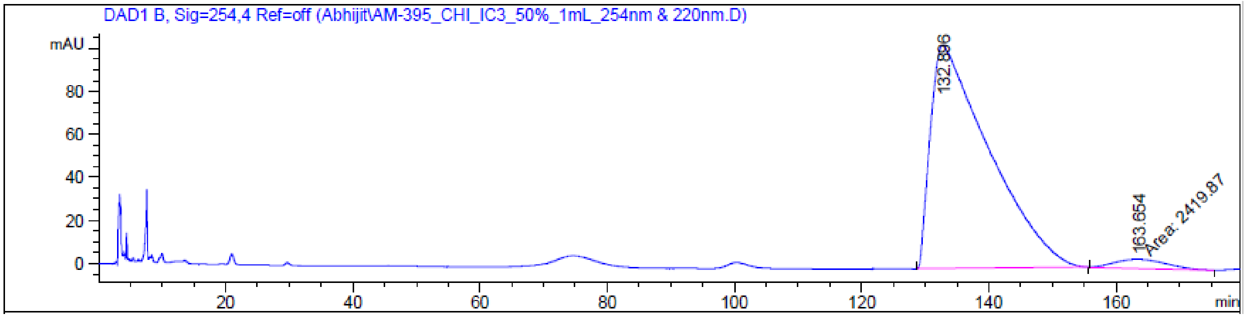

Peak #	RetTime [min]	Type	Width [min]	Area [mAU*s]	Height [mAU]	Area %
1	132.896	BB	7.5136	6.66341e4	103.82426	96.4957
2	163.654	MM	9.3190	2419.86792	4.32785	3.5043

Compound 3J ($CDCl_3$, ^1H NMR: 400 MHz, $^{13}C\{^1H\}$ NMR: 101 MHz)

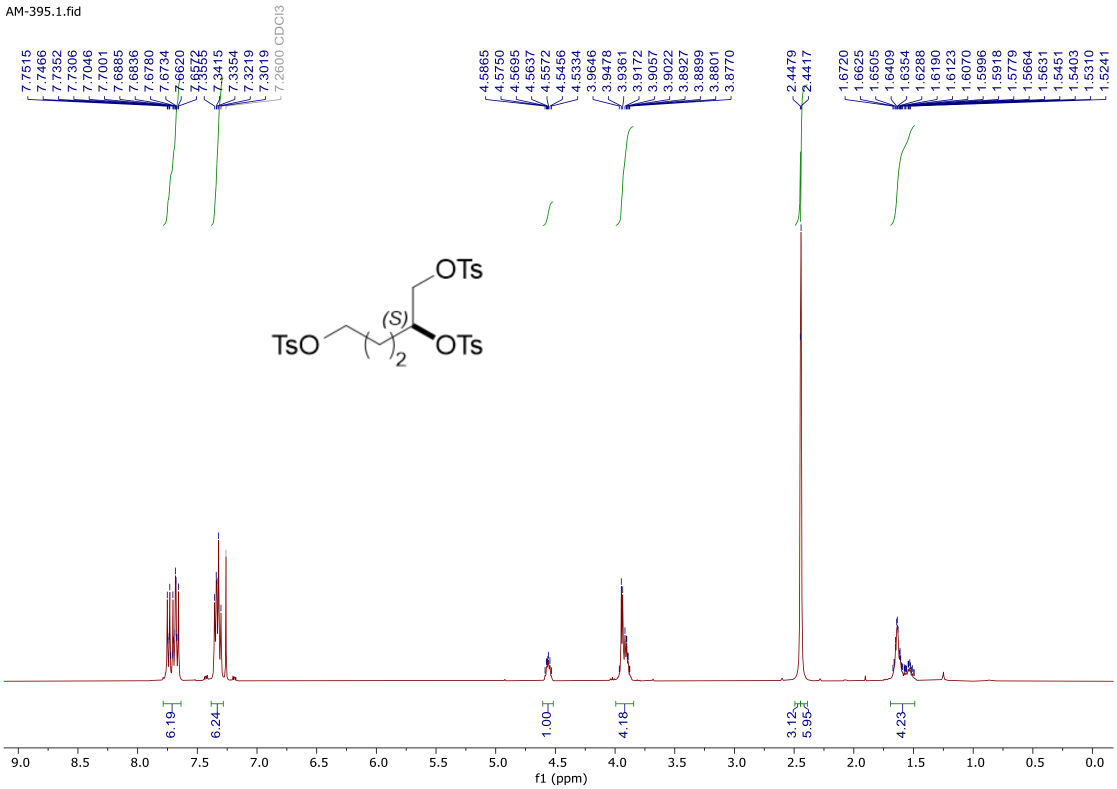

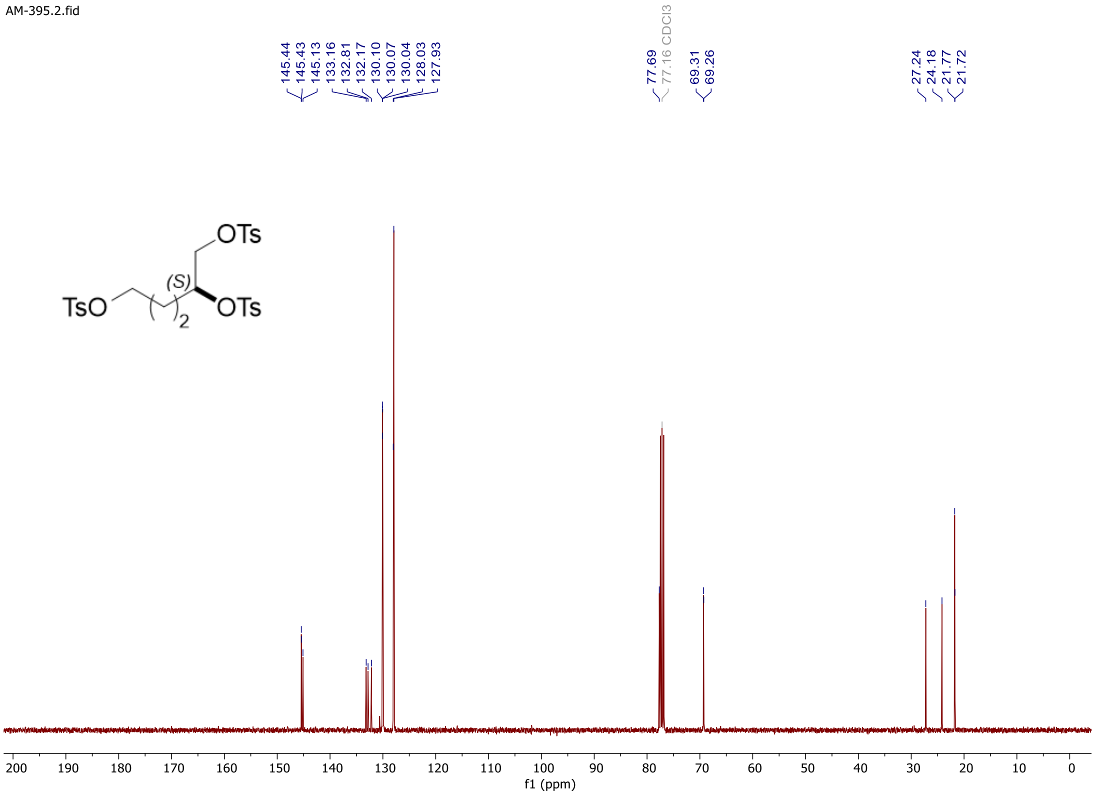

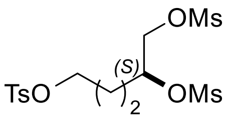

(*S*)-5-(tosyloxy)pentane-1,2-diyl dimethanesulfonate

Compound 3K: Synthesized using the General Procedure on a 0.2 mmol scale; Purified using a gradient of 2% ethyl acetate in hexane to 35% ethyl acetate in hexane on silica gel; Predominant enantiomer depicted; (colorless oil, 53.0 mg, 0.123 mmol, 62% yield, 80% ee).

^{1}H NMR (400 MHz, $CDCl_3$) δ 7.82 – 7.72 (m, 2H), 7.35 (d, *J* = 8.1 Hz, 2H), 4.84 (qd, *J* = 6.0, 2.8 Hz, 1H), 4.36 (dd, *J* = 11.6, 3.1 Hz, 1H), 4.23 (dd, *J* = 11.6, 6.3 Hz, 1H), 4.14 – 3.98 (m, 2H), 3.08 (s, 3H), 3.07 (s, 3H), 2.44 (s, 3H), 1.89 – 1.74 (m, 4H).

$^{13}C\{^1H\}$ NMR (101 MHz, $CDCl_3$) δ 145.2, 132.7, 130.1, 128.0, 78.1, 69.6, 69.4, 38.8, 37.8, 27.3, 24.5, 21.7.

IR ν 3054, 2987, 2306, 1422, 1363, 1266, 1178, 972, 919, 897, 815, 742, 705, 664, 554 cm^{-1}.

HRMS (ESI) m/z = $[M + Na]^+$ Calcd $C_{14}H_{22}O_9S_3Na^+$ 453.0324. Found 453.0339 (3.3 ppm error).

Specific Rotation: $[\alpha]_D^{24}$ = +3.5 (c = 2.55 g/100 mL, $CHCl_3$, 80% ee).

Stereochemistry assigned by analogy to an authentic sample of 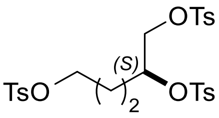 **.**

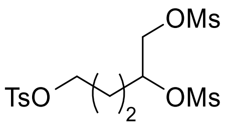

Racemic Sample: HPLC (IC-3, Isopropanol/hexanes = 40/60, flow rate = 1 mL/min, I = 220 nm), t_R = 129.2 min, 138.8 min.

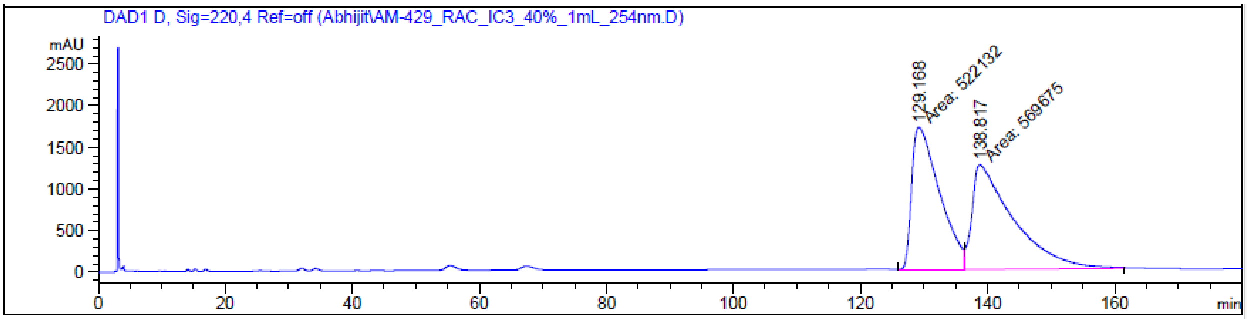

Peak #	RetTime [min]	Type	Width [min]	Area [mAU*s]	Height [mAU]	Area %
1	129.168	MF	5.0790	5.22132e5	1713.37134	47.8227
2	138.817	FM	7.5610	5.69675e5	1255.73621	52.1773

Scalemic Sample, +80% ee: HPLC (IC-3, Isopropanol/hexanes =40/60, flow rate = 1 mL/min, I = 220 nm), t_R = 130.7 min, 147.0 min.

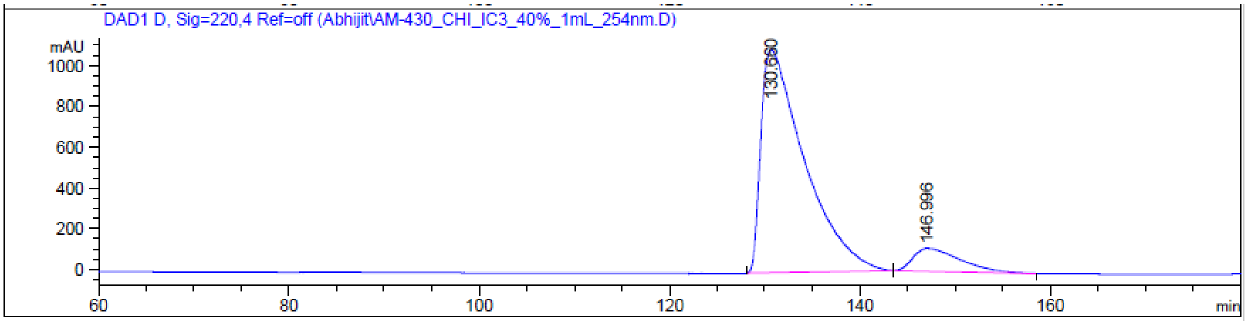

Peak #	RetTime [min]	Type	Width [min]	Area [mAU*s]	Height [mAU]	Area %
1	130.660	BB	3.7288	3.48757e5	1095.90405	90.0230
2	146.996	BB	4.0323	3.86516e4	111.99839	9.9770

Compound 3K ($CDCl_3$, ^1H NMR: 400 MHz, $^{13}C\{^1H\}$ NMR: 101 MHz)

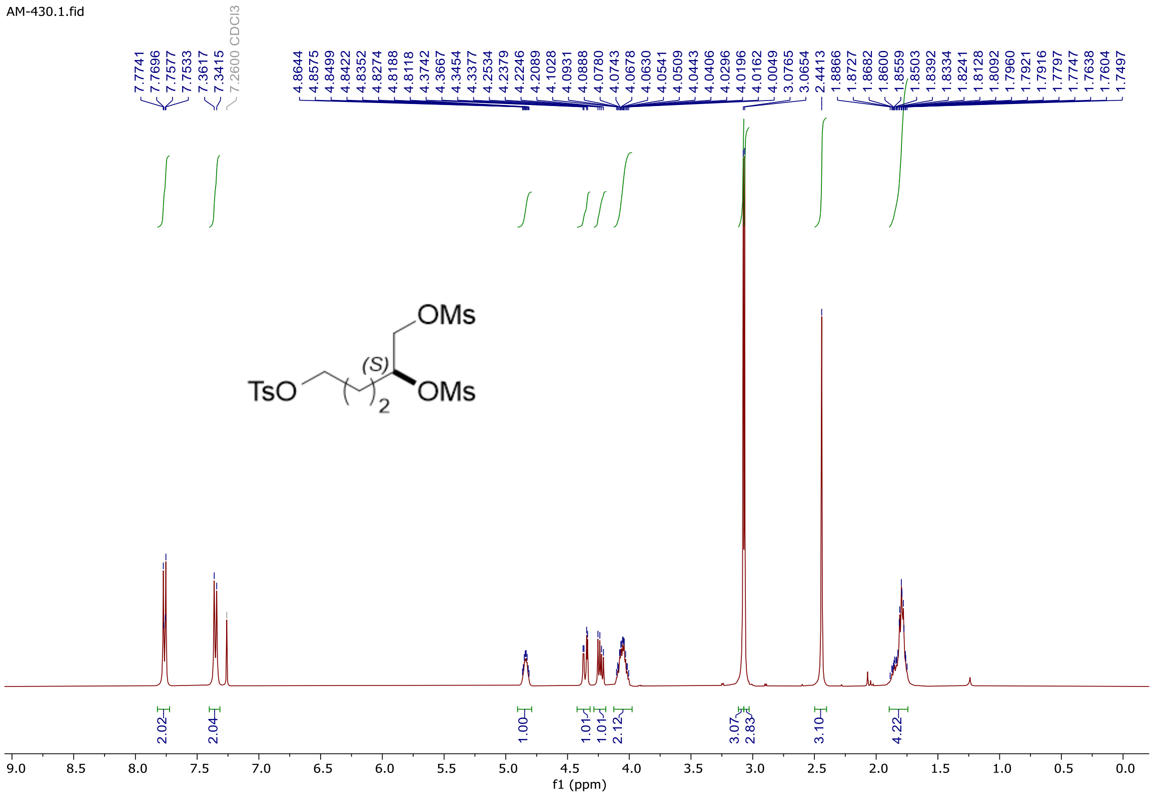

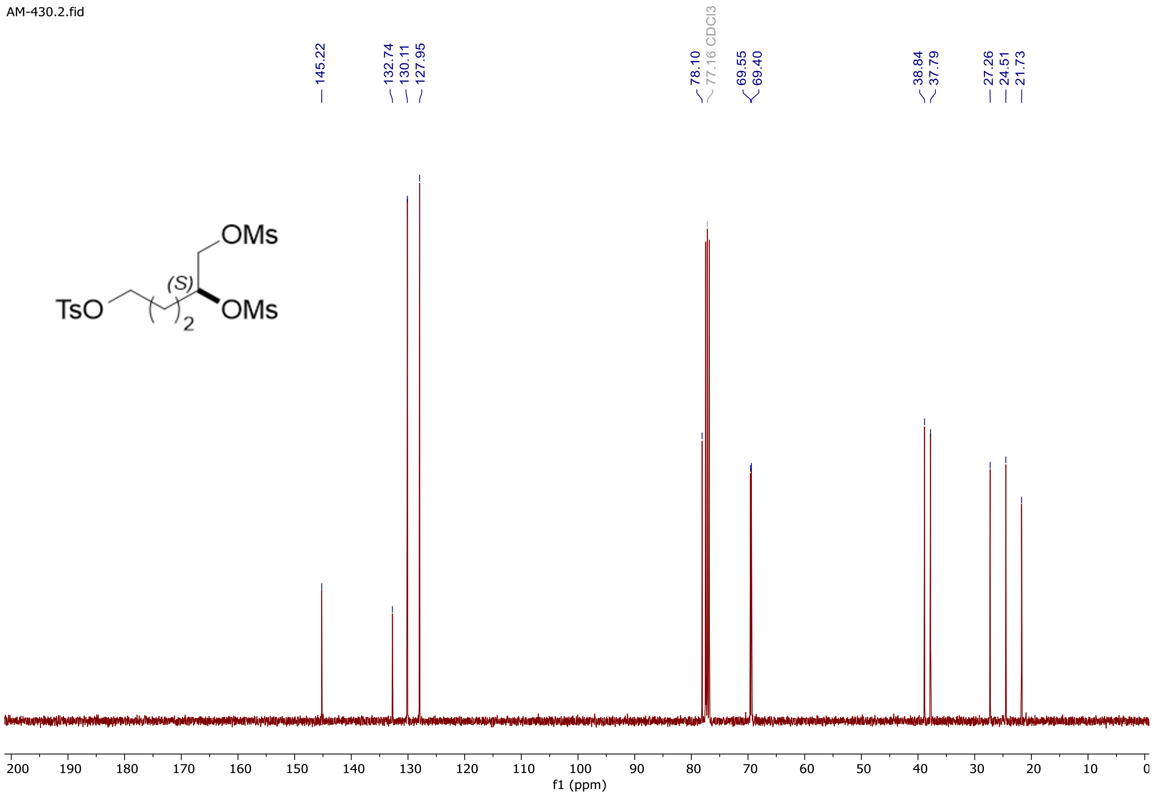

Substrate-3L ($CDCl_3$, ^1H NMR: 400 MHz, $^{13}C\{^1H\}$ NMR: 101 MHz)

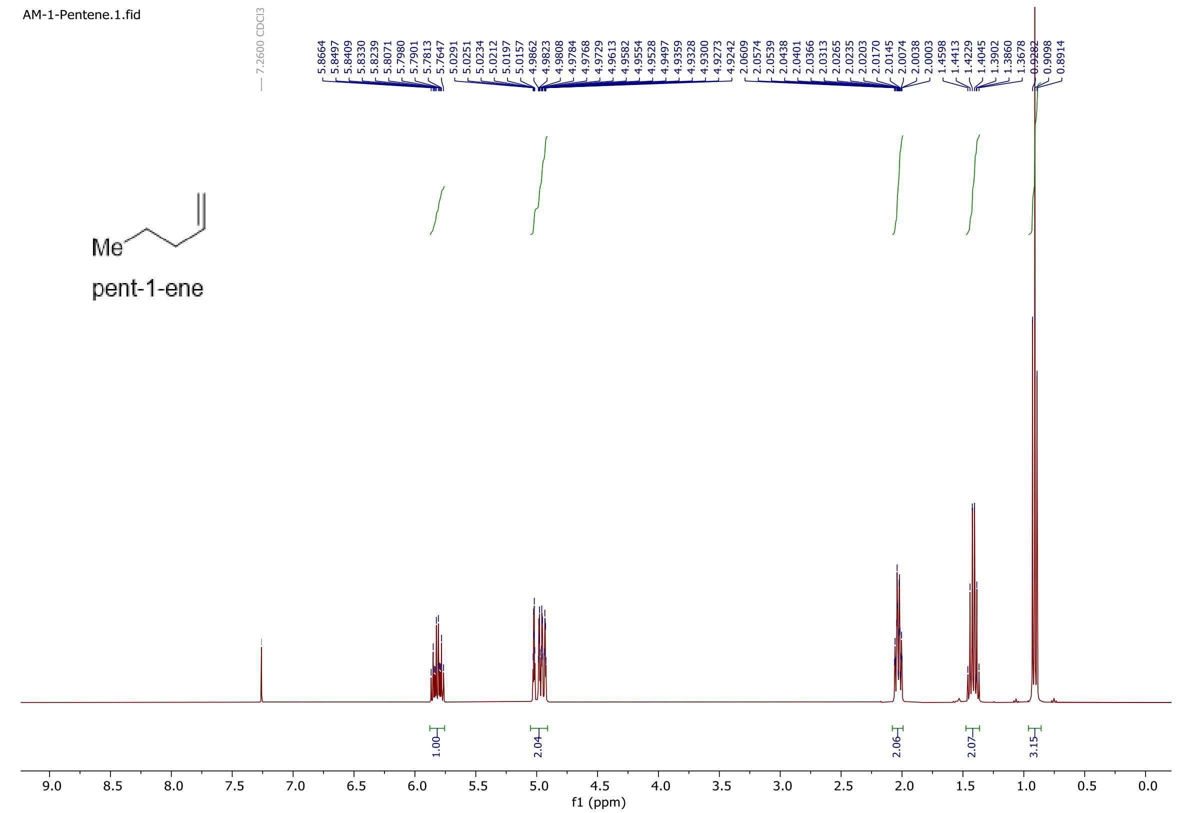

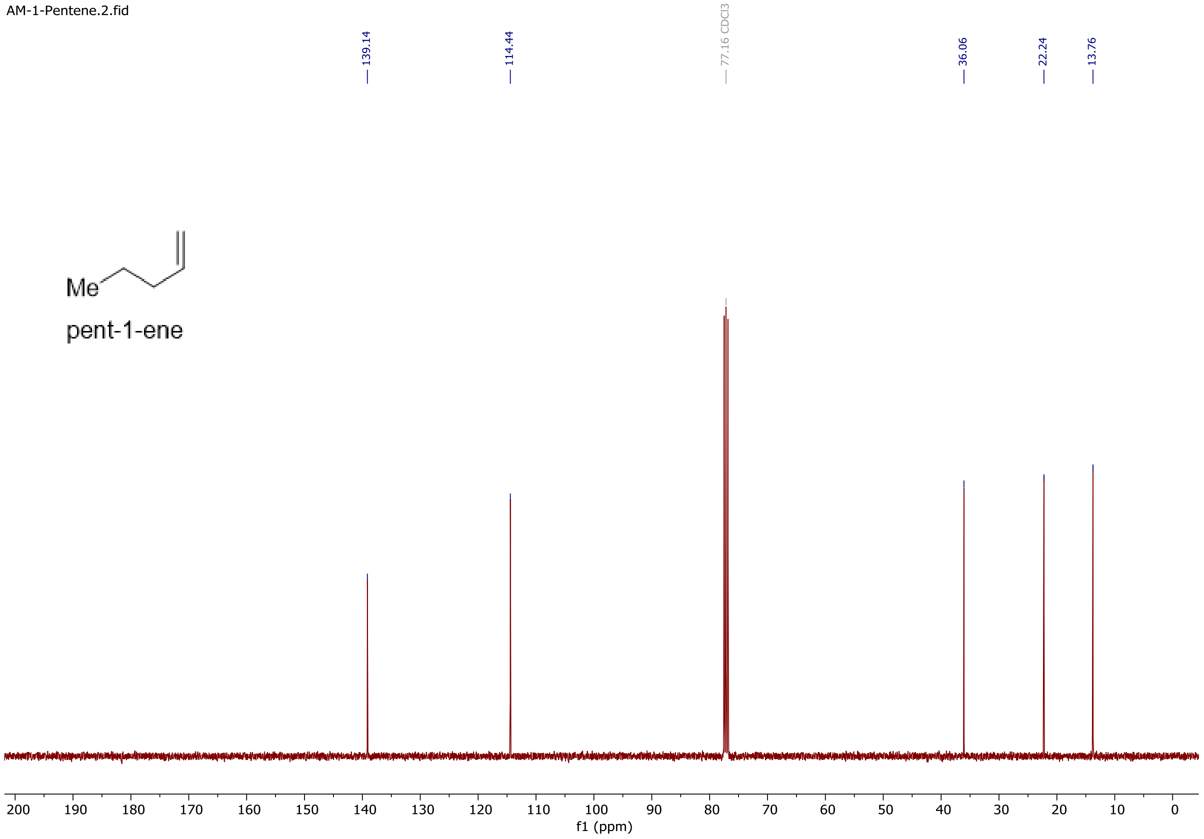

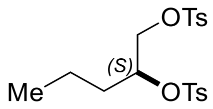

(*S*)-pentane-1,2-diyl bis(4-methylbenzenesulfonate)

Compound 3L: Synthesized using the General Procedure on a 0.2 mmol scale; Purified using a gradient of 2% ethyl acetate in hexane to 8% ethyl acetate in hexane on silica gel; Predominant enantiomer depicted; (colorless oil, 50.0 mg, 0.121 mmol, 61% yield, 82% ee).

^{1}H NMR (400 MHz, $CDCl_3$) δ 7.76 – 7.66 (m, 4H), 7.39 – 7.28 (m, 4H), 4.65 – 4.56 (m, 1H), 4.08 – 3.96 (m, 2H), 2.45 (s, 3H), 2.44 (s, 3H), 1.68 – 1.50 (m, 2H), 1.31 – 1.13 (m, 2H), 0.80 (t, *J* = 7.4 Hz, 3H).

^{13}C{^{1}H} NMR (101 MHz, $CDCl_3$) δ 145.2, 145.0, 133.5, 132.4, 130.0, 129.9, 128.0, 127.9, 78.7, 69.5, 33.1, 21.7, 17.9, 13.6.

IR ν 3057, 2965, 2876, 2305, 1599, 1366, 1266, 1191, 1177, 1096, 918, 815, 746, 706, 665, 554 cm^{-1}.

HRMS (ESI) m/z = $[M + Na]^+$ Calcd $C_{19}H_{24}O_6S_2Na^+$ 435.0912. Found 435.0890 (5.1 ppm error).

Specific Rotation: $[\alpha]_D^{24}$ = –11.2 (c = 2.2 g/100 mL, $CHCl_3$, 82% ee).

Stereochemistry assigned by comparison with an authentic sample of 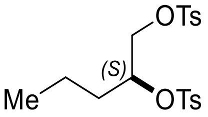

.

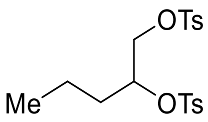

Racemic Sample: HPLC (IC-3, Isopropanol/hexanes = 20/80, flow rate = 1 mL/min, I = 254 nm), t_R = 42.9 min, 47.6 min.

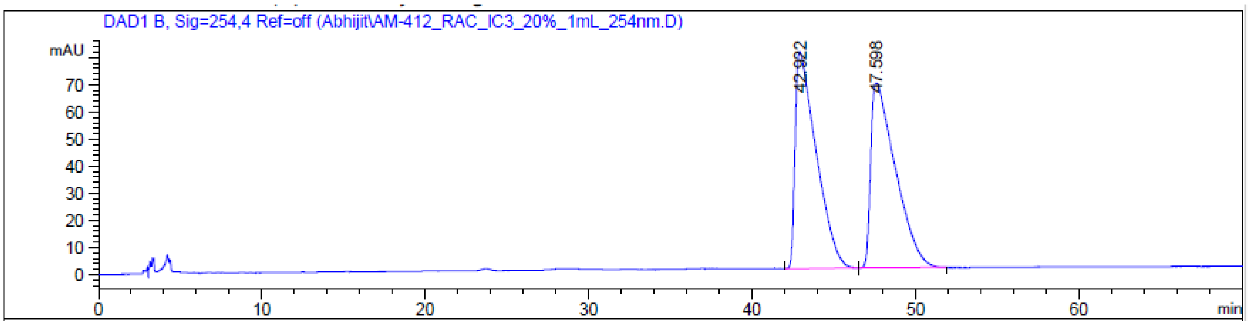

Peak #	RetTime [min]	Type	Width [min]	Area [mAU*s]	Height [mAU]	Area %
1	42.922	BB	1.2197	7147.93896	79.90186	50.0029
2	47.598	BB	1.3728	7147.11426	68.18777	49.9971

Scalemic Sample, +82% ee: HPLC (IC-3, Isopropanol/hexanes =20/80, flow rate = 1 mL/min, I = 254 nm), t_R = 38.7 min, 46.7 min.

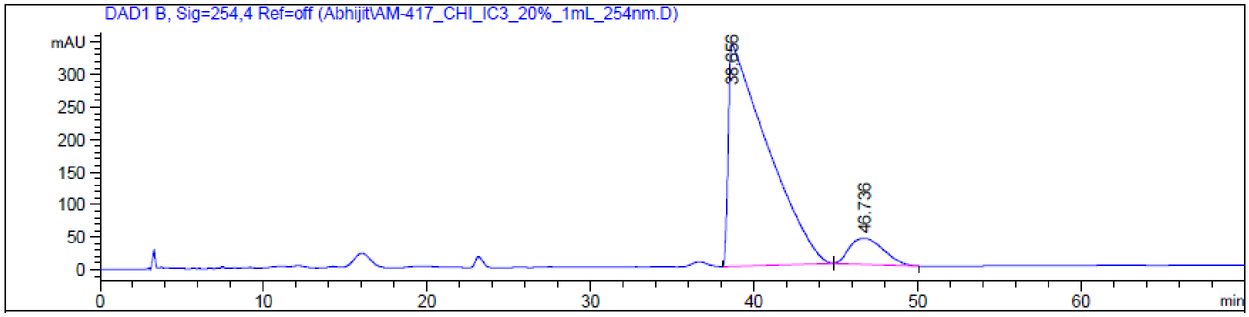

Peak #	RetTime [min]	Type	Width [min]	Area [mAU*s]	Height [mAU]	Area %
1	38.656	BB	2.0616	5.67003e4	341.05994	90.7946
2	46.736	BB	1.6935	5748.68115	39.98056	9.2054

Compound 3L (CDCl₃, ¹H NMR: 400 MHz, ¹³C{¹H} NMR: 101 MHz)

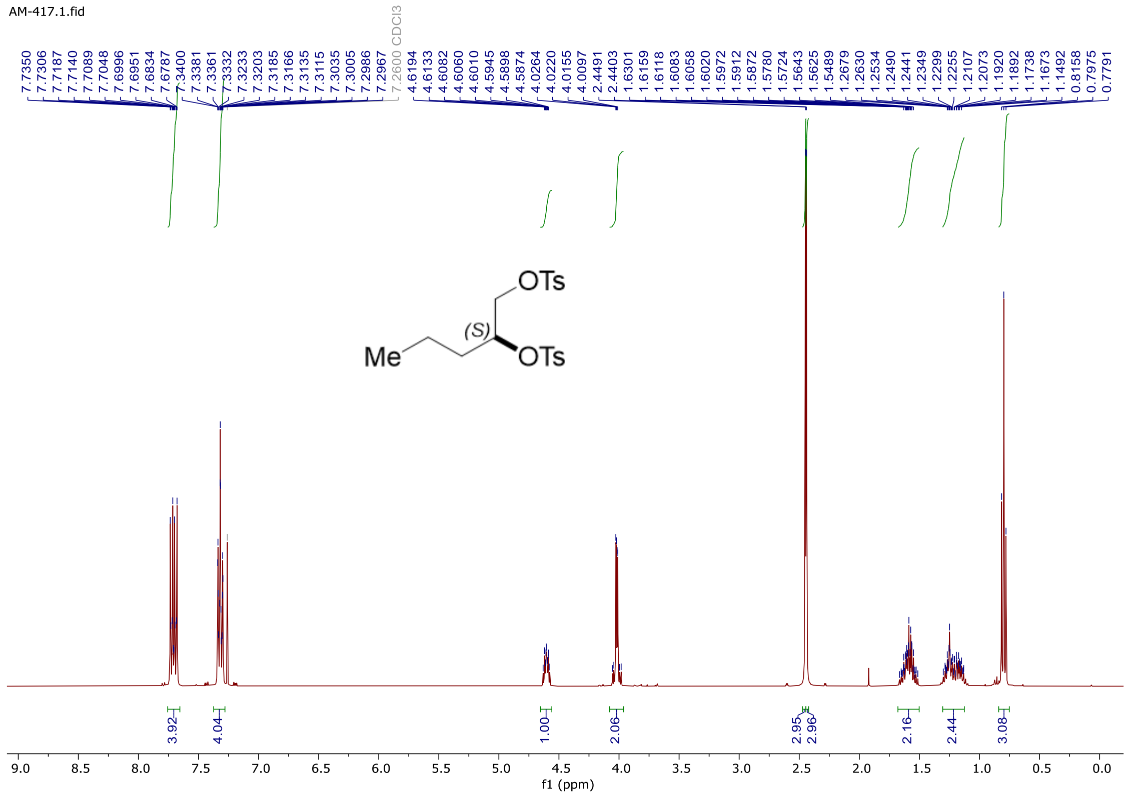

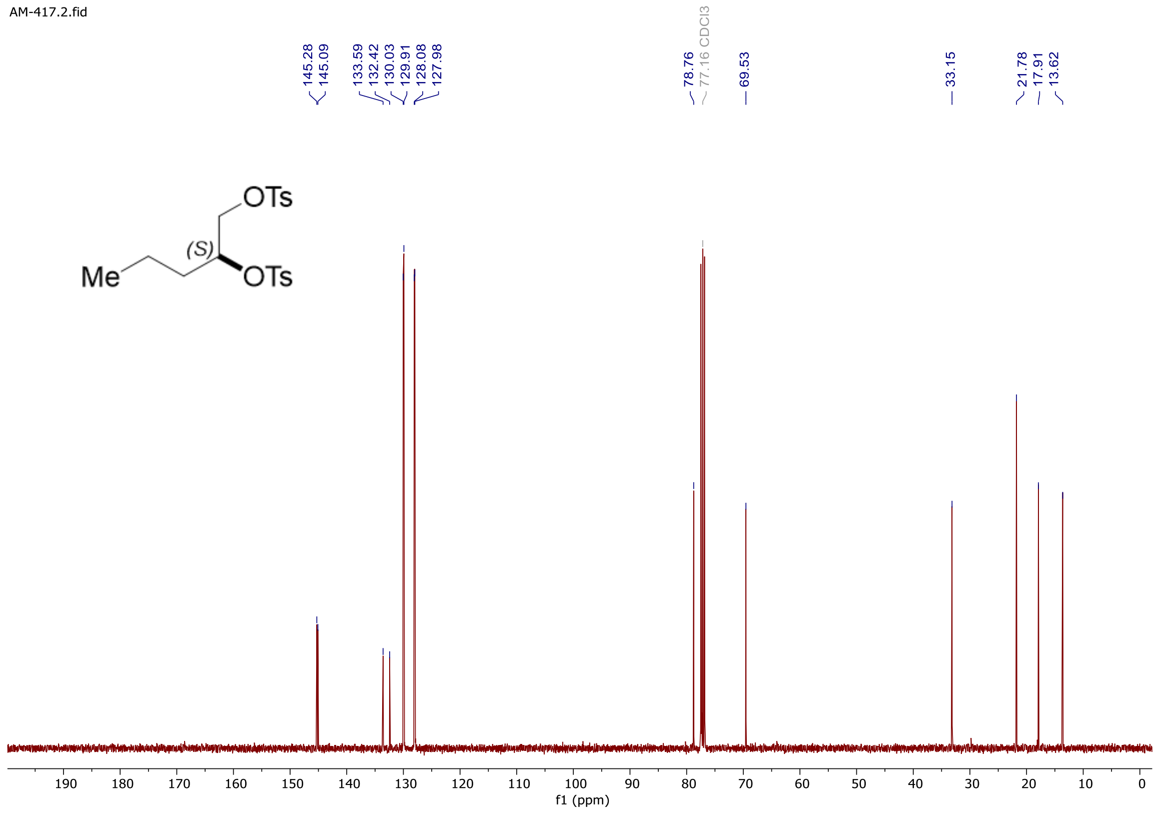

Substrate-3M ($CDCl_3$, ^{1}H NMR: 400 MHz, $^{13}C\{^1H\}$ NMR: 101 MHz)

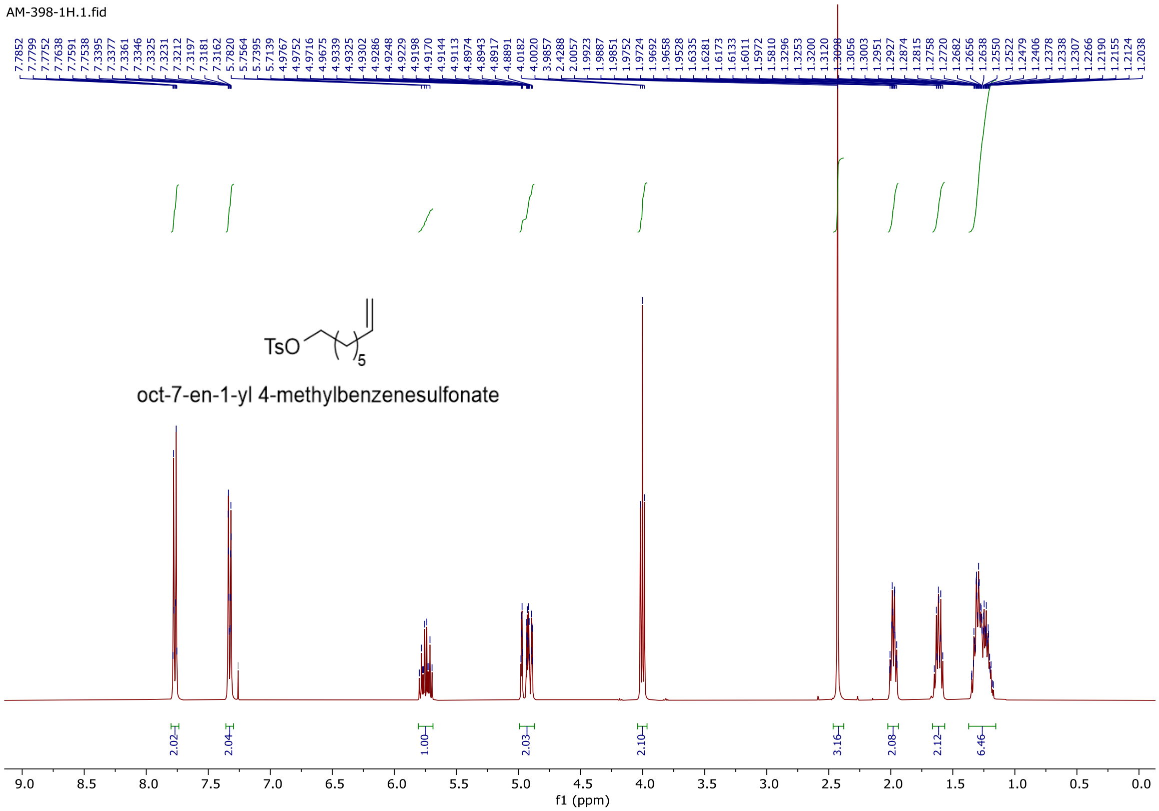

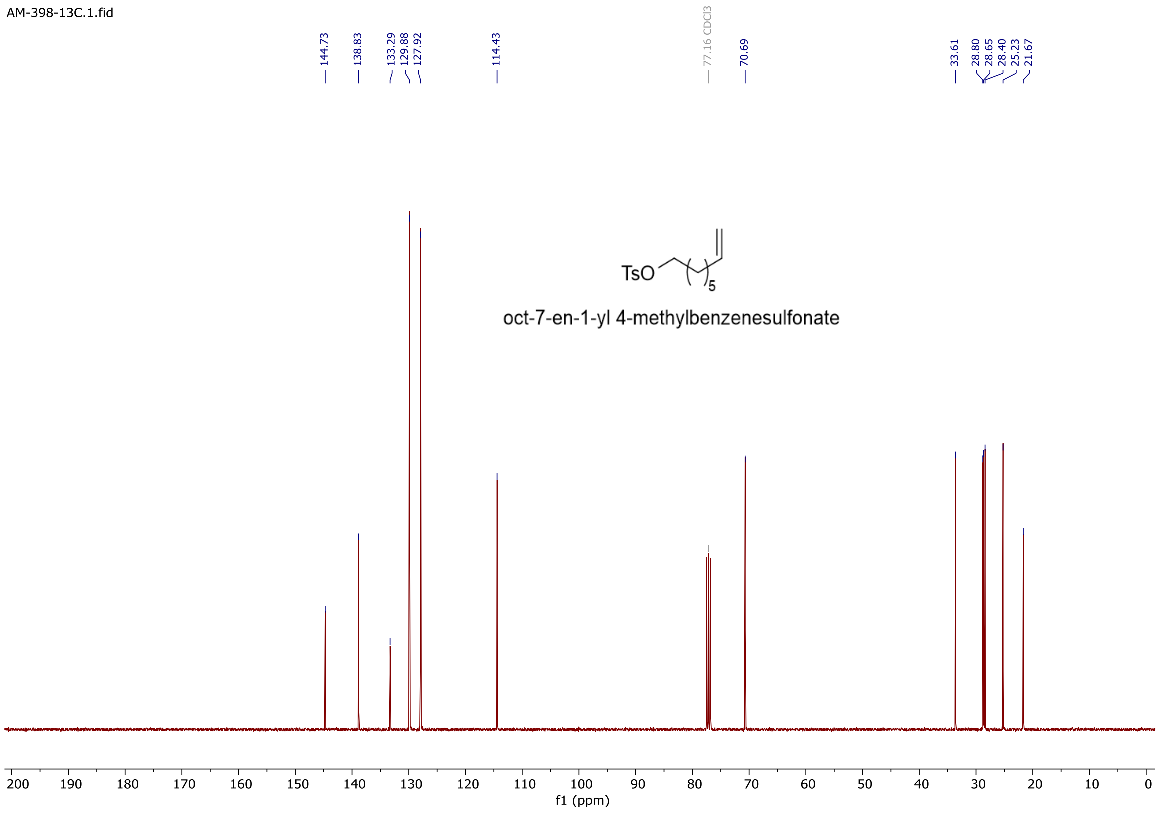

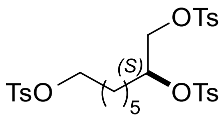

(*S*)-octane-1,2,8-triyl tris(4-methylbenzenesulfonate)

Compound 3M: Synthesized using the General Procedure on a 0.2 mmol scale; Purified using a gradient of 2% ethyl acetate in hexane to 20% ethyl acetate in hexane on silica gel; Predominant enantiomer depicted; (colorless oil, 98.6 mg, 0.158 mmol, 79% yield, 90% ee).

^{1}H NMR (400 MHz, $CDCl_3$) δ 7.80 – 7.74 (m, 2H), 7.73 – 7.65 (m, 4H), 7.37 – 7.28 (m, 6H), 4.56 (dq, *J* = 7.3, 4.9 Hz, 1H), 4.03 – 3.92 (m, 4H), 2.44 (s, 3H), 2.43 (overlapping s, 6H), 1.62 – 1.45 (m, 4H), 1.26 – 1.01 (m, 6H).

^{13}C{^{1}H} NMR (101 MHz, $CDCl_3$) 145.3, 145.2, 144.9, 133.5, 133.2, 132.3, 130.04, 129.95, 129.93, 128.0, 127.93, 127.92, 78.7, 70.5, 69.4, 30.9, 28.6, 28.4, 25.1, 24.2, 21.8, 21.73, 21.70.

IR ν 3057, 2942, 2862, 1599, 1363, 1266, 1191, 1177, 1096, 915, 815, 740, 703, 666, 554 cm^{-1}.

HRMS (ESI) m/z = $[M + Na]^+$ Calcd $C_{29}H_{36}O_9S_3Na^+$ 647.1419. Found 647.1410 (1.4 ppm error).

Specific Rotation: $[\alpha]_D^{23}$ = +8.7 (c = 4.55 g/100 mL, $CHCl_3$, 90% ee).

Absolute stereochemistry assigned by analogy to an authentic sample of 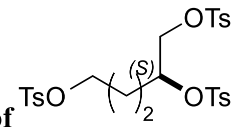 **.**

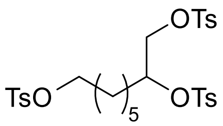

Racemic Sample: HPLC (IC-3, Isopropanol/hexanes = 50/50, flow rate = 1 mL/min, I = 220 nm), t_R = 164.6 min, 186.7 min.

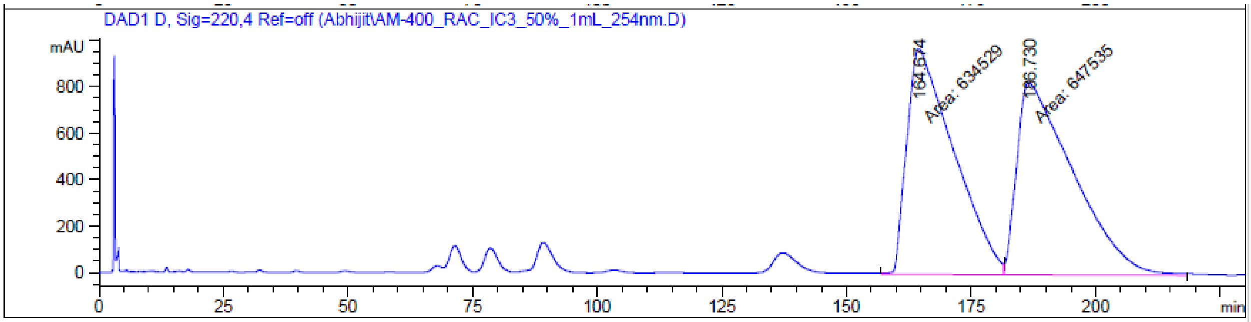

Peak #	RetTime [min]	Type	Width [min]	Area [mAU*s]	Height [mAU]	Area %
1	164.674	MF	10.9340	6.34529e5	967.21271	49.4928
2	186.730	FM	13.0370	6.47535e5	827.81927	50.5072

Scalemic Sample, +90% ee: HPLC (IC-3, Isopropanol/hexanes =50/50, flow rate = 1 mL/min, I = 220 nm), t_R = 157.6 min, 199.5 min.

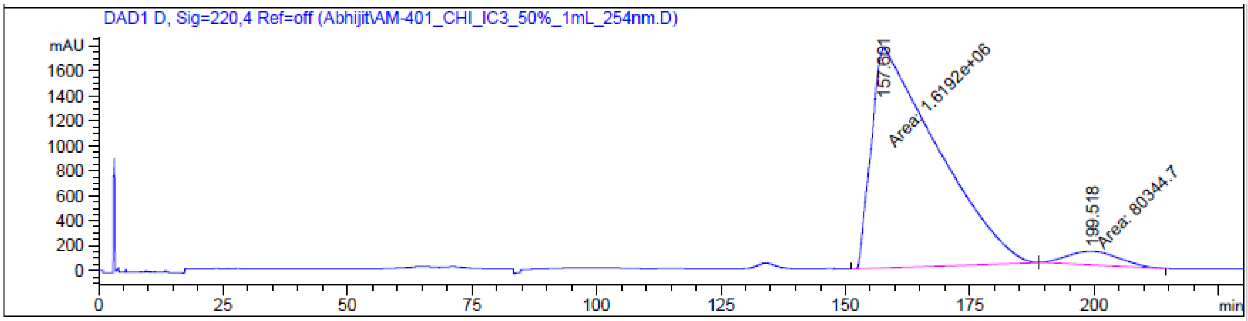

Peak #	RetTime [min]	Type	Width [min]	Area [mAU*s]	Height [mAU]	Area %
1	157.601	MM	15.3165	1.61920e6	1761.93140	95.2726
2	199.518	MM	12.0530	8.03447e4	111.09946	4.7274

Compound 3M ($CDCl_3$, ^1H NMR: 400 MHz, $^{13}C\{^1H\}$ NMR: 101 MHz)

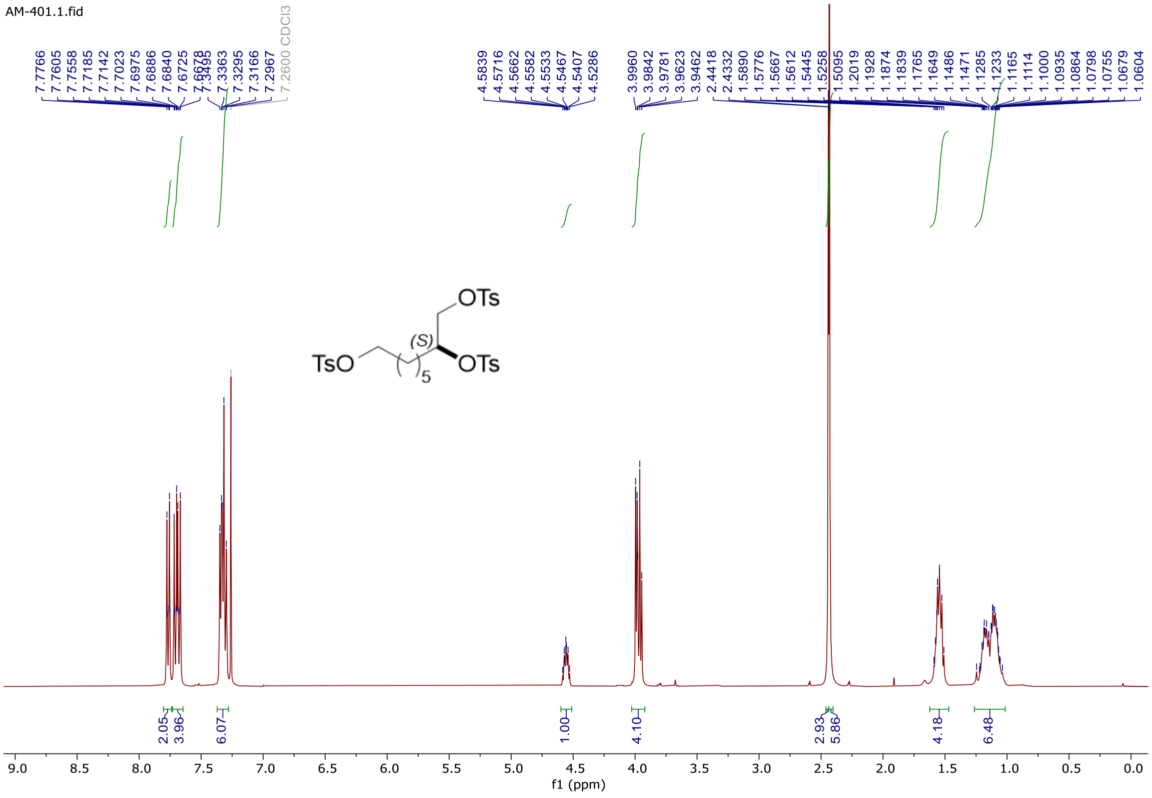

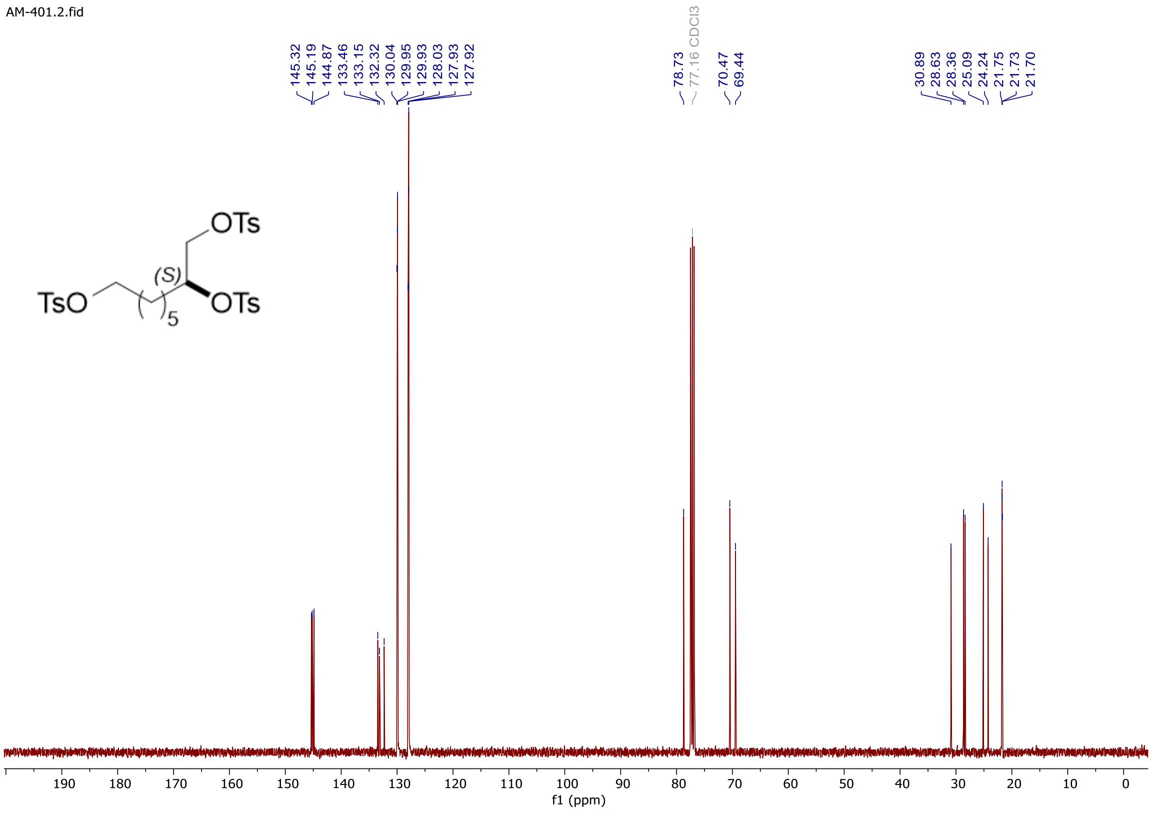

Substrate-3N ($CDCl_3$, ^{1}H NMR: 400 MHz, $^{13}C\{^{1}H\}$ NMR: 101 MHz)

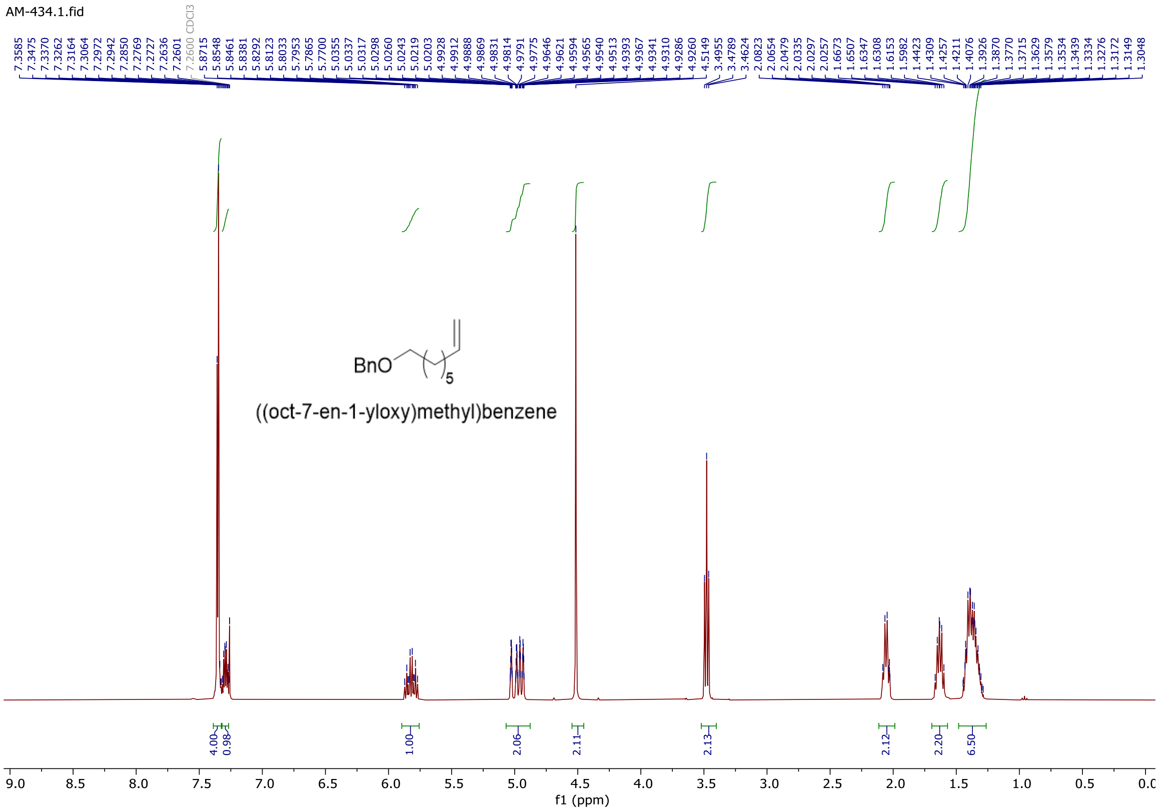

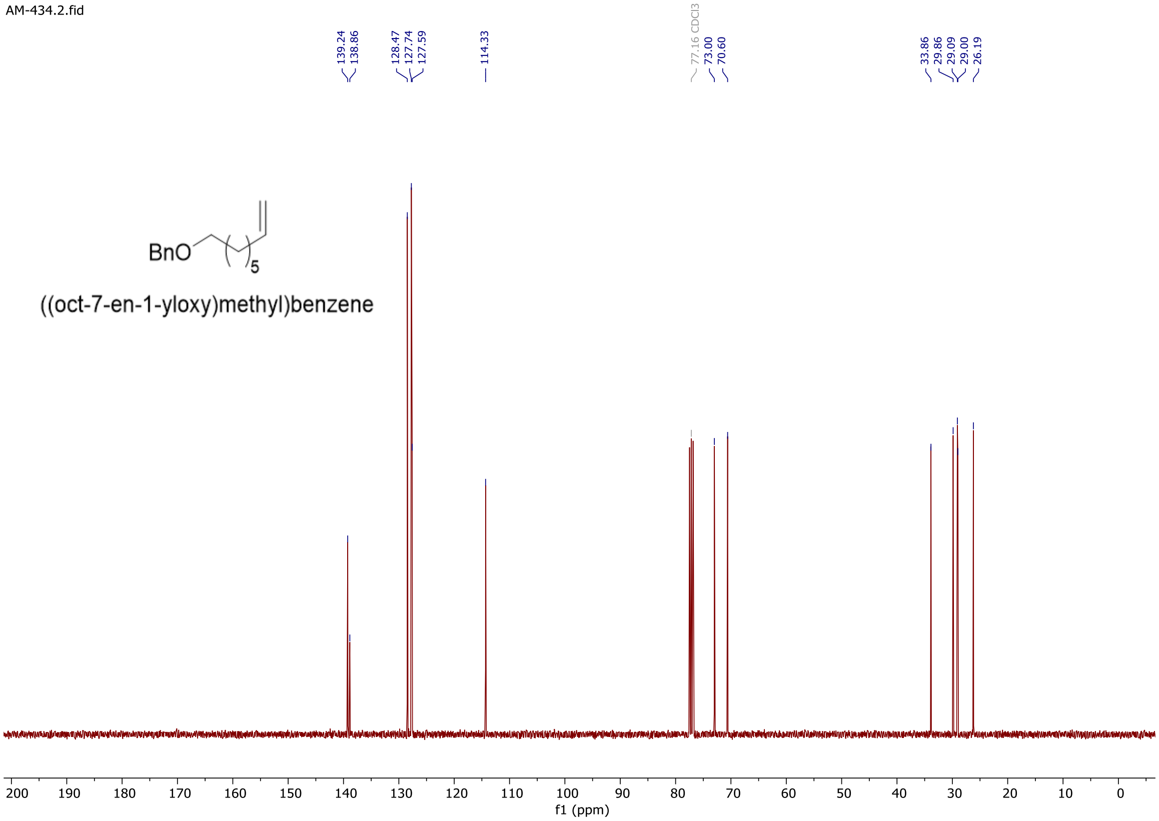

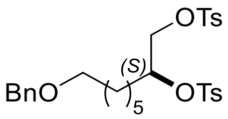

(*S*)-8-(benzyloxy)octane-1,2-diyl bis(4-methylbenzenesulfonate)

Compound 3N: Synthesized using the General Procedure on a 0.2 mmol scale; Purified using a gradient of 2% ethyl acetate in hexane to 20% ethyl acetate in hexane on silica gel; Predominant enantiomer depicted; (colorless oil, 58.1 mg, 0.104 mmol, 52% yield, 91% ee).

^{1}H NMR (400 MHz, $CDCl_3$) δ 7.77 – 7.64 (m, 4H), 7.38 – 7.27 (m, 9H), 4.58 (ddt, *J* = 7.1, 5.7, 4.7 Hz, 1H), 4.49 (s, 2H), 4.09 – 3.98 (m, 2H), 3.47 – 3.38 (m, 2H), 2.45 (s, 3H), 2.43 (s, 3H), 1.64 – 1.49 (m, 4H), 1.28 – 1.07 (m, 6H).

^{13}C{^{1}H} NMR (101 MHz, $CDCl_3$) δ 145.3, 145.1, 138.7, 133.6, 132.5, 130.1, 129.9, 128.5, 128.1, 128.0, 127.8, 127.7, 78.9, 73.0, 70.4, 69.5, 31.1, 29.7, 29.0, 26.0, 24.5, 21.81, 21.79.

IR ν 3054, 2987, 2306, 1599, 1422, 1368, 1266, 1190, 1178, 1098, 896, 738, 705, 667, 555 cm^{-1}.

HRMS (ESI) m/z = $[M + Na]^+$ Calcd $C_{29}H_{36}O_7S_2Na^+$ 583.1800. Found 583.1803 (0.5 ppm error).

Specific Rotation: $[\alpha]_D^{24}$ = –7.1 (c = 2.1 g/100 mL, $CHCl_3$, 91% ee).

Absolute stereochemistry assigned by analogy to an authentic sample of 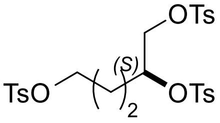 **.**

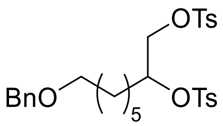

Racemic Sample: HPLC (IC-3, Isopropanol/hexanes = 20/80, flow rate = 1 mL/min, I = 254 nm), t_R = 73.3 min, 80.5 min.

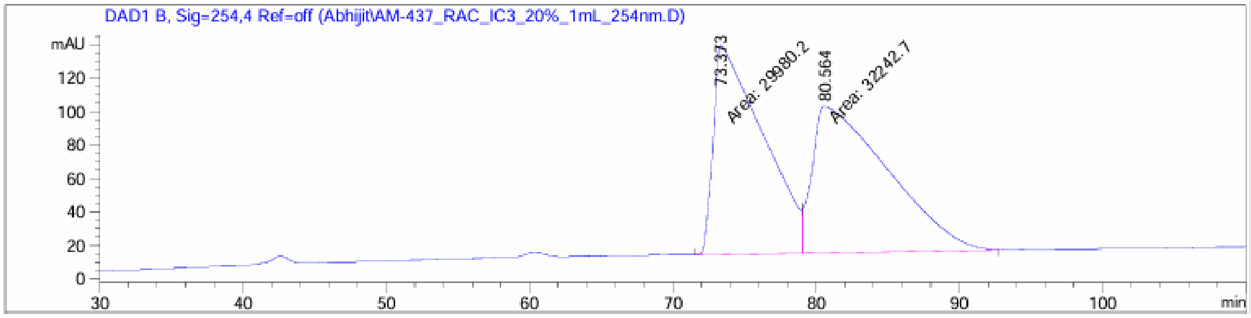

Peak #	RetTime [min]	Type	Width [min]	Area [mAU*s]	Height [mAU]	Area %
1	73.373	MF	3.9976	2.99802e4	124.99129	48.1819
2	80.564	FM	6.1232	3.22427e4	87.76165	51.8181

Scalemic Sample, +91% ee: HPLC (IC-3, Isopropanol/hexanes =20/80, flow rate = 1 mL/min, I = 254 nm), t_R = 69.8 min, 83.2 min.

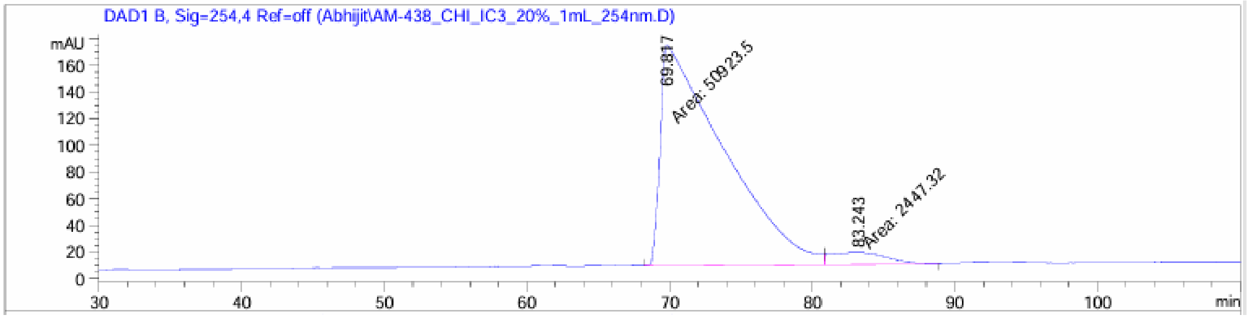

Peak #	RetTime [min]	Type	Width [min]	Area [mAU*s]	Height [mAU]	Area %
1	69.817	MF	5.1487	5.09235e4	164.84117	95.4145
2	83.243	FM	4.6318	2447.31787	8.80615	4.5855

Compound 3N ($CDCl_3$, ^1H NMR: 400 MHz, $^{13}C\{^1H\}$ NMR: 101 MHz)

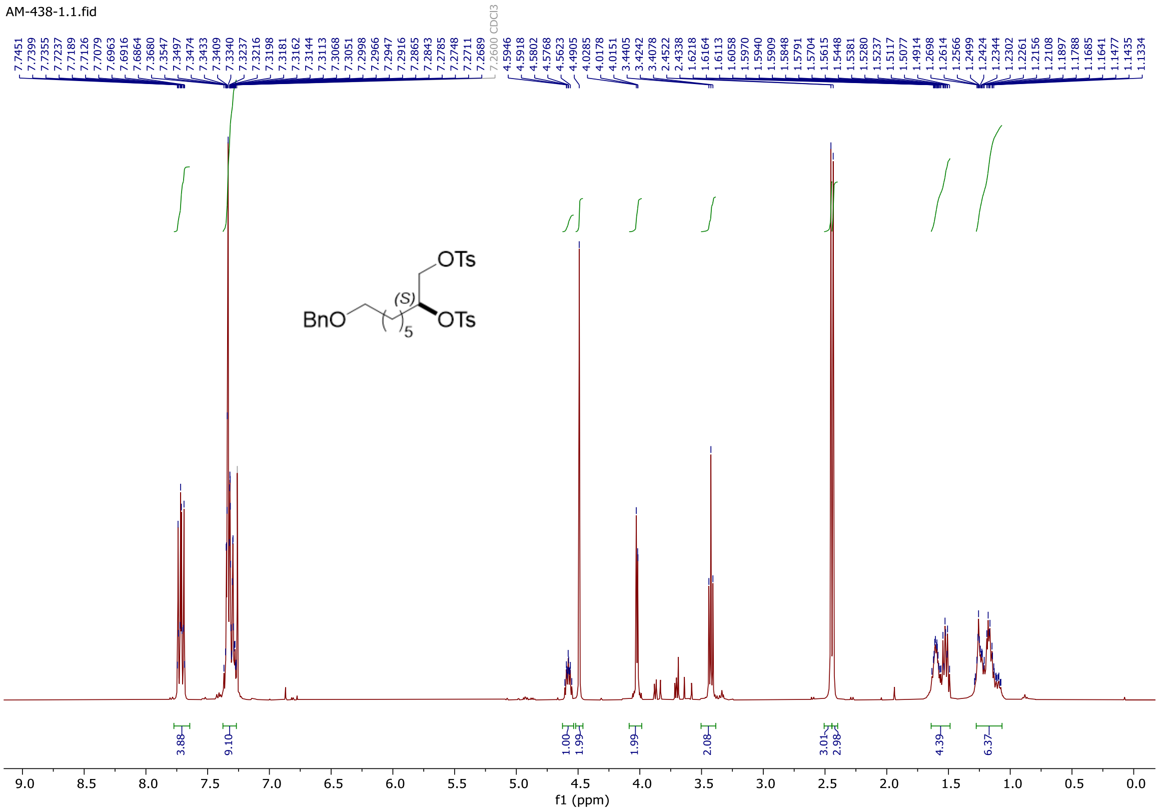

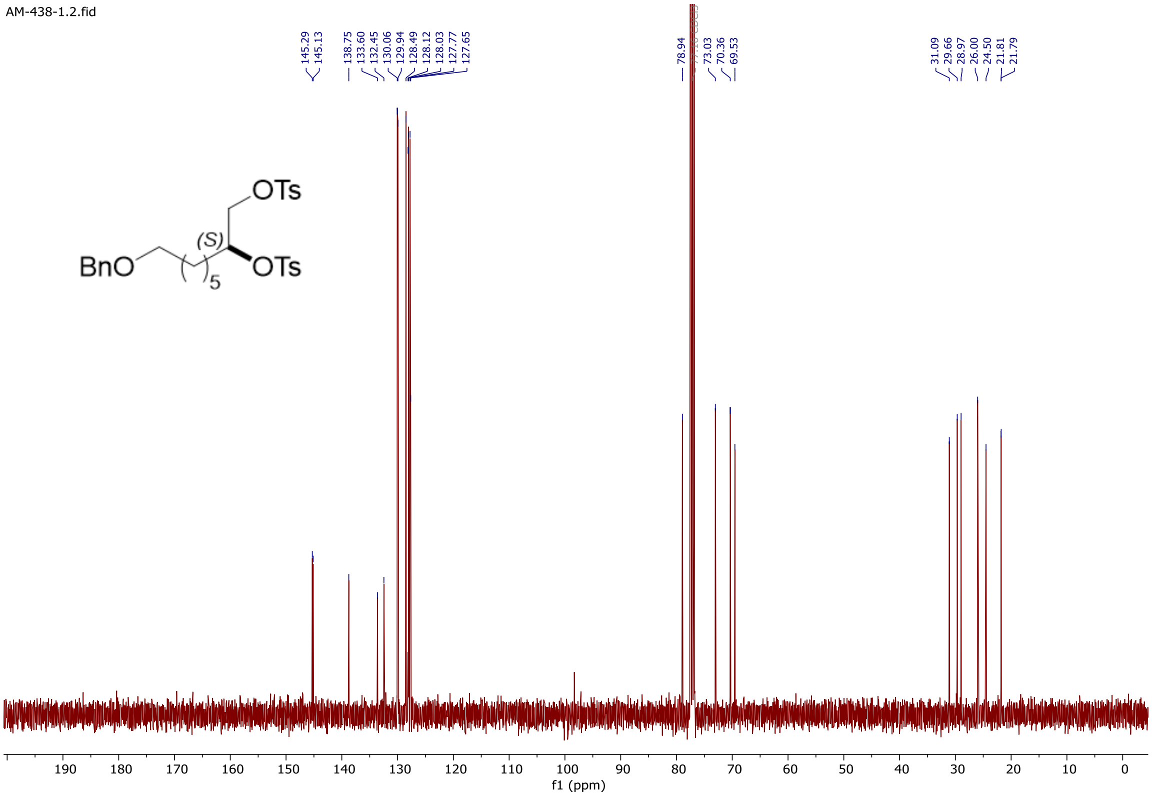

Substrate-3O ($CDCl_3$, ^1H NMR: 400 MHz, $^{13}C\{^1H\}$ NMR: 101 MHz)

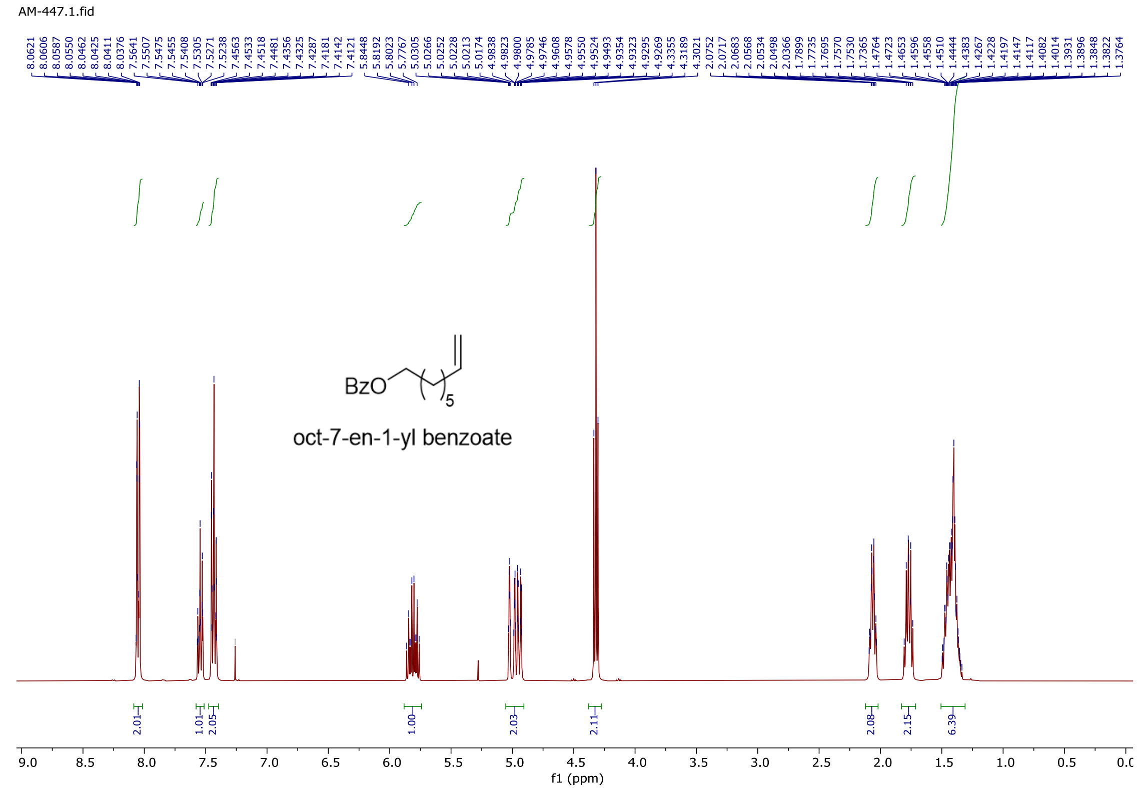

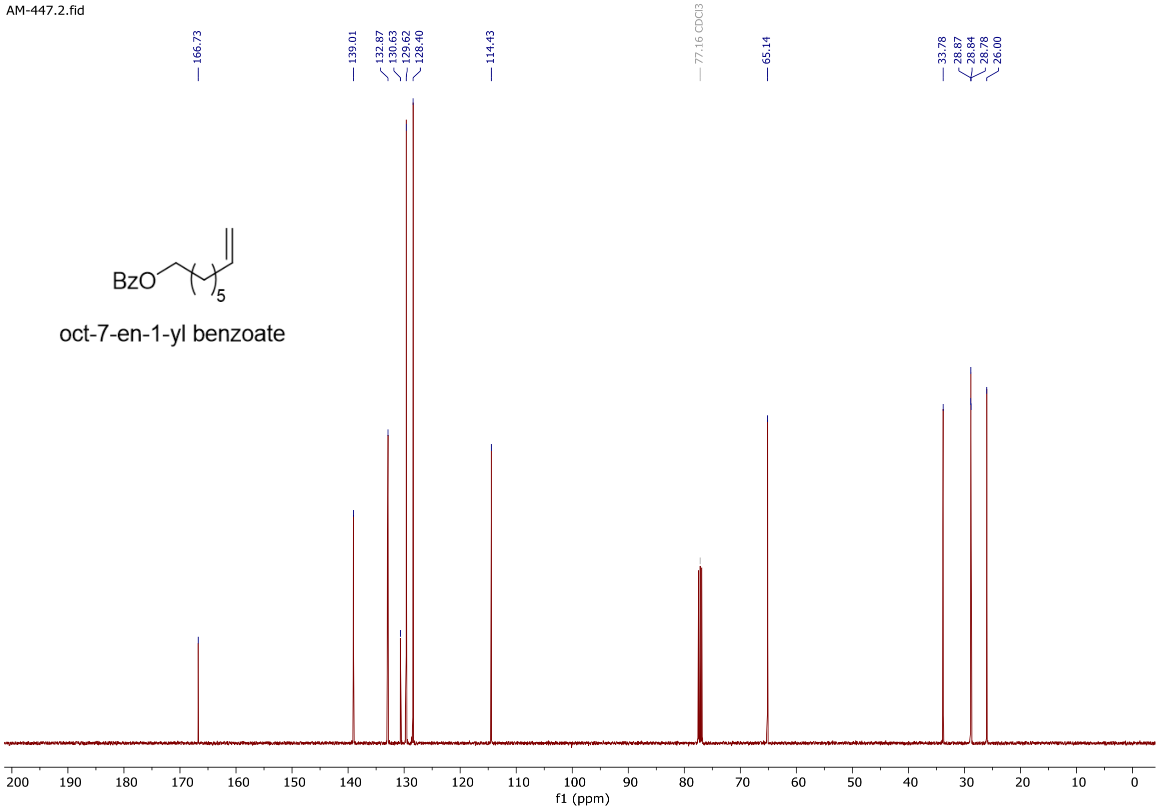

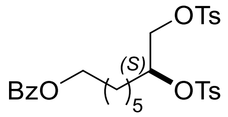

(*S*)-7,8-bis(tosyloxy)octyl benzoate

Compound 3O: Synthesized using the General Procedure on a 0.2 mmol scale; Purified using by reversed-phase HPLC (Redisep Prep C18 column, gradient of 0 to 100% acetonitrile in water); Predominant enantiomer depicted; (colorless oil, 55.5 mg, 0.097 mmol, 48% yield, 85% ee).

^{1}H NMR (400 MHz, $CDCl_3$) δ 8.07 – 8.00 (m, 2H), 7.76 – 7.66 (m, 4H), 7.59 – 7.53 (m, 1H), 7.48 – 7.41 (m, 2H), 7.36 – 7.28 (m, 4H), 4.59 (dq, *J* = 6.8, 4.8 Hz, 1H), 4.32 – 4.21 (m, 2H), 4.09 – 3.97 (m, 2H), 2.44 (s, 3H), 2.43 (s, 3H), 1.73 – 1.57 (m, 4H), 1.38 – 1.29 (m, 2H), 1.28 – 1.14 (m, 4H).

^{13}C{^{1}H} NMR (101 MHz, $CDCl_3$) δ 166.7, 145.3, 145.1, 133.5, 133.0, 132.4, 130.5, 130.0, 129.9, 129.6, 128.5, 128.1, 128.0, 78.8, 69.5, 64.9, 31.0, 28.8, 28.6, 25.8, 24.4, 21.78, 21.75.

IR ν 3054, 2987, 2937, 2306, 1715, 1599, 1422, 1368, 1266, 1190, 1178, 897, 738, 705, 666, 555 cm^{-1}.

HRMS (ESI) m/z = $[M + Na]^+$ Calcd $C_{29}H_{34}O_8S_2Na^+$ 597.1593. Found 597.1597 (0.7 ppm error).

Specific Rotation: $[\alpha]_D^{24}$ = –22.2 (c = 2.7 g/100 mL, $CHCl_3$, 85% ee).

Absolute stereochemistry assigned by analogy to an authentic sample of 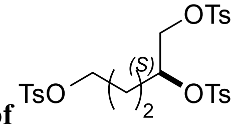

.

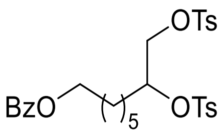

Racemic Sample: HPLC (IC-3, Isopropanol/hexanes = 30/70, flow rate = 1 mL/min, I = 254 nm), t_R = 79.8 min, 92.1 min.

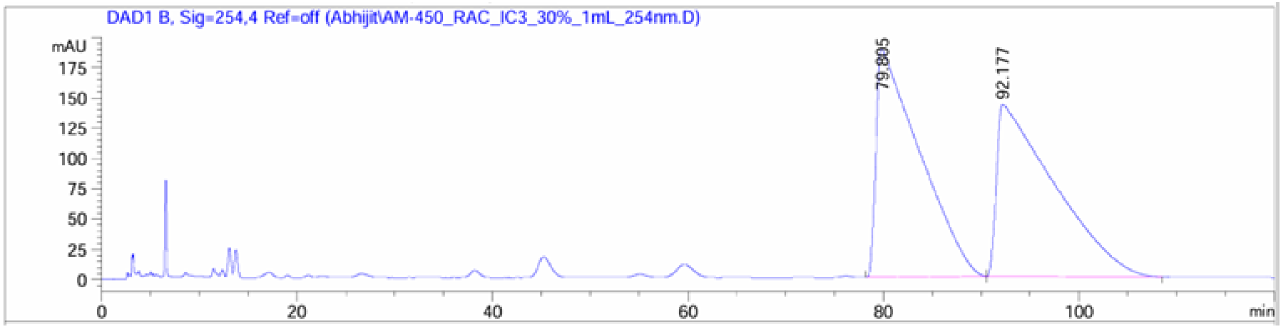

Peak #	RetTime [min]	Type	Width [min]	Area [mAU*s]	Height [mAU]	Area %
1	79.805	BB	3.8031	5.81680e4	188.45151	49.9826
2	92.177	BB	4.9494	5.82086e4	141.73286	50.0174

Scalemic Sample, +85% ee: HPLC (IC-3, Isopropanol/hexanes = 30/70, flow rate = 1 mL/min, I = 254 nm), t_R = 80.8 min, 98.9 min.

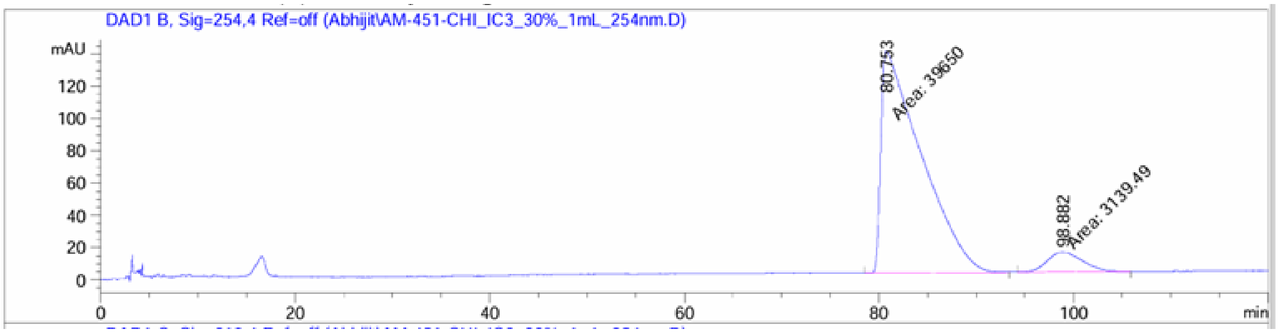

Peak #	RetTime [min]	Type	Width [min]	Area [mAU*s]	Height [mAU]	Area %
1	80.753	MM	4.8074	3.96500e4	137.46054	92.6629
2	98.882	MM	4.2786	3139.49438	12.22931	7.3371

Compound 3O (CDCl$_3$, ^{1}H NMR: 400 MHz, ^{13}C{^{1}H} NMR: 101 MHz)

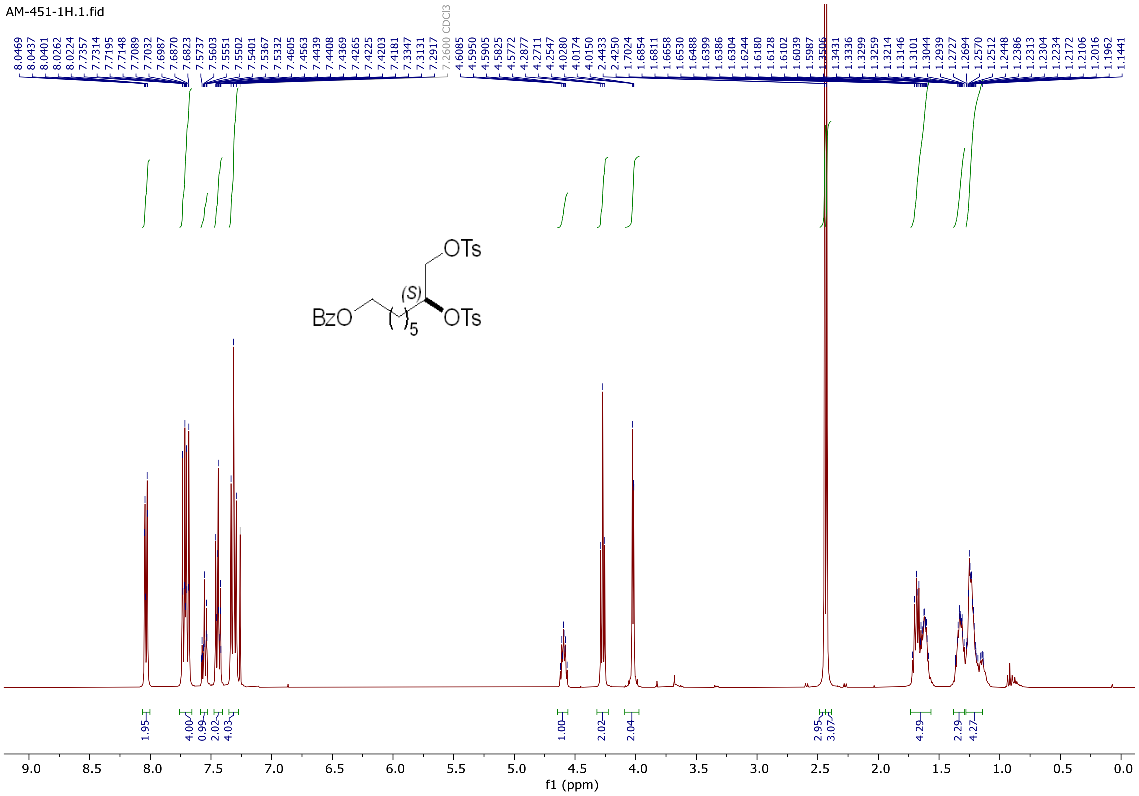

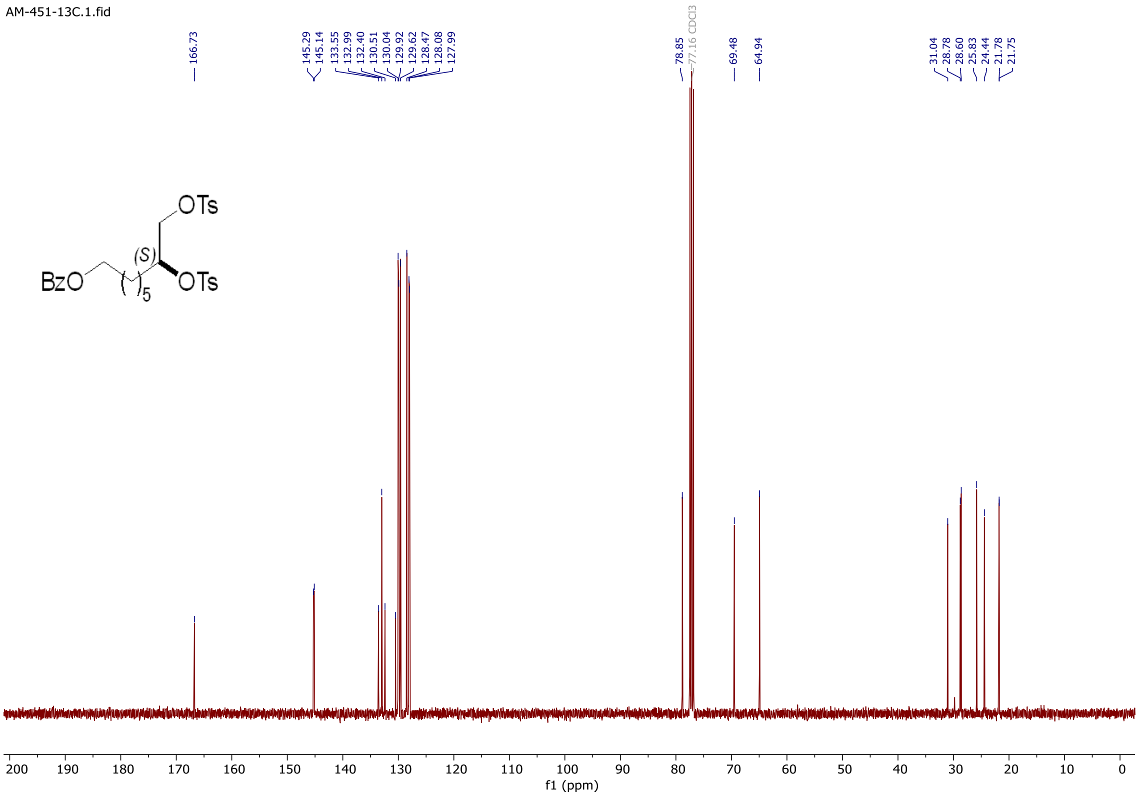

Substrate-3P (CDCl$_3$, ^{1}H NMR: 400 MHz, ^{13}C{^{1}H} NMR: 101 MHz)

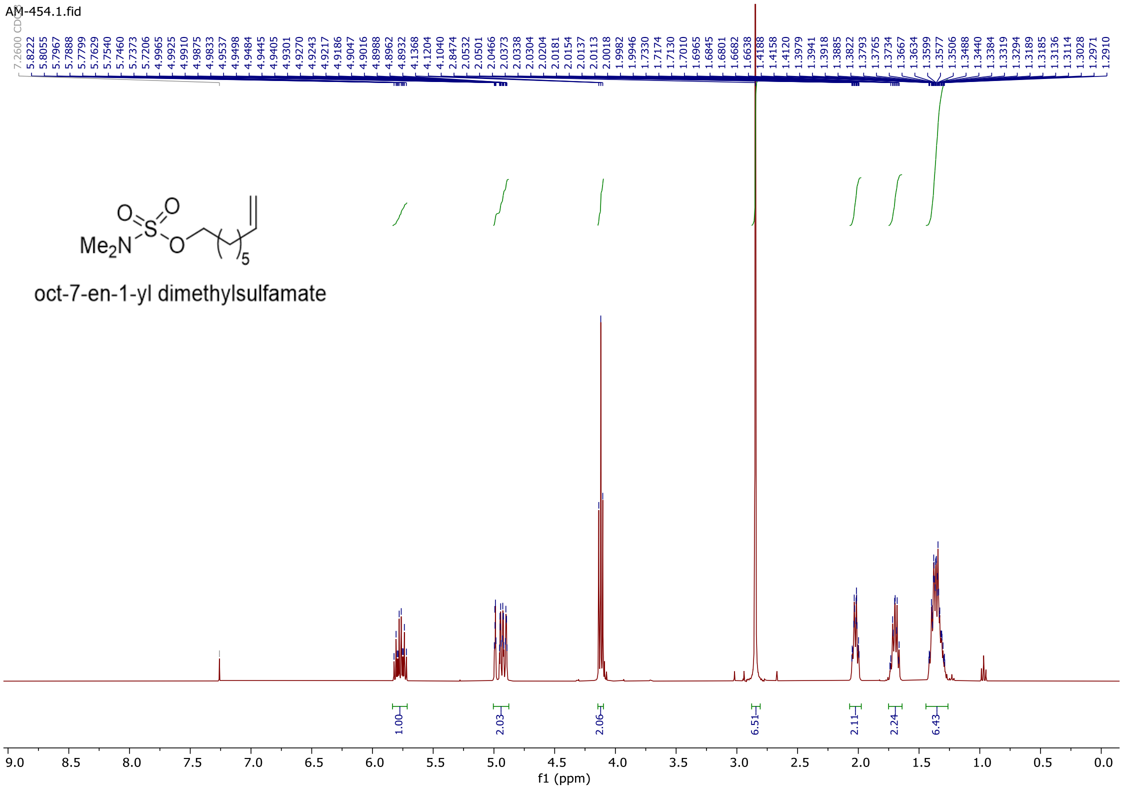

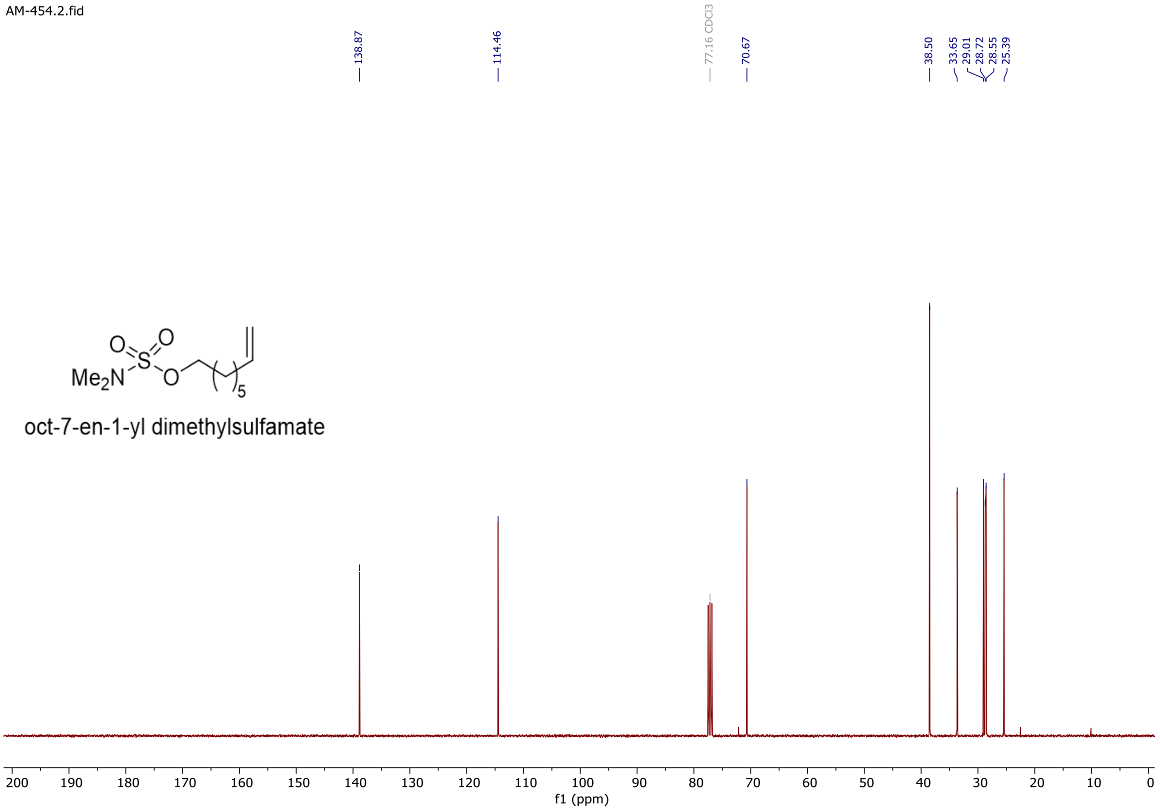

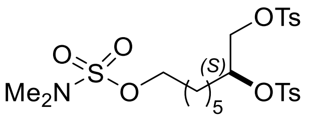

(S)-7,8-bis(tosyloxy)octyl dimethylsulfamate

Compound 3P: Synthesized using the General Procedure on a 0.2 mmol scale; Purified using a gradient of 2% ethyl acetate in hexane to 35% ethyl acetate in hexane on silica gel; Predominant enantiomer depicted; (colorless oil, 77.0 mg, 0.133 mmol, 67% yield, 85% ee).

^{1}H NMR (400 MHz, $CDCl_3$) δ 7.76 – 7.64 (m, 4H), 7.36 – 7.28 (m, 4H), 4.59 (tt, J = 7.0, 4.8 Hz, 1H), 4.13 – 4.07 (m, 2H), 4.03 – 3.96 (m, 2H), 2.86 (s, 6H), 2.45 (s, 3H), 2.44 (s, 3H), 1.69 – 1.55 (m, 4H), 1.32 – 1.11 (m, 6H).

$^{13}C\{^{1}H\}$ NMR (101 MHz, $CDCl_3$) δ 145.3, 145.2, 133.5, 132.4, 130.1, 129.9, 128.1, 128.0, 78.8, 70.5, 69.5, 38.6, 31.0, 28.9, 28.5, 25.2, 24.3, 21.77, 21.76.

IR ν 3056, 2987, 2942, 2306, 1599, 1422, 1366, 1266, 1190, 1178, 1096, 947, 916, 897, 739, 705 cm^{-1}.

HRMS (ESI) m/z = $[M + Na]^+$ Calcd $C_{24}H_{35}NO_9S_3Na^+$ 600.1372. Found 600.1378 (1.0 ppm error).

Specific Rotation: $[\alpha]_D^{24}$ = –19.1 (c = 3.5 g/100 mL, $CHCl_3$, 85% ee).

Absolute stereochemistry assigned by analogy to an authentic sample of 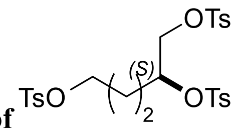

.

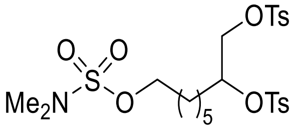

Racemic Sample: HPLC (IC-3, Isopropanol/hexanes = 50/50, flow rate = 1 mL/min, I = 220 nm), t_R = 111.5 min, 129.3 min.

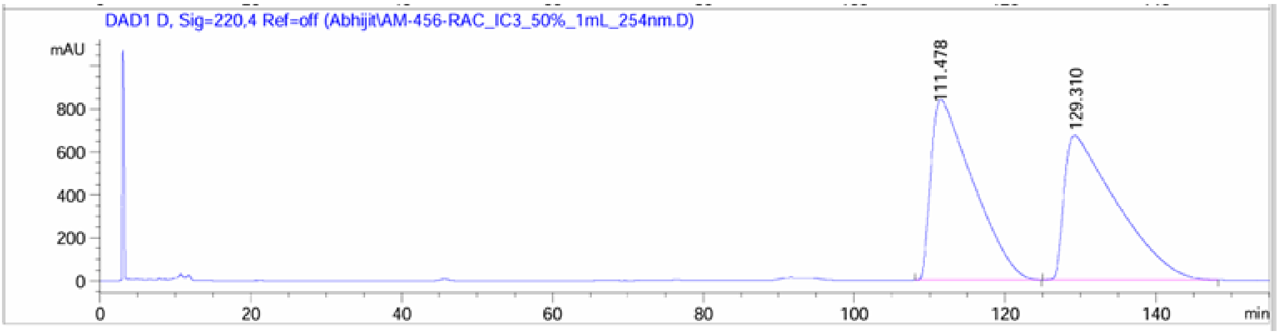

Peak #	RetTime [min]	Type	Width [min]	Area [mAU*s]	Height [mAU]	Area %
1	111.478	BB	4.5450	3.26436e5	841.25793	49.9781
2	129.310	BB	5.6934	3.26722e5	672.81494	50.0219

Scalemic Sample, +85% ee: HPLC (IC-3, Isopropanol/hexanes = 50/50, flow rate = 1 mL/min, I = 220 nm), t_R = 110.4 min, 141.3 min.

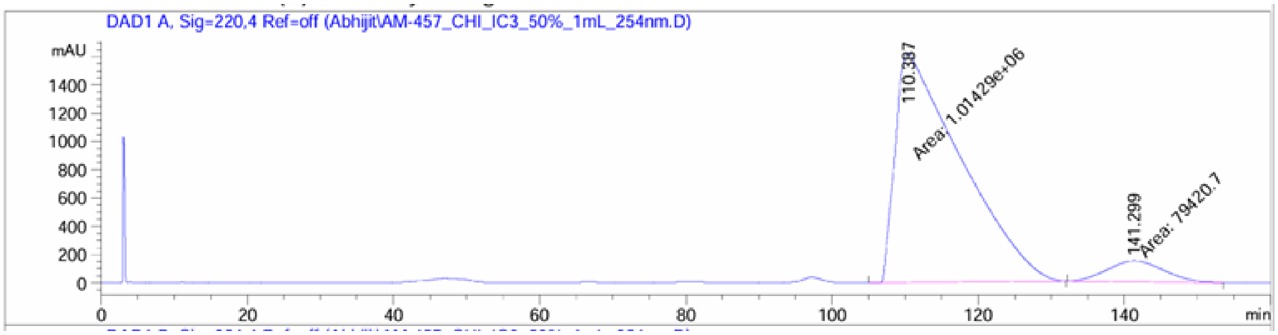

Peak #	RetTime [min]	Type	Width [min]	Area [mAU*s]	Height [mAU]	Area %
1	110.387	MM	10.4177	1.01429e6	1622.70483	92.7384
2	141.299	MM	8.9014	7.94207e4	148.70401	7.2616

Compound 3P ($CDCl_3$, ^1H NMR: 400 MHz, $^{13}C\{^1H\}$ NMR: 101 MHz)

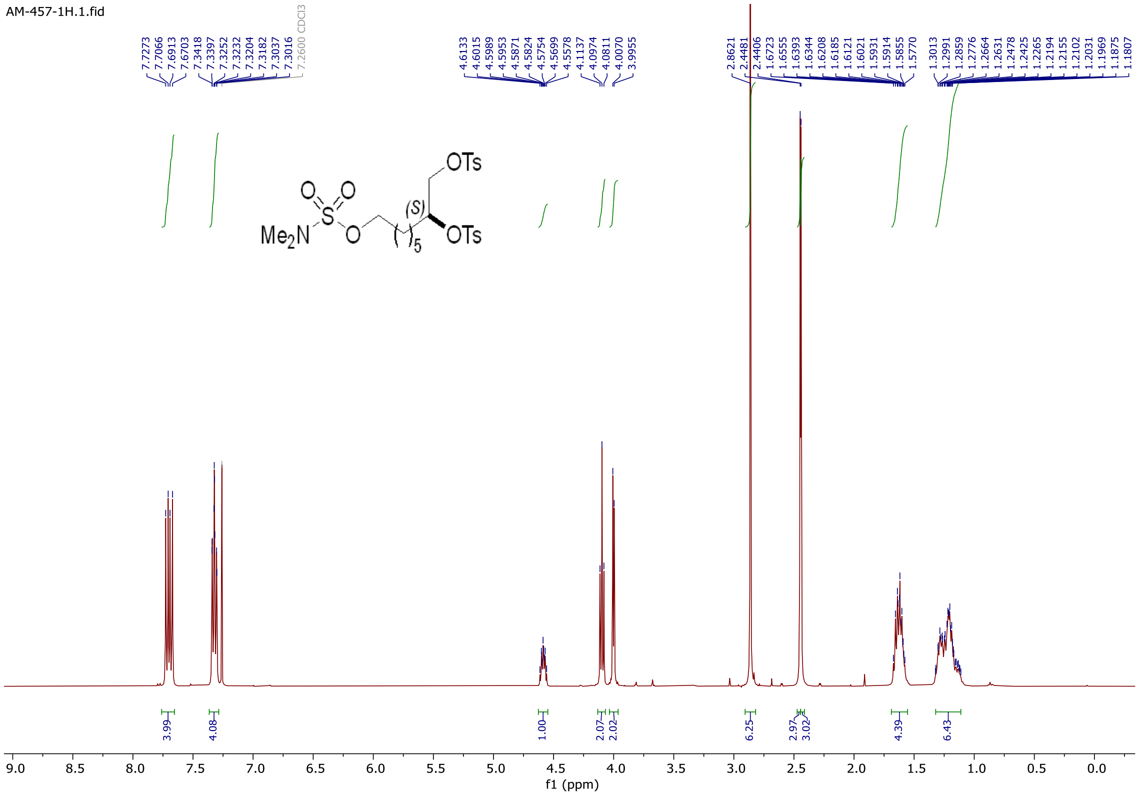

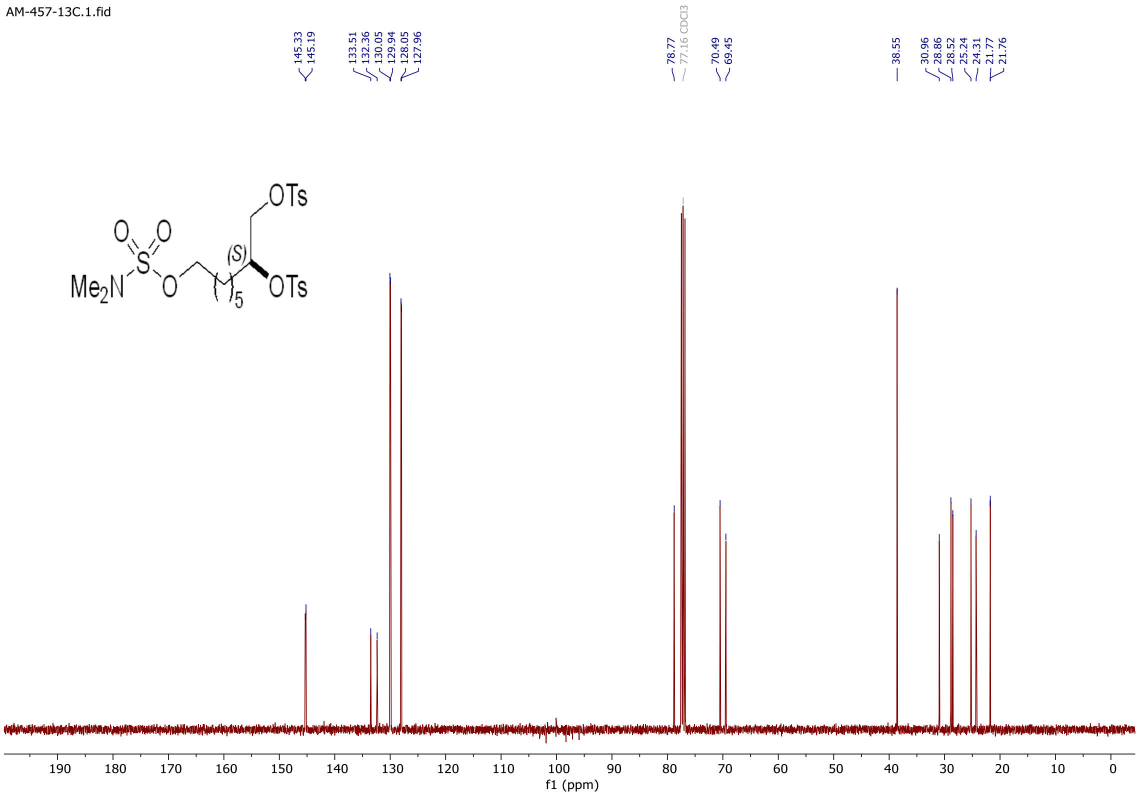

Substrate-3Q (CDCl₃, ^{1}H NMR: 400 MHz, $^{13}C\{^{1}H\}$ NMR: 101 MHz)

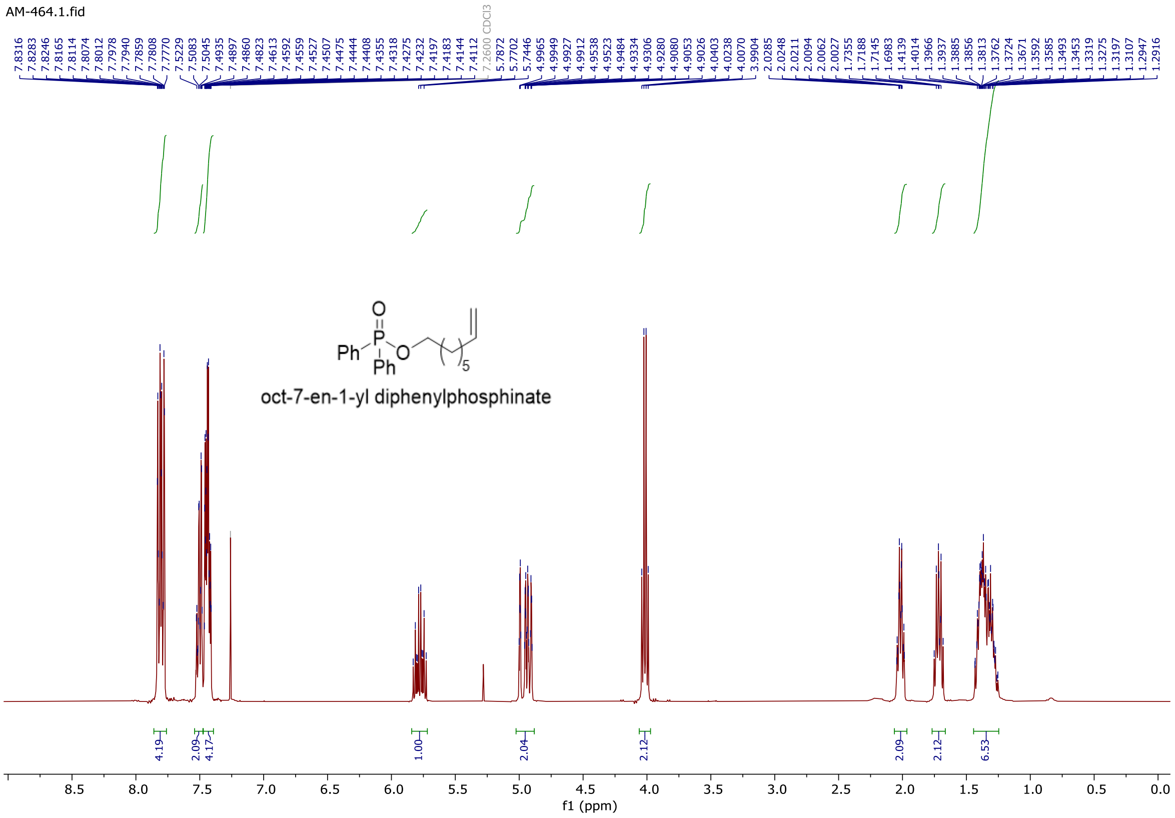

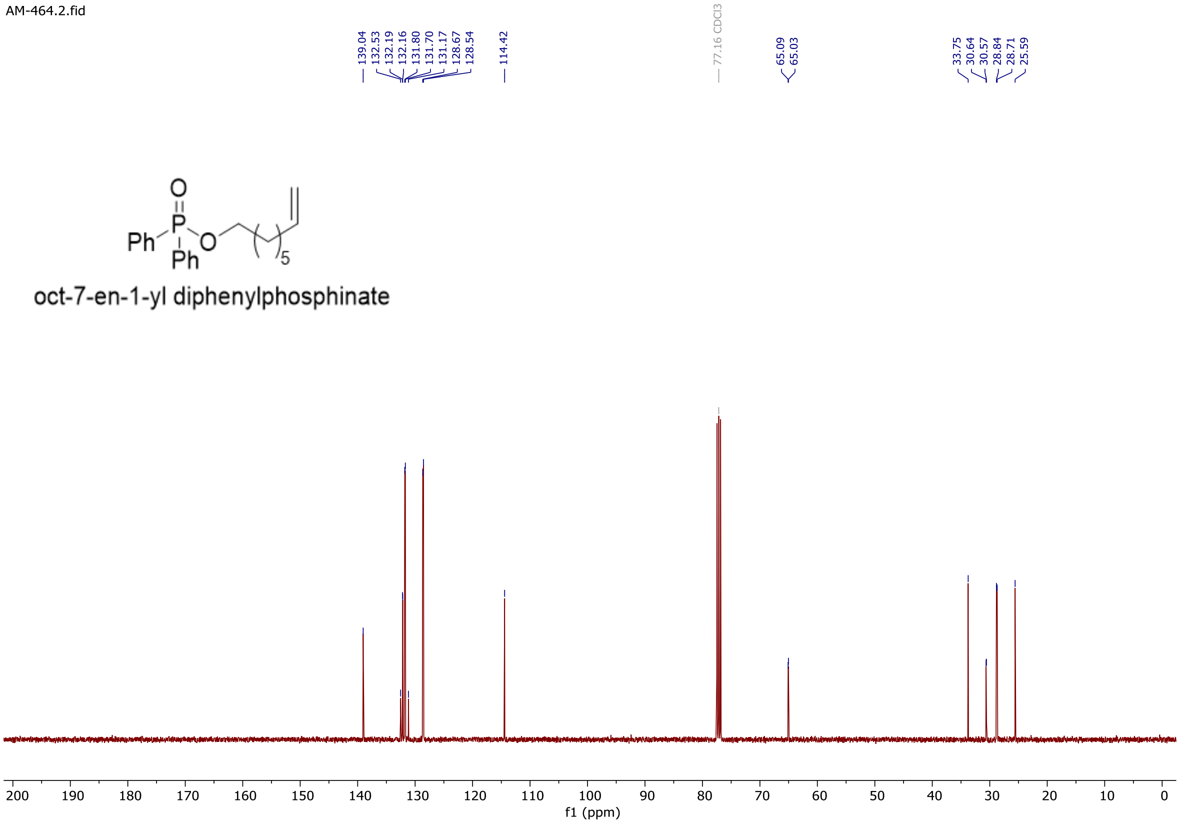

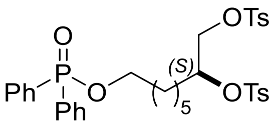

(*S*)-8-((diphenylphosphoryl)oxy)octane-1,2-diyl bis(4-methylbenzenesulfonate)

Compound 3Q: Synthesized using the General Procedure on a 0.2 mmol scale; Purified using a gradient of 5% ethyl acetate in hexane to 50% ethyl acetate in hexane on silica gel; Predominant enantiomer depicted; (colorless oil, 82.2 mg, 0.122 mmol, 61% yield, 86% ee).

^{1}H NMR (400 MHz, $CDCl_3$) δ 7.85 – 7.74 (m, 4H), 7.72 – 7.63 (m, 4H), 7.54 – 7.47 (m, 2H), 7.47 – 7.39 (m, 4H), 7.34 – 7.27 (m, 4H), 4.56 (dt, *J* = 7.0, 5.1 Hz, 1H), 4.04 – 3.91 (m, 4H), 2.42 (s, 3H), 2.40 (s, 3H), 1.68 – 1.52 (m, 4H), 1.32 – 1.09 (m, 6H).

^{13}C{^{1}H} NMR (101 MHz, $CDCl_3$) δ 145.3, 145.1, 133.5, 132.4 (d, *J* = 136.4 Hz), 132.2 (d, *J* = 3.0 Hz), 131.7 (d, *J* = 10.1 Hz), 130.0, 129.9, 128.7 (d, *J* = 13.1 Hz), 128.0, 127.9, 78.8, 69.4, 64.8 (d, *J* = 6.1 Hz), 31.0, 30.4 (d, *J* = 7.1 Hz), 28.6, 25.4, 24.3, 21.74, 21.72.

IR ν 3054, 2985, 2937, 2306, 1597, 1440, 1422, 1368, 1266, 1190, 1178, 1131, 1097, 995, 916, 897 cm^{-1}.

HRMS (ESI) m/z = $[M + Na]^+$ Calcd $C_{34}H_{39}PO_8S_2Na^+$ 693.1722. Found 693.1743 (3.0 ppm error).

Specific Rotation: $[\alpha]_D^{22}$ = +12.0 (c = 3.6 g/100 mL, $CHCl_3$, 86% ee).

Absolute stereochemistry assigned by analogy to an authentic sample of 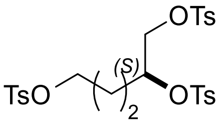 **.**

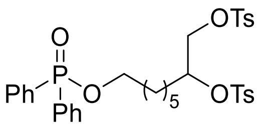

Racemic Sample: HPLC (IC-3, Isopropanol/hexanes = 50/50, flow rate = 1 mL/min, I = 254 nm), t_R = 147.1 min, 164.5 min.

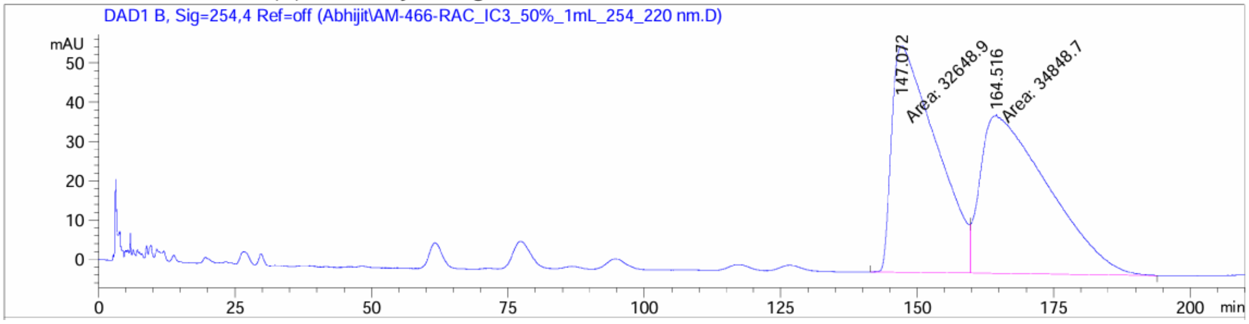

Peak #	RetTime [min]	Type	Width [min]	Area [mAU*s]	Height [mAU]	Area %
1	147.072	MF	9.4484	3.26489e4	57.59166	48.3705
2	164.516	FM	14.3709	3.48487e4	40.41586	51.6295

Scalemic Sample, +86% ee: HPLC (IC-3, Isopropanol/hexanes = 50/50, flow rate = 1 mL/min, I = 254 nm), t_R = 144.8 min, 176.4 min.

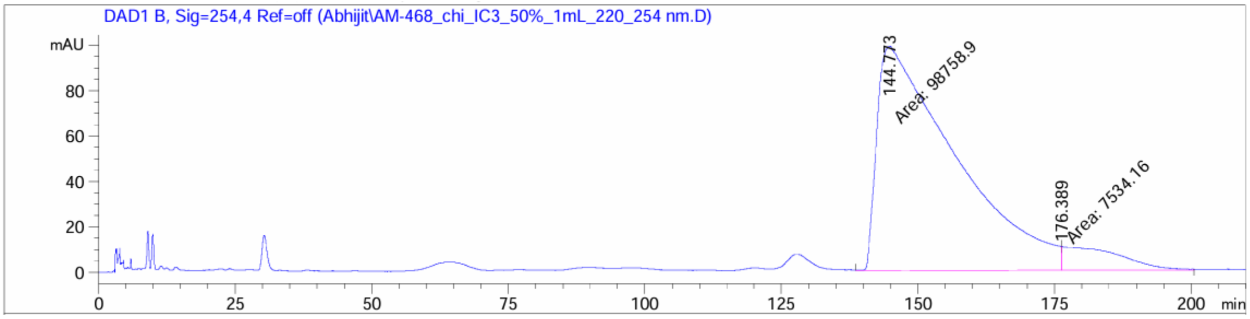

Peak #	RetTime [min]	Type	Width [min]	Area [mAU*s]	Height [mAU]	Area %
1	144.773	MF	16.6350	9.87589e4	98.94685	92.9119
2	176.389	FM	12.0923	7534.15771	10.38422	7.0881

Compound 3Q ($CDCl_3$, ^1H NMR: 400 MHz, $^{13}C\{^1H\}$ NMR: 101 MHz)

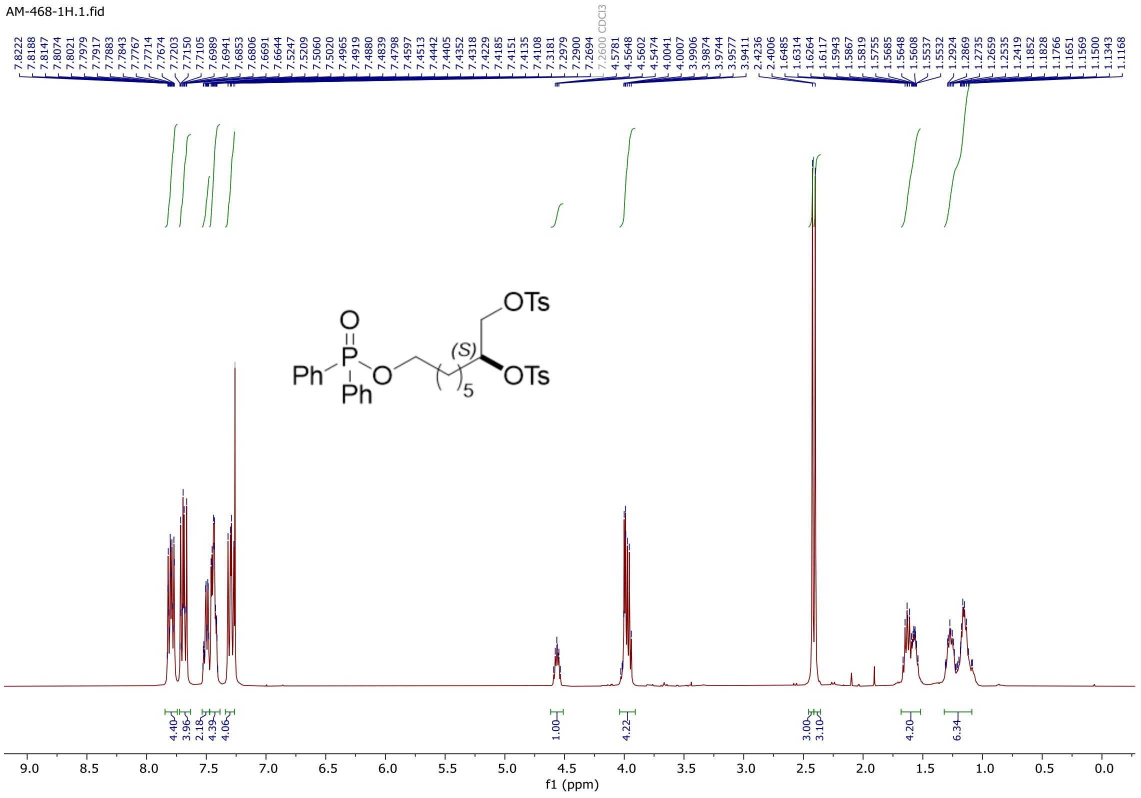

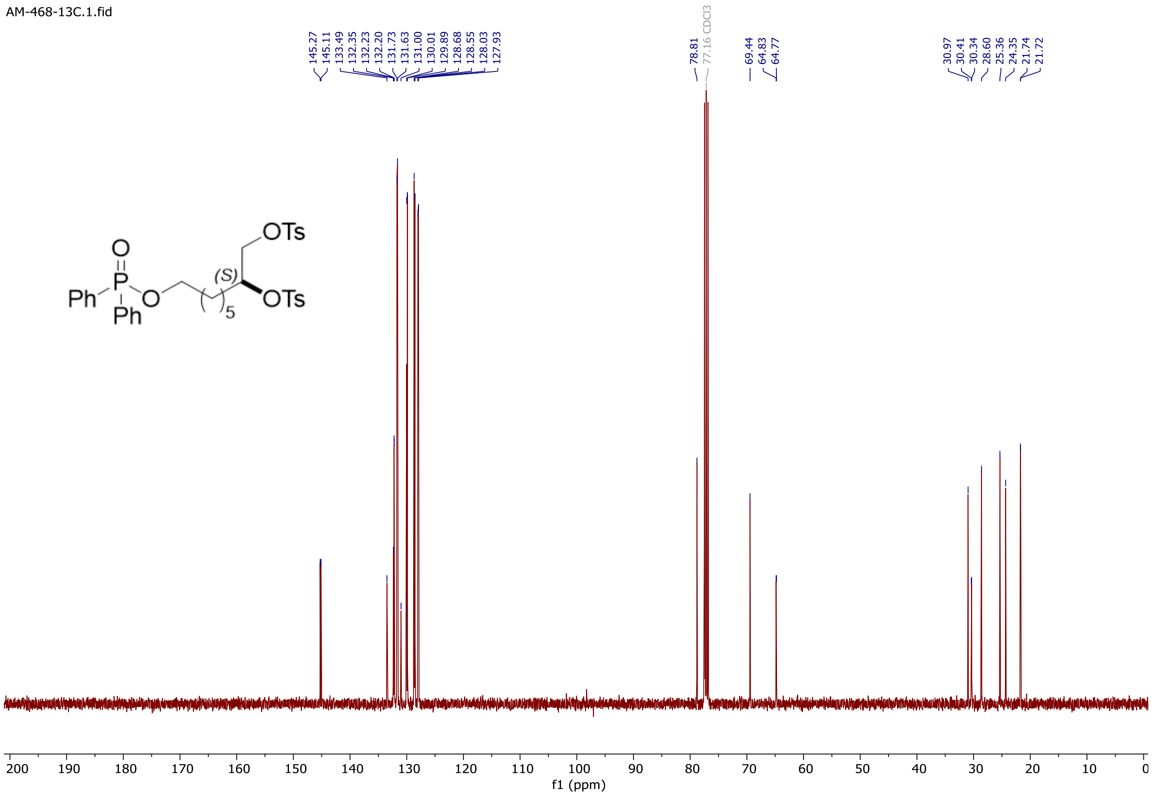

Substrate-3R ($CDCl_3$, ^{1}H NMR: 400 MHz, $^{13}C\{^{1}H\}$ NMR: 101 MHz)

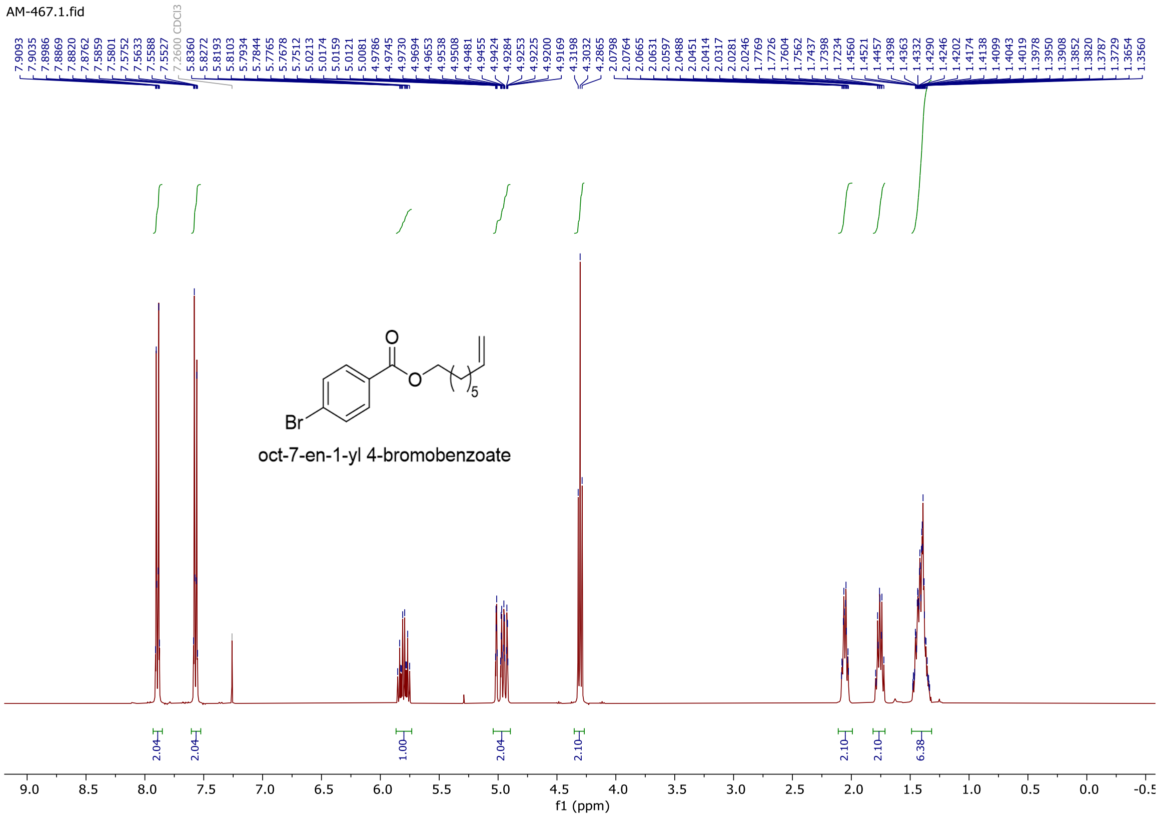

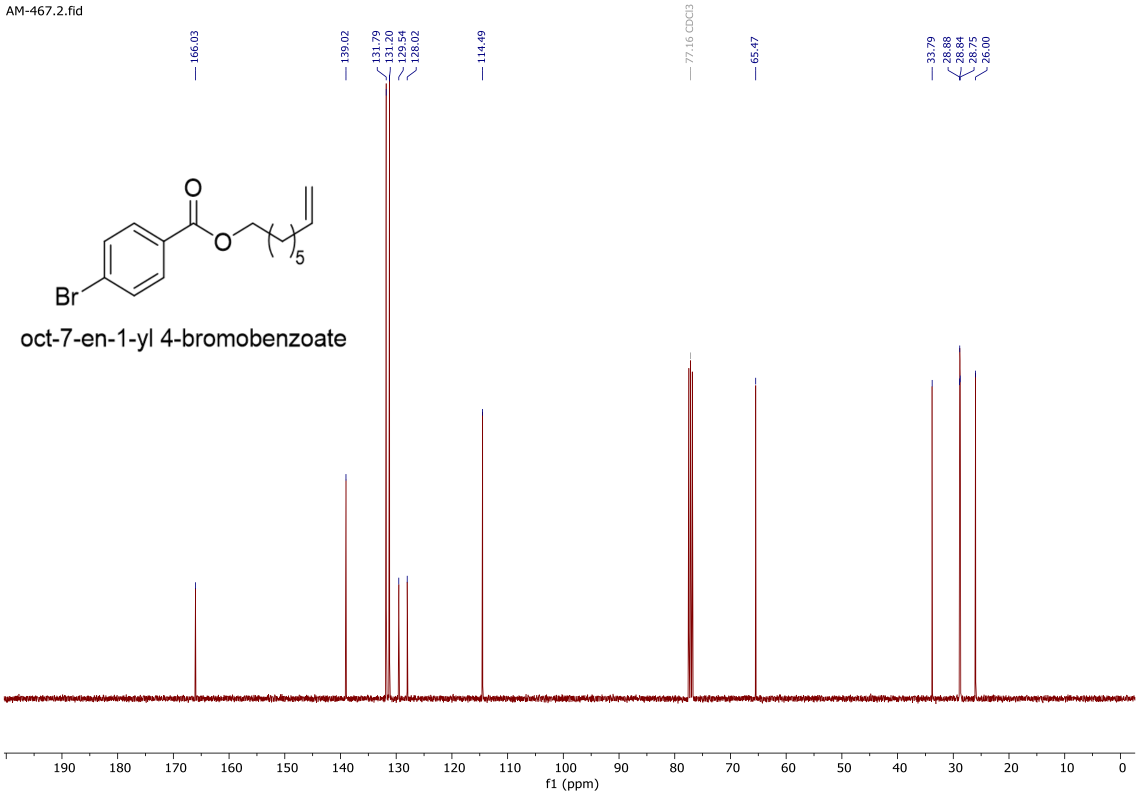

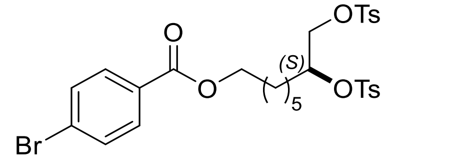

(*S*)-7,8-bis(tosyloxy)octyl 4-bromobenzoate

Compound 3R: Synthesized using the General Procedure on a 0.2 mmol scale; Purified using reversed-phase HPLC (Redisep Prep C18 column, gradient of 0 to 100% acetonitrile in water); Predominant enantiomer depicted; (colorless oil, 85.0 mg, 0.130 mmol, 65% yield, 84% ee).

^{1}H NMR (400 MHz, $CDCl_3$) δ 7.93 – 7.84 (m, 2H), 7.75 – 7.64 (m, 4H), 7.61 – 7.54 (m, 2H), 7.36 – 7.27 (m, 4H), 4.60 (tt, *J* = 7.0, 4.8 Hz, 1H), 4.30 – 4.20 (m, 2H), 4.06 – 3.96 (m, 2H), 2.44 (s, 3H), 2.42 (s, 3H), 1.73 – 1.57 (m, 4H), 1.33 – 1.17 (m, 6H).

^{13}C{^{1}H} NMR (101 MHz, $CDCl_3$) δ 166.0, 145.3, 145.1, 133.5, 132.4, 131.8, 131.2, 130.0, 129.9, 129.4, 128.1, 128.0, 78.8, 69.4, 65.2, 31.0, 28.7, 28.5, 25.8, 24.4, 21.8, 21.7.

IR ν 3054, 2987, 2306, 1717, 1592, 1422, 1368, 1266, 1190, 1178, 1104, 1013, 896, 815, 742 cm^{-1}.

HRMS (ESI) m/z = $[M + Na]^+$ Calcd $C_{29}H_{33}BrO_8S_2Na^+$ 675.0698. Found 675.0697 (0.1 ppm error).

Specific Rotation: $[\alpha]_D^{24}$ = –7.4 (c = 3.9 g/100 mL, $CHCl_3$, 84% ee).

Absolute stereochemistry assigned by analogy to an authentic sample of 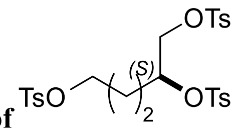 **.**

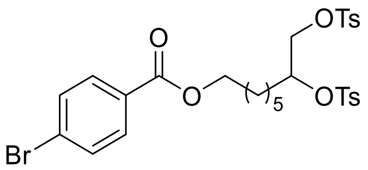

Racemic Sample: HPLC (IC-3, Isopropanol/hexanes = 30/70, flow rate = 1 mL/min, l = 254 nm), t_R = 72.1 min, 82.7 min.

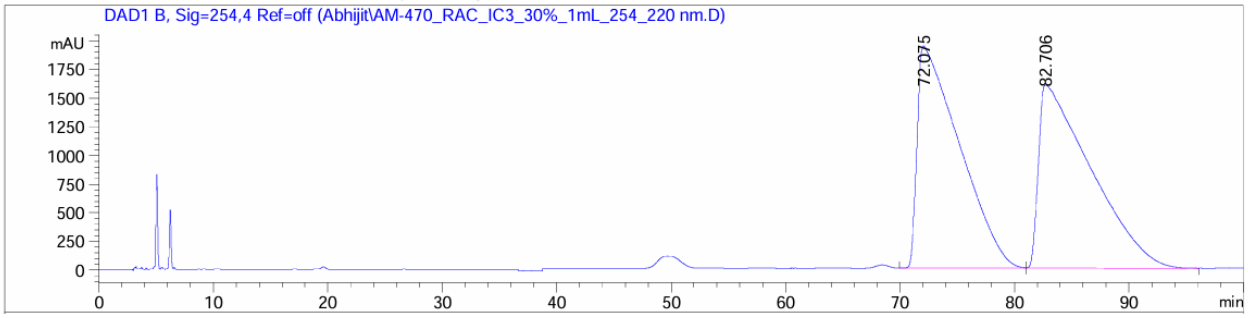

Peak #	RetTime [min]	Type	Width [min]	Area [mAU*s]	Height [mAU]	Area %
1	72.075	BB	3.0125	4.95757e5	1936.66528	49.4608
2	82.706	BB	3.8726	5.06566e5	1594.42725	50.5392

Scalemic Sample, +84% ee: HPLC (IC-3, Isopropanol/hexanes = 30/70, flow rate = 1 mL/min, l = 254 nm), t_R = 70.5 min, 86.4 min.

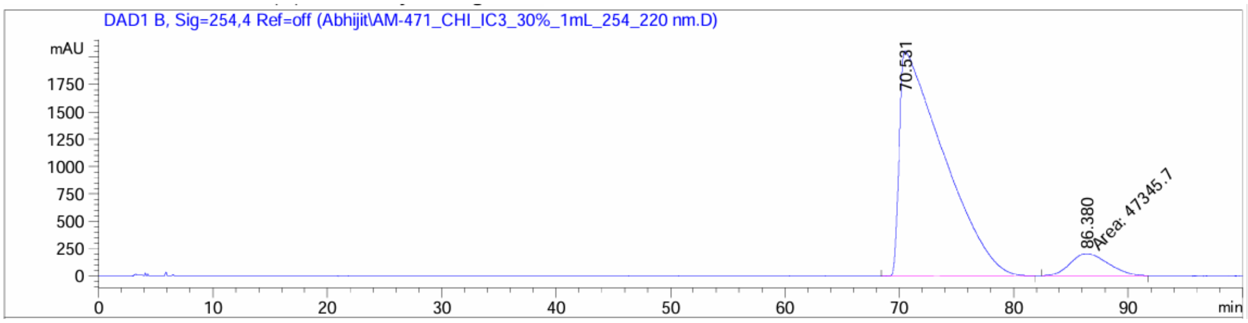

Peak #	RetTime [min]	Type	Width [min]	Area [mAU*s]	Height [mAU]	Area %
1	70.531	BB	3.1548	5.53312e5	2056.64087	92.1177
2	86.380	MM	3.8710	4.73457e4	203.84985	7.8823

Compound 3R (CDCl₃, ^{1}H NMR: 400 MHz, $^{13}C\{^{1}H\}$ NMR: 101 MHz)

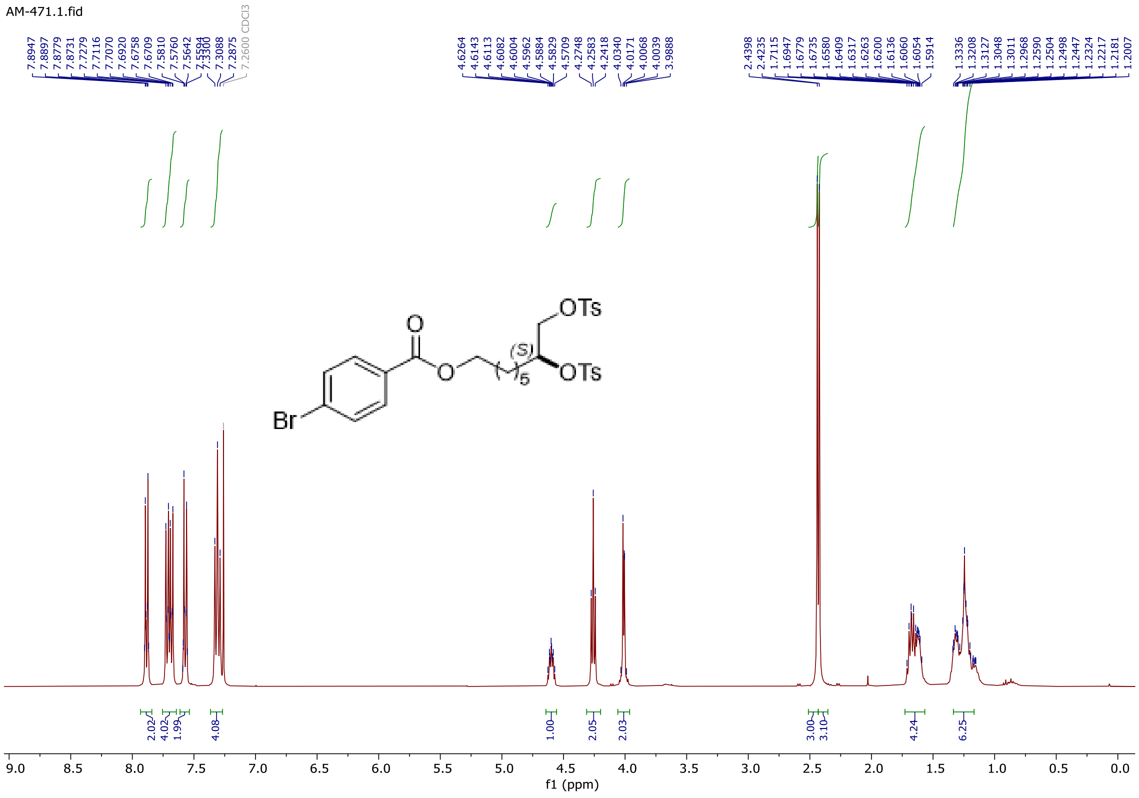

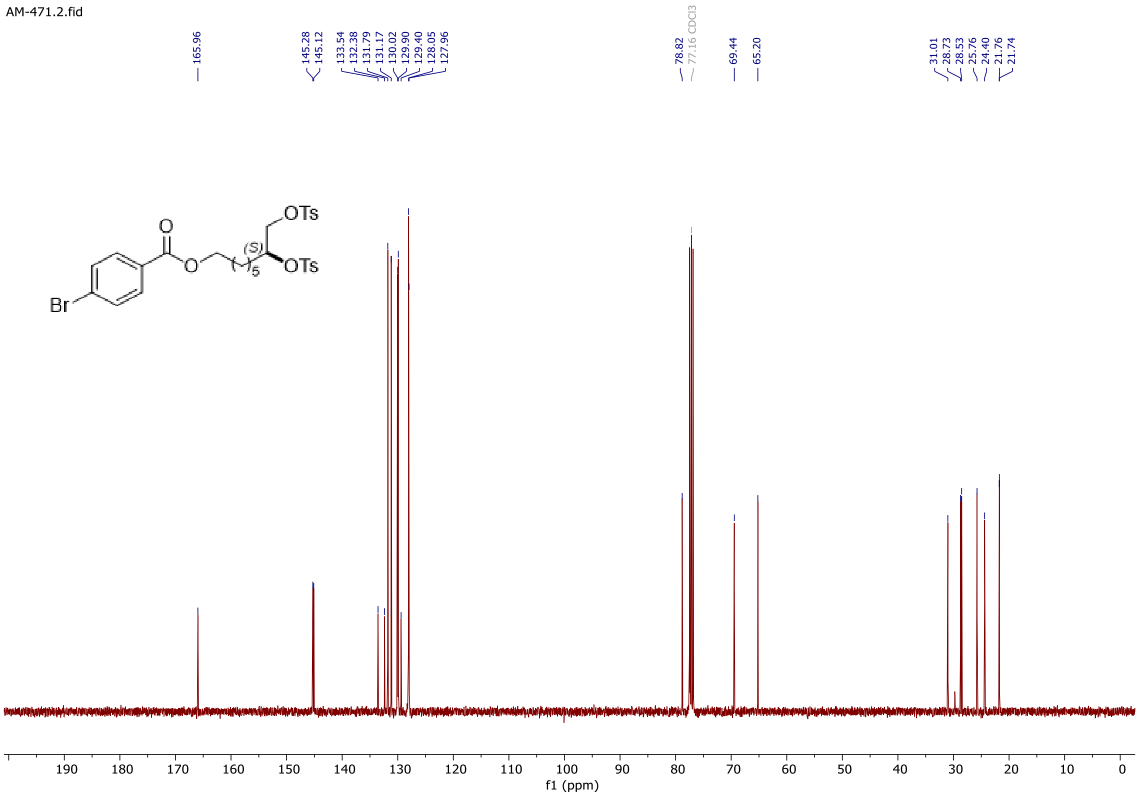

Substrate-3S ($CDCl_3$, ^{1}H NMR: 400 MHz, $^{13}C\{^1H\}$ NMR: 101 MHz)

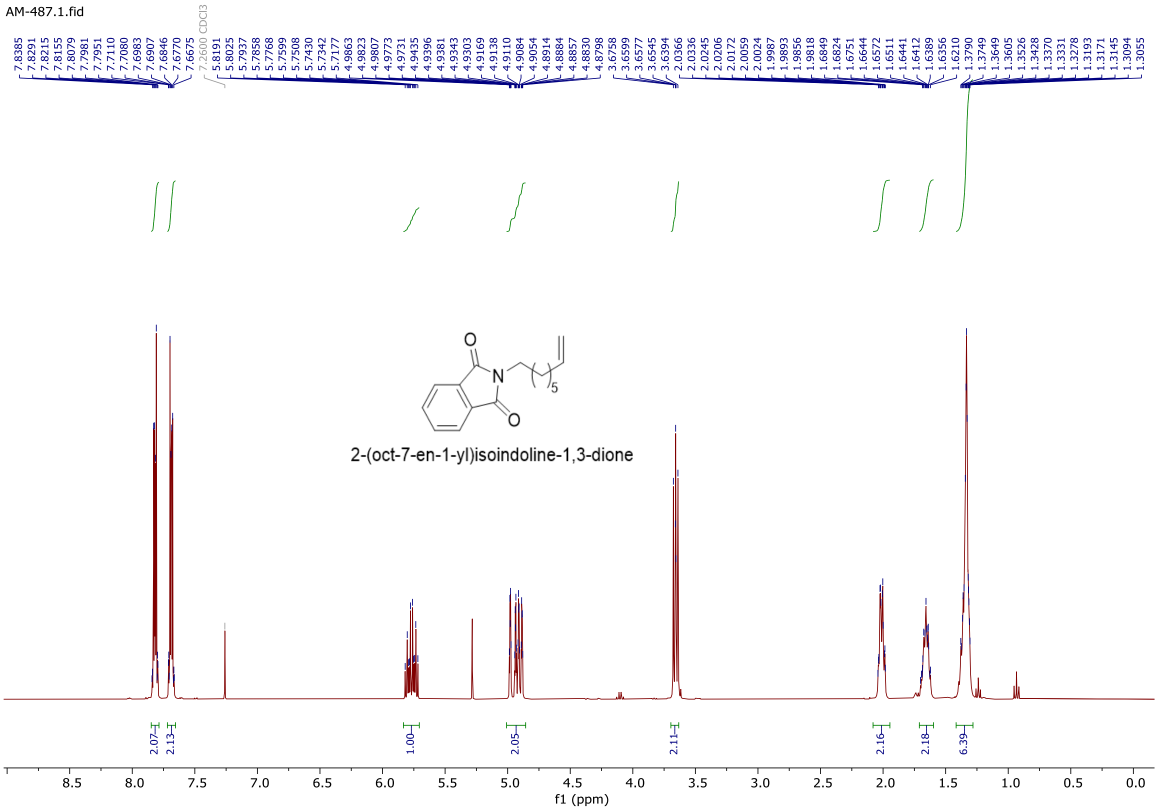

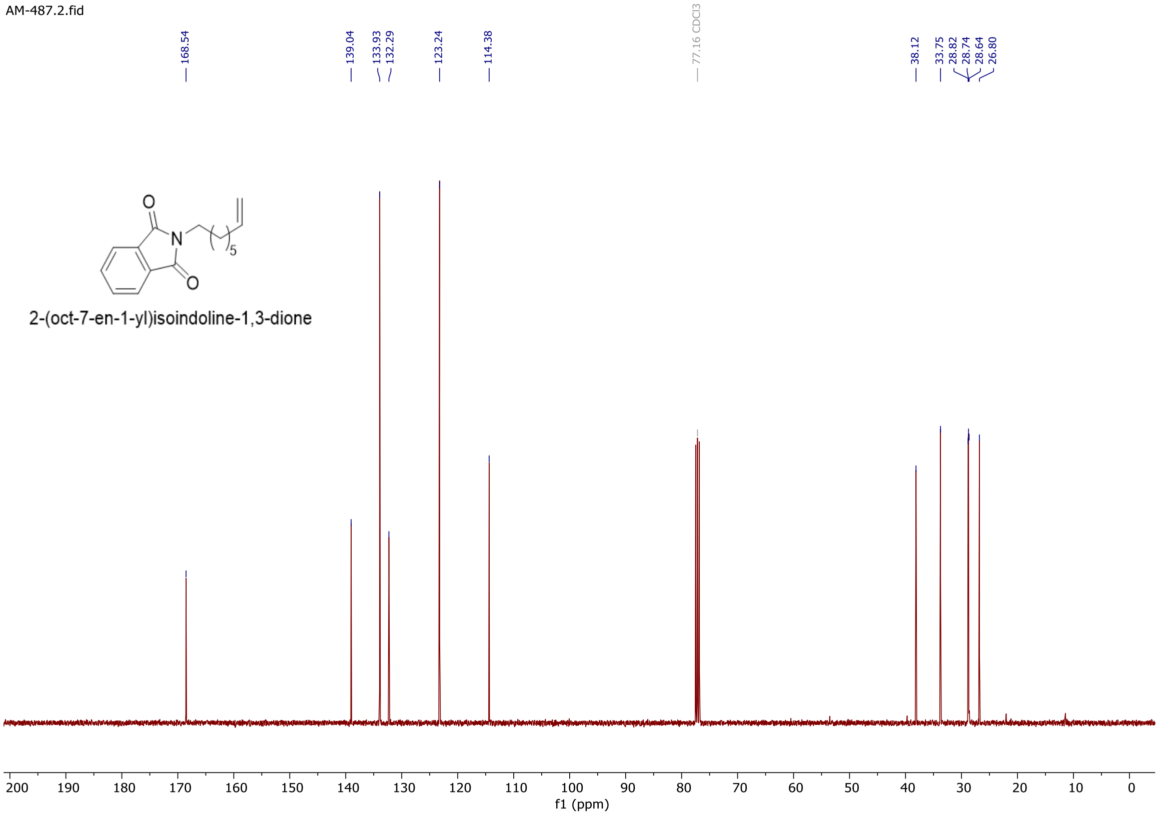

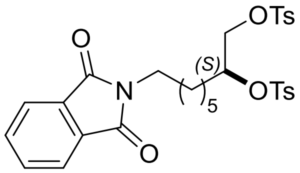

(*S*)-8-(1,3-dioxoisoindolin-2-yl)octane-1,2-diyl bis(4-methylbenzenesulfonate)

Compound 3S: Synthesized using the General Procedure on a 0.2 mmol scale; Purified using a gradient of 2% ethyl acetate in hexane to 20% ethyl acetate in hexane on silica gel; Predominant enantiomer depicted; (colorless oil, 91.0 mg, 0.152 mmol, 76% yield, 86% ee).

^{1}H NMR (400 MHz, $CDCl_3$) δ 7.83 (dd, *J* = 5.4, 3.1 Hz, 2H), 7.74 – 7.66 (m, 6H), 7.36 – 7.27 (m, 4H), 4.56 (dq, *J* = 7.0, 4.9 Hz, 1H), 4.05 – 3.98 (m, 2H), 3.66 – 3.59 (m, 2H), 2.44 (s, 3H), 2.43 (s, 3H), 1.64 – 1.52 (m, 4H), 1.23 – 1.02 (m, 6H).

$^{13}C\{^1H\}$ NMR (101 MHz, $CDCl_3$) δ 168.5, 145.3, 145.1, 134.0, 133.5, 132.4, 132.2, 130.0, 129.9, 128.1, 128.0, 123.3, 78.8, 69.5, 37.9, 31.0, 28.6, 28.5, 26.6, 24.4, 21.78, 21.75.

IR ν 3054, 2987, 2306, 1714, 1398, 1369, 1266, 1190, 1178, 897, 742, 705, 666, 555 cm^{-1}.

HRMS (ESI) m/z = $[M + Na]^+$ Calcd $C_{30}H_{33}NO_8S_2Na^+$ 622.1545. Found 622.1532 (2.1 ppm error).

Specific Rotation: $[\alpha]_D^{23}$ = +5.7 (c = 4.45 g/100 mL, $CHCl_3$, 86% ee).

Absolute stereochemistry assigned by analogy to an authentic sample of 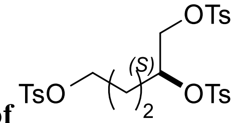

.

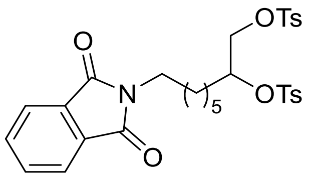

Racemic Sample: HPLC (IA-3, Isopropanol/hexanes = 10/90, flow rate = 1 mL/min, I = 220 nm), t_R = 108.9 min, 126.4 min.

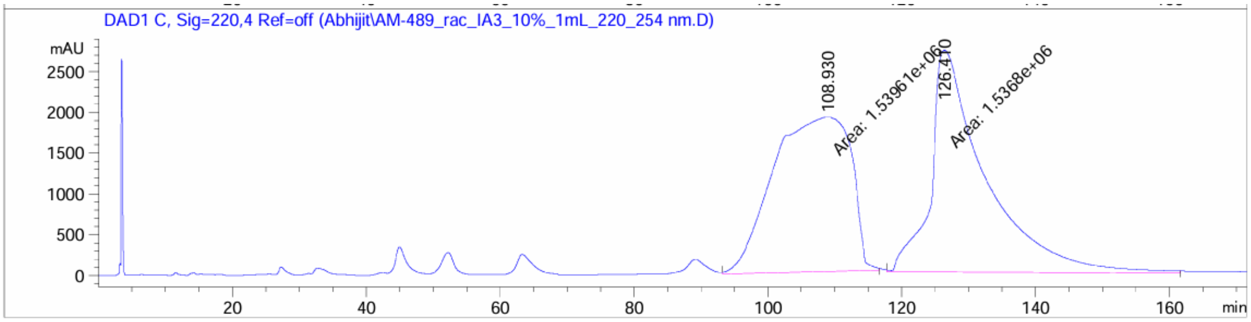

Peak #	RetTime [min]	Type	Width [min]	Area [mAU*s]	Height [mAU]	Area %
1	108.930	MM	13.5256	1.53961e6	1897.15918	50.0458
2	126.410	MM	9.4271	1.53680e6	2716.97070	49.9542

Scalemic Sample, -86% ee: HPLC (IA-3, Isopropanol/hexanes = 10/90, flow rate = 1 mL/min, I = 220 nm), t_R = 98.6 min, 122.7 min

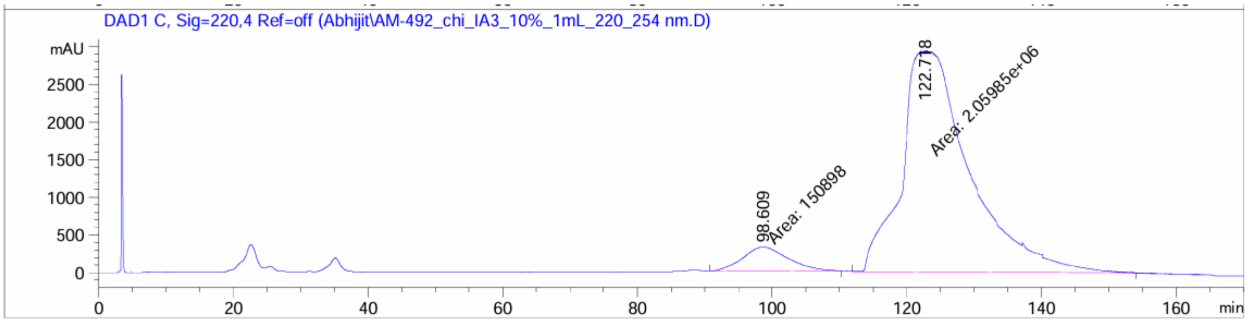

Peak #	RetTime [min]	Type	Width [min]	Area [mAU*s]	Height [mAU]	Area %
1	98.609	MM	7.8695	1.50898e5	319.58478	6.8257
2	122.718	MM	11.6789	2.05985e6	2939.56592	93.1743

Compound 3S ($CDCl_3$, ^1H NMR: 400 MHz, $^{13}C\{^1H\}$ NMR: 101 MHz)

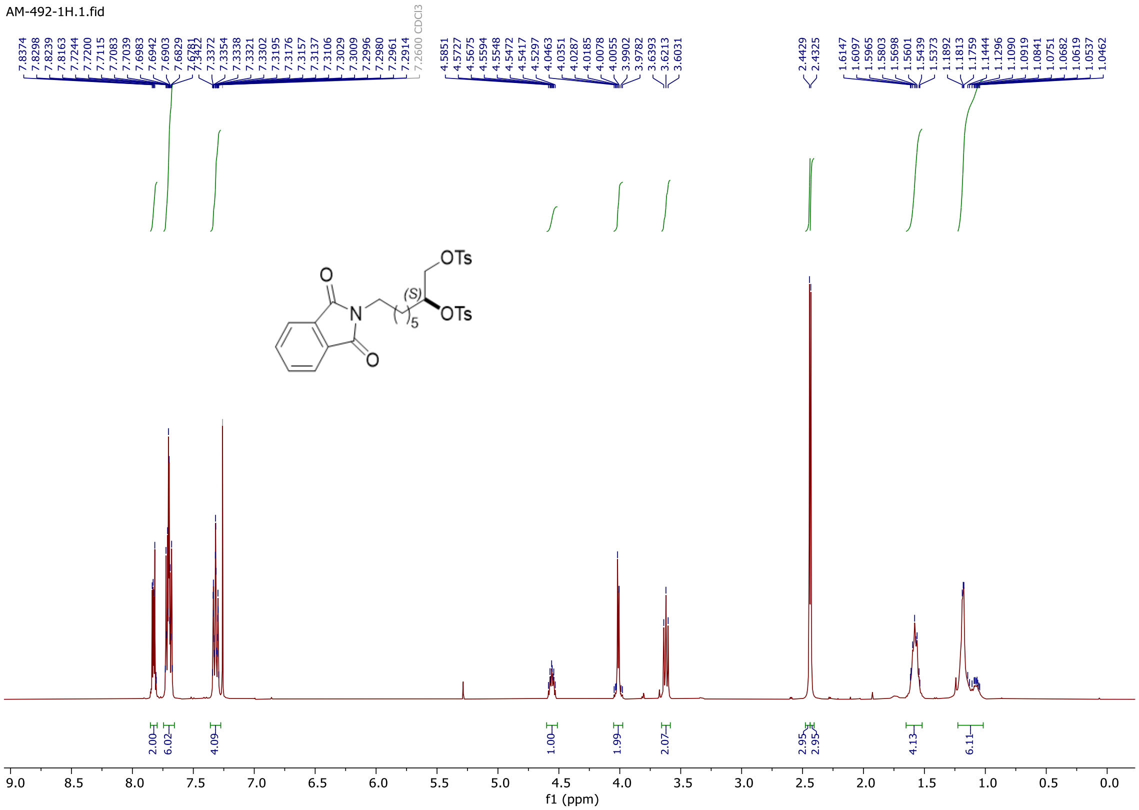

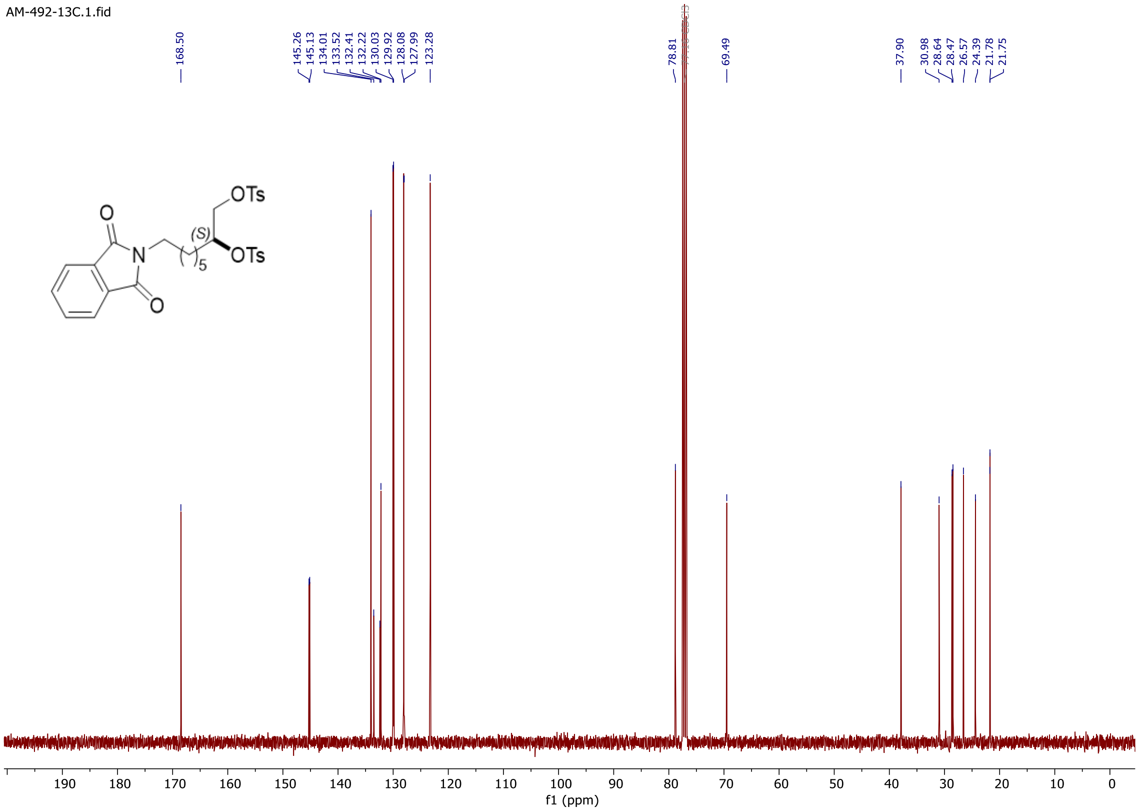

Substrate-3T-U ($CDCl_3$, ^{1}H NMR: 400 MHz, $^{13}C\{^{1}H\}$ NMR: 101 MHz)

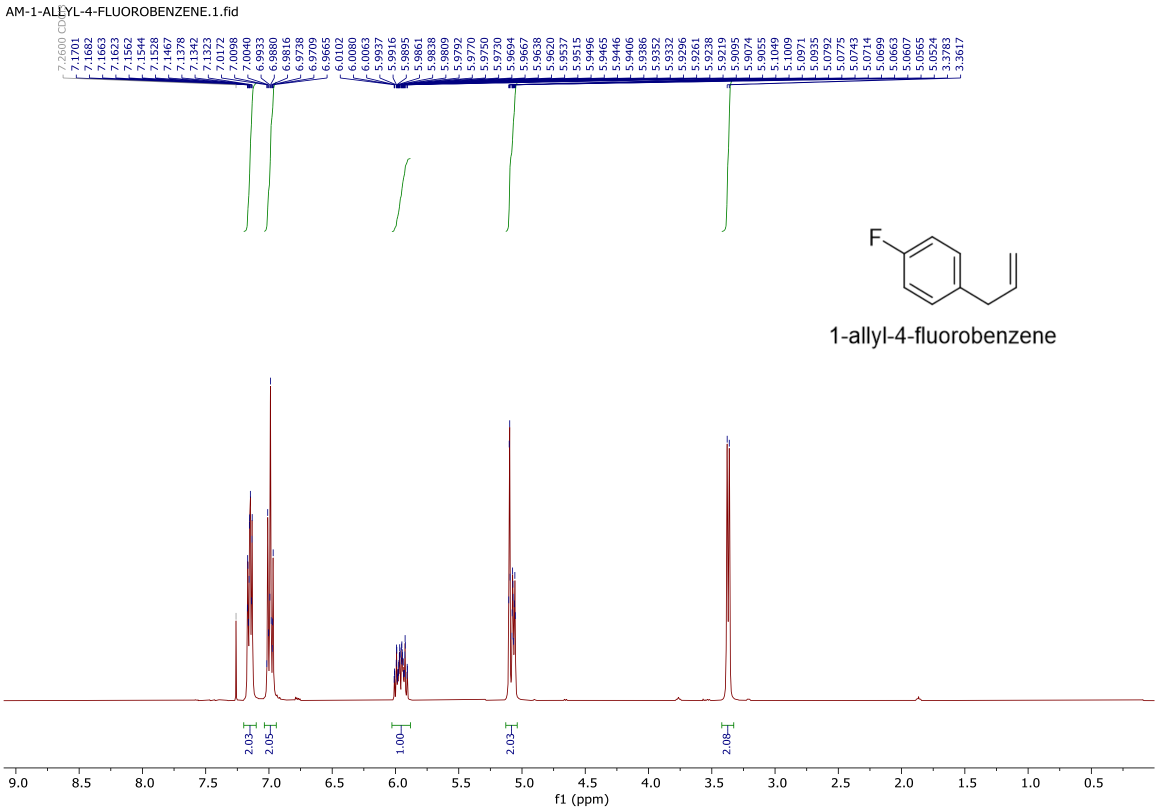

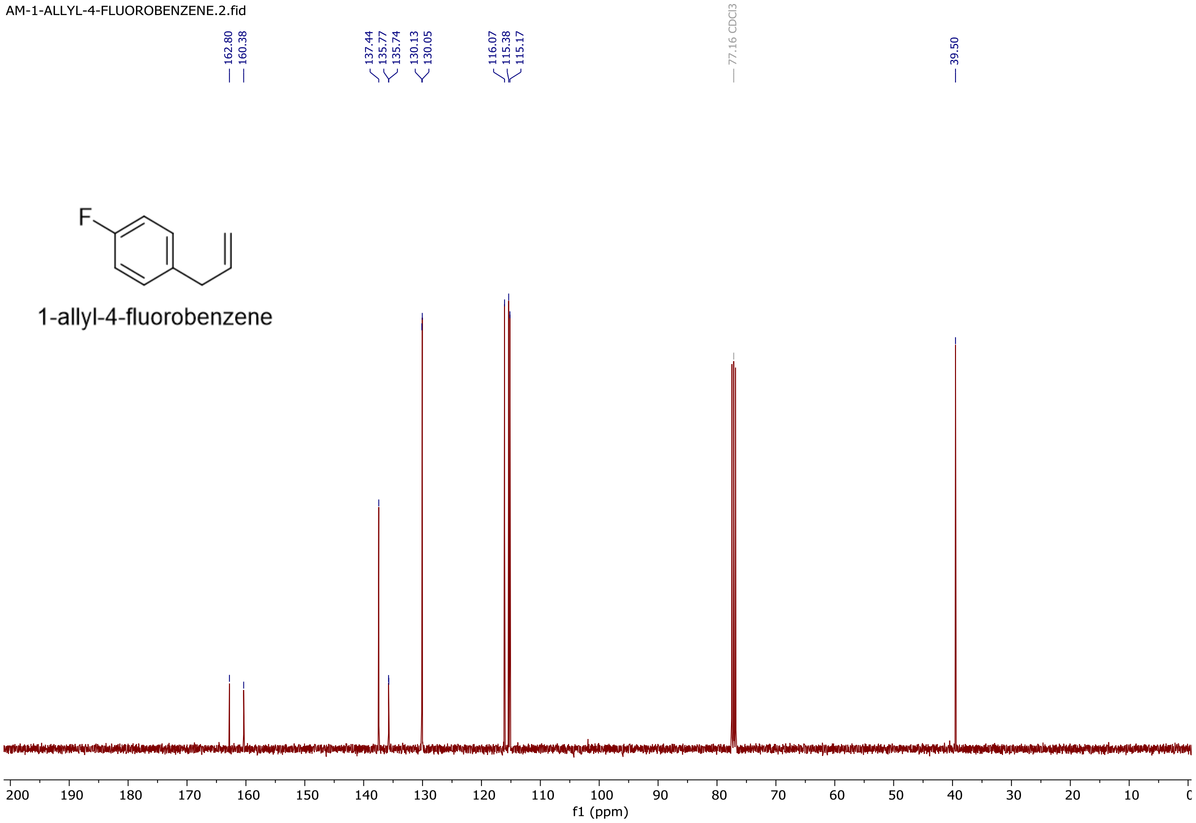

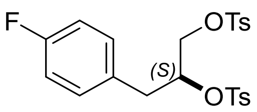

(*S*)-3-(4-fluorophenyl)propane-1,2-diyl bis(4-methylbenzenesulfonate)

Compound 3T: Synthesized using the General Procedure on a 0.2 mmol scale; Purified using a gradient of 2% ethyl acetate in hexane to 8% ethyl acetate in hexane on silica gel; Predominant enantiomer depicted; (colorless oil, 35.0 mg, 0.073 mmol, 37% yield, 18% ee).

^{1}H NMR (400 MHz, $CDCl_3$) δ 7.73 (d, *J* = 8.4 Hz, 2H), 7.53 (d, *J* = 8.1 Hz, 2H), 7.35 (d, *J* = 8.1 Hz, 2H), 7.20 (d, *J* = 8.1 Hz, 2H), 6.97 – 6.90 (m, 2H), 6.85 – 6.78 (m, 2H), 4.69 – 4.61 (m, 1H), 4.11 – 4.00 (m, 2H), 2.98 – 2.83 (m, 2H), 2.47 (s, 3H), 2.42 (s, 3H).

^{13}C{^{1}H} NMR (101 MHz, $CDCl_3$) δ 163.3 (d, *J* = 246.4 Hz), 145.5, 145.2, 132.9, 132.3, 131.0 (d, *J* = 8.1 Hz), 130.6 (d, *J* = 3.0 Hz), 130.1, 129.9, 128.2, 127.9, 115.7 (d, *J* = 21.2 Hz), 79.1, 68.8, 36.6, 21.8, 21.7.

^{19}F{^{1}H} NMR (377 MHz, $CDCl_3$) δ -115.4.

IR ν 1509, 1363, 1222, 1176, 1096, 921, 754, 554 cm^{-1}.

HRMS (ESI) m/z = $[M + Na]^+$ Calcd $C_{23}H_{23}FO_6S_2Na^+$ 501.0818. Found 501.0795 (4.6 ppm error).

Specific rotation: $[\alpha]_D^{23}$ = –2.34 (c = 1.4 g/100 mL, $CHCl_3$, 18% ee).

Stereochemistry assigned by analogy to an authentic sample of 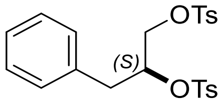 .

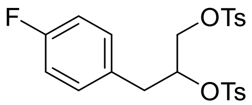

Racemic Sample: HPLC (IA-3, Isopropanol/hexanes = 5/95, flow rate = 0.5 mL/min, I = 220 nm), t_R = 76.3 min, 83.4 min.

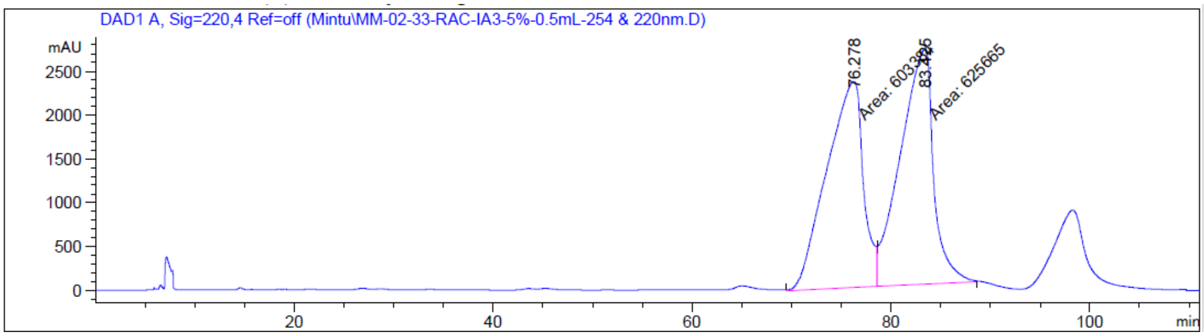

Peak #	RetTime [min]	Type	Width [min]	Area [mAU*s]	Height [mAU]	Area %
1	76.278	MF	4.2722	6.03384e5	2353.91431	49.0935
2	83.425	FM	3.8991	6.25665e5	2674.43091	50.9065

Scalemic Sample, -18% ee: HPLC (IA-3, Isopropanol/hexanes = 5/95, flow rate = 0.5 mL/min, I = 220 nm), t_R = 74.8 min, 82.3 min.

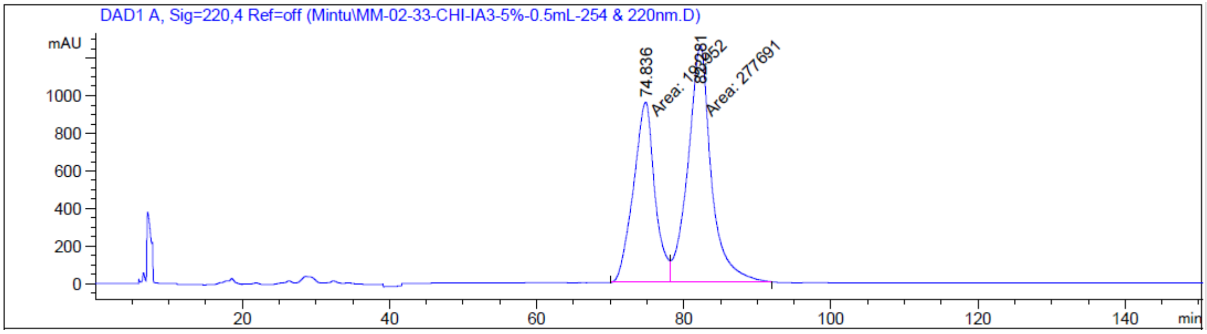

Peak #	RetTime [min]	Type	Width [min]	Area [mAU*s]	Height [mAU]	Area %
1	74.836	MF	3.3696	1.93952e5	959.32135	41.1227
2	82.281	FM	3.6754	2.77691e5	1259.22644	58.8773

Compound 3T ($CDCl_3$, ^{1}H NMR: 400 MHz, $^{13}C\{^{1}H\}$ NMR: 101 MHz)

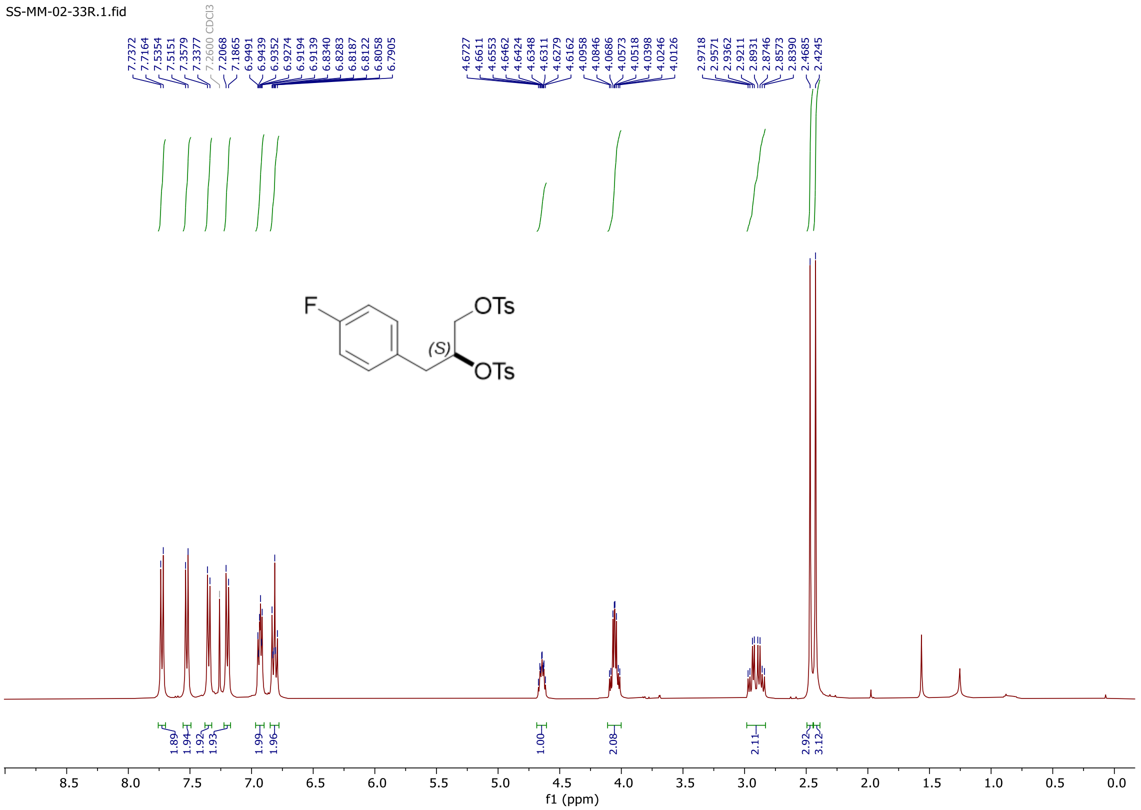

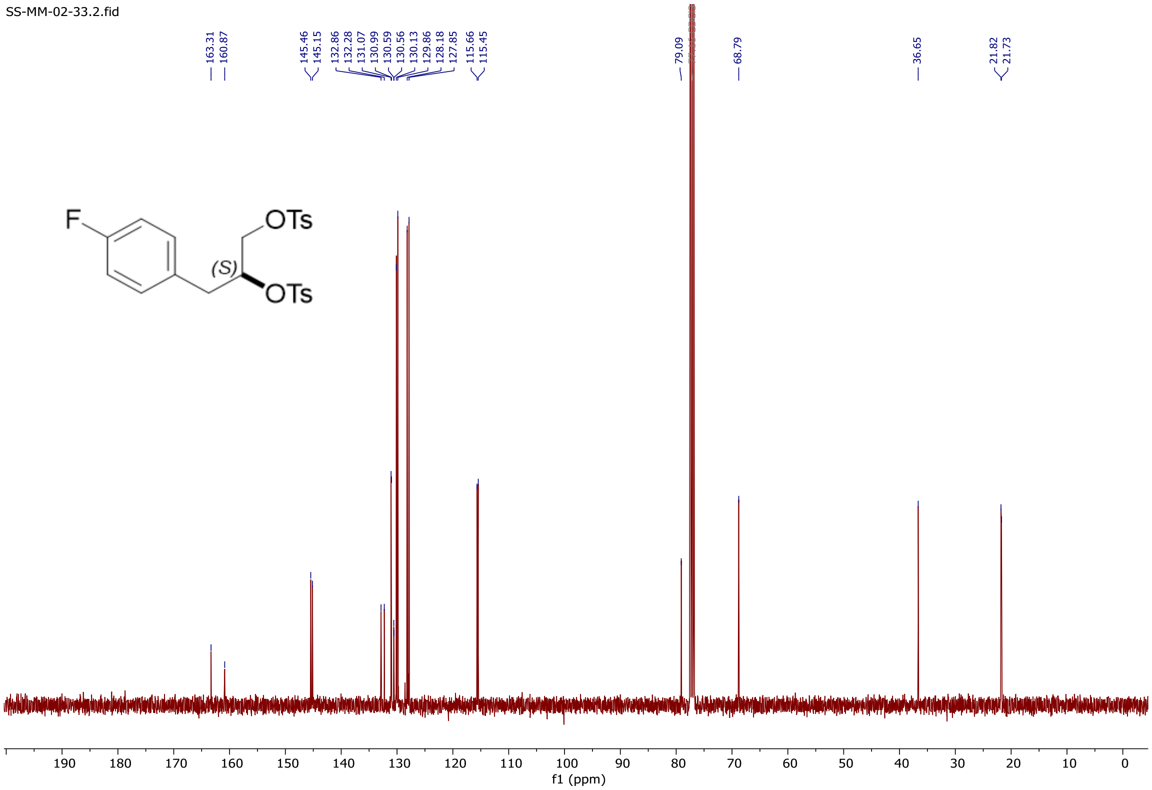

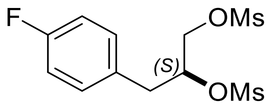

(*S*)-3-(4-fluorophenyl)propane-1,2-diyl dimethanesulfonate

Compound 3U: Synthesized using the General Procedure on a 0.2 mmol scale; Purified using a gradient of 2% ethyl acetate in hexane to 20% ethyl acetate in hexane on silica gel; Predominant enantiomer depicted; (colorless oil, 38.0 mg, 0.116 mmol, 58% yield, 9% ee).

^{1}H NMR (400 MHz, $CDCl_3$) δ 7.25 – 7.20 (m, 2H), 7.07 – 7.01 (m, 2H), 4.98 (tdd, *J* = 6.9, 5.7, 3.3 Hz, 1H), 4.39 (dd, *J* = 11.6, 3.3 Hz, 1H), 4.25 (dd, *J* = 11.6, 5.8 Hz, 1H), 3.07 (s, 3H), 3.06 – 3.04 (m, 2H), 2.75 (s, 3H).

^{13}C{^{1}H} NMR (101 MHz, $CDCl_3$) δ 163.5 (d, *J* = 248.5 Hz), 131.3 (d, *J* = 8.1 Hz), 130.6 (d, *J* = 3.6 Hz), 116.0 (d, *J* = 21.4 Hz), 79.6, 69.0, 38.4, 37.8, 36.8.

^{19}F NMR (377 MHz, $CDCl_3$) δ -114.4.

IR ν 1509, 1354, 1222, 1174, 918, 820, 525 cm^{-1}.

HRMS (ESI) m/z = $[M + Na]^+$ Calcd $C_{11}H_{15}FO_6S_2Na^+$ 349.0192. Found 349.0168 (6.9 ppm error).

Specific rotation: close to 0

Stereochemistry assigned by analogy to an authentic sample of 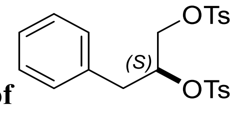 **.**

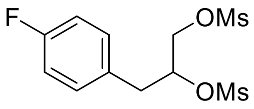

Racemic Sample: HPLC (IC-3, Isopropanol/hexanes = 20/80, flow rate = 1.0 mL/min, I = 254 nm), t_R = 17.1 min, 24.8 min.

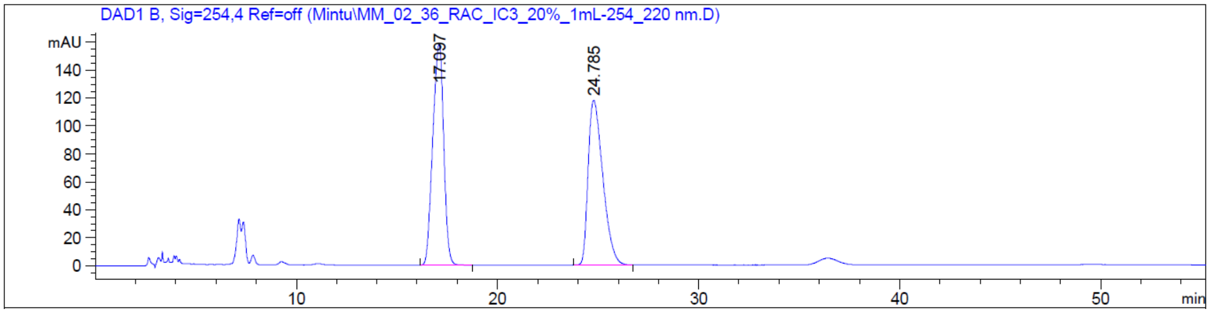

Peak #	RetTime [min]	Type	Width [min]	Area [mAU*s]	Height [mAU]	Area %
1	17.097	BB	0.6104	5958.52441	157.85263	50.0074
2	24.785	BB	0.7800	5956.75830	117.78445	49.9926

Scalemic Sample, +9% ee: HPLC (IC-3, Isopropanol/hexanes = 20/80, flow rate = 1.0 mL/min, I = 254 nm), t_R = 17.4 min, 25.3 min.

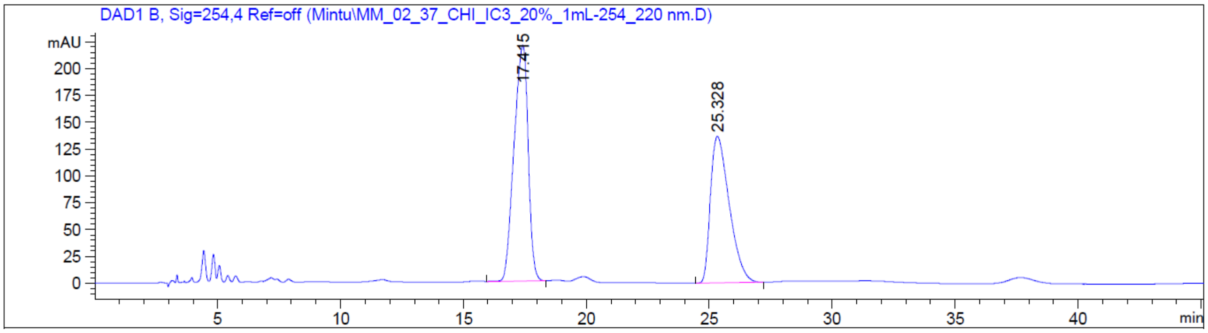

Peak #	RetTime [min]	Type	Width [min]	Area [mAU*s]	Height [mAU]	Area %
1	17.415	BB	0.6545	8736.02246	219.40315	54.5736
2	25.328	BB	0.8175	7271.76611	136.50281	45.4264

Compound 3U (CDCl$_3$, ^{1}H NMR: 400 MHz, ^{13}C{^{1}H} NMR: 101 MHz)

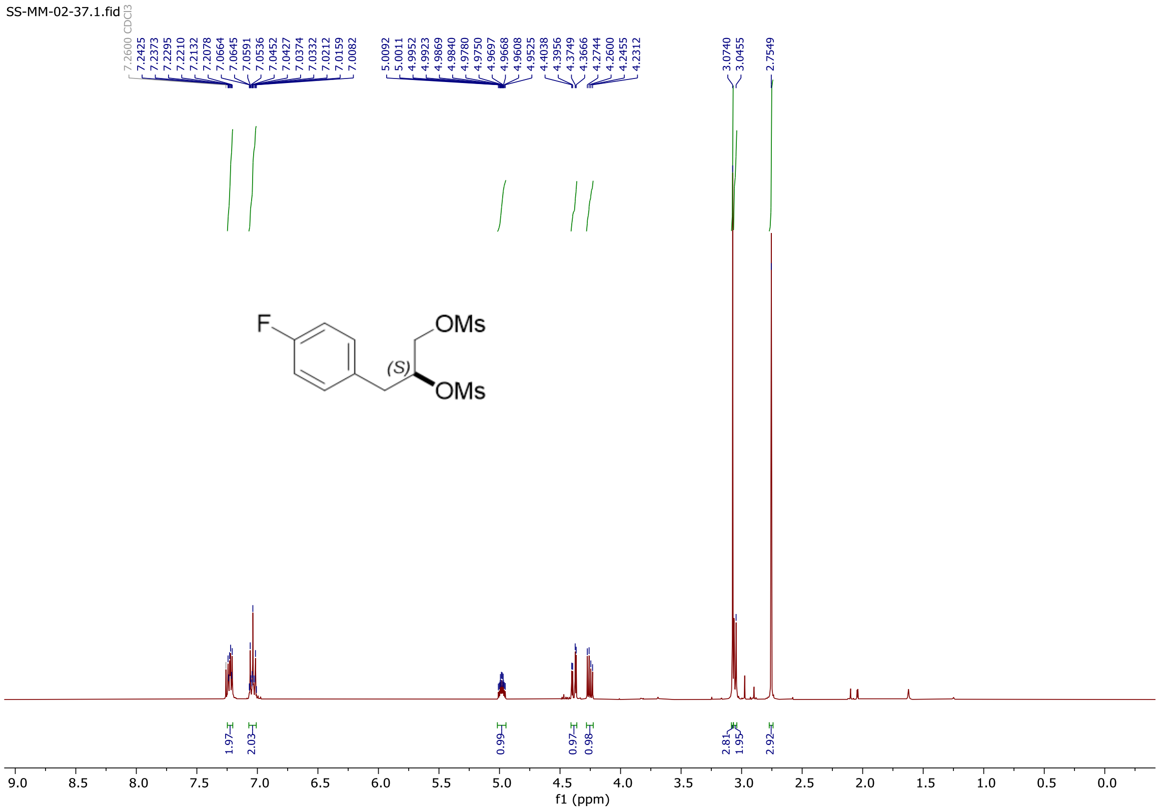

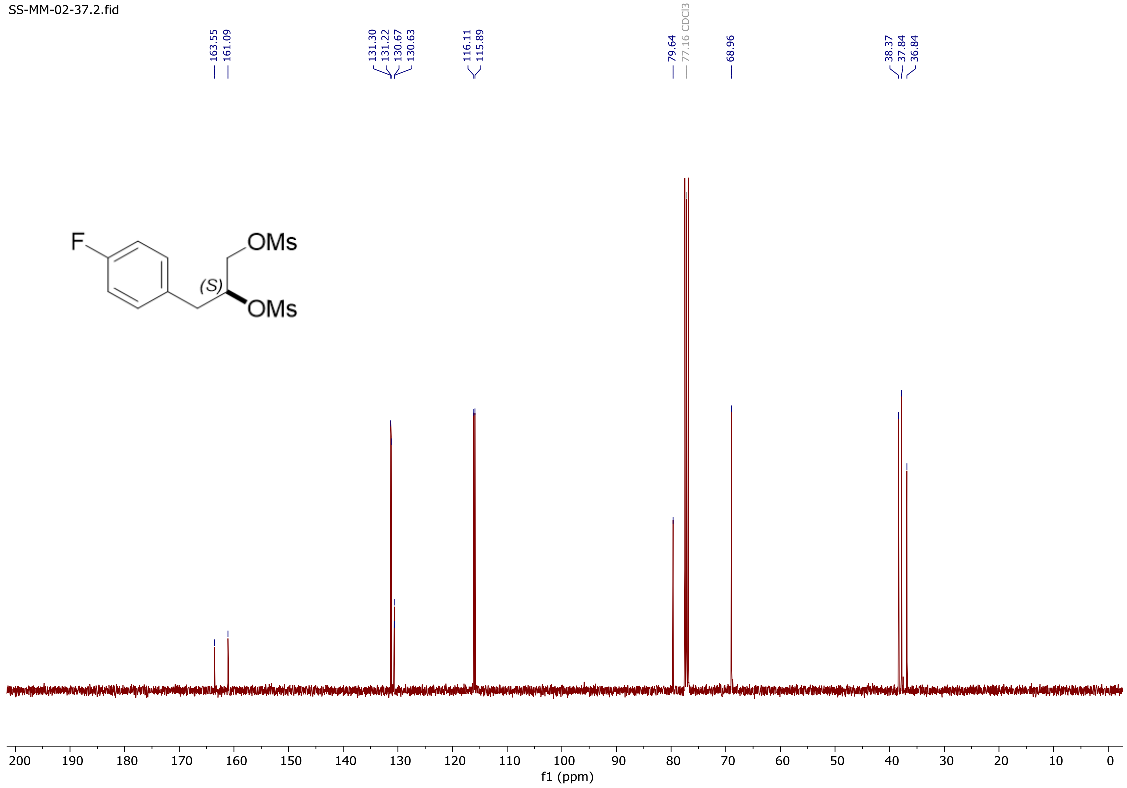

Substrate-3V ($CDCl_3$, ^{1}H NMR: 400 MHz, $^{13}C\{^{1}H\}$ NMR: 101 MHz)

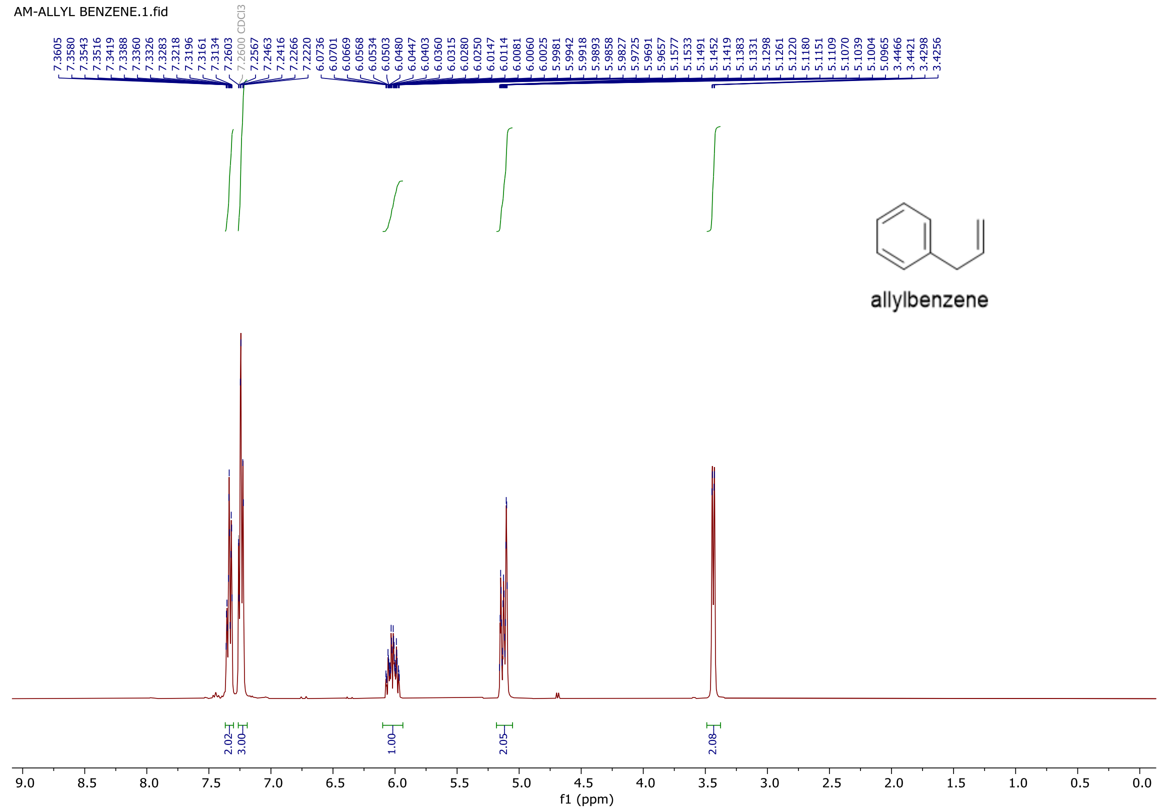

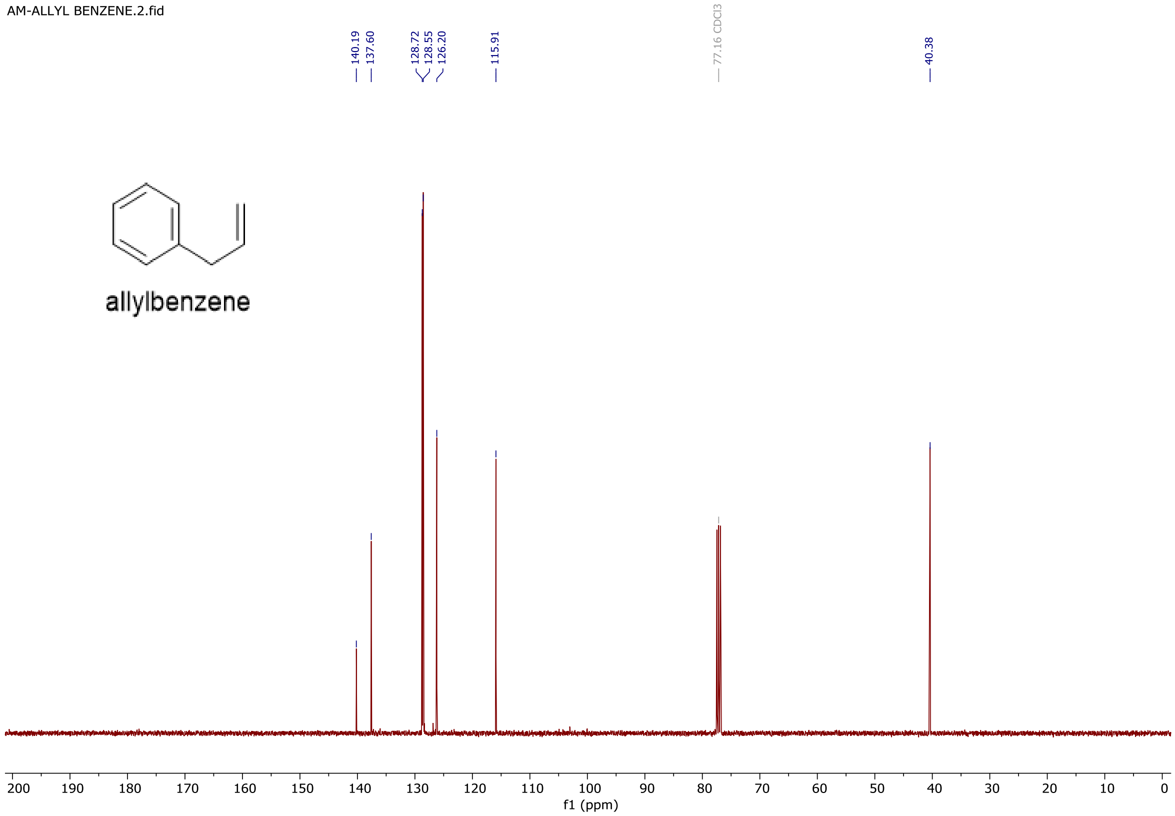

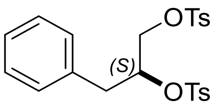

(*S*)-3-phenylpropane-1,2-diyl bis(4-methylbenzenesulfonate)

Compound 3V: Synthesized using the General Procedure on a 0.2 mmol scale; Purified using a gradient of 2% ethyl acetate in hexane to 8% ethyl acetate in hexane on silica gel; Predominant enantiomer depicted; (colorless oil, 38.0 mg, 0.083 mmol, 41% yield, 22% ee).

^{1}H NMR (400 MHz, $CDCl_3$) δ 7.73 – 7.68 (m, 2H), 7.59 – 7.54 (m, 2H), 7.33 (d, *J* = 8.2 Hz, 2H), 7.23 – 7.14 (m, 5H), 7.01 – 6.96 (m, 2H), 4.69 (tt, *J* = 6.7, 4.5 Hz, 1H), 4.07 – 4.00 (m, 2H), 2.99 – 2.88 (m, 2H), 2.46 (s, 3H), 2.42 (s, 3H).

^{13}C{^{1}H} NMR (101 MHz, $CDCl_3$) δ 145.3, 145.0, 134.8, 133.0, 132.3, 130.1, 129.9, 129.5, 128.8, 128.2, 127.9, 127.2, 79.2, 68.7, 37.5, 21.82, 21.78.

IR ν 1363, 1174, 915, 812, 663, 554 cm^{-1}.

HRMS (ESI) m/z = $[M + Na]^+$ Calcd $C_{23}H_{24}O_6S_2Na^+$ 483.0912. Found 483.0890 (4.6 ppm error).

Specific Rotation: $[\alpha]_D^{22}$ = –3.18 (c = 1.2 g/100 mL, $CHCl_3$, 22% ee).

Stereochemistry assigned by analogy to an authentic sample of 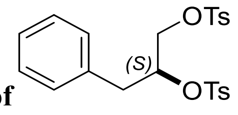 **.**

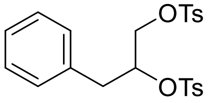

Racemic Sample: HPLC (IA-3, Isopropanol/hexanes = 5/95, flow rate = 0.6 mL/min, I = 220 nm), t_R = 53.1 min, 60.1 min.

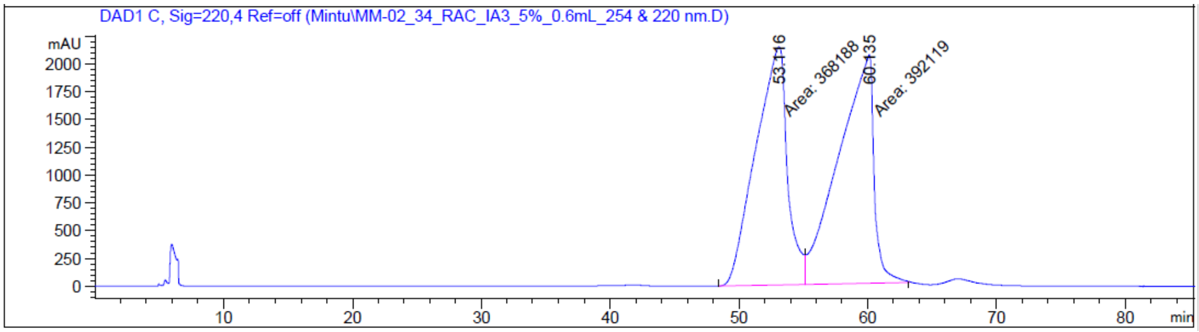

Peak #	RetTime [min]	Type	Width [min]	Area [mAU*s]	Height [mAU]	Area %
1	53.116	MF	2.8605	3.68188e5	2145.22388	48.4262
2	60.135	FM	3.1728	3.92119e5	2059.77612	51.5738

Scalemic Sample, -22% ee: HPLC (IA-3, Isopropanol/hexanes = 5/95, flow rate = 0.6 mL/min, I = 220 nm), t_R = 54.7 min, 62.8 min.

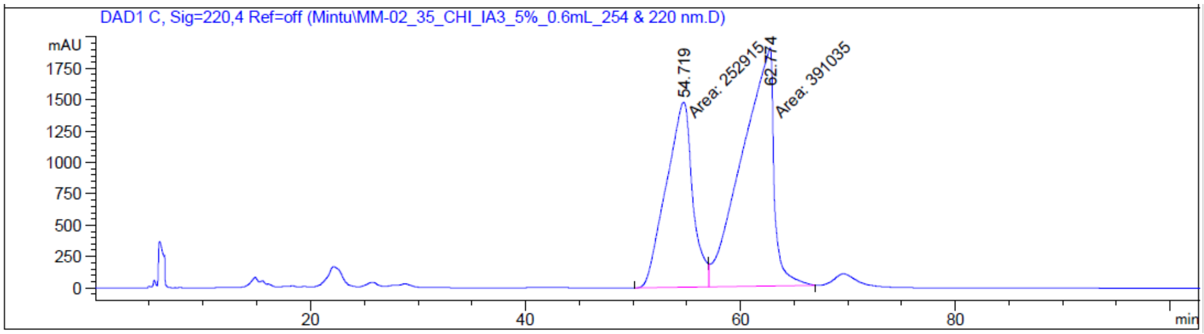

Peak #	RetTime [min]	Type	Width [min]	Area [mAU*s]	Height [mAU]	Area %
1	54.719	MF	2.8588	2.52915e5	1474.45898	39.2755
2	62.774	FM	3.4349	3.91035e5	1897.38855	60.7245

Compound 3V ($CDCl_3$, ^1H NMR: 400 MHz, $^{13}C\{^1H\}$ NMR: 101 MHz)

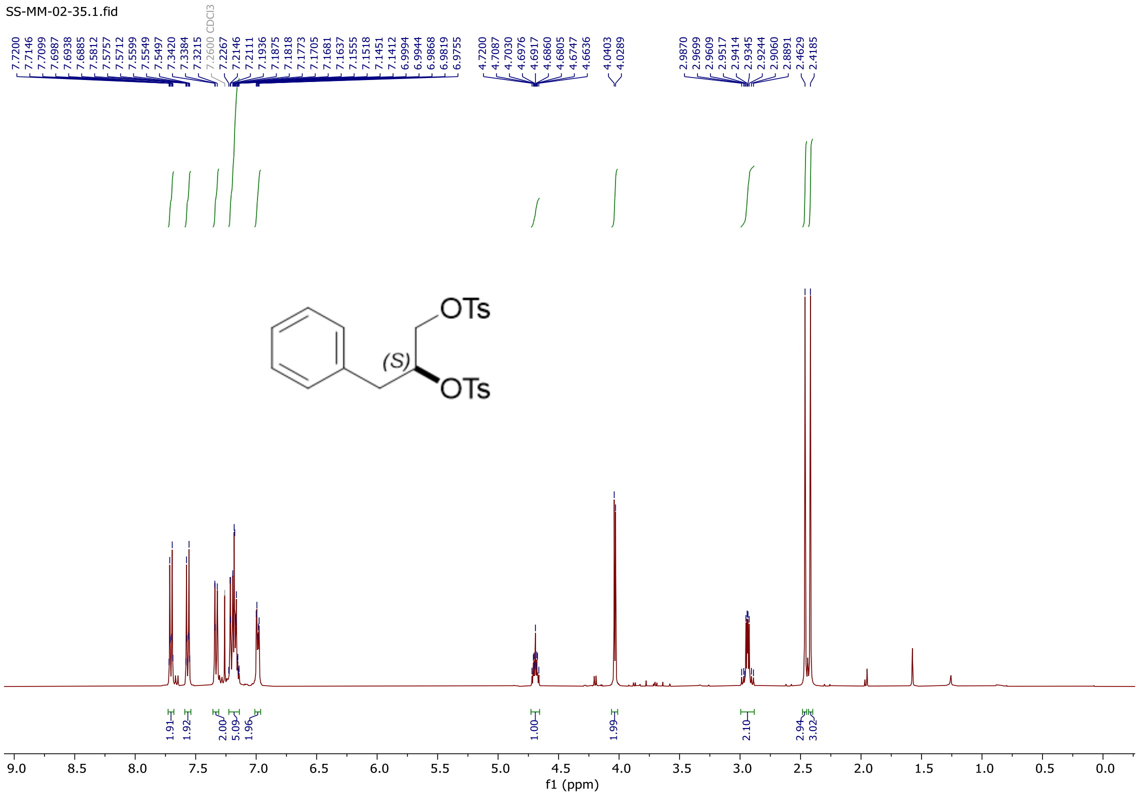

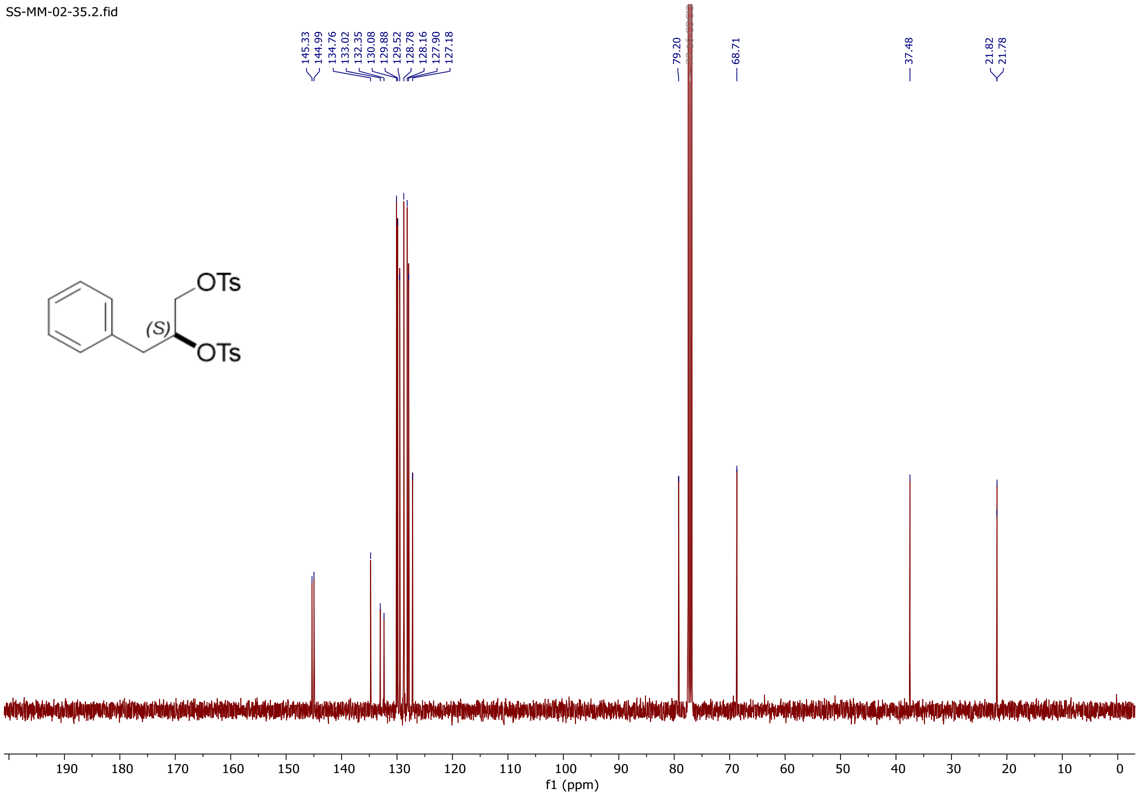

V. Procedures, Characterization Data, HPLC Traces, and NMR Spectra for Scale-Up and Application Examples

Scale-up procedure

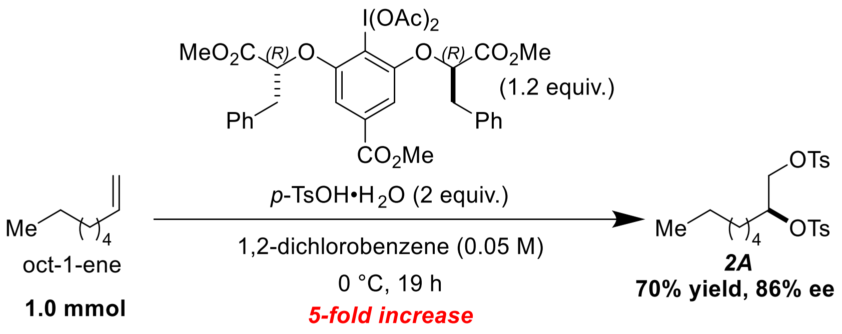

An oven-dried 50 mL round-bottom flask was cooled under nitrogen gas and charged with a stir bar, oct-1-ene (0.112 g, 1.0 mmol, 1 equiv.), 1,2-dichlorobenzene (20 mL, reaction concentration = 0.05 M), enantiopure I(III) oxidant (0.884 g, 1.2 mmol, 1.2 equiv.), and p-TsOH•H_2O (0.380 g, 2.0 mmol, 2 equiv.). The flask was sealed, and the reaction mixture was cooled to 0 °C using a Julabo immersion cooler and stirred at this temperature for 19 h. Following this time, the seal was broken, and the contents of the flask were transferred to a separatory funnel with DCM (50 mL). The organic layer was washed with saturated aqueous $Na_2S_2O_3$ solution (50 mL) followed by saturated aqueous $NaHCO_3$ solution (50 mL). The organic layer was collected, dried with Na_2SO_4, filtered, and concentrated under reduced pressure. The resulting residue was purified by chromatography on silica gel using a gradient of 2% ethyl acetate in hexane to 8% ethyl acetate in hexane to yield pure product as a colorless oil (318.0 mg, 0.7 mmol, 70% yield, 86% ee).

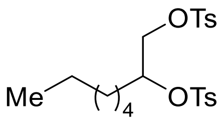

Racemic Sample: HPLC (IC-3, Isopropanol/hexanes = 10/90, flow rate = 1 mL/min, I = 254 nm), t_R = 79.5 min, 91.4 min.

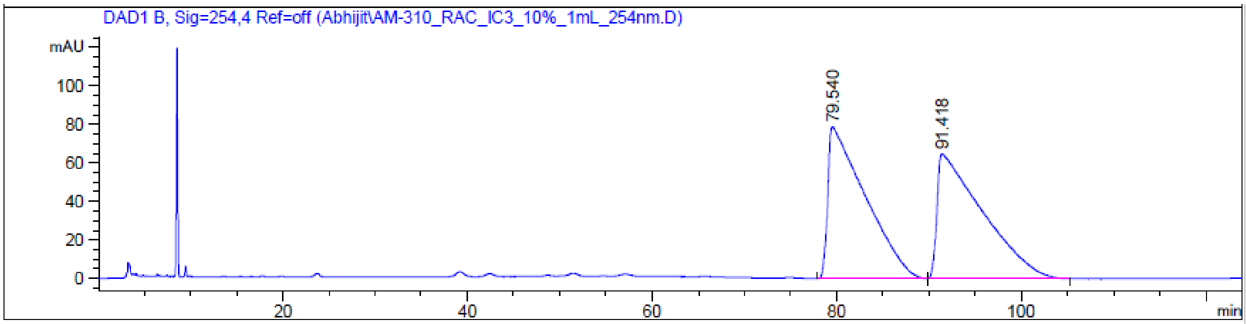

Peak #	RetTime [min]	Type	Width [min]	Area [mAU*s]	Height [mAU]	Area %
1	79.540	BB	3.4424	2.21416e4	78.79465	49.9636
2	91.418	BB	4.1422	2.21739e4	64.76858	50.0364

Scalemic Sample, +86% ee: HPLC (IC-3, Isopropanol/hexanes = 10/90, flow rate = 1 mL/min, I = 254 nm), t_R = 78.5 min, 94.7 min

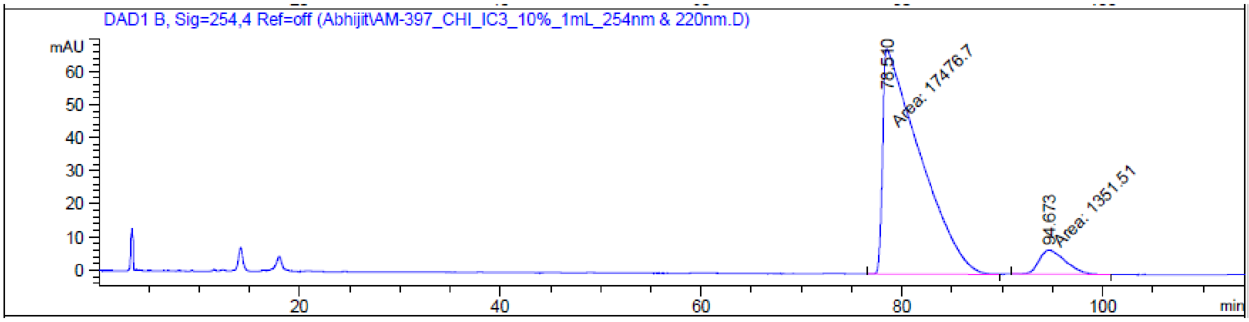

Peak #	RetTime [min]	Type	Width [min]	Area [mAU*s]	Height [mAU]	Area %
1	78.510	MM	4.2985	1.74767e4	67.76289	92.8219
2	94.673	MM	3.1109	1351.50647	7.24065	7.1781

Applications

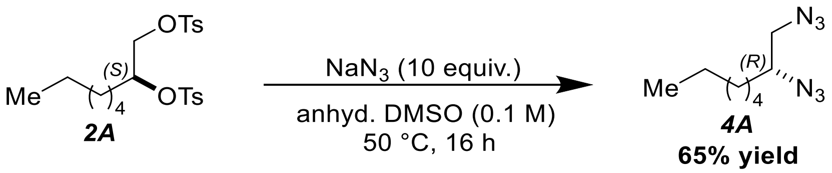

A 10 mL microwave vial equipped with a magnetic stir bar was charged with compound **2A** (0.318 g, 0.7 mmol, 1 equiv.), NaN_3 (0.455 g, 7 mmol, 10 equiv.), and anhydrous DMSO (7 mL, final reaction concentration = 0.1 M). The vial was sealed and immersed into an oil-bath pre-heated to 50 °C. The reaction was stirred at this temperature for 16 hours. Then, the vial was removed from the oil bath, cooled to room temperature, and the seal was broken. The contents of the vial were transferred to a separatory funnel with EtOAc (20 mL). The organic layer was washed with brine (2 x 20 mL), collected, dried with Na_2SO_4, filtered, and concentrated *in vacuo*. The resulting residue was purified by chromatography on silica gel (gradient of 0 to 2% EtOAc/hexanes) to give **4A** (colorless oil, 0.089 g, 0.453 mmol, 65% yield).

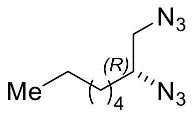

(*R*)-1,2-diazidooctane

Compound 4A:

^{1}H NMR (400 MHz, $CDCl_3$) δ 3.50 – 3.42 (m, 1H), 3.41 – 3.27 (m, 2H), 1.60 – 1.50 (m, 2H), 1.46 – 1.24 (m, 8H), 0.95 – 0.84 (m, 3H).

^{13}C{^{1}H} NMR (101 MHz, $CDCl_3$) δ 62.2, 55.0, 31.9, 31.7, 29.1, 26.0, 22.6, 14.1.

IR ν 3054, 2932, 2860, 2306, 2105, 1422, 1340, 1266, 896, 746, 705, 554 cm^{-1}.

HRMS (ESI): Molecular ion not detected.

Specific Rotation: $[\alpha]_D^{25}$ = +31.5 (c = 1.25 g/100 mL, $CHCl_3$).

Compound 4A ($CDCl_3$, ^1H NMR: 400 MHz, $^{13}C\{^1H\}$ NMR: 101 MHz)

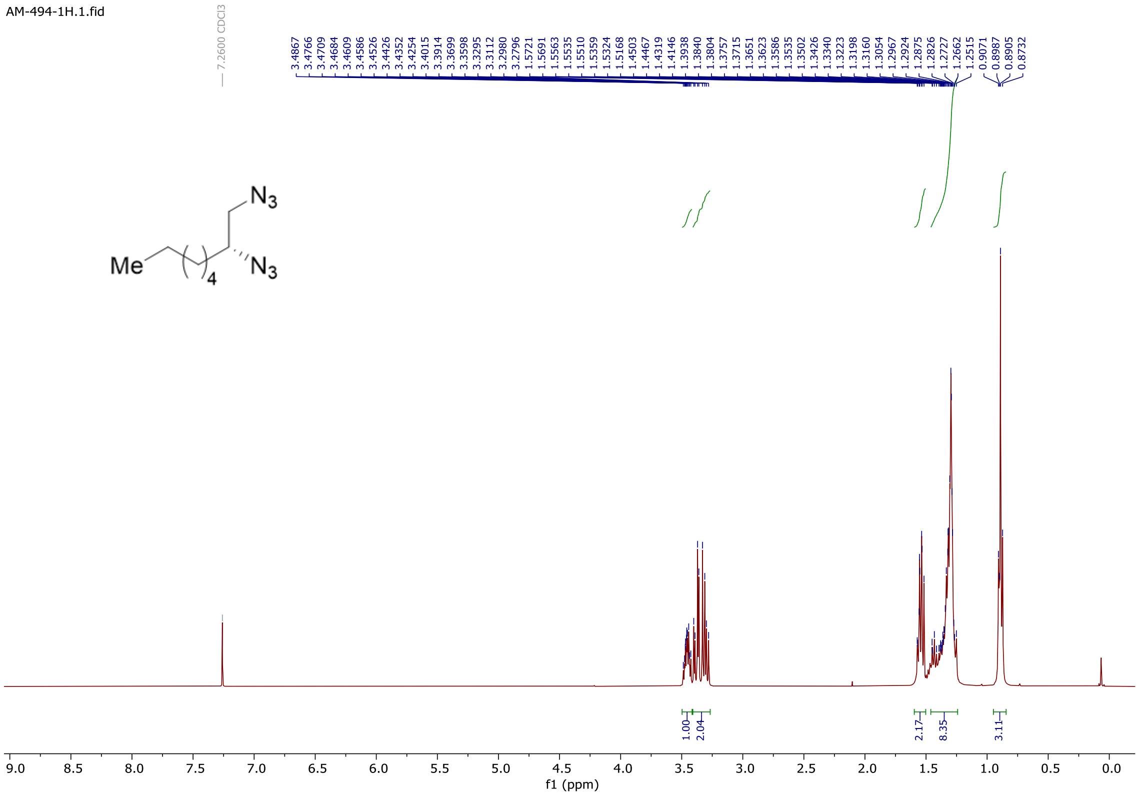

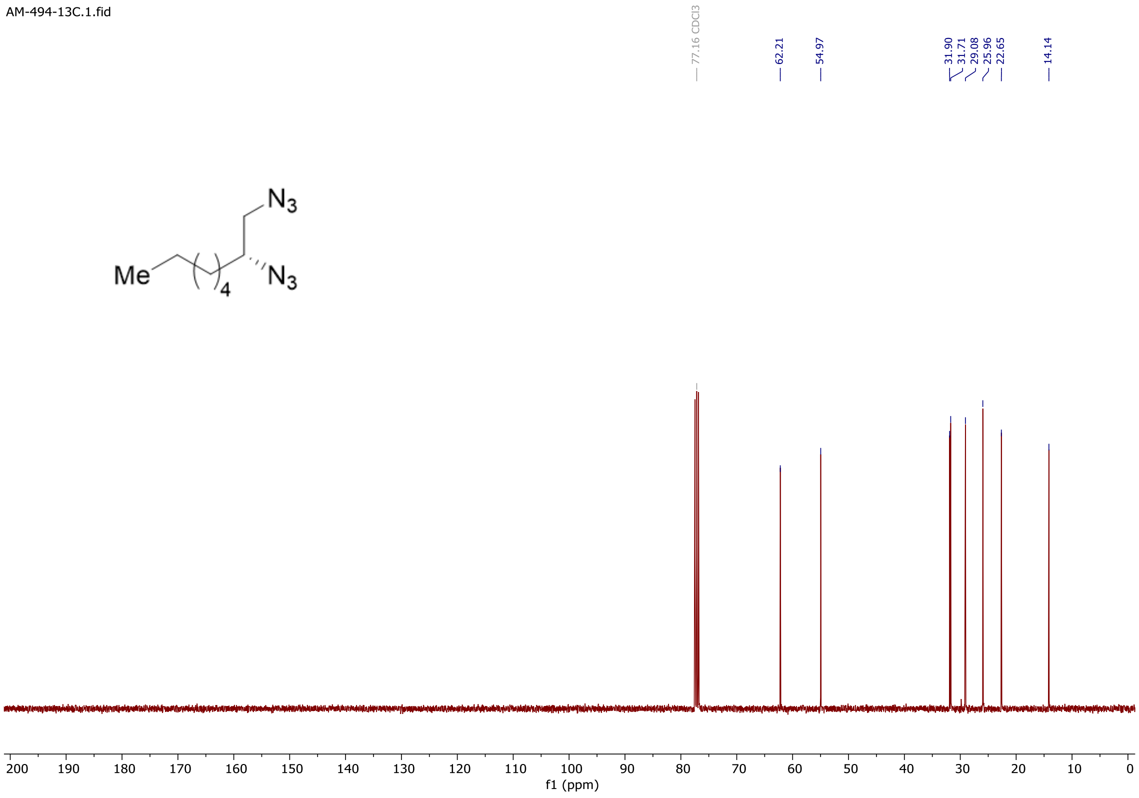

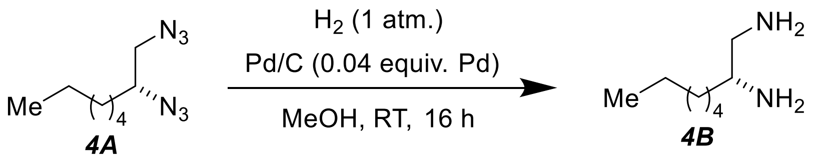

A 25 mL round-bottom flask equipped with a magnetic stir bar was charged with **4A** (78.5 mg, 0.4 mmol, 1 equiv.), anhydrous MeOH (7.0 mL, Reaction Concentration = 0.057 M), and 10% Pd/C (0.016 g of mixture, 0.0016 g of Pd, 0.015 mmol Pd, 0.04 equiv. Pd). The reaction mixture was stirred under 1 atm of H_2 gas (delivered by a rubber balloon) for 16 hours at room temperature. Following this time, the reaction mixture was filtered through a short pad of Celite using MeOH (30 mL). The filtrate was concentrated under reduced pressure to give product **4B** as a colorless oil (44.4 mg, 0.31 mmol, 77% yield), and this was used without further purification.

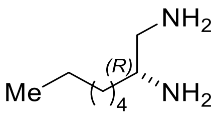

(*R*)-octane-1,2-diamine

Compound 4B:

^{1}H NMR (400 MHz, $CDCl_3$) δ 2.84 – 2.54 (m, 2H), 2.43 (dd, *J* = 12.3, 7.3 Hz, 1H), 1.92 – 1.55 (m, 4H), 1.45 – 1.33 (m, 2H), 1.32 – 1.14 (m, 8H), 0.89 – 0.81 (m, 3H).

$^{13}C\{^1H\}$ NMR (101 MHz, $CDCl_3$) δ 53.6, 48.5, 35.8, 31.9, 29.5, 26.3, 22.7, 14.2.

IR ν 3054, 2987, 2306, 1419, 1266, 896, 739, 706 cm^{-1}.

HRMS (ESI) m/z = $[M + H]^+$ Calcd $C_8H_{21}N_2^+$ 145.1705. Found 145.1699 (4.1 ppm error).

Compound 4B ($CDCl_3$, ^1H NMR: 400 MHz, $^{13}C\{^1H\}$ NMR: 101 MHz)

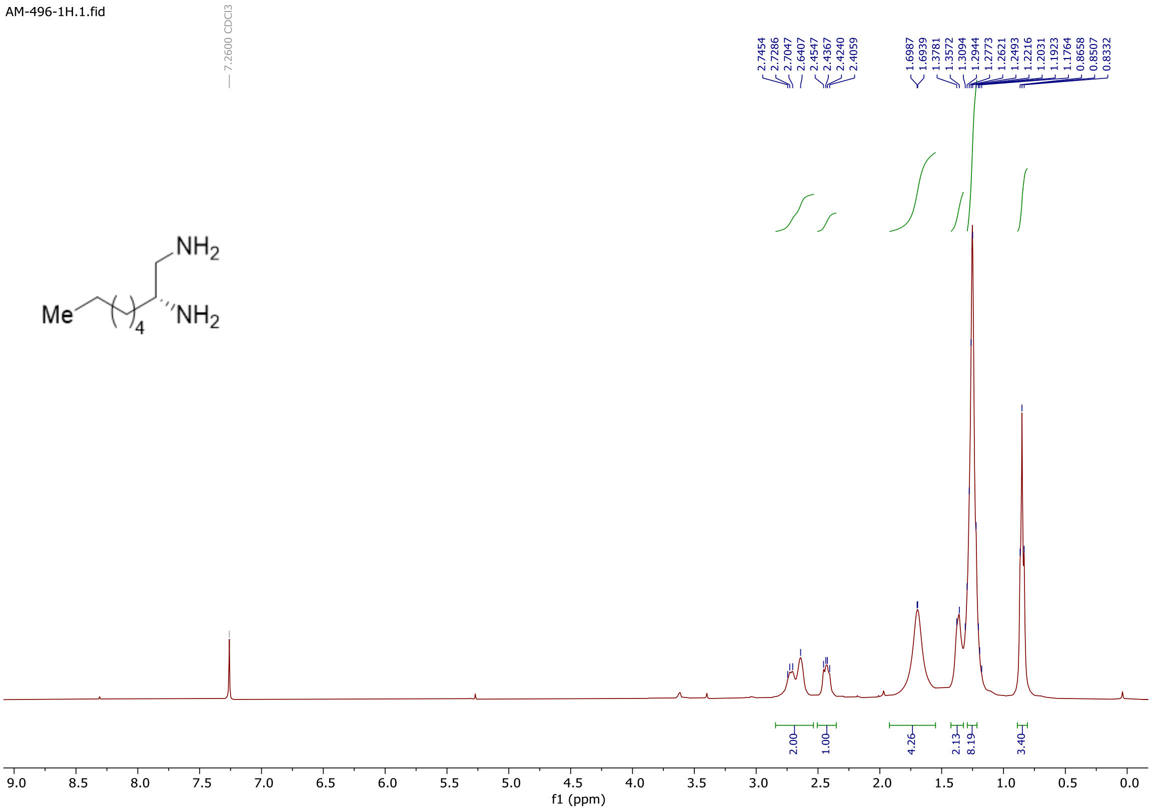

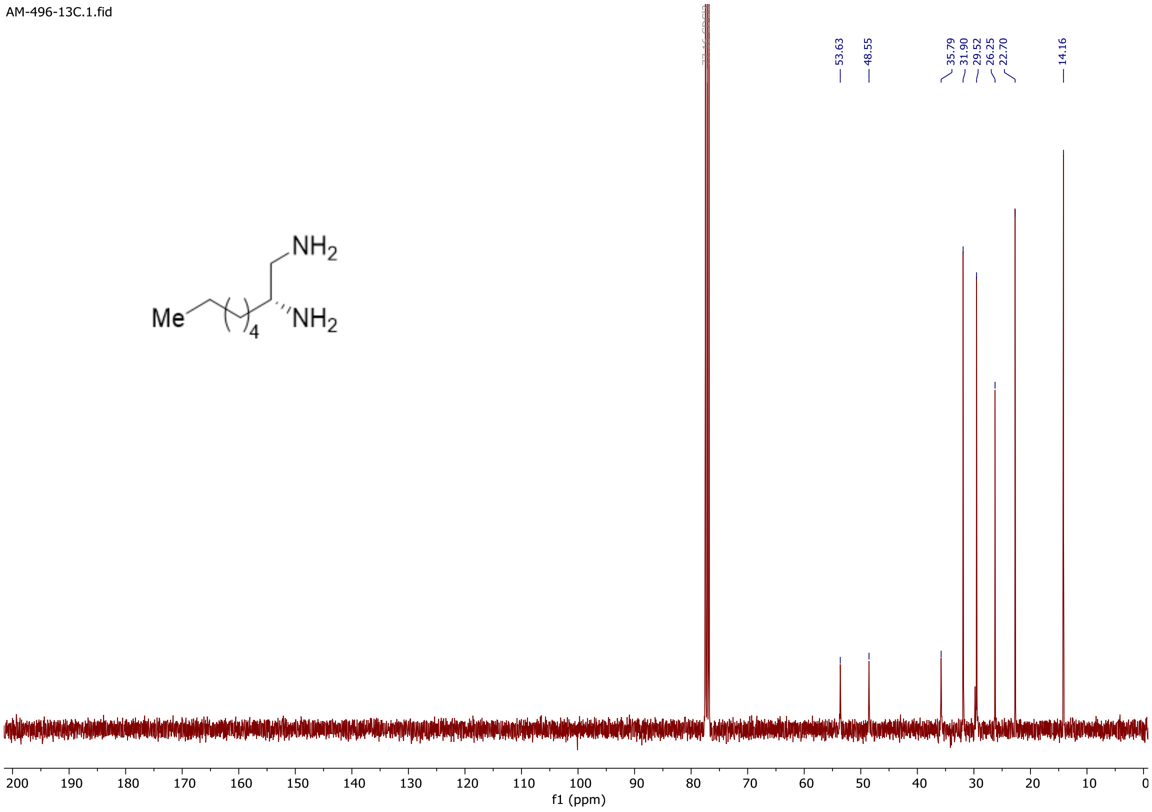

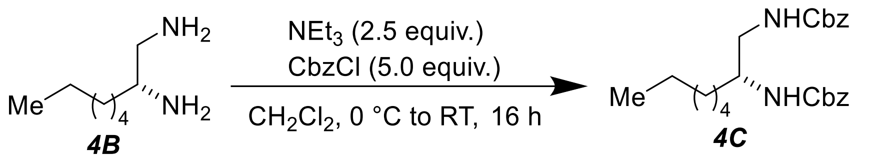

A 10 mL microwave vial equipped with a magnetic stir bar was charged with **4B** (21.6 mg, 0.15 mmol, 1 equiv.) and anhydrous CH_2Cl_2 (1.5 mL, reaction concentration = 0.1 M). The vial was sealed and cooled to 0 °C using an ice-water bath. Et_3N (53 µL, 37.9 mg, 0.375 mmol, 2.5 equiv.) and CbzCl (106 µL, 127 mg, 0.74 mmol, ~5 equiv.) were added dropwise to the vial at 0 °C. Then, the stirring reaction mixture was warmed to room temperature over a period of 16 hours. Following this time, the seal was broken, and the contents of the vial were transferred to a separatory funnel with CH_2Cl_2 (5 mL). The organic layer was washed with brine (2 x 10 mL), collected, dried with Na_2SO_4, filtered, and concentrated *in vacuo*. The resulting residue was purified by chromatography on silica gel (gradient of 4 to 20% EtOAc/hexanes) to give **4C** (white solid, 31.5 mg, 0.076 mmol, 51% yield, 88% ee).

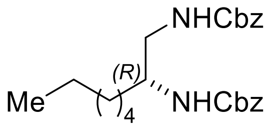

(*R*)-dibenzyl octane-1,2-diyl-dicarbamate

Compound 4C:

^{1}H NMR (400 MHz, $CDCl_3$) δ 7.40 – 7.27 (m, 10H), 5.16 (s, 1H), 5.12 – 5.02 (m, 4H), 4.92 – 4.75 (m, 1H), 3.77 – 3.63 (m, 1H), 3.33 (dt, *J* = 13.9, 4.9 Hz, 1H), 3.22 (d, *J* = 7.0 Hz, 1H), 1.38 – 1.18 (m, 10H), 0.91 – 0.82 (m, 3H).

^{13}C NMR (101 MHz, $CDCl_3$) δ 157.1, 156.7, 136.62, 136.57, 128.65, 128.63, 128.24, 128.19, 128.14, 66.9 (2C), 52.0, 45.6, 32.9, 31.8, 29.2, 25.9, 22.7, 14.2.

IR ν 3054, 2987, 2306, 1719, 1517, 1508, 1422, 1266, 896, 746, 705 cm^{-1}.

HRMS (ESI) m/z = $[M + Na]^+$ Calcd $C_{24}H_{32}N_2O_4Na^+$ 435.2260. Found 435.2244 (3.7 ppm error).

Specific Rotation: $[\alpha]_D^{24}$ = +6.2 (c = 1.0 g/100 mL, $CHCl_3$, 88% ee).

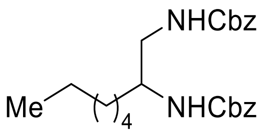

Racemic Sample: HPLC (IC-3, Isopropanol/hexanes = 05/95, flow rate = 1 mL/min, I = 254 nm), t_R = 22.2 min, 36.0 min.

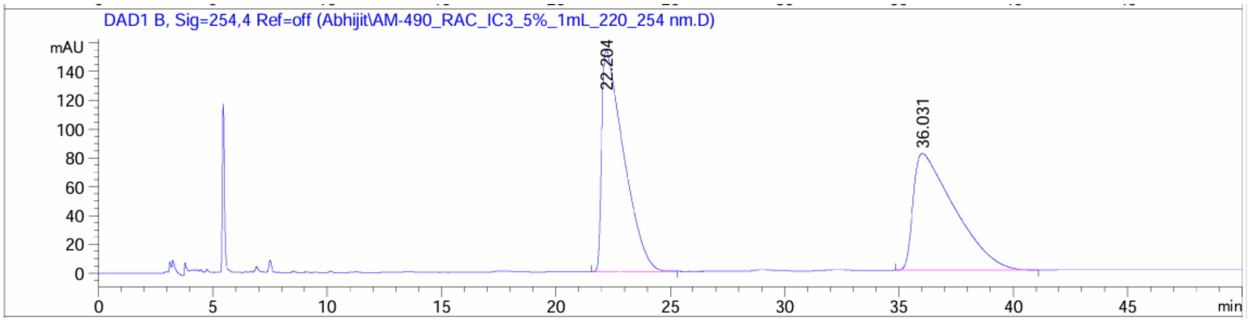

Peak #	RetTime [min]	Type	Width [min]	Area [mAU*s]	Height [mAU]	Area %
1	22.204	BB	0.9068	1.02573e4	154.05122	50.0952
2	36.031	BB	1.6628	1.02183e4	81.16633	49.9048

Scalemic Sample, +88% ee: HPLC (IC-3, Isopropanol/hexanes = 05/95, flow rate = 1 mL/min, I = 254 nm), t_R = 22.3 min, 38.2 min

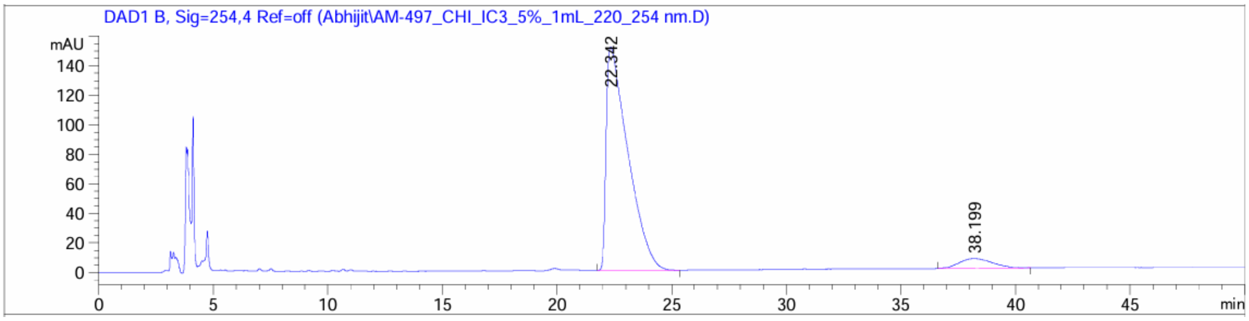

Peak #	RetTime [min]	Type	Width [min]	Area [mAU*s]	Height [mAU]	Area %
1	22.342	BB	0.9079	9978.10547	151.59738	93.7501
2	38.199	BB	1.1800	665.19226	6.64405	6.2499

Compound 4C ($CDCl_3$, ^1H NMR: 400 MHz, $^{13}C\{^1H\}$ NMR: 101 MHz)

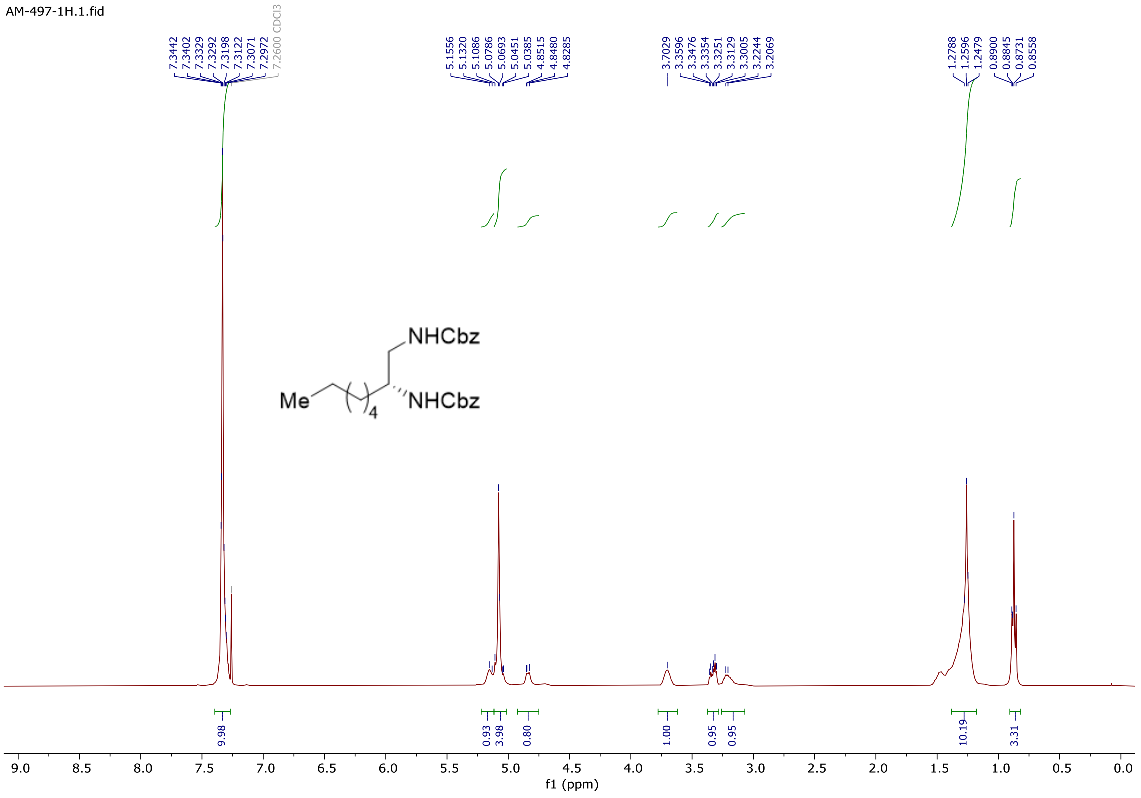

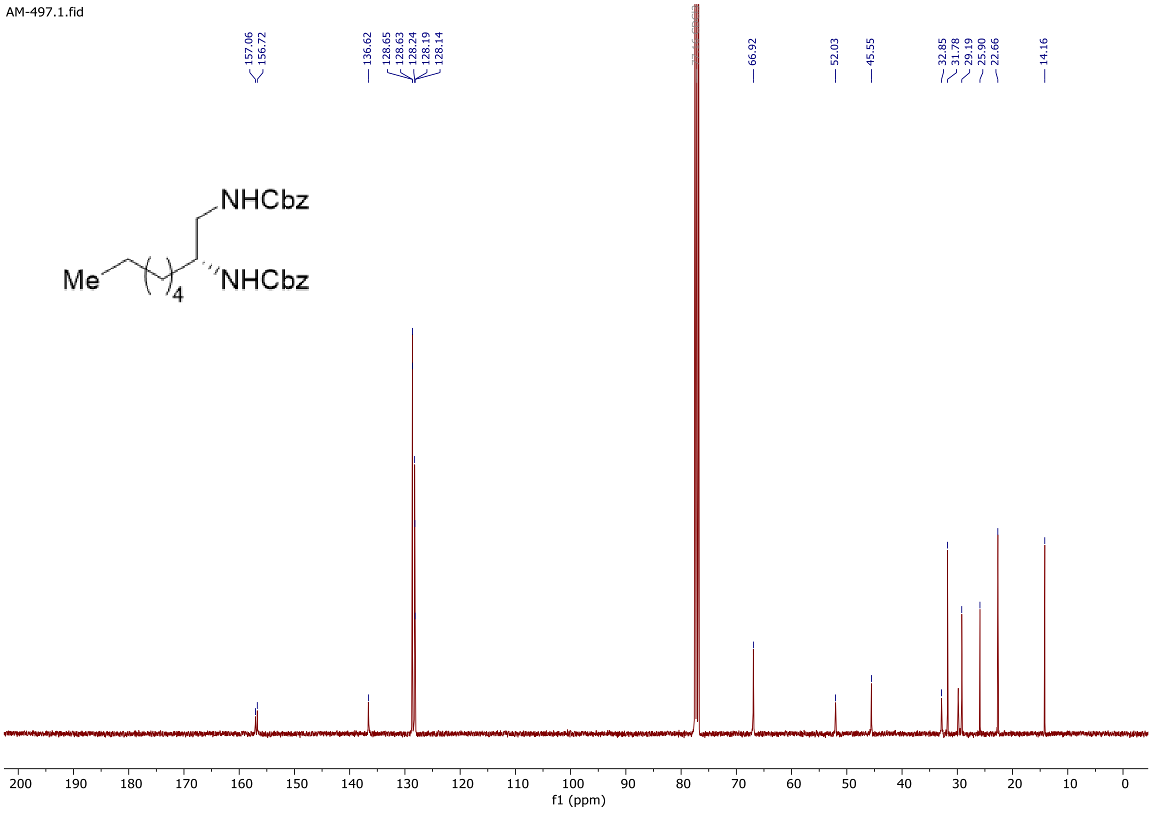

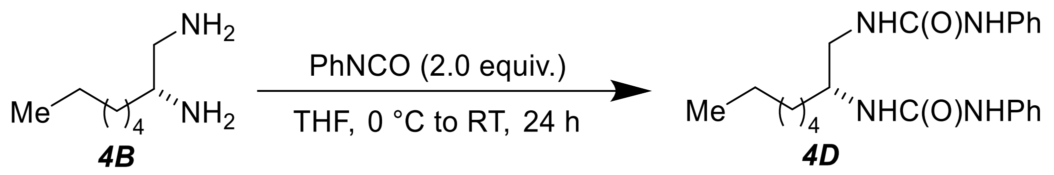

A 10 mL microwave vial equipped with a magnetic stir bar was charged with **4B** (21.6 mg, 0.15 mmol, 1 equiv.) and anhydrous THF (1.5 mL, reaction concentration = 0.1 M). The vial was sealed and cooled to 0 °C using an ice-water bath. Phenyl isocyanate (33 µL, 36 mg, 0.3 mmol, 2 equiv.) was added dropwise to the vial at 0 °C. Then, the stirring reaction mixture was warmed to room temperature over a period of 24 hours. Following this time, the seal was broken, and the reaction mixture was concentrated *in vacuo*. The resulting residue was purified by chromatography on silica gel (gradient of 4 to 40% EtOAc/hexanes) to give **4D** (white solid, 30.4 mg, 0.079 mmol, 53% yield, 82% ee).

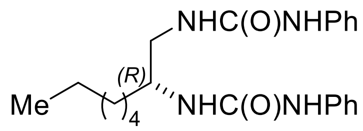

(*R*)-1,1'-(octane-1,2-diyl)bis(3-phenylurea)

Compound 4D:

^{1}H NMR (400 MHz, CD_3OD) δ 7.37 – 7.28 (m, 4H), 7.26 – 7.17 (m, 4H), 6.99 – 6.89 (m, 2H), 3.82 (dq, *J* = 8.6, 4.8 Hz, 1H), 3.40 – 3.33 (m, 1H), 3.20 (dd, *J* = 13.7, 8.0 Hz, 1H), 1.46 – 1.40 (m, 2H), 1.40 – 1.22 (m, 8H), 0.93 – 0.87 (m, 3H).

^{13}C{^{1}H} NMR (101 MHz, CD_3OD) δ 158.6, 158.3, 140.88, 140.86, 129.8, 123.5, 123.4, 120.4, 120.3, 51.7, 45.3, 34.1, 32.9, 30.3, 27.0, 23.7, 14.4.

IR ν 3054, 2987, 2306, 1633, 1550, 1437, 1422, 1266, 896, 742, 706 cm^{-1}.

HRMS (ESI) m/z = $[M + Na]^+$ Calcd $C_{22}H_{30}N_4O_2Na^+$ 405.2266. Found 405.2252 (3.5 ppm error).

Specific Rotation: $[\alpha]_D^{22}$ = +20.9 (c = 0.75 g/100 mL, MeOH, 82% ee).

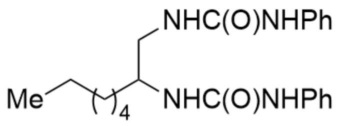

Racemic Sample: HPLC (OD-H, Isopropanol/hexanes = 02/98, flow rate = 0.5 mL/min, I = 254 nm), t_R = 26.2 min, 41.6 min.

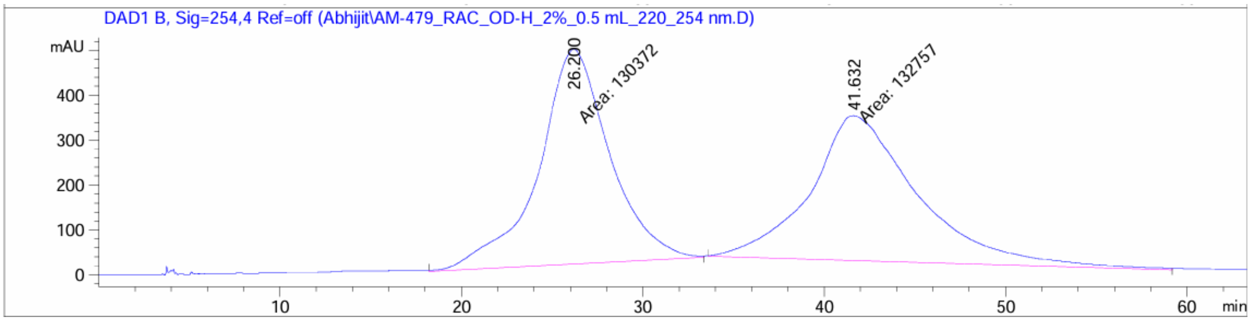

Peak #	RetTime [min]	Type	Width [min]	Area [mAU*s]	Height [mAU]	Area %
1	26.200	MM	4.5404	1.30372e5	478.56528	49.5466
2	41.632	MM	6.8684	1.32757e5	322.14459	50.4534

Scalemic Sample, +82% ee: HPLC (OD-H, Isopropanol/hexanes = 02/98, flow rate = 0.5 mL/min, I = 254 nm), t_R = 26.0 min, 44.2 min.

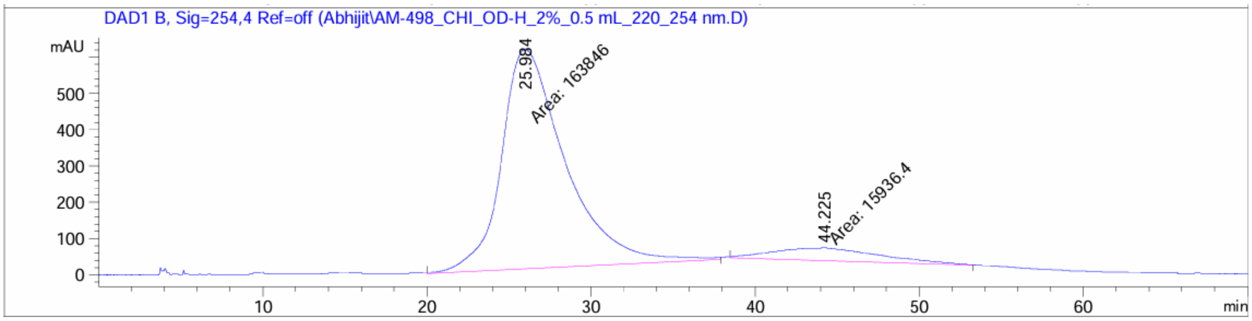

Peak #	RetTime [min]	Type	Width [min]	Area [mAU*s]	Height [mAU]	Area %
1	25.984	MM	3.1787	1.63846e5	605.24268	91.1357
2	44.225	MM	7.5374	1.59364e4	35.23859	8.8643

Compound 4D (CD$_3$OD, ^{1}H NMR: 400 MHz, ^{13}C{^{1}H} NMR: 101 MHz)

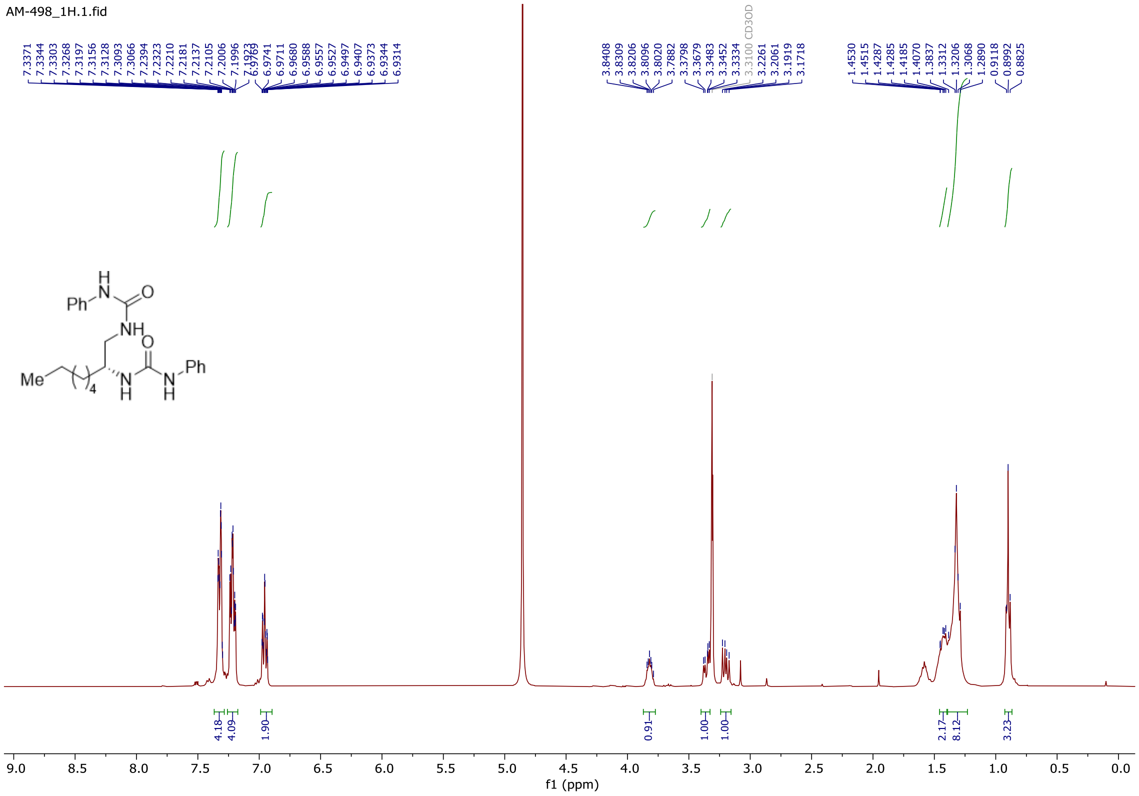

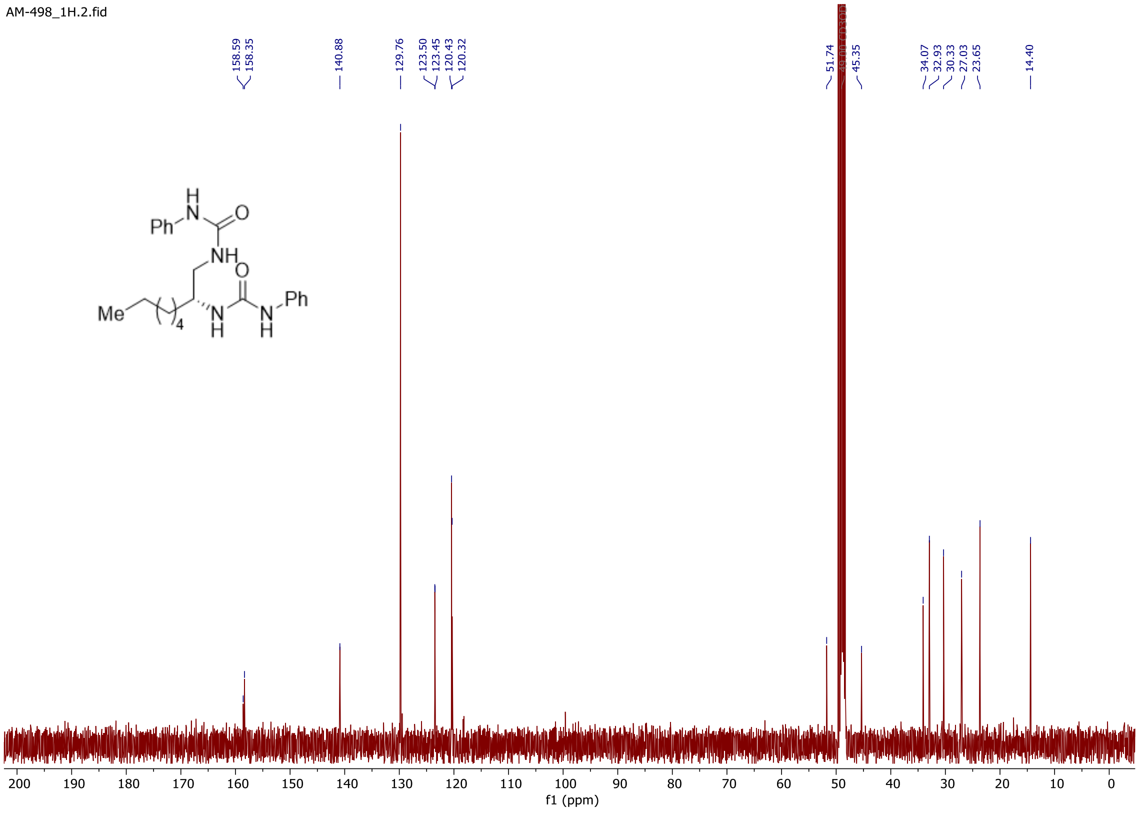

VI. Structural Reasoning

The absolute stereochemistry of:

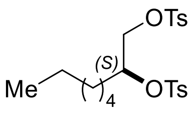

2A

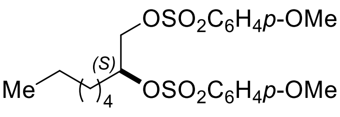

2B

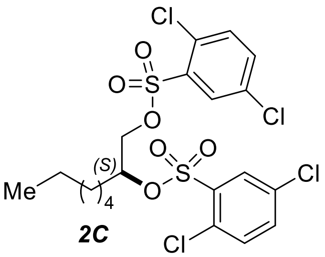

2C

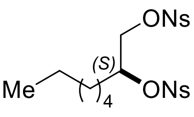

2D

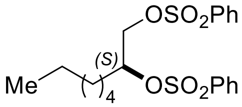

2E

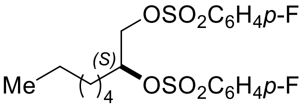

2F

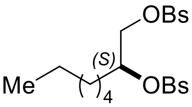

2G

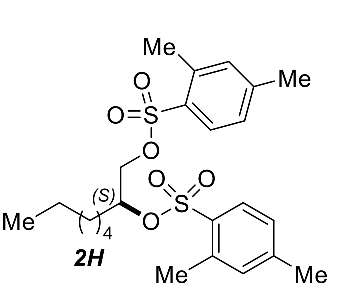

2H

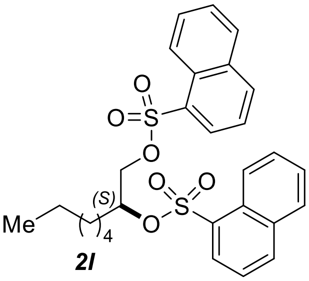

2I

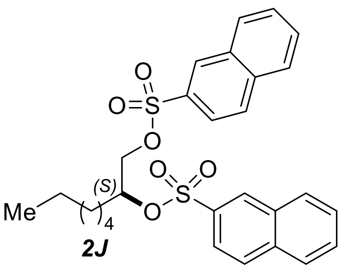

2J

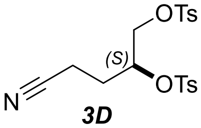

3D

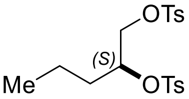

3L

has been assigned by analogy to an authentic sample of:

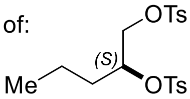

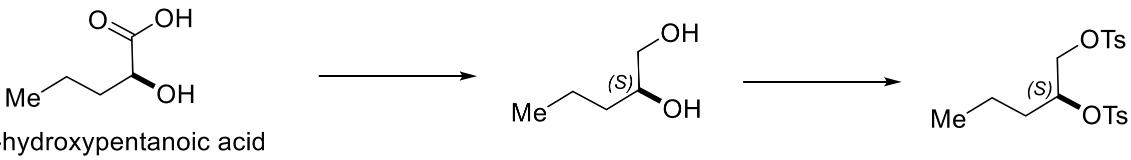

(S)-2-hydroxypentanoic acid
commercial

The authentic sample was prepared according to a literature procedure using for similar compounds (*Synthesis* **1981**, 76 – 78).

Authentic Sample

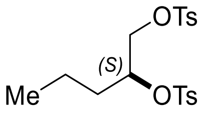

Specific Rotation: $[\alpha]_D^{24}$ = –12.4 (c = 7.9 g/100 mL, $CHCl_3$) (Authentic sample)

Racemic Sample: HPLC (IC-3, Isopropanol/hexanes = 20/80, flow rate = 1 mL/min, I = 254 nm), t_R = 39.7 min, 44.3 min.

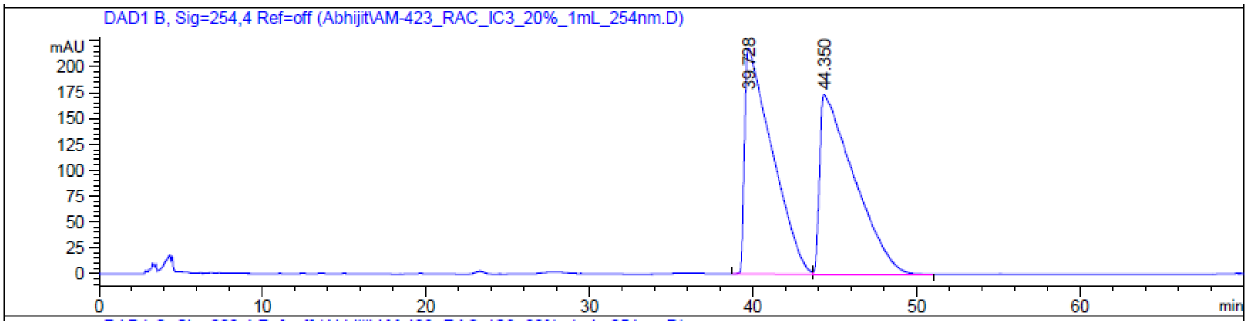

Peak #	RetTime [min]	Type	Width [min]	Area [mAU*s]	Height [mAU]	Area %
1	39.728	BV	1.5064	2.53108e4	217.38399	49.9646
2	44.350	VB	1.8166	2.53466e4	173.07399	50.0354

Authentic Sample, >99% ee: HPLC (IC-3, Isopropanol/hexanes =20/80, flow rate = 1 mL/min, I = 254 nm), t_R = 34.4 min.

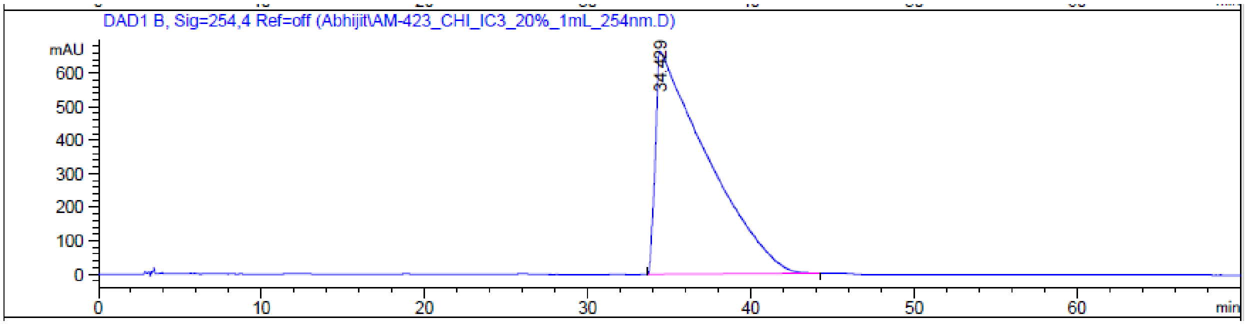

Peak #	RetTime [min]	Type	Width [min]	Area [mAU*s]	Height [mAU]	Area %
1	34.429	BB	2.7351	1.46985e5	665.91772	100.0000

Synthetic Scalemic Sample

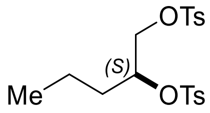

Specific Rotation: $[\alpha]_D^{24}$ = –11.2 (c = 2.2 g/100 mL, $CHCl_3$) (Synthetic scalemic sample)

Racemic Sample: HPLC (IC-3, Isopropanol/hexanes = 20/80, flow rate = 1 mL/min, I = 254 nm), t_R = 42.9 min, 47.6 min.

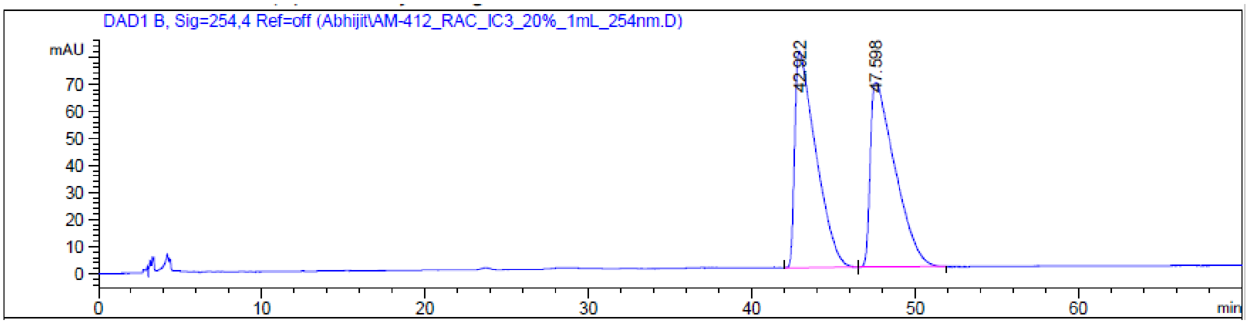

Peak #	RetTime [min]	Type	Width [min]	Area [mAU*s]	Height [mAU]	Area %
1	42.922	BB	1.2197	7147.93896	79.90186	50.0029
2	47.598	BB	1.3728	7147.11426	68.18777	49.9971

Scalemic Sample, +82% ee: HPLC (IC-3, Isopropanol/hexanes =20/80, flow rate = 1 mL/min, I = 254 nm), t_R = 38.6 min, 46.7 min.

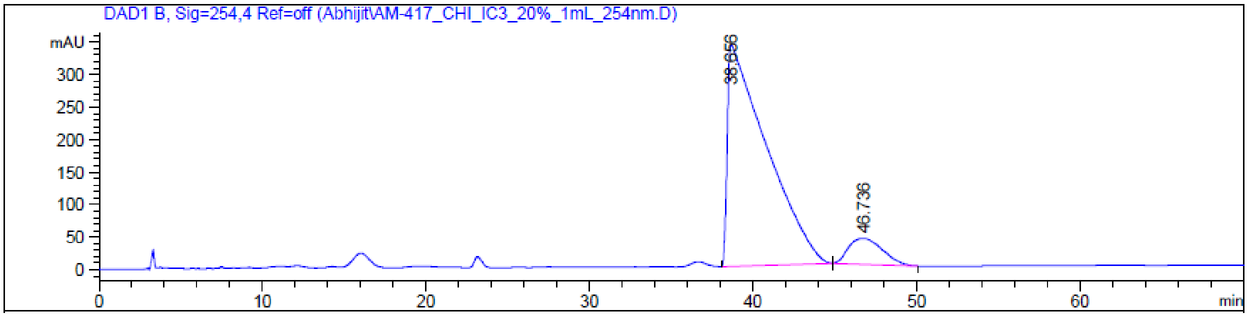

Peak #	RetTime [min]	Type	Width [min]	Area [mAU*s]	Height [mAU]	Area %
1	38.656	BB	2.0616	5.67003e4	341.05994	90.7946
2	46.736	BB	1.6935	5748.68115	39.98056	9.2054

CDCl$_3$, 400 MHz

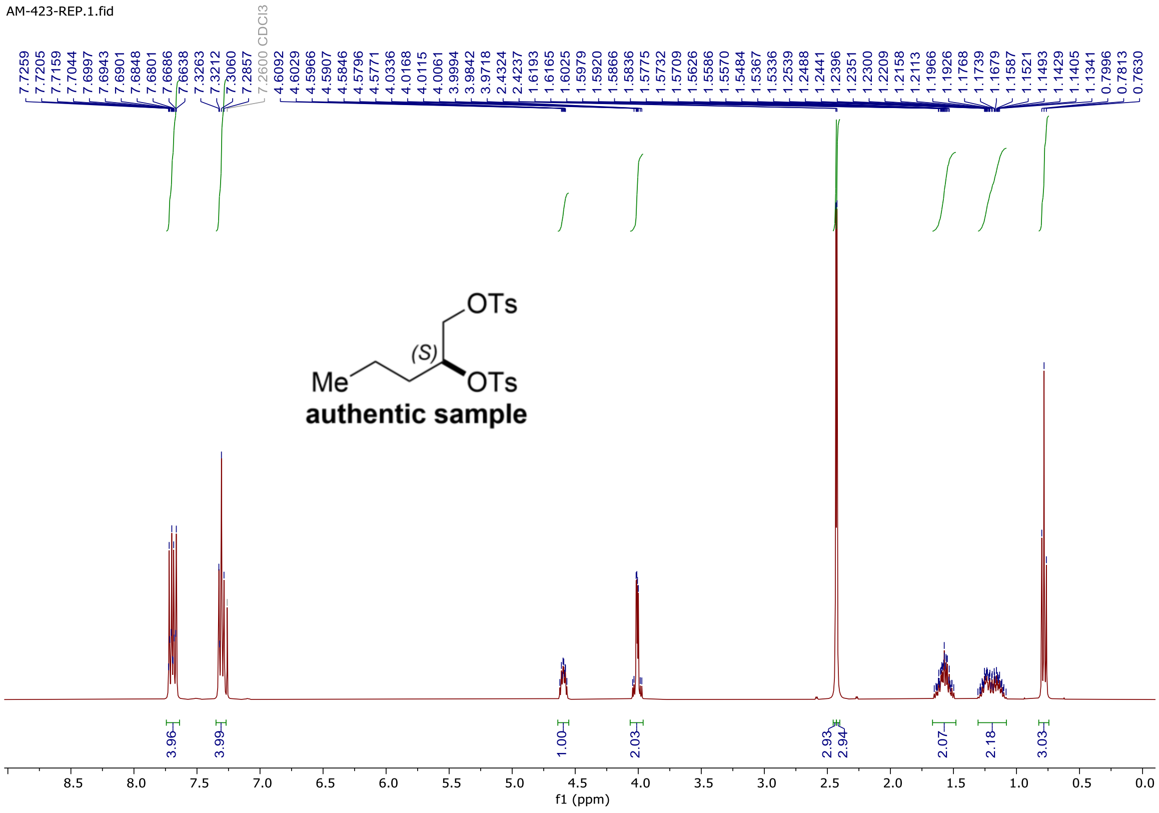

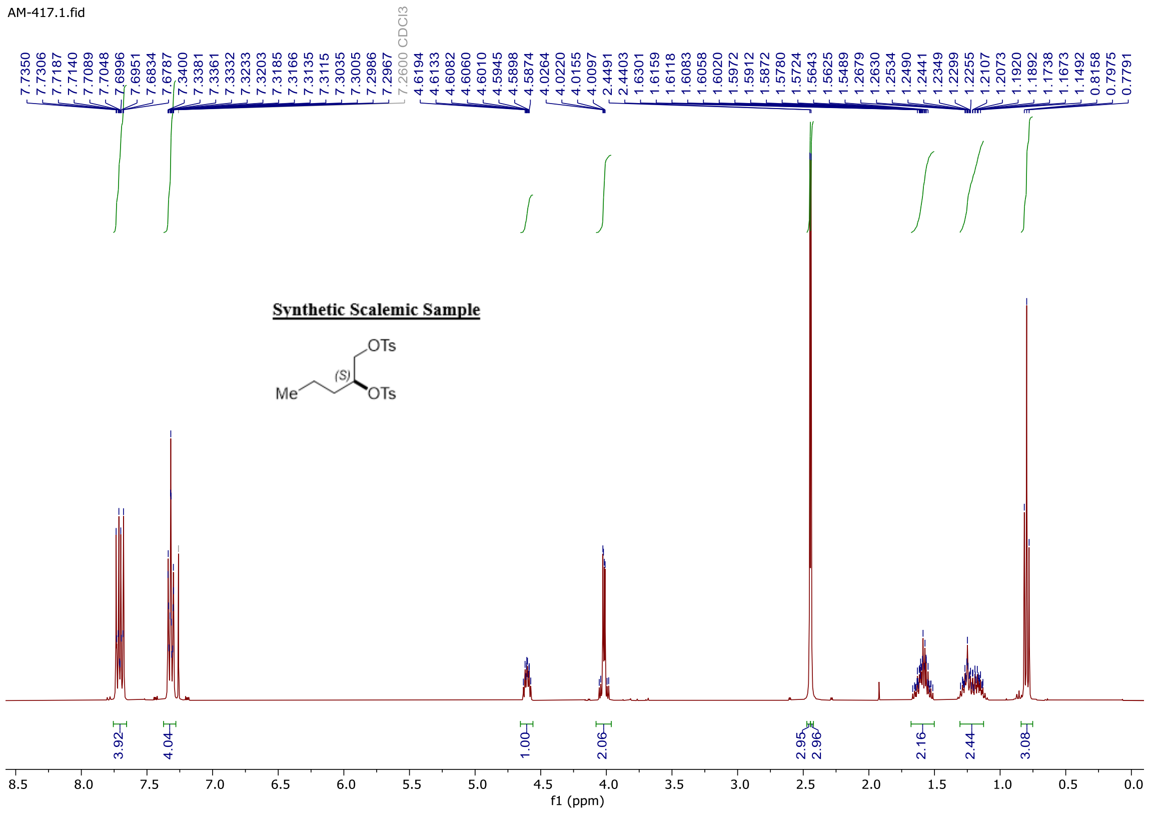

The absolute stereochemistry of:

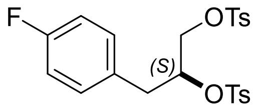

3T

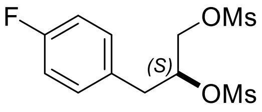

3U

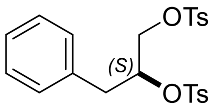

3V

has been assigned by analogy to an authentic sample of:

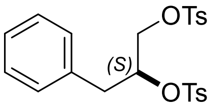

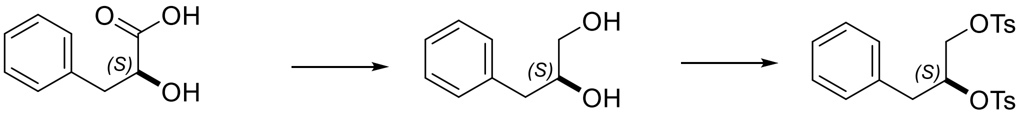

(*S*)-2-hydroxy-3-phenylpropanoic acid
commercial

The authentic sample was prepared according to a literature procedure (*Synthesis* **1981**, 76 – 78).

Authentic Sample

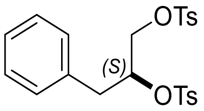

Specific Rotation: $[\alpha]_D^{23}$ = –11.3 (c = 1.3 g/100 mL, $CHCl_3$) [Authentic sample]

Racemic Sample: HPLC (IA-3, Isopropanol/hexanes = 5/95, flow rate = 0.6 mL/min, I = 220 nm), t_R = 53.4 min, 59.9 min.

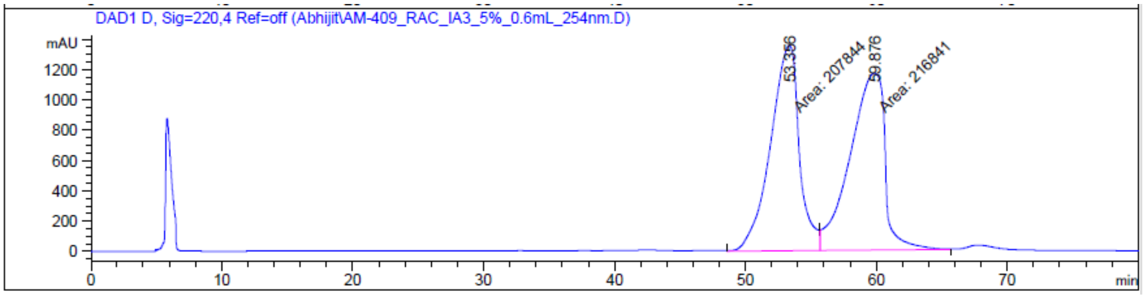

Peak #	RetTime [min]	Type	Width [min]	Area [mAU*s]	Height [mAU]	Area %
1	53.356	MF	2.5582	2.07844e5	1354.08521	48.9407
2	59.876	FM	3.0961	2.16841e5	1167.27368	51.0593

Authentic Sample, >99% ee: HPLC (IA-3, Isopropanol/hexanes = 5/95, flow rate = 0.6 mL/min, I = 220 nm), t_R = 54.0 min, 65.3 min.

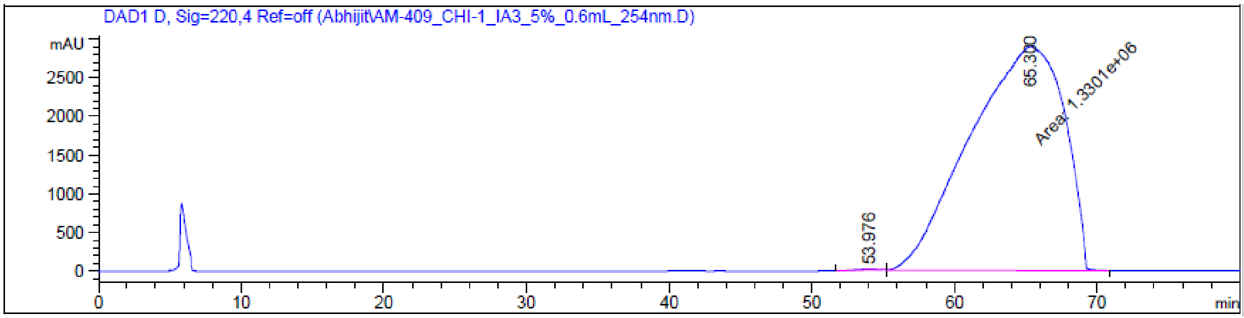

Peak #	RetTime [min]	Type	Width [min]	Area [mAU*s]	Height [mAU]	Area %
1	53.976	BB	1.1890	1319.03101	13.09671	0.0991
2	65.300	MM	7.6547	1.33010e6	2896.02002	99.9009

__Synthetic Scalemic Sample__

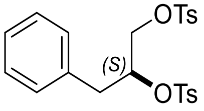

Specific Rotation: $[\alpha]_D^{22}$ = –3.18 (c = 1.2 g/100 mL, $CHCl_3$) (Synthetic scalemic sample)

Racemic Sample: HPLC (IA-3, Isopropanol/hexanes = 5/95, flow rate = 0.6 mL/min, l = 220 nm), t_R = 53.1 min, 60.1 min.

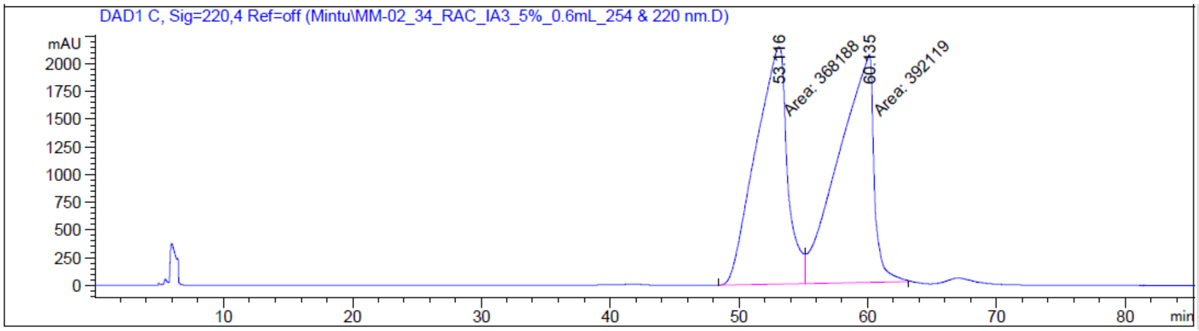

Peak #	RetTime [min]	Type	Width [min]	Area [mAU*s]	Height [mAU]	Area %
1	53.116	MF	2.8605	3.68188e5	2145.22388	48.4262
2	60.135	FM	3.1728	3.92119e5	2059.77612	51.5738

Scalemic Sample, -22% ee: HPLC (IA-3, Isopropanol/hexanes = 5/95, flow rate = 0.6 mL/min, l = 220 nm), t_R = 54.7 min, 62.8 min.

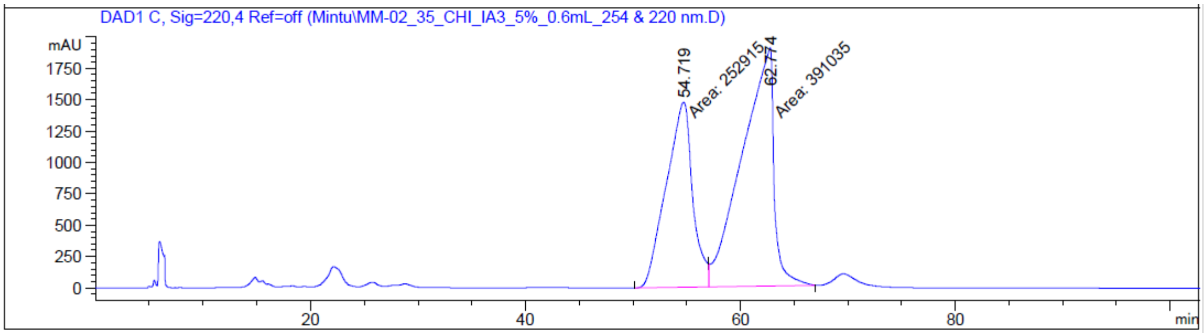

Peak #	RetTime [min]	Type	Width [min]	Area [mAU*s]	Height [mAU]	Area %
1	54.719	MF	2.8588	2.52915e5	1474.45898	39.2755
2	62.774	FM	3.4349	3.91035e5	1897.38855	60.7245

$CDCl_3$, 400 MHz

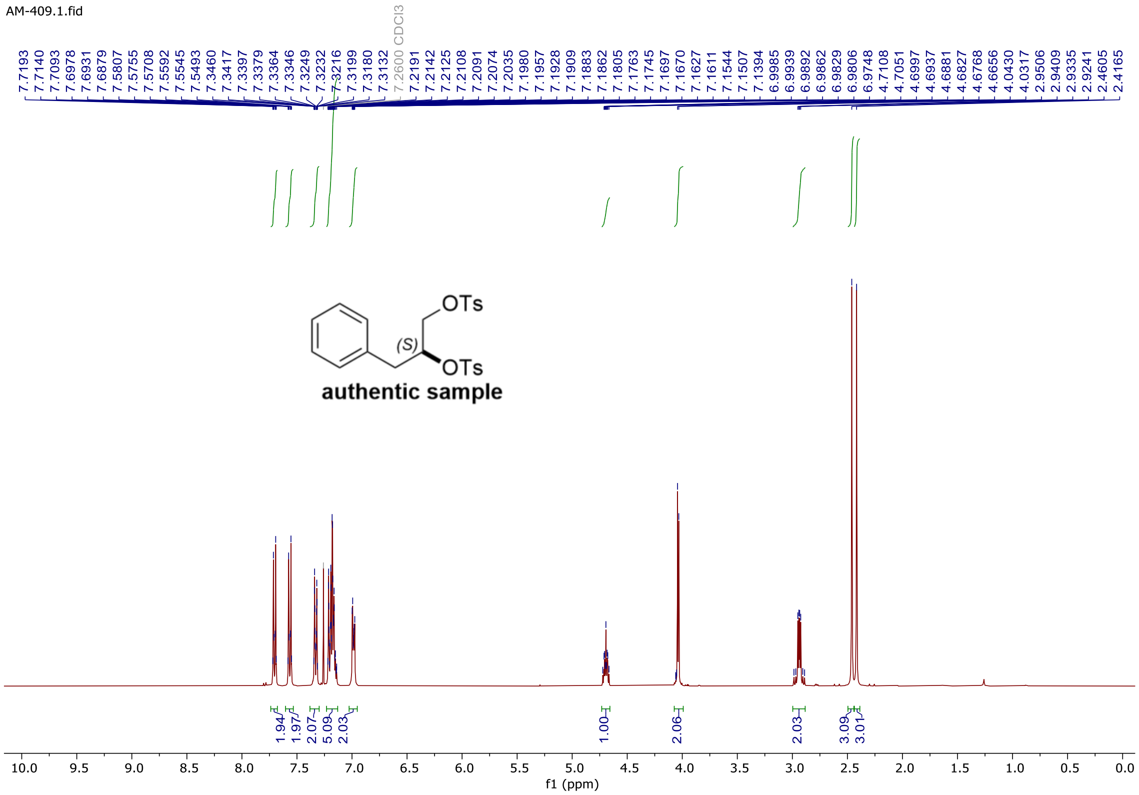

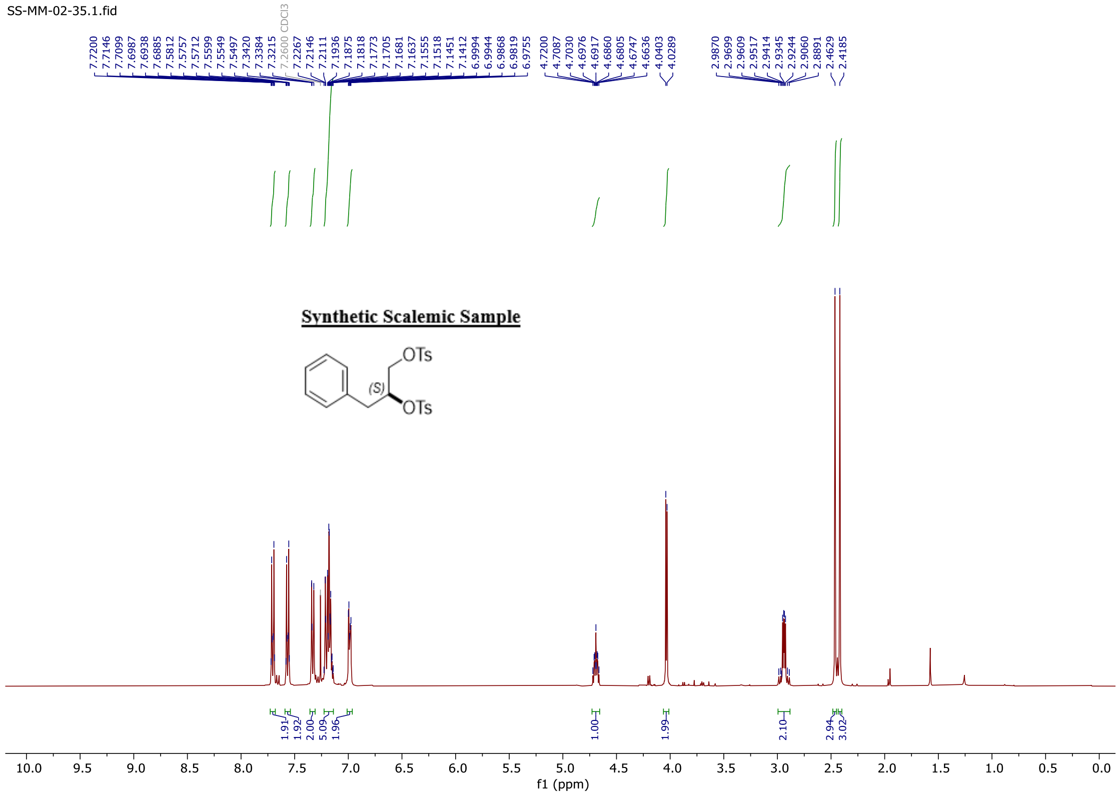

The absolute stereochemistry of:

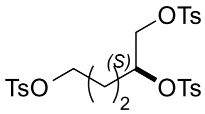

3J

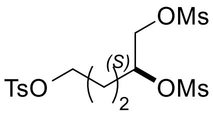

3K

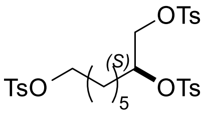

3M

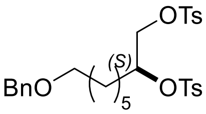

3N

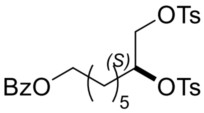

3O

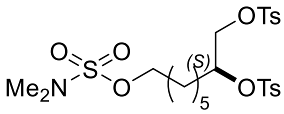

3P

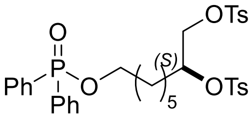

3Q

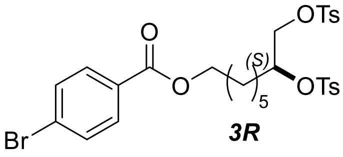

3R

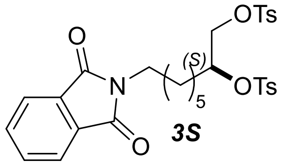

3S

has been assigned by analogy to an authentic sample of:

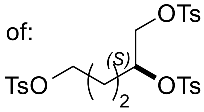

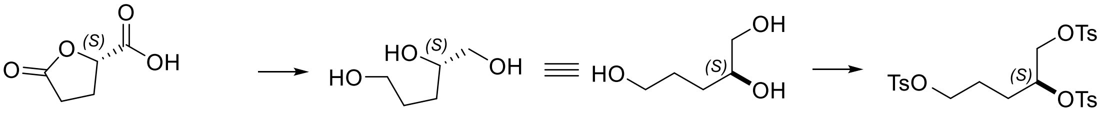

(S)-5-Oxo-tetrahydrofuran-2-carboxylic acid
commercial

The authentic sample was prepared according to a literature procedure (*Synthesis* **1989**, 706 – 709).

Authentic Sample

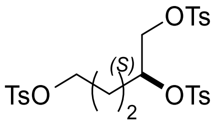

Specific Rotation: $[\alpha]_D^{22}$ = –5.3 (c = 3.8 g/100 mL, $CHCl_3$) [Authentic sample]

Racemic Sample: HPLC (IC-3, Isopropanol/hexanes = 50/50, flow rate = 1 mL/min, I = 254 nm), t_R = 134.4 min, 151.7 min.

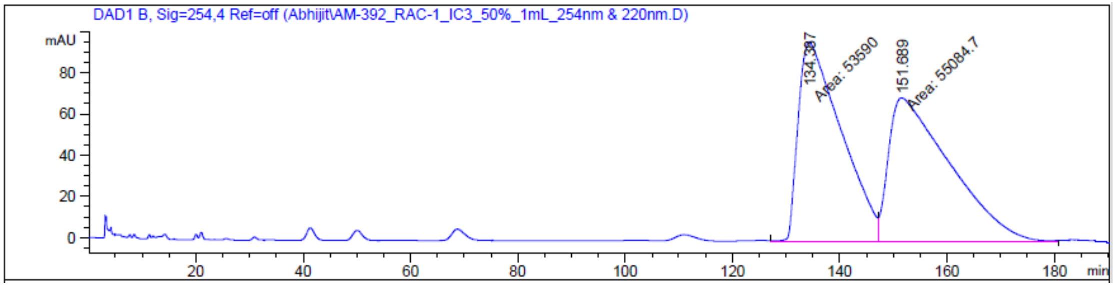

Peak #	RetTime [min]	Type	Width [min]	Area [mAU*s]	Height [mAU]	Area %
1	134.387	MF	9.2094	5.35900e4	96.98395	49.3123
2	151.689	FM	13.1915	5.50847e4	69.59633	50.6877

Authentic Sample, >99% ee: HPLC (IC-3, Isopropanol/hexanes =50/50, flow rate = 1 mL/min, I = 254 nm), t_R = 138.1 min.

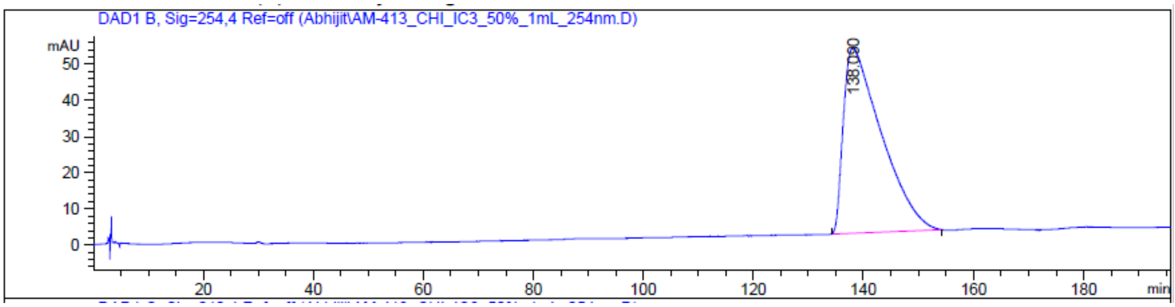

Peak #	RetTime [min]	Type	Width [min]	Area [mAU*s]	Height [mAU]	Area %
1	138.090	BB	5.6055	2.45611e4	51.22876	100.0000

Synthetic Scalemic Sample

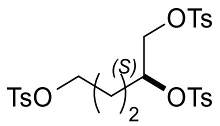

Specific Rotation: $[\alpha]_D^{25}$ = –8.3 (c = 2.9 g/100 mL, $CHCl_3$) (Synthetic scalemic sample)

Racemic Sample: HPLC (IC-3, Isopropanol/hexanes = 50/50, flow rate = 1 mL/min, I = 254 nm), t_R = 134.4 min, 151.7 min.

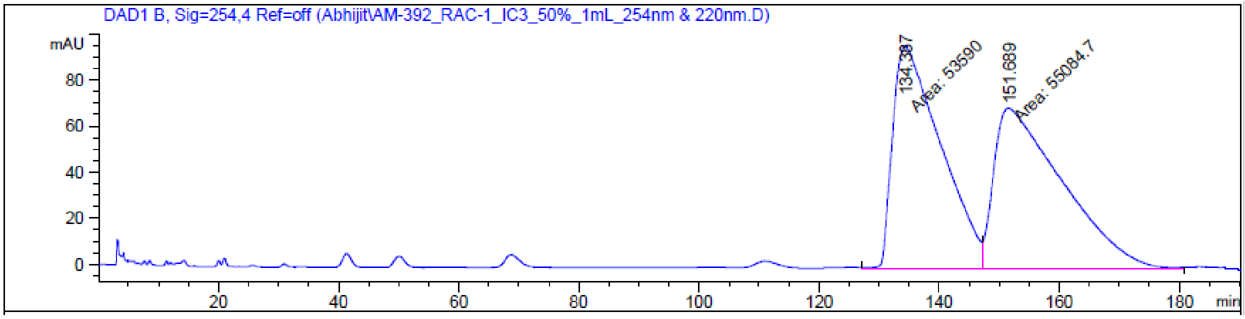

Peak #	RetTime [min]	Type	Width [min]	Area [mAU*s]	Height [mAU]	Area %
1	134.387	MF	9.2094	5.35900e4	96.98395	49.3123
2	151.689	FM	13.1915	5.50847e4	69.59633	50.6877

Scalemic Sample, +93% ee: HPLC (IC-3, Isopropanol/hexanes =50/50, flow rate = 1 mL/min, I = 254 nm), t_R = 132.9 min, 163.7 min.

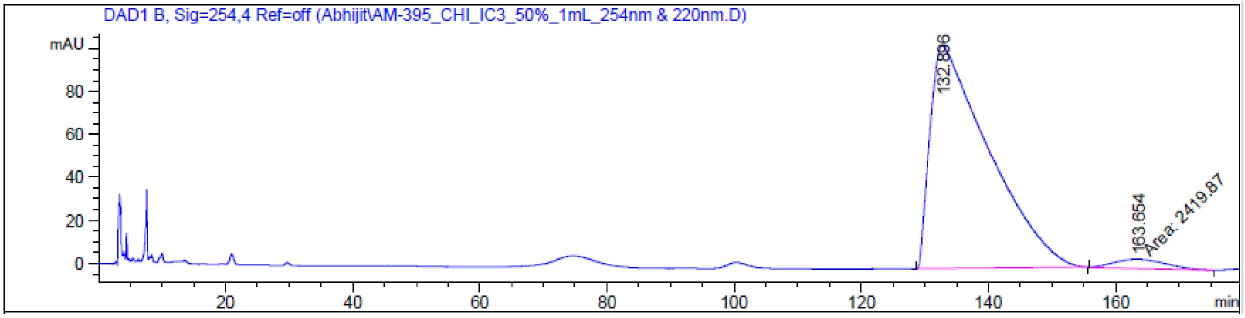

Peak #	RetTime [min]	Type	Width [min]	Area [mAU*s]	Height [mAU]	Area %
1	132.896	BB	7.5136	6.66341e4	103.82426	96.4957
2	163.654	MM	9.3190	2419.86792	4.32785	3.5043

$CDCl_3$, 400 MHz

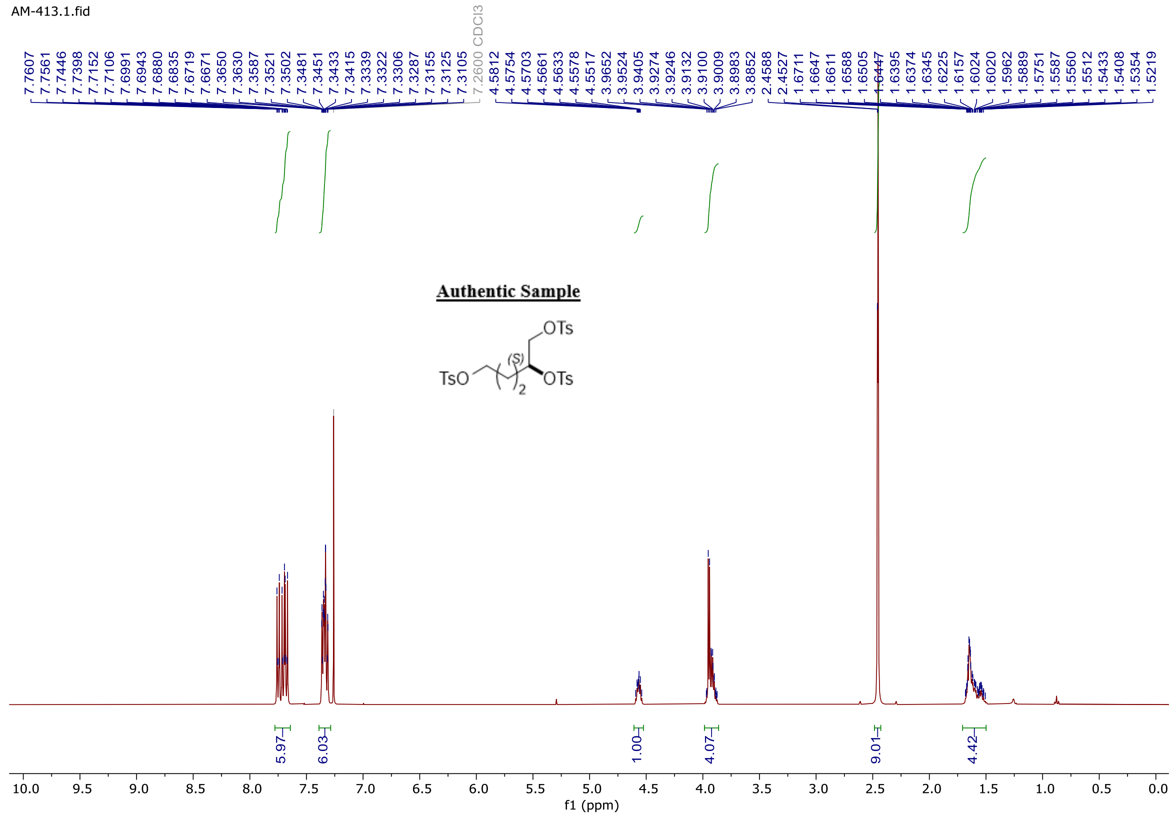

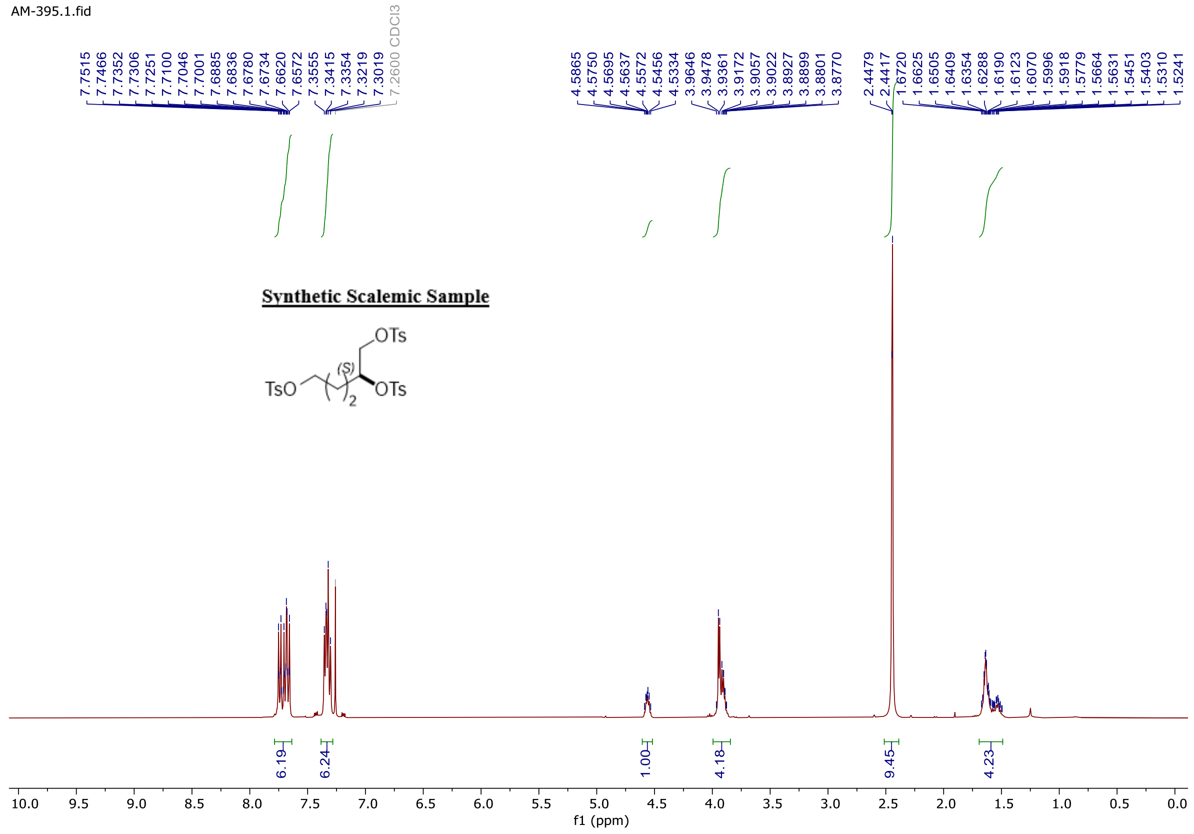

The absolute stereochemistry of:

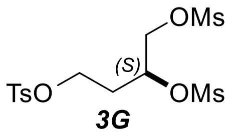

3G

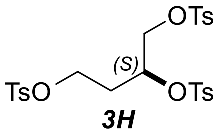

3H

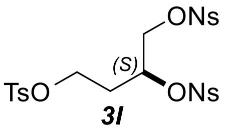

3I

has been assigned by analogy to an authentic sample of:

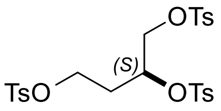

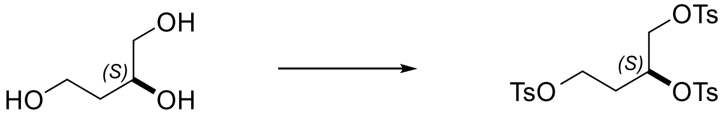

(*S*)-butane-1,2,4-triol
commercial

The authentic sample was prepared according to a literature procedure (*Synthesis* **1989***,* 706 – 709*)*.

Authentic Sample

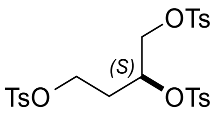

Specific Rotation: $[\alpha]_D^{24}$ = –23.2 (c = 1.1 g/100 mL, $CHCl_3$) [Authentic sample]

Racemic Sample: HPLC (IA-3, Isopropanol/hexanes = 10/90, flow rate = 1 mL/min, I = 254 nm), t_R = 61.7 min, 72.8 min.

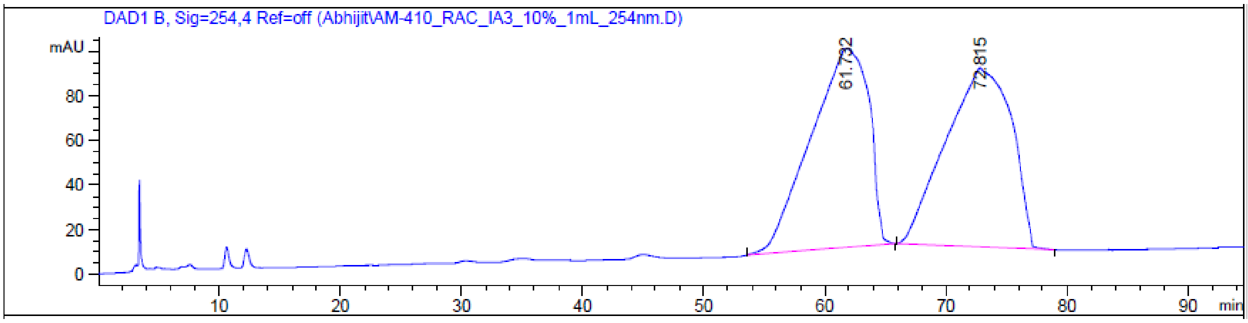

Peak #	RetTime [min]	Type	Width [min]	Area [mAU*s]	Height [mAU]	Area %
1	61.732	BB	3.8932	2.97289e4	89.22466	49.3575
2	72.815	BB	4.5012	3.05029e4	80.23660	50.6425

Authentic Sample, >99% ee: HPLC (IA-3, Isopropanol/hexanes = 10/90, flow rate = 1 mL/min, I = 254 nm), t_R = 68.3 min.

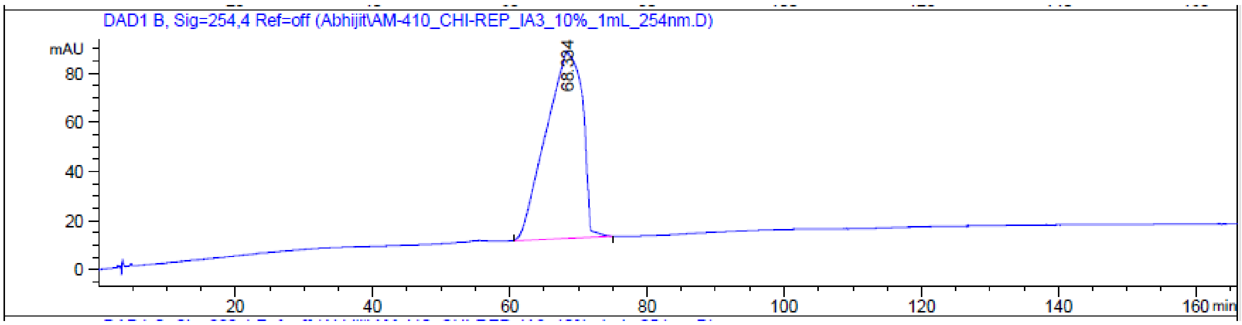

Peak #	RetTime [min]	Type	Width [min]	Area [mAU*s]	Height [mAU]	Area %
1	68.334	BB	4.3328	2.79768e4	76.31833	100.0000

Synthetic Scalemic Sample

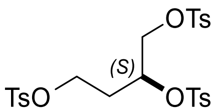

Specific rotation: $[\alpha]_D^{21} = -19.1$ (c = 1.1 g/100 mL, $CHCl_3$) (Synthetic Scalemic Sample)

Racemic Sample: HPLC (IA-3, Isopropanol/hexanes = 10/90, flow rate = 1 mL/min, I = 254 nm), t_R = 65.3 min, 77.0 min.

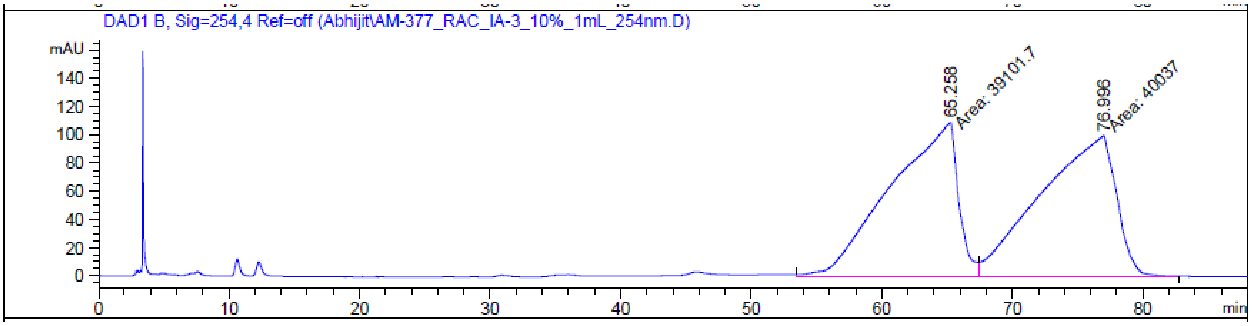

Peak #	RetTime [min]	Type	Width [min]	Area [mAU*s]	Height [mAU]	Area %
1	65.258	MF	5.9610	3.91017e4	109.32719	49.4091
2	76.996	FM	6.6817	4.00370e4	99.86734	50.5909

Scalemic Sample, -69% ee: HPLC (IA-3, Isopropanol/hexanes = 10/90, flow rate = 1 mL/min, I = 254 nm), t_R = 62.5 min, 77.4 min.

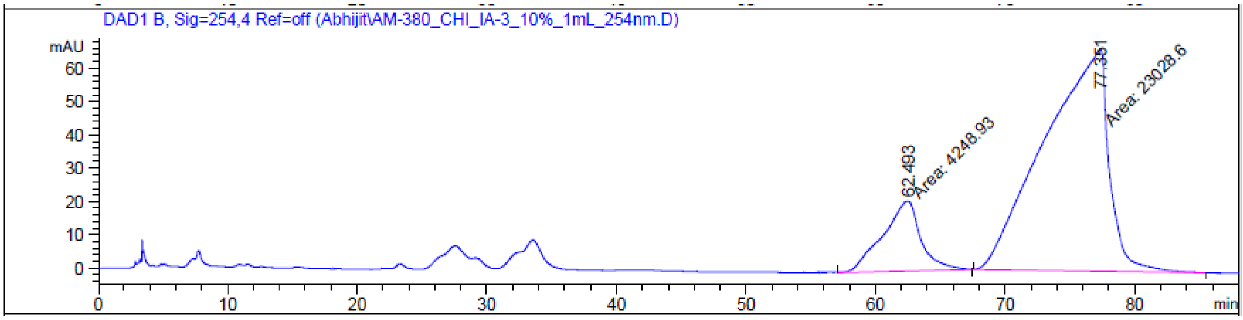

Peak #	RetTime [min]	Type	Width [min]	Area [mAU*s]	Height [mAU]	Area %
1	62.493	MM	3.3898	4248.92969	20.89062	15.5767
2	77.351	MF	5.7607	2.30286e4	66.62522	84.4233

CDCl$_3$, 400 MHz

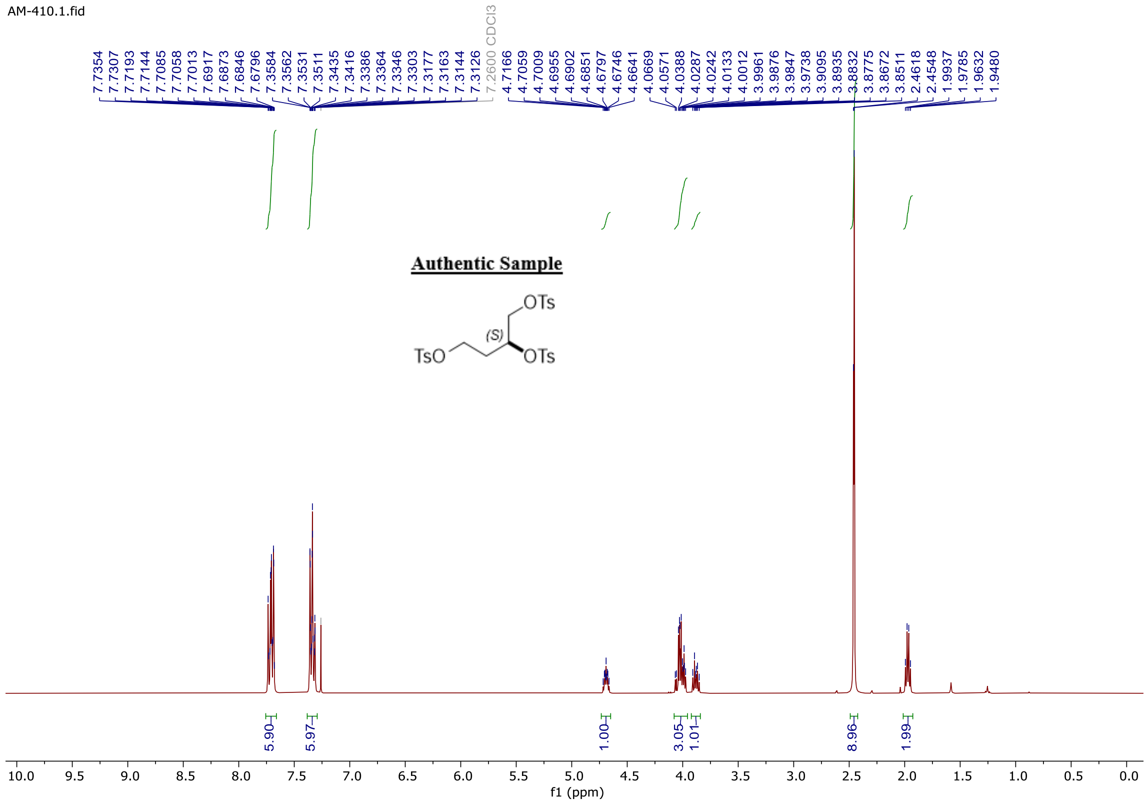

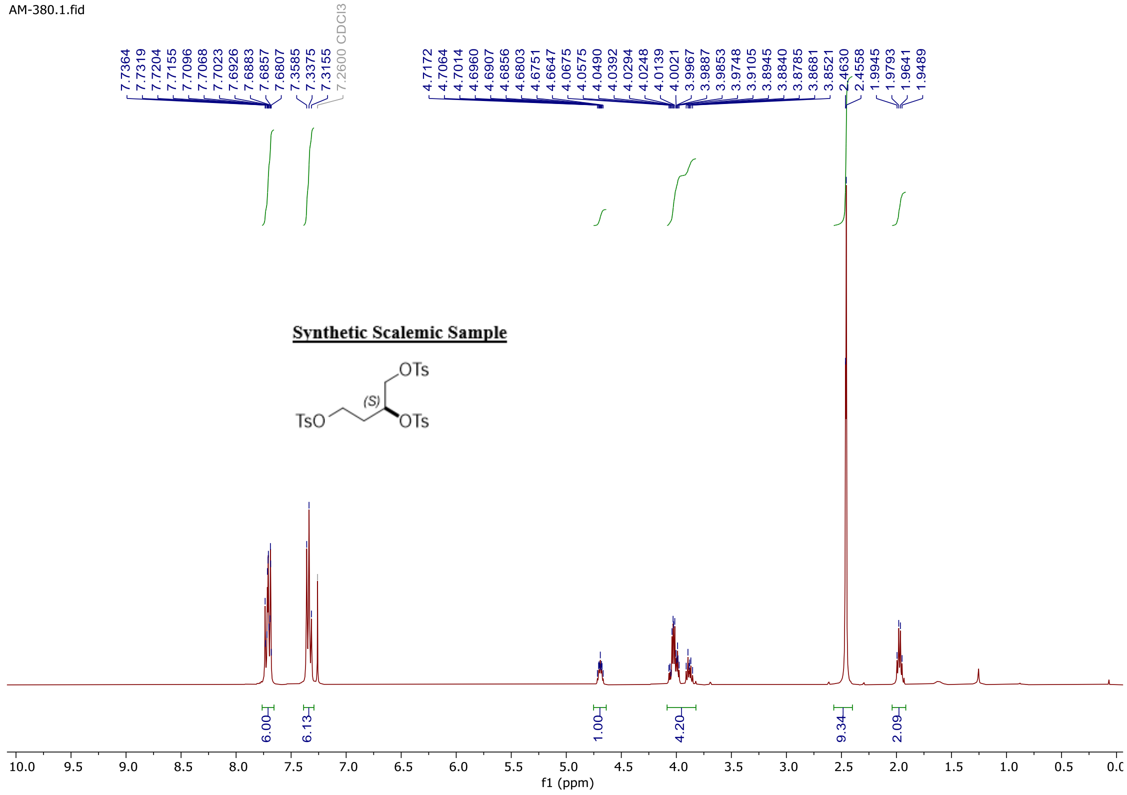

The absolute stereochemistry of:

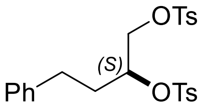

3A

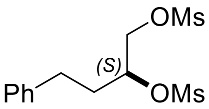

3B

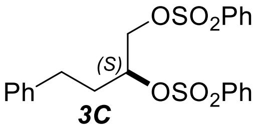

3C

has been assigned by analogy to an authentic sample of:

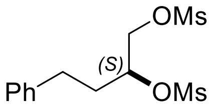

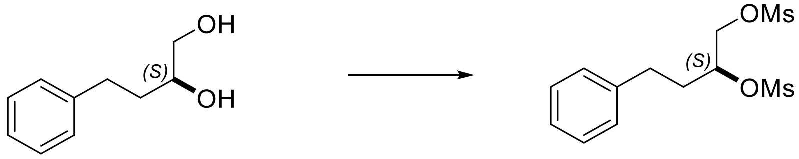

(*S*)-4-phenylbutane-1,2-diol

commercial

The authentic sample was prepared by mesylation of the commercial diol.

Authentic Sample

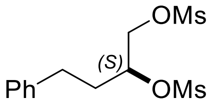

Specific Rotation: $[\alpha]_D^{25}$ = +1.9 (c = 6.1 g/100 mL, $CHCl_3$) (Authentic sample)

Racemic Sample: HPLC (IC-3, Isopropanol/hexanes = 20/80, flow rate = 1 mL/min, I = 254 nm), t_R = 43.5 min, 54.0 min.

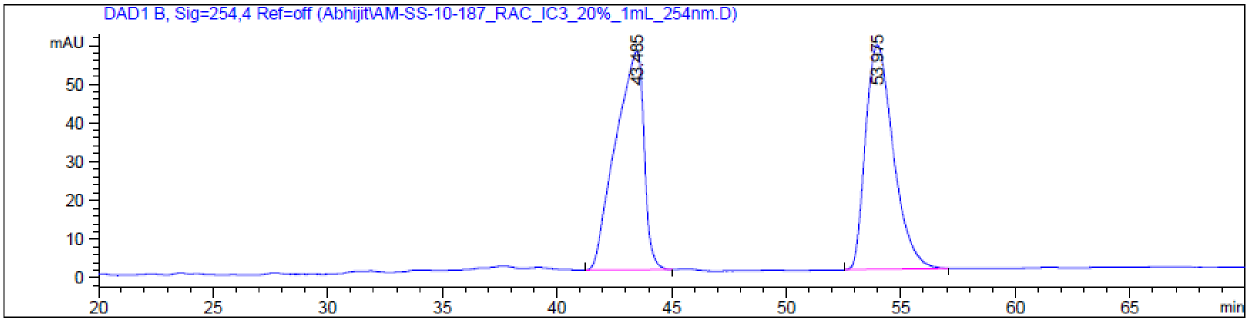

Peak #	RetTime [min]	Type	Width [min]	Area [mAU*s]	Height [mAU]	Area %
1	43.485	BB	1.1768	4846.65186	56.38557	49.7868
2	53.975	BB	1.2830	4888.16064	57.78041	50.2132

Authentic Sample, >99% ee: HPLC (IC-3, Isopropanol/hexanes = 20/80, flow rate = 1 mL/min, I = 254 nm), t_R = 44.2 min.

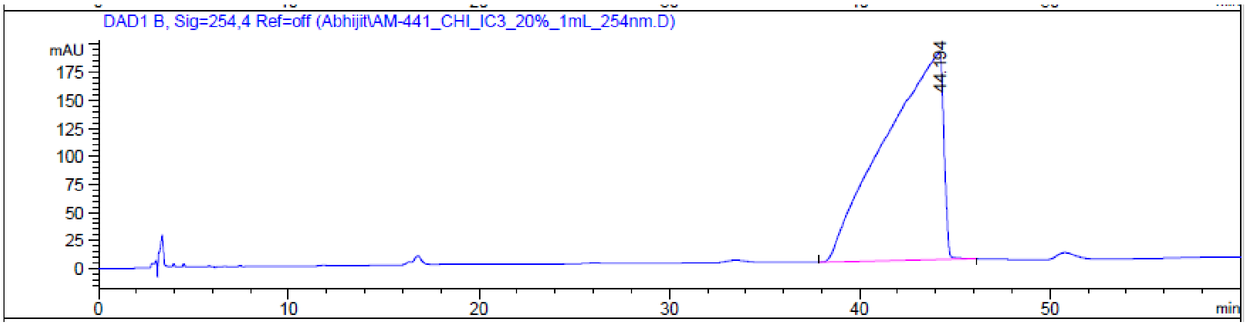

Peak #	RetTime [min]	Type	Width [min]	Area [mAU*s]	Height [mAU]	Area %
1	44.194	BB	2.5575	3.91442e4	184.93164	100.0000

Synthetic Scalemic Sample

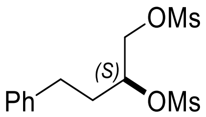

Specific Rotation: $[\alpha]_D^{23}$ = +2.7 (c = 1.6 g/100 mL, $CHCl_3$) (Synthetic Scalemic Sample)

Racemic Sample: HPLC (IC-3, Isopropanol/hexanes = 20/80, flow rate = 1 mL/min, I = 254 nm), t_R = 43.5 min, 54.0 min.

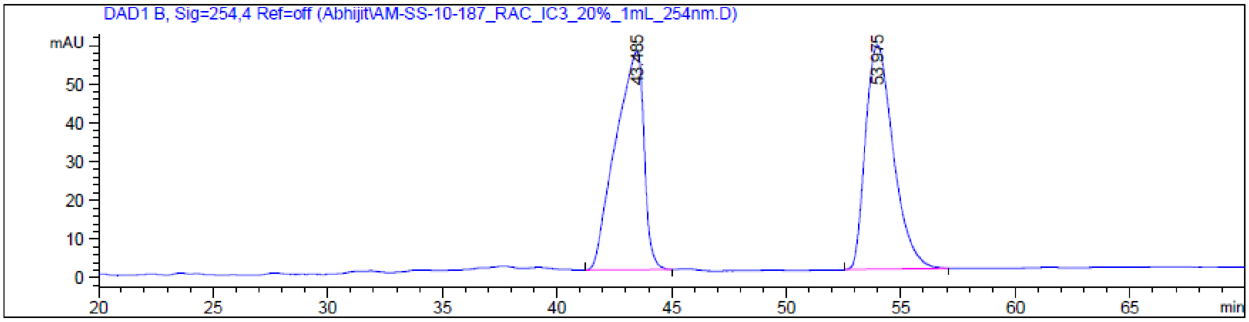

Peak #	RetTime [min]	Type	Width [min]	Area [mAU*s]	Height [mAU]	Area %
1	43.485	BB	1.1768	4846.65186	56.38557	49.7868
2	53.975	BB	1.2830	4888.16064	57.78041	50.2132

Scalemic Sample, +79% ee: HPLC (IC-3, Isopropanol/hexanes = 20/80, flow rate = 1 mL/min, I = 254 nm), t_R = 43.8 min, 54.0 min.

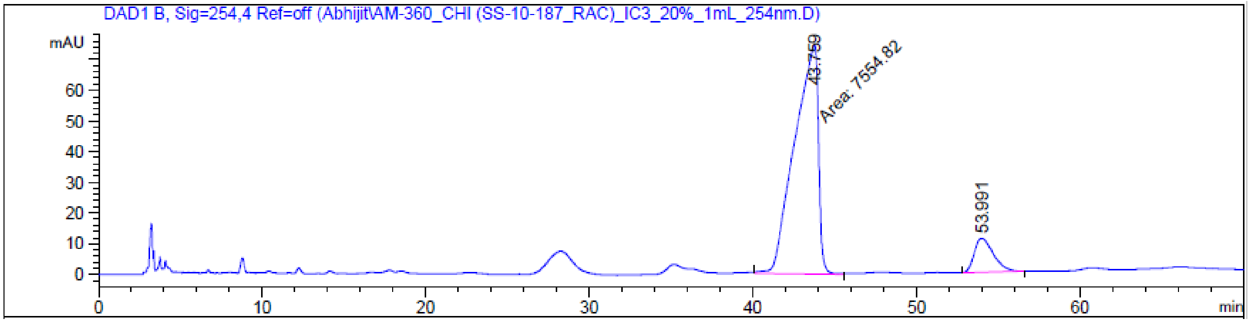

Peak #	RetTime [min]	Type	Width [min]	Area [mAU*s]	Height [mAU]	Area %
1	43.759	MM	1.6878	7554.81689	74.60140	89.2924
2	53.991	BB	0.9790	905.94543	11.13725	10.7076

CDCl$_3$, 400 MHz

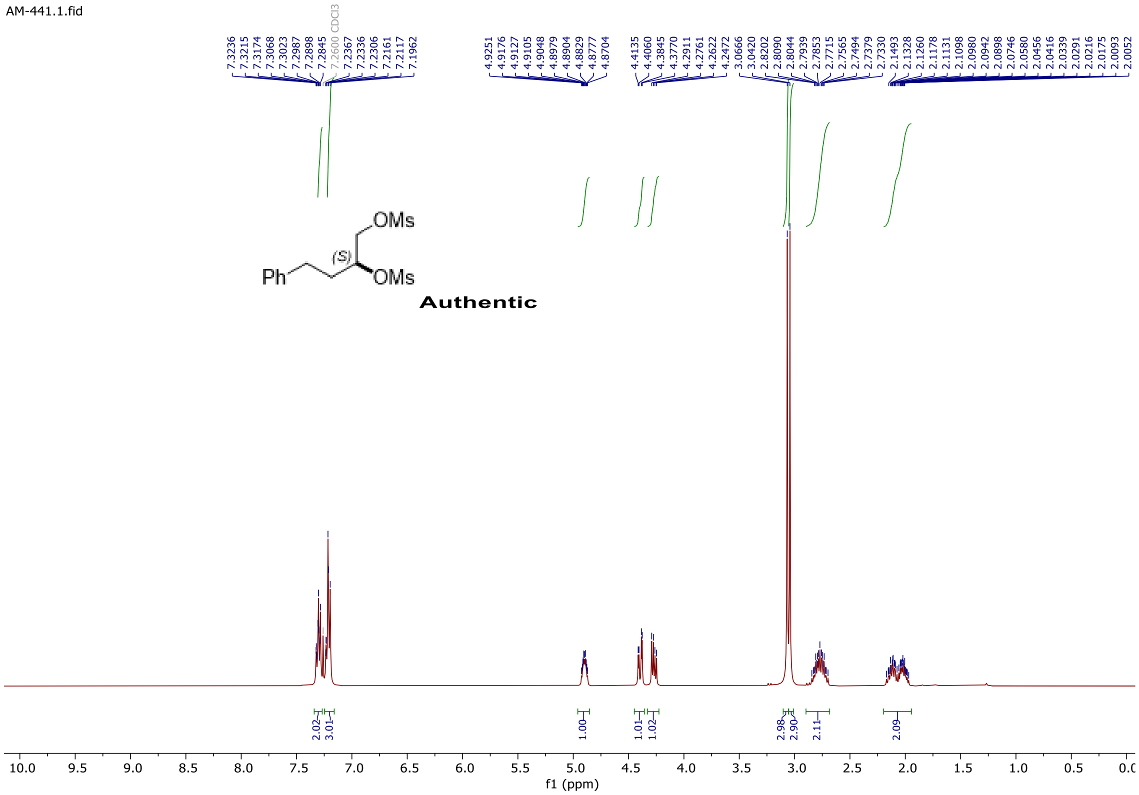

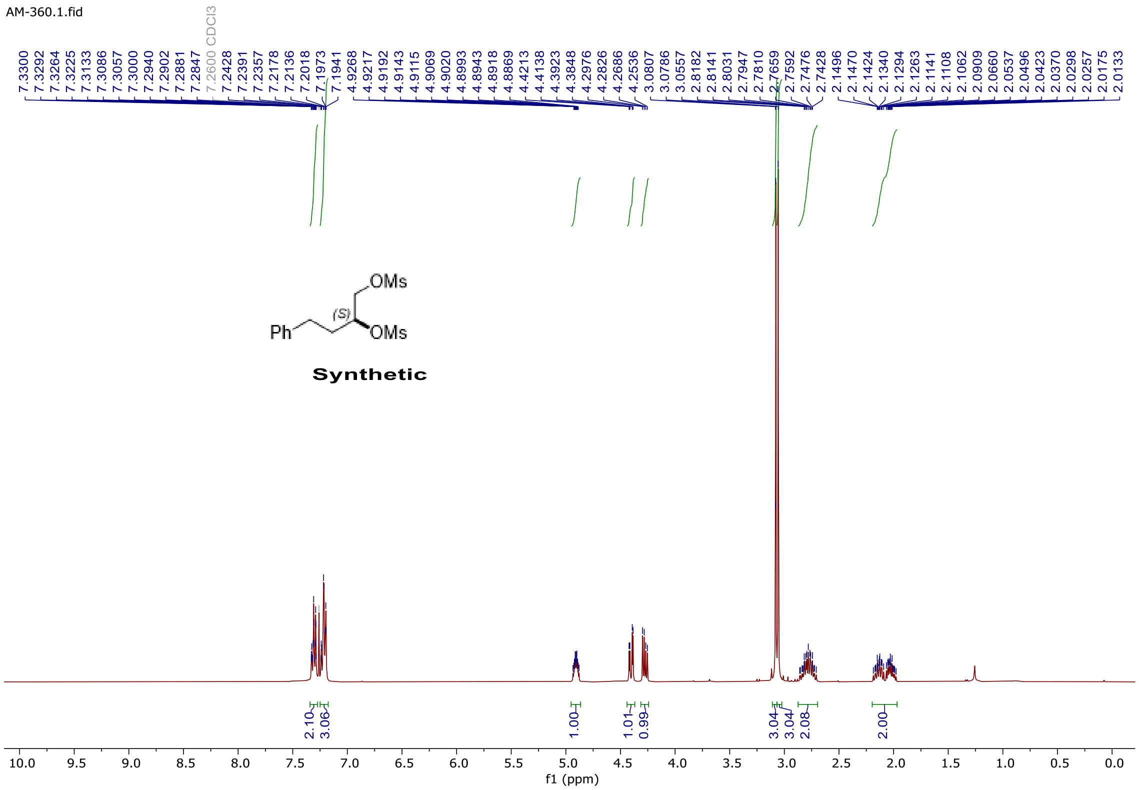

The absolute stereochemistry of:

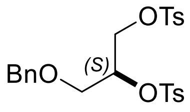

3E

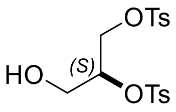

3F

has been assigned by analogy to an authentic sample of:

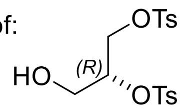

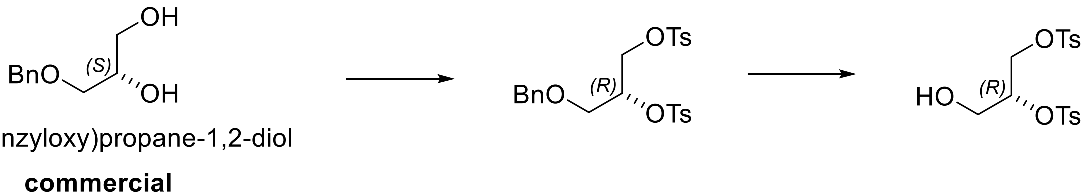

(*S*)-3-(benzyloxy)propane-1,2-diol

commercial

The ditosylate was prepared according to a literature procedure (*J. Org. Chem,* **1982,** *47*, 468 – 473*)*. Removal of the benzyl group proceeded by treatment with Pd/C under 1 atm. of hydrogen gas.

Authentic Sample

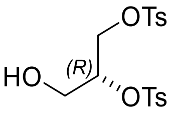

authentic sample

Specific Rotation: $[\alpha]_D^{23}$ = +75.2 (c = 1.4 g/100 mL, $CHCl_3$) (Authentic sample)

Racemic Sample: HPLC (IC-3, Isopropanol/hexanes = 20/80, flow rate = 1 mL/min, I = 254 nm), t_R = 60.0 min, 65.1 min.

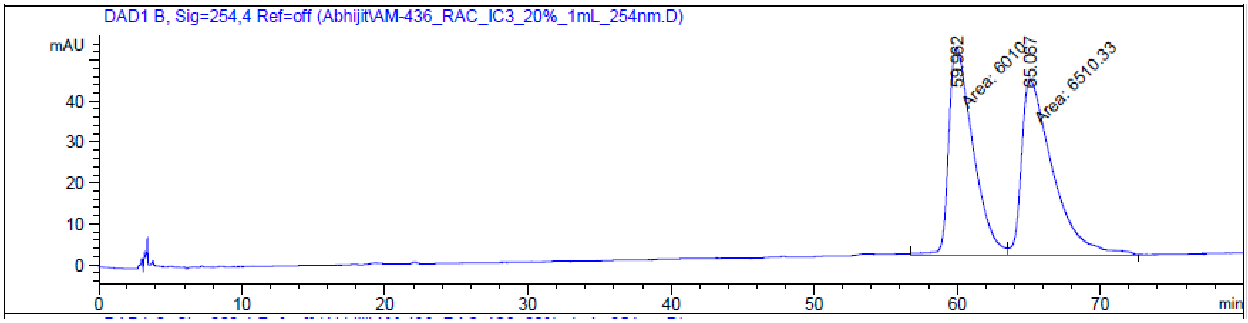

Peak #	RetTime [min]	Type	Width [min]	Area [mAU*s]	Height [mAU]	Area %
1	59.962	MF	1.9682	6010.00000	50.89263	48.0019
2	65.067	FM	2.5269	6510.32617	42.94024	51.9981

Authentic Sample, >99% ee: HPLC (IC-3, Isopropanol/hexanes = 20/80, flow rate = 1 mL/min, I = 254 nm), t_R = 55.0 min.

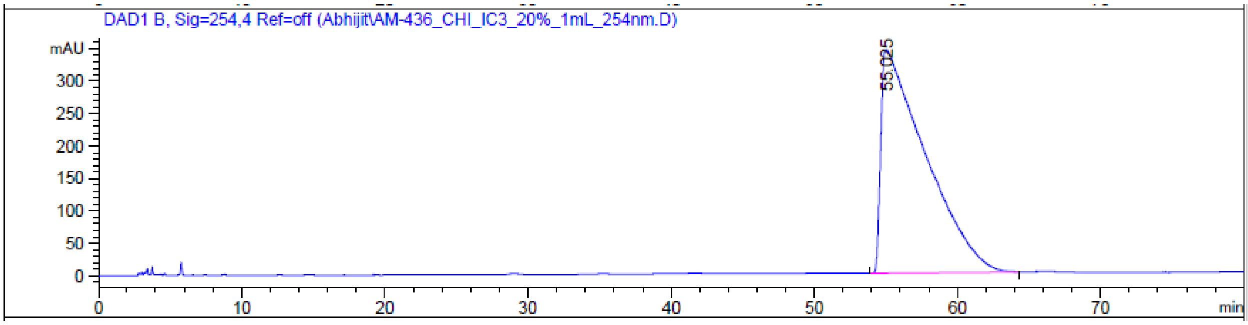

Peak #	RetTime [min]	Type	Width [min]	Area [mAU*s]	Height [mAU]	Area %
1	55.025	BB	2.7135	7.44694e4	343.74039	100.0000

Synthetic Scalemic Sample

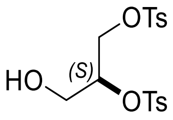

Specific Rotation: $[\alpha]_D^{21}$ = –5.77 (c = 0.7 g/100 mL, $CHCl_3$) (synthetic scalemic sample)

Racemic Sample: HPLC (IC-3, Isopropanol/hexanes = 20/80, flow rate = 1 mL/min, I = 254 nm), t_R = 65.7 min, 71.8 min.

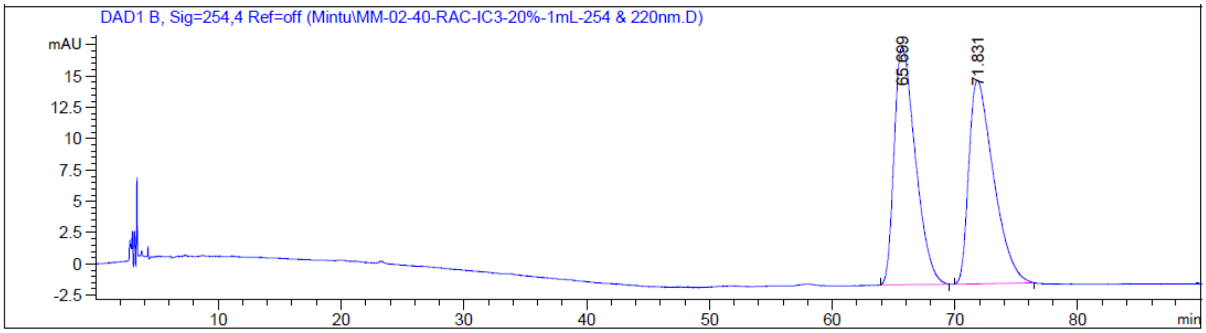

Peak #	RetTime [min]	Type	Width [min]	Area [mAU*s]	Height [mAU]	Area %
1	65.699	BB	1.4186	2279.39502	18.93259	50.0634
2	71.831	BB	1.6456	2273.61890	16.23780	49.9366

Scalemic Sample, -37% ee: HPLC (IC-3, Isopropanol/hexanes = 20/80, flow rate = 1 mL/min, I = 254 nm), t_R = 64.6 min, 69.6 min.

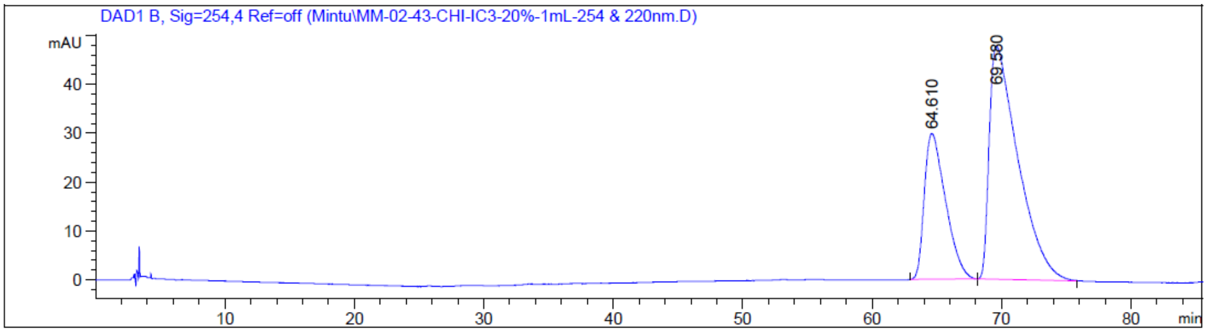

Peak #	RetTime [min]	Type	Width [min]	Area [mAU*s]	Height [mAU]	Area %
1	64.610	BB	1.5080	3402.68262	29.84715	31.6056
2	69.580	BB	1.9825	7363.39307	47.65899	68.3944

$CDCl_3$, 400 MHz

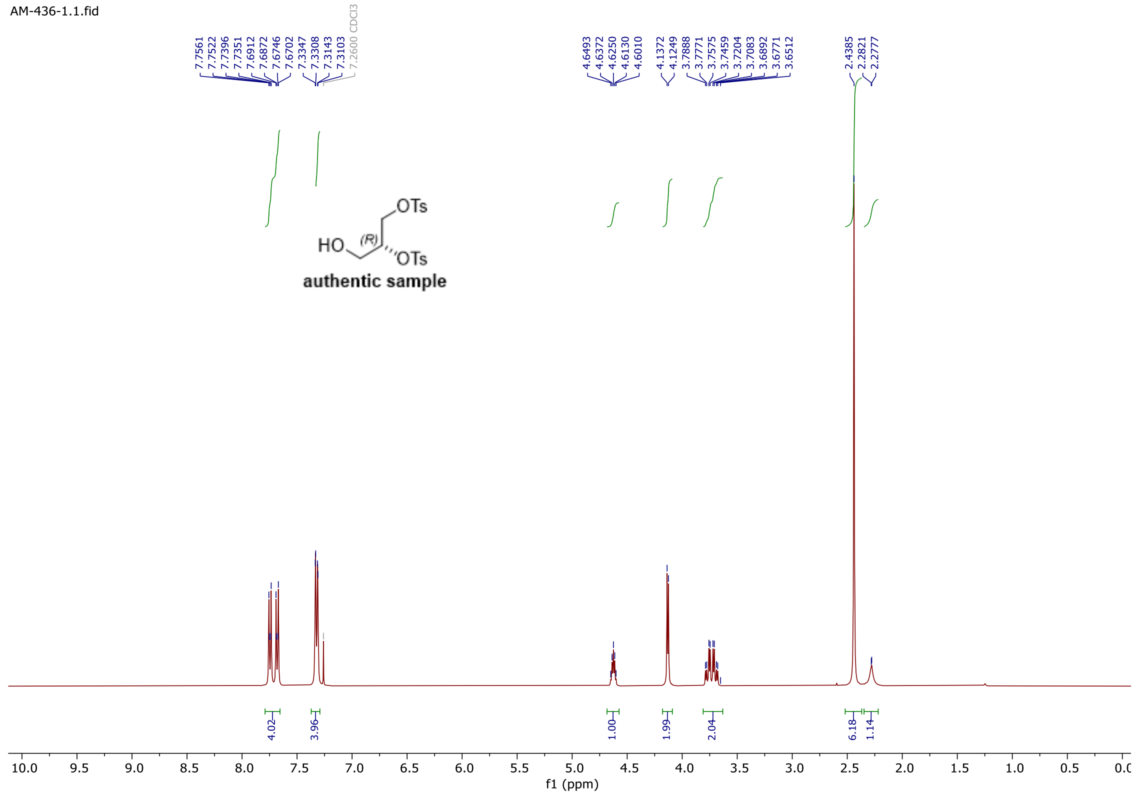

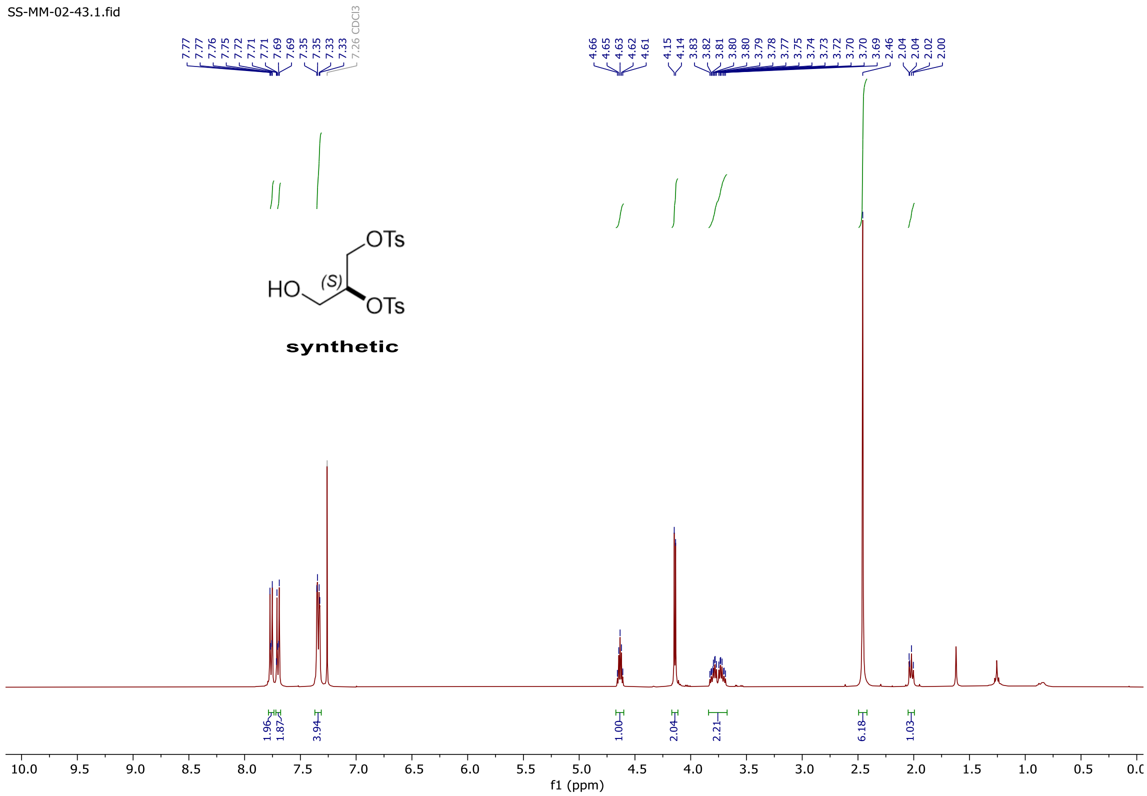

Supplement: Supporting Information [file NIHMS2165358-supplement-Supporting_Information.pdf]
